# Bacterial structure and dynamics in mango (*Mangifera indica*) orchards after long term organic and conventional treatments under subtropical ecosystem

**DOI:** 10.1038/s41598-021-00112-0

**Published:** 2021-10-15

**Authors:** Govind Kumar, Archana Suman, Shatrohan Lal, R. A. Ram, Pankaj Bhatt, Ghanshyam Pandey, Parul Chaudhary, Shailendra Rajan

**Affiliations:** 1 ICAR-Central Institute for Subtropical Horticulture (CISH), Lucknow, India; 2 Division of Microbiology, IARI, New Delhi, India; 3 SCAU, Integrative Microbiology Research Centre SCAU, Guangzhou, China; 4 Department of Microbiology, GB Pant University of Agriculture and Technology, Pantnagar, India

**Keywords:** Biotechnology, Chemical biology, Ecology, Microbiology, Ecology, Environmental sciences

## Abstract

This study explores the comparative effect of conventional and organic treatments on the rhizosphere microbiome of *Mangifera indica* cv. Dashehari. The long-term exposures (about 20 years) were monitored under a subtropical ecosystem. Based on plant growth properties and acetylene reduction assay, 12 bacterial isolates (7 from G1-organic and 5 from G2-conventional systems) were identified as *Pseudomonas* and *Bacillus* spp. In the conventional system, *dehydrogenase* activity significantly decreased (0.053 µg TPF formed g^−1^ of soil h^−1^) and adversely affected the bacterial diversity composition. In comparison, organic treatments had a good impact on *dehydrogenase* activity (0.784 µg TPF formed g^−1^ of soil h^−1^), *alkaline phosphatase* (139.25 µg PNP g^−1^ soil h^−1^), and bacterial community composition. The Metagenomics approach targeted the V3 and V4 regions to see the impact in the phylum, order, family, genus, and species for both the treatments. Results showed that phylum *Acidobacteria* (13.6%), *Firmicutes* (4.84%), and *Chloroflexi* (2.56%) were dominating in the G2 system whereas phylum *Bacteroides* (14.55%), *Actinobacteria* (7.45%), and *Proteobacteria* (10.82%) were abundantly dominated in the G1 system. Metagenome sequences are at the NCBI-GenBank sequence read archive with SRX8289747 (G1) and SRX8289748 (G2) in the study PRJNA631113. Results indicated that conventional and organic conditions affect rhizosphere microbiome and their environment.

## Introduction

The microbiome of the rhizosphere can be used as a bio-indicator of stress caused by anthropogenic activities used in agricultural applications^1^. The rhizosphere´s microbial community structure has been studied by culturable practices, including isolation and characterization of the indigenous bacterial strains in laboratory^2^. In recent years, the next-generation sequencing (NGS) technology has evolved and intensified to analyze soil microbiome diversity and use it to identify bio-indicators. The NGS was found limited to the microorganisms cultivated *in-vitro*, while microorganisms cannot be cultured due to complex nutrient requirements^3^. The comparative effectiveness of organic and conventional agricultural practices is the most critical challenge in modern time and determines the environmental impact of organic and conventional systems. At present, organic farming is considered as cost-effective, eco-friendly, and sustainable approach and found to have a low detrimental impact on the environment. On the other hand, conventional practices may have negative consequences due to the extensive use of pesticides and chemical fertilizers in the soil and water system^4–6^.

In horticultural fruit crops like mango (*Mangifera indica* cv. Dashehari), the productivity in the conventional system is usually higher than in organically treated orchards, while the quality of fruit is low and the environment is debated^7–9^. Understanding the effects of different treatments on rhizosphere microbial diversity has been a subject of interest but due to complex ecosystem and microbial cultivation practices limit the study. Microbiomes found in organic and conventional orchards play a significant role in plant vigor, either synergistically or negatively. However, the role of organic and conventional treatments has not been elucidated in horticultural crops, especially in mango crops. Some of the previous researchers’ studies indicated that, in traditional treatment, rhizosphere microbial respiration is reduced by 36–46% in forest trees^10,11^. In comparison, organic treatment enhanced rhizosphere microbial diversity and soil organic matter^12^.

Maillard and Angers^13^, investigated that the soil organic matter, nutrient availability, and microbial diversity were significantly enhanced by organic treatment. The organic and inorganic fertilizer sources identified as the cause for shaping the rhizosphere microbiome of wheat crop and microbial activities were highly responsive to both the treatments^11^. Microbial phylum like *Acidobacteria* and *Nitrospirae* were highly abundant in inorganic treatment, and this alteration in microbial dynamics and reduction in diversity resumed by only organic treatment^14,15^. The effects of such treatments on rhizosphere bacterial structure and their actions in subtropical climate are still unclear. To address this, we have focused on the culturable and metagenomic approach. We can cultivate only about 0.3% of the total microbial population using culturable methods^16^. Still, this limitation can be overcome using metagenomic approaches in which the highly conserved 16S rDNA region (V3–V4) is targeted for bacterial diversity analysis. All research data obtained using metagenomic analysis is not very consistent with the received data using a culturable approach^17^.

This study, investigated the comparative effects of organic and conventional agricultural practices on mango rhizosphere microbial diversity for up to 20 years. These research objectives were conducted as follows; (i) to determine the direct impact of organic and conventionally treated mango rhizosphere on microbial enumeration, PGP (Zn, P, K solubilization, etc.), Acetylene reduction assay, and soil enzyme activities by using culturable approach (ii) to assess the targeted identification (metagenomic analysis) of bacterial diversity of both treatments under subtropical climate by focusing on V3 and V4 region of 16SrRNA gene (iii) ascertained the overall impact of treatment by using bioinformatics, heat map, PCA and theoretical analysis.

## Conclusion

In addition to a culturable approach, metagenomic studies have investigated the comparative rhizosphere microbiome of organic and conventionally managed mango orchards. With the long term exposure to conventional treatments, bacterial groups *Acidobacteria, Firmicutes,*and *Chloroflexi* were dominating. In contrast, high plant growth promotory groups like *Proteobacteria, Actinobacteria,*and *Bacteroides* were in abundance in organically managed orchards. Soil enzymes, ARA activity and, plant growth promotion properties were observed higher in organic treatment. Based on the obtained results, the organic treatment favourably shifts the rhizosphere microbiome compared to conventionally treated under solely chemical fertilization. Such impacts mainly were considered to the quality of organic treatments. More minor differences may observe when an integrated approach is provided to the conventionally managed rhizosphere soil.

## Results

### Bacterial strains isolation and identification

**Figure 1:**
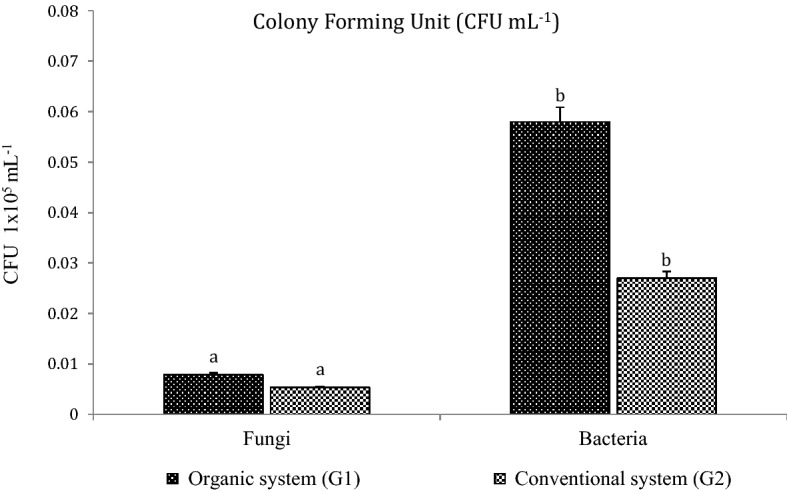
Comparative microbial enumeration of organic and conventional treated mango rhizosphere soil the CFU mL^−1^ of selected samples showing growth of fungus and bacterial populations under two different treatments i.e. organic and conventional. The results are the average of five replicates (n = 5), with bars representing standard error. Significant differences based on the analysis variance (ANOVA) are shown by different letters above the error bars, followed by the post hoc DMRT test (*p* ≤ 0.05) using the software SPSS.

Fifty six bacterial cultures were isolated from both management systems (G1 and G2) of mango orchards (rhizosphere) at CISH, Lucknow, India. Isolation of microorganisms using spread plate methods revealed that the Nutrient agar medium had the highest number of colony appearances compared to the Rose Bengal Agar medium. Microbial enumeration showed organic system enriched with higher bacterial and fungal population than conventional system (Fig. 1). From organic system, thirty seven bacteria were isolated out of which, twenty-three isolates were (G^+^), and fourteen were (G^−^). While, in the conventional system, nineteen bacteria were isolated, out of which fifteen were (G^+^) and four were (G^−^) isolates.

### Plant growth promotion properties

For plant growth promotory properties out of fifty-six bacterial isolates total, ten bacterial cultures (2, 3, 4, 8, 15, 23 and 31) from the organic system showed positive results for phosphate solubilization. In contrast, three bacterial cultures (I1, I8 and I9) from the inorganic system (conventional system) showed positive phosphate solubilization in Pikovaskya’s agar medium. For siderophore production, bacterial cultures (2, 3, 4, 8, 12 and 26) from the organic system showed positive results, while four bacterial cultures (I1, I6, I8 and I9) inorganic system showed positive results. Bacterial cultures (2, 3, 4 and 8) from the organic system showed positive results for K-solubilization, while five bacterial cultures (I1, I2, I7, I8 and I9) from the inorganic system showed positive K-solubilization. A total of ten isolates (7 from organic and 3 from the inorganic system) possessed Zn-solubilizing activity. The test isolated from the organic system showed better Zn (ZnO), Zn_3_ (PO_4_)_2,_ and (ZnCO_3_) solubilization as compared to test culture isolated from the inorganic system (Supplementary S1.8).

### Acetylene reduction assay (ARA)

Results from acetylene reduction assay showed in aerophilic condition, bacterial isolates 1, 3, 4 (from organic treated soil) and I1, I8 and I9 (conventional system) showed 134.8, 37.70, 36.73, 13.15, 16.70 and 12.87 ppm of ethylene tube^−1^ h^−1^, respectively. In case of microaerophilic condition, bacterial isolates 4, 9, I9 showed 24.17, 19.14, and 12.71 ppm ethylene, respectively. Results indicate possible use of these bacterial isolates as a bioinoculant agent for horticultural crops, especially mango and other subtropical climate fruit crops.

### Soil enzymatic study

**Figure 2:**
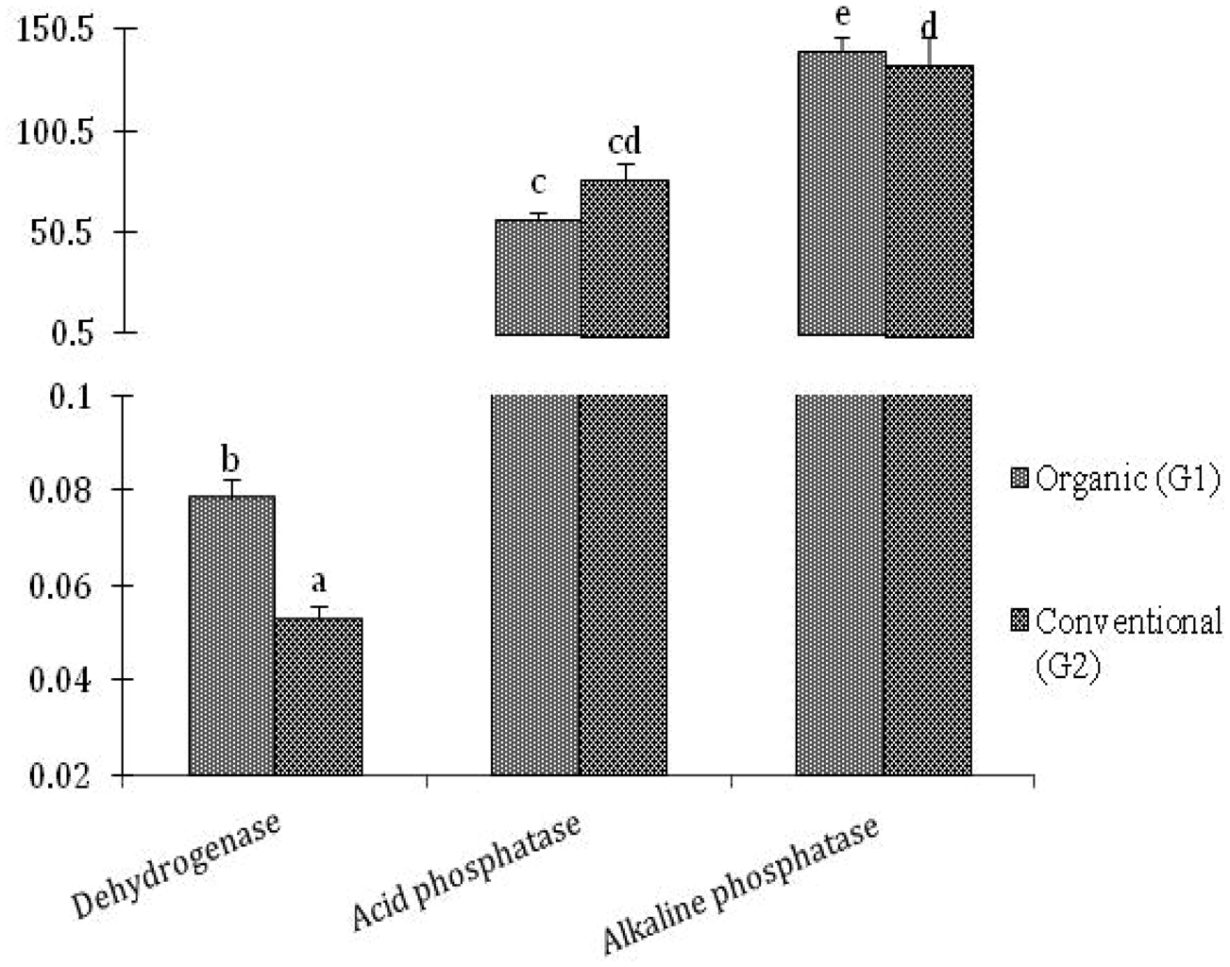
Comparative soil enzymes activities of conventional and organic treated mango rhizosphere soil the *dehydrogenase*, *acid phosphatase* and *alkaline phosphatase* activities were showing in µg TPF formed g^−1^ of soil h^−1^ and µg PNP g^−1^ soil h^−1^ respectively. The results are the average of five replicates (n = 5), with bars representing standard error. Significant differences based on the analysis variance (ANOVA) are shown by different letters above the error bars, followed by the post hoc DMRT test (*p* ≤ 0.05) using the software SPSS.

The soil enzymatic activity in the organic system (G1) showed better *dehydrogenase* activity than the conventional system (G2). For both methods, *alkaline phosphatase* almost showed similar activity (at pH 11), while in the case of *acid phosphatase* showed better activity in the inorganic system (G2) as compared to the organic system (G1) at pH level 6.5 (Fig. 2). The *dehydrogenase* enzyme oxidizes the organic matter, and it belongs to the *oxidoreductase* type of enzyme. In the process of respiration of soil microorganisms, the *dehydrogenase* enzyme facilitates the transfer of protons and electrons from the substrate to the acceptor. It was significant to observe that the *dehydrogenase* activity was higher in organic treated soils (0.784 µg TPF g^−1^ h^−1^) than in conventional system (0.053 µg TPF g^−1^ h^−1^).

### Alpha biodiversity with samples and rarefaction curves

**Figure 3:**
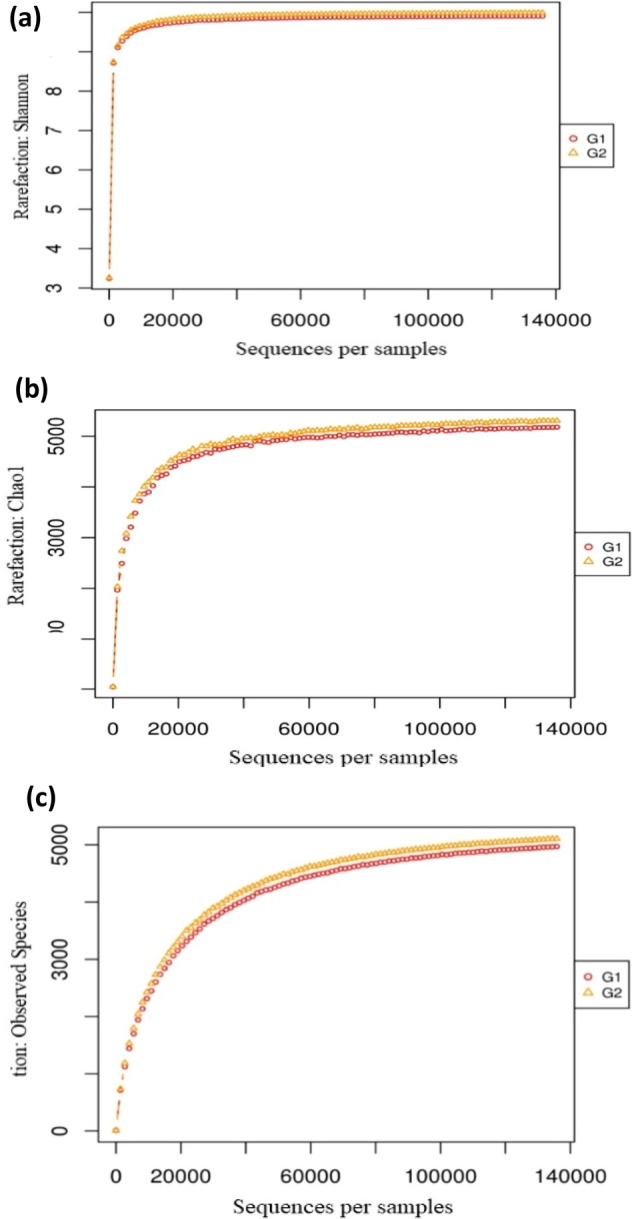
Shanon (**a**), Chao1 (**b**) curves and observed species (**c**) obtained for the samples (G1 and G2).

In this segment, by measuring Shannon, Chao1, and observed species metrics, we analyze the microbial diversity within the samples. The chao1 metric measures the richness of the ecosystem, while the Shannon metric is the formula for calculating reported OTU abundances and accounts for both prosperity and equality. The rarefaction curve is provided in Fig. 3 for each metric. Using QIIME software, the metric measurement was done. The impact of both treatments on the microbial complexity and abundance in the sample was also revealed using the Shannon diversity Index (depicting richness and evenness) and Chao 1 representing only richness. Shannon’s diversity index of the bacterial community in the treatment (G1 and G2) was 8.06 and 8.12. The Simpson index in ecology is used to quantify biological diversity in a region, which was also nearly similar in both the treatments. Chao 1 richness estimator showed an increase in species richness. Rarefaction analysis conducted to confirm species richness revealed a difference in the number of reads and OTUs between the samples. The Rare fraction curve had a similar pattern for both samples and showed an impact on the bacterial population in the experiment (Fig. 3a–c).

### Bacterial diversity analysis at phyla level

**Figure 4:**
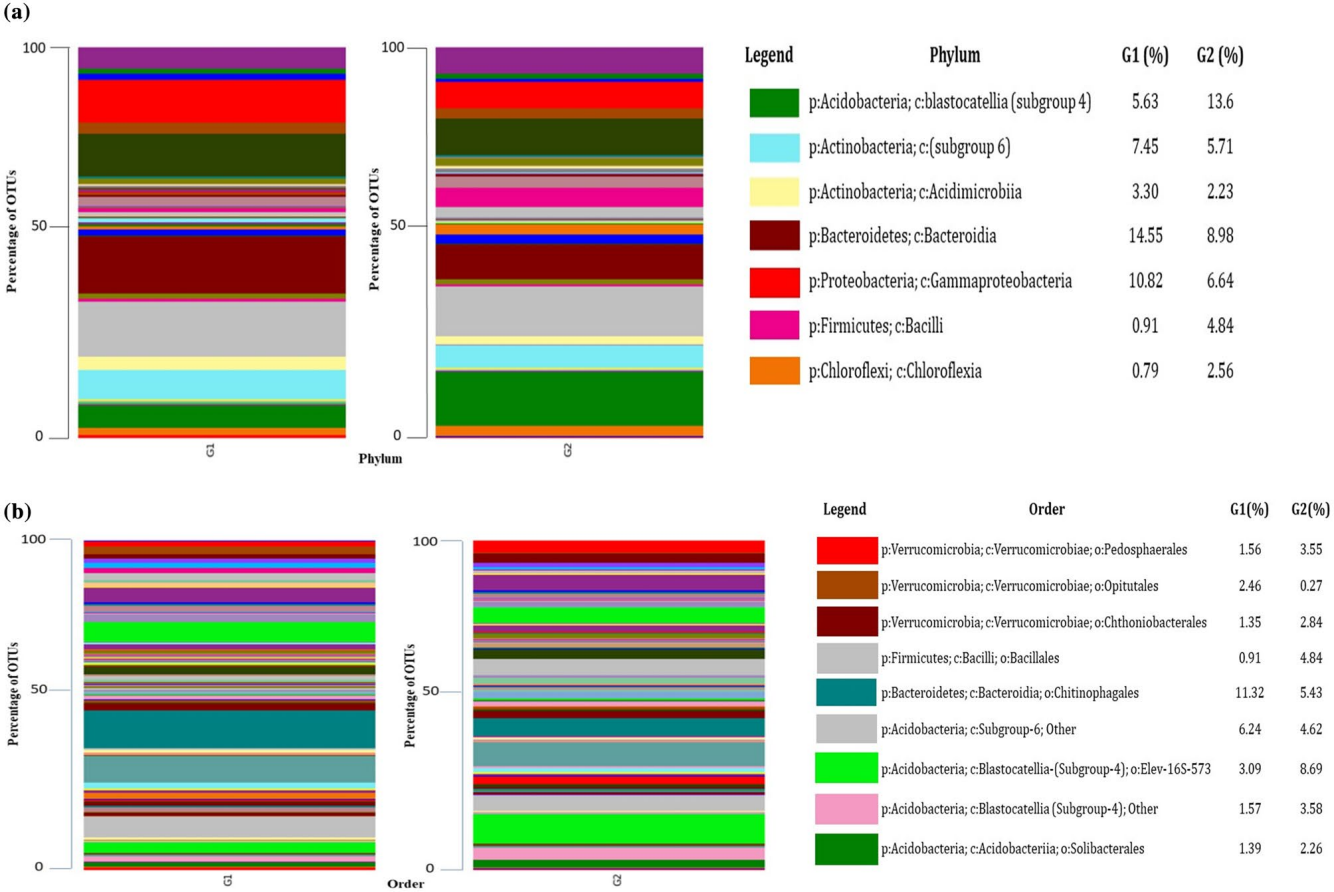
Comparative microbiome (a-phylum and b-order) analysis of organic (G1) and conventional (G2) treated mango orchards soil by using metagenomic (V3 and V4 region) approach.

Taxonomic study of the 16S rRNA gene amplicon reads yielded seven classifiable bacterial phyla. Six phyla, namely *Acidobacteria, Actinobacteria, Bacteroides, Proteobacteria, Firmicutes, and Chloroflexi* were dominant in both the systems. The Organically treated soil (G1) sample harbored a higher percentage of *Bacteroidetes* (14.55%), *Actinobacteria* (7.45%), and *Proteobacteria* (10.82%) as compared to conventional treatment (G2) 8.98%, 5.71%, and 6.64%, respectively. However, phylum *Acidobacteria*(13.6%), *Firmicutes*(4.84%), and *Chloroflexi* (2.56) were higher abundance in conventional treatment as compared to the organic treatment, which showed the same phyla with lesser quantity, i.e., 5.63%, 0.91%, and 0.79% respectively (Fig. 4a).

### Distribution of bacterial community at order level

The bacterial orders in both systems were diversified. The most abundant orders in organic and conventional systems were *Chitinophagales* (Organic-11.32%, Conventional-43%), *Elev-16S-573* (Organic-3.09%, Conventional-8.69%), *Pedosphaerales* (Organic-1.56%, Conventional-3.55%), *Opitutales* (Organic-2.46%, Conventional-0.27%), *Chthoniobacterales *(Organic-1.35%, Conventional-2.84%), *Bacillales* (Organic-0.91%, Conventional-4.84%) and *Solibacterales* (Organic-1.39%, Conventional-2.26%) (Fig. 4b).

### Bacterial community distribution at family level

**Figure 5:**
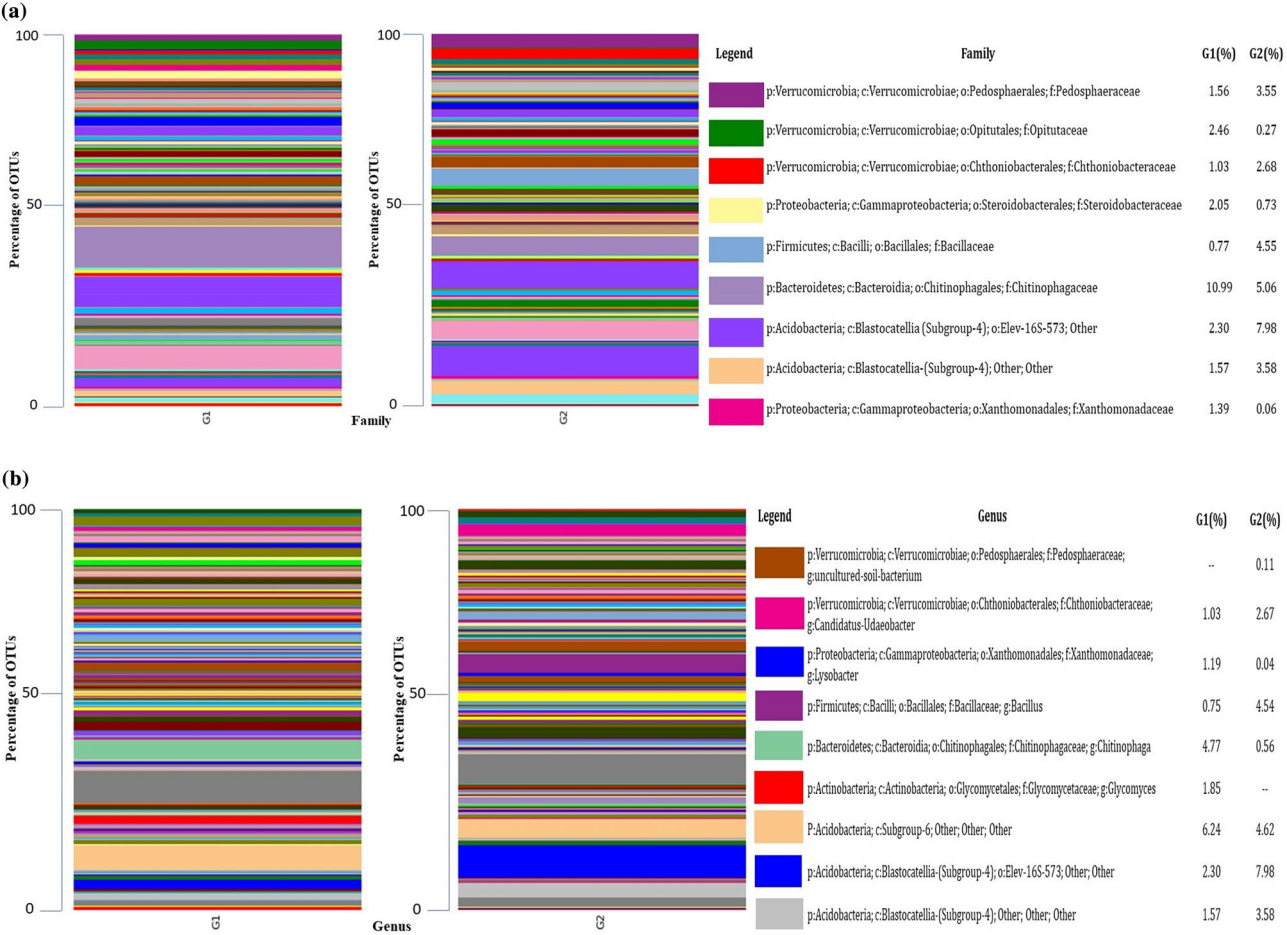
Comparative microbiome (a-family and b-genus) analysis of organic (G1) and conventional (G2) treated mango orchards soil by using metagenomic (V3 and V4 region) approach.

Bacterial family members were identified and enriched including *Pedosphaeraceae* (O-1.56%, C-3.55%)*, Opitutaceae* (O-2.46%, C-0.27%)*, Chthoniobacteraceae* (O-1.03%, C-2.68%)*, Steroidobacteraceae* (O-2.05%, C-0.73%)*, Bacillaceae* (O-0.77%, C-4.55%)*, Chitinophagaceae* (O-10.99%, C-5.06%)*, and Xanthomonadaceae* (O-1.39%, C-0.06%) and other families (Fig. 5a).

### Bacterial community distribution at the genus level

Comparative abundance of unidentified genus in organic system were uncultured soil bacterium, *Glycomyces, Chitinophaga, Lysobacter, Udaeobacter, Bacillus* (not detected, 1.85%, 4.77%, 1.19%,1.03% and 0.75% respectively) whereas same genus-group were observed in conventional system with different percentage i.e., 0.11%, not detected, 0.56%, 0.04%, 2.67%, 4.54% respectively (Fig. 5b).

### Bacterial communities at species level

**Figure 6:**
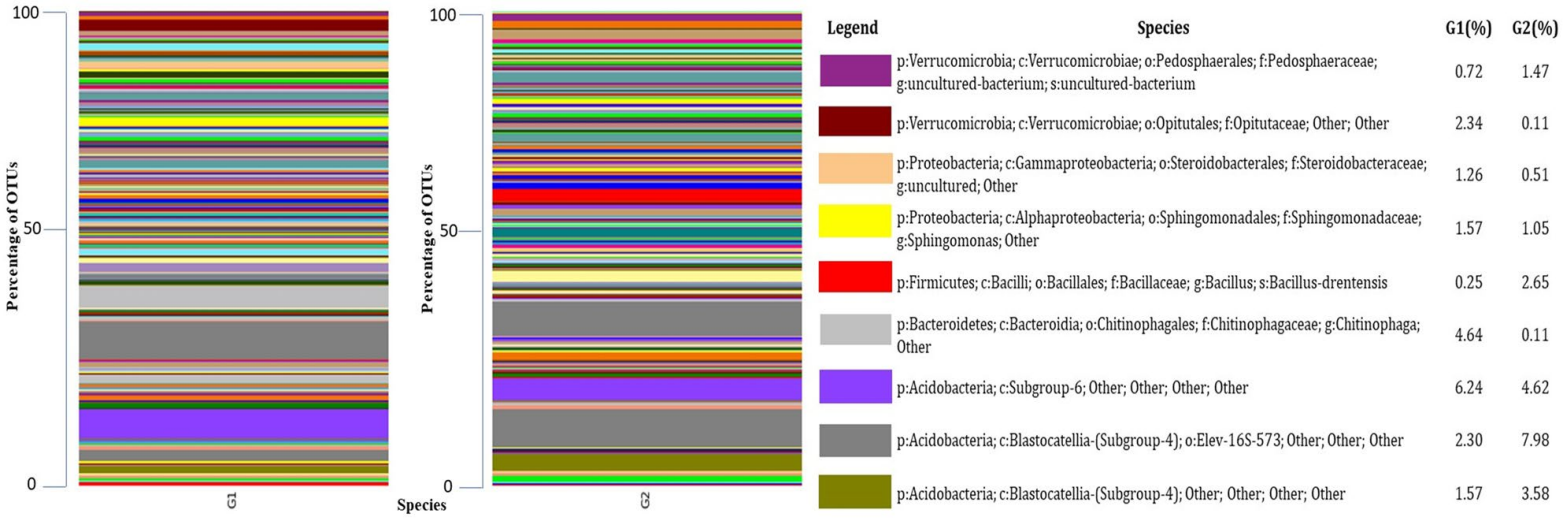
Comparative microbiome (Species) analysis of organic (G1) and conventional (G2) treated mango orchards soil by using metagenomic (V3 and V4 regions) approach.

Because most of the species were unidentified and uncultured bacterium based on relative abundance, they could not be assigned a species name in either sample. Few species are identified in both systems, like *Sphingomonas sp.* (O-1.57%, C-1.05%), *Bacillus drentensis* (O-0.25%, C-2.65%), and *Chitinophaga sp.* (O-4.64%, C-0.11%) (Fig. 6).

### Heat map and PCA analysis

**Figure 7:**
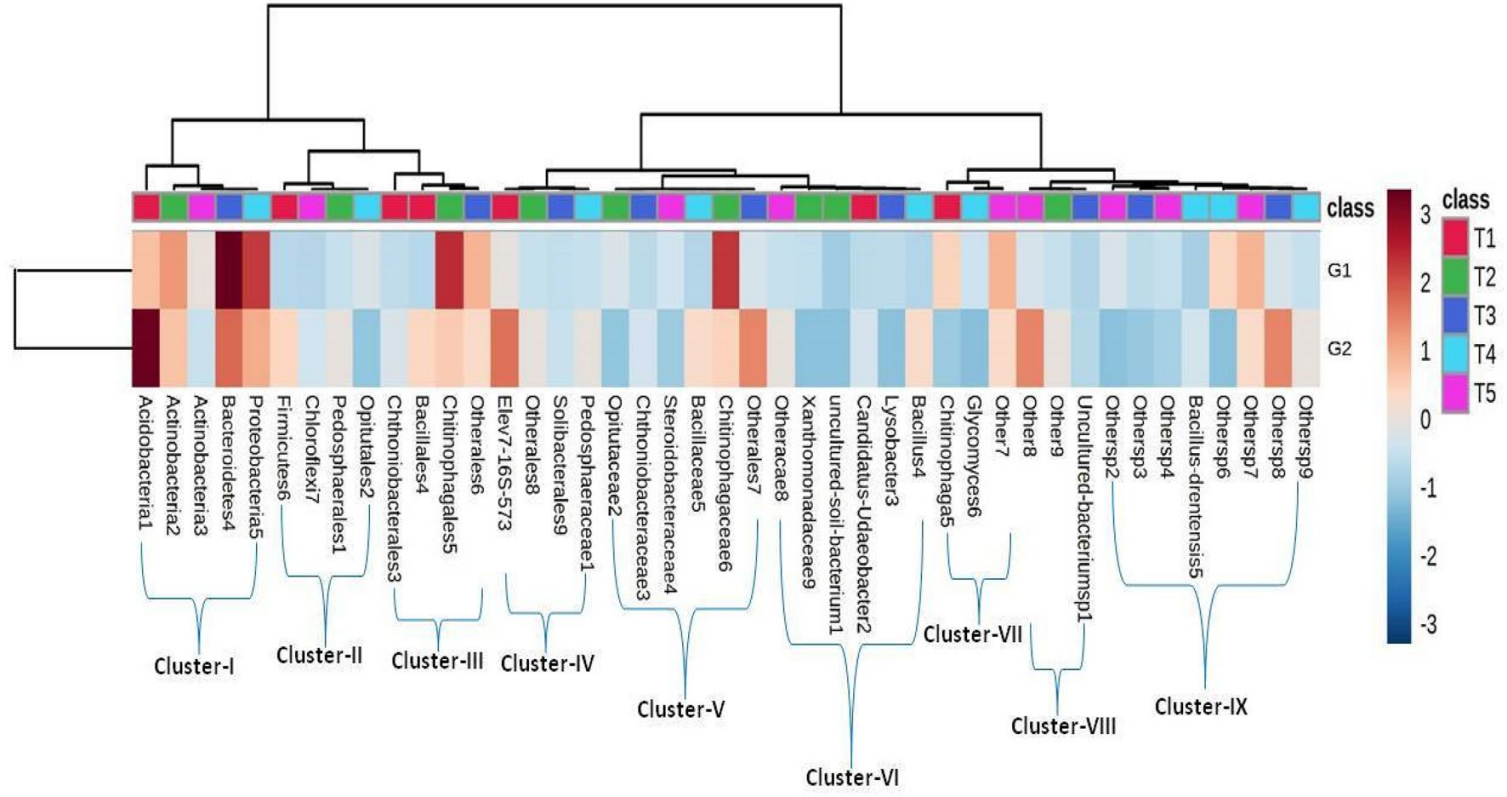
Comparative (G1 organic and G2 conventional) heat map of dominant microbial diversity and their clusters in terms of T1 (phylum), T2 (order), T3 (family), T4 (Genus) and T5 (Species).

**Figure 8:**
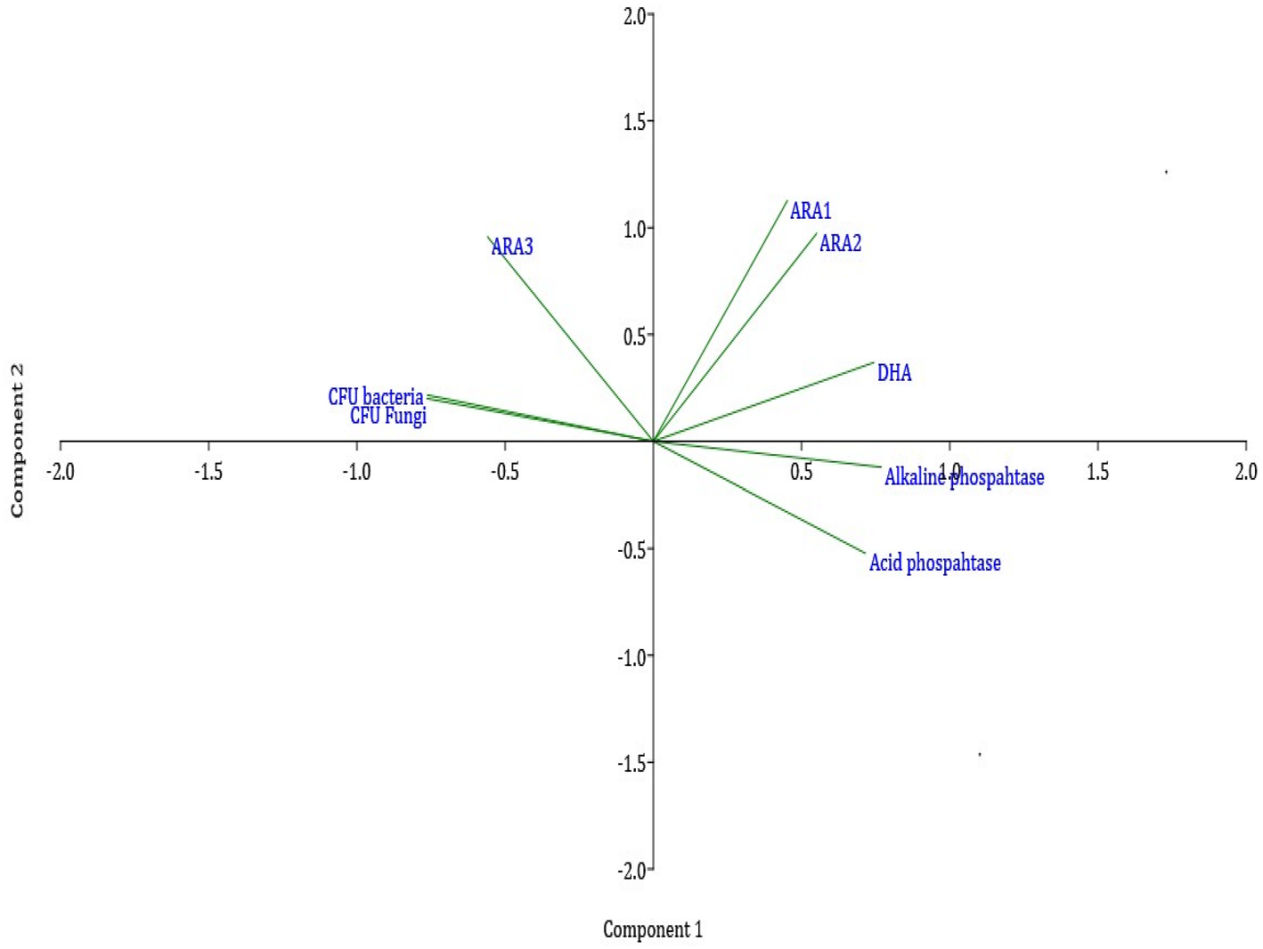
PCA analysis of different parameters for organic and conventional systems.

Under long-term exposure of organic and conventional treatments, a microbial shift was observed in the rhizosphere microbiome of mango orchards. Based on percent abundance, nine different microbial genera *Acidobacteria, Actinobacteria, Bacteroidetes and Proteobacteria* formed Cluster I. While, *Firmicutes, Chloroflexi and Opitutales* were abundances in cluster II. Cluster III includes *Chitinobacterales, Bacillales, Chitinophagarales and Otherales* genera. Whereas cluster IV (*Elev7-16S-573, Otherales, Solibacterales and Pedobacteriaceae*), cluster V (*Opitutaceae, Chitnobacteraceae, Bacillaceae, Chitinophagaceae and Otherales*), cluster VI (*Xanthomonadaceae, Uncultured soil bacterium, Candidatus-Udaeobacter, Lysobacter and Bacillus*), cluster VII (*Chitinophaga, Glycomyces and Other*), cluster VIII (*Uncultured bacterium and Others*) and cluster IX (*Bacillus drentensis and Others*) (Fig. 7). The cluster I observed with the highest abundance was closely related to clusters II and III. Cluster IV to IX created large groups and is distantly related to cluster I to III of the microbial groups in organic and conventional systems (Fig. 7). In the organic system (G1), microbial groups like *Proteobacteria, Actinobacteria, Bacteroidetes,* and *Opitutaceae* were largely dominated and provided benefits to the mango rhizosphere in terms of nutrient availability, plant growth promotion, and protection against biotic and abiotic stress. Phylum *Proteobacteria and Actinobacteria* are closely linked with the rhizosphere and identified as potential PGPR. *Acidobacteria* and *firmicutes,* on the other hand, were dominated primarily by conventional systems and serve as a bio-indicator of anthropogenic stress caused by excessive chemical fertilizer application. Undefined *Acidobacteria* is oligotrophic in nature and considered as an indicator of low organic carbon and acidic environment. To desire higher productivity, the indiscriminate use of chemical fertilizers or pesticides in conventional systems leads to low nutrient availability, microbial shift, less PGPR, and developing the environment for *Acidobacteria, Firmicutes* and *Chloroflexia* group of microorganisms. Principal component analysis (PCA) was performed for both systems (organic-component 1; conventional-component 2). The total variables of principal component analysis were the percentage of different parameters such as *alkaline phosphatase*, *acid phosphatase*, DHA, Acetylene reduction assay (ARA1, ARA2, ARA3), and CFU mL^−1^ (bacteria and fungi). The results of PCA yielded two components that explained 100% of the total variance in the data and had an Eigen value of 6.1 for component 1. In contrast, 1.8 for component 2 and together they described 100% of the total variance in the data (Fig. 8). In the organic system, the loading factor with score plot indicates that component-1 is positively associated with DHA, ARA1, ARA2, *alkaline phosphatase*, *acid phosphatase* while negatively correlated with CFU ARA3 activity. Component-1 explains the 76.42% variance of the experimental data, while component-2 explains 23.58%. The second component (PC2) represents the positive association with DHA, ARA1, ARA2, ARA3 activity, and CFU while negatively correlated with *alkaline phosphatase* and *acid phosphatase*. In the conventional system, the loading factor with score plot indicates that component-1 is positively associated with single variable *acid phosphatise* while negatively correlated with DHA, ARA1, ARA2, ARA3, CFU, and *alkaline phosphatase* activity. The second component (PC2) of the conventional system showed positive association with DHA, ARA1, ARA2, ARA3 activity, and CFU, while the negative association with *alkaline phosphatase* and *acid phosphatase.*

## Discussion

By using the culturable approach we find significant differences in bacterial and fungal populations after long term (> 20 years) organic and conventional treatments in mango orchards. Owning to organic treatment increases the species richness, decreases evenness, dispersion, and shifting the structure of the soil microbiome than conventionally managed soil^18^. According to Hartmann et al.^19^, Organic application (FYM) is identified as a significant contributor to microbial diversity by changing factors like composition, dispersion reduction, higher richness, and reducing the evenness of the soil microbiome. Many organic compounds are carried by organic treatment with indigenous microbiota that may inhabit the soil for an extended period of time. The higher enrichment of bacteria in the organic system may also be represented as an indicator of the organic fertilizer inputs. The reduction in microbial population in conventional system is mainly due to stress posed by the indiscriminate and long exposure of chemical fertilizers, fungicides, pesticides, and herbicides application^20^. Total microbial diversity reduced drastically due to excessive agrochemical applications. Such chemical applications can eliminate certain microbial groups and retain the few microbes acclimatized to grow in stressful conventional farming^21–23^.

In our study, rhizosphere microbial community impacted both positively and negatively in some extent due to release of different kinds of root exudates like sterol, fatty acids, etc., by the plant system for microbial enrichment and proliferation. In contrast to this study, most studies focused on the bulk soils compared to the rhizosphere soil microbiome^20^. In the organic system we observed, a congenial environment for microbes with higher PGPR activity, including siderophore production, P, Zn, K- solubilization, etc., than conventional ones. In traditional systems, stress and low microbial activity lead to the depletion of essential nutrients to survive and multiply microbial diversity and plant pathogens^24^. On the other hand, organic treatment provides a rich medium to support high microbial activity and may also help in the proliferating diverse microbial populations in the rhizosphere^25^.

Soil *dehydrogenase *is the part of rhizosphere microbiome management system as organic treatment showed positive influence on DHA activity. As DHA production was directly influenced by orchard ground-floor management system^26^. In general, the functions of enzymes in the soil system are linked with the substances of organic matter.

According to Mandal et al.^27^, for higher production of microbial enzymes, the balanced use of nutrients and manure concentrations were essential components that lead to enhanced microbial biomass. *Dehydrogenase* activity with the application of organic sources may be related to greater soil abundance of microbes and substrates^28^. The composition (quality) and quantity of organic matter incorporated into the soil lead to higher *dehydrogenase* activity, as Pramanik et al.^29^ show. Soil enzymatic activities, including *dehydrogenase* (DHA), acid, and *alkaline phosphatase,* directly tracked the organic matter pattern in the soil. Higher *dehydrogenase* and *alkaline phosphatase* were observed in organic system rhizosphere soil compared to the conventional system. In contrast, due to acidification caused by chemical fertilizers, *acid phosphatase* was higher in the conventional system. Building up organic carbon over the last 20 years of organic treatment has resulted in microbial activity and organic matter decomposition.

For the unculturable study, the metagenomic sequencing was used to identify specific microbial shifts at the phylum, order, family, genus, and species that harbor a new impact on managing the rhizosphere microbiome of proliferating beneficial and reducing pathogenic organisms. Our study focused on the effect of agricultural practices on rhizosphere microbial diversity, analyzing microbial properties, soil enzymes, etc. have major significance in the light of higher sustainable productivity. This study revealed that nutrient-rich treatments like FYM, vermicompost, etc. enhanced the richness by promoting a copiotrophic environment and microbial niche that reduced the evenness. In contrast, the conventional treatment led to an oligotrophic climate, reduced nutrient level, and richness while enhancing evenness. Few studies found no noticeable differences in alpha microbial diversity when treated with organic and conventional systems^18^.

It has also been reported that in organic treatment organic farming, bacterial evenness increased for the first few years but then decreased over long-term treatment^30^. According to Hartmann and Widmer^31^, the effect of conventional and organic practices in microbial diversity was difficult to conclude. Some recent studies on metagenomic studies revealed that microbial diversity was enhanced in organic systems^15,32^, while unaffected by the richness in a meaningful manner^32,33^. In contrast to this, our result contravened with the effects observed in previous reports investigating that soil microbiome richness decreased and evenness increased significantly in organic treatment. Our study supports the results obtained in a few earlier reports by Hartmann et al.^34^, van Diepeningen et al.^30^ and Ge et al.^35^ concluded that organic treatment enhanced the microbial richness and reduced evenness.

According to Hartmann et al.^34^, the organic treatment positively impacted microbial diversity composition and the α-diversity parameters after pyrosequencing data analysis. In organic treatments, the richness of microbial diversity was increased significantly^30,34,35^. In organic treatment, the increased richness can positively affect the PGP rhizobacteria and crop production^2,15,33,36^. The organic treatment was mainly responsible for significant shift or changes in the predominance of PGP rhizobacteria and other microbes^33,34^. In this, the ratio of PGP bacteria may be greater in organic treatment than in conventional, and it was justified in the long term of exposure with stable conditions^37^.

The indications for taxonomic analyses observed at the phylum level, i.e., *Proteobacteria* and *Actinobacteria*, lead to dominance in the organic systems, while *Acidobacteria* were in abundance on conventionally managed farmlands^36,38^. In addition, the high quantity of PGP bacteria, including *Pseudomonas, Stenotrophomonas, Bradyrhizobium*, *Mesorhizobium*, *Rhodoplanes*, etc*.* comes under *Proteobacteria* in organic treatment system^2,15,33,36^.

In our study, results of culturable approach and V3 and V4 region metagenome analysis revealed higher bacterial abundance in organically treated soil (G1); while in the conventional treatment (G2), relatively low microbial abundance was found. These results may connect with the variation in the spaces in the soil's pore structure created by organic treatment. Organic soils typically have small isolated and connected channels with large pores that promote bacterial growth and fungal population growth, creating an ideal environment for developing an effective rhizosphere microbiome.

**Figure 9:**
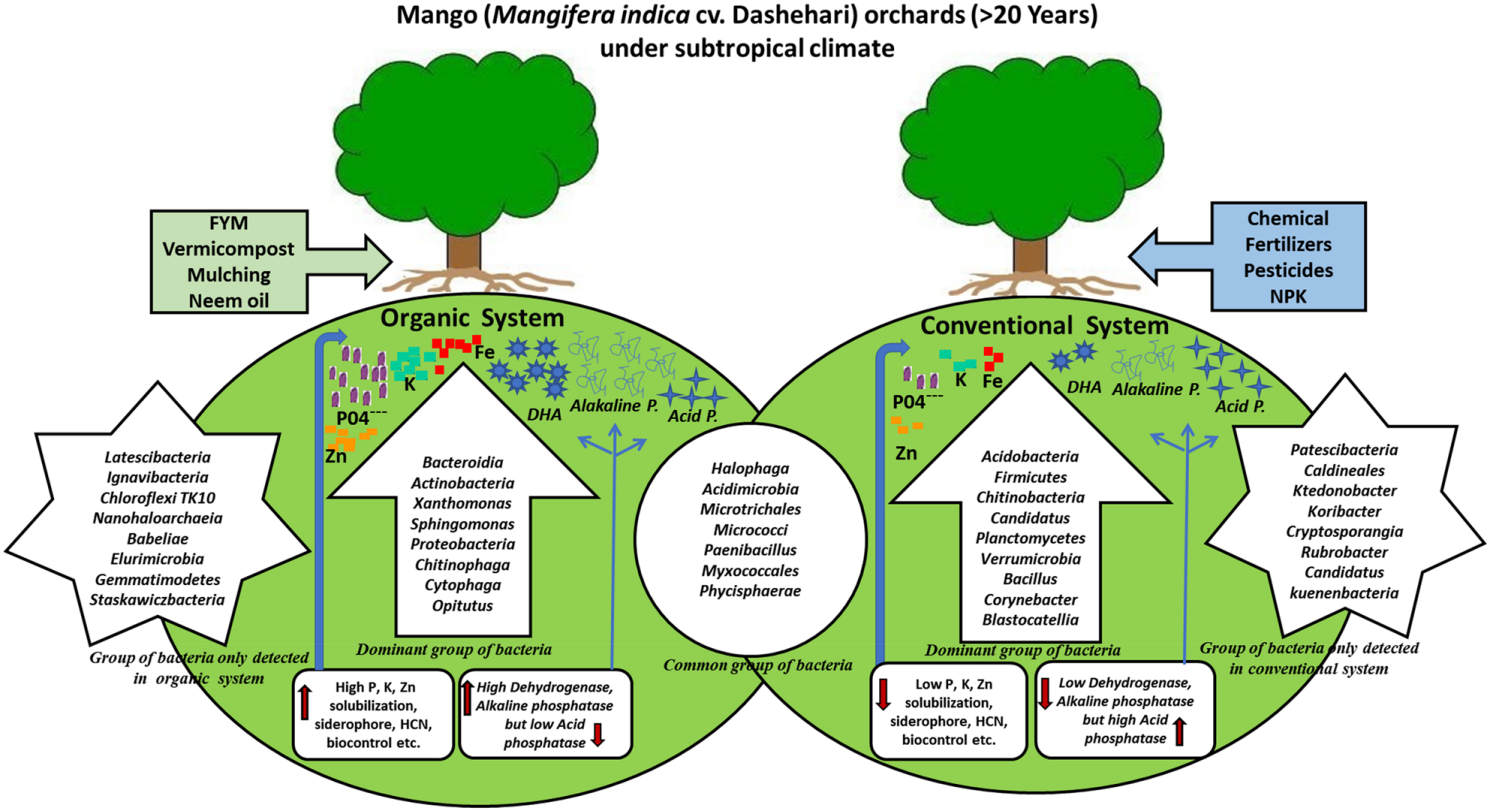
Community assessment of dominant, common and specific microbial groups and their function in organic and conventional management system.

The environmental inference of dominant, standard, and other specific microbial groups can be explained based on V3 and V4 metagenomic sequencing and a culturable approach, and we hypothesized that: (i) long-term organic treatment results in a more diverse plant growth-promoting group of microbial community structure; (ii) conventional fertilization reduces nutrient availability, PGP microbes, and causes a significant change in soil bacterial community structure; and (iii) conventional fertilization causes a significant change in soil bacterial community structure. In conventional treatment, plant–microbe interaction was reduced due to suppressing the utilization of root exudates by rhizosphere microbiome (iv). In organic treatment, rhizosphere microbiome growth was not limited to root exudates secreted by plant roots but can efficiently utilize organic carbon enhanced by FYM and vermicompost treatment. Based on obtained results, it was evident that organic treatment activates more PGP microbes than conventional treatment^11^ (Fig. 9). The schematic diagram (Fig. 9) showed the overall impact and hypothesis of both management systems (organic and conventional) in mango orchards in a subtropical climate. In the organic management system, continuous use of FYM, vermicompost, and mulching boosts organic carbon, enhancing microbial diversity and nutrient availability. *Dehydrogenase* (DHA) and *alkaline phosphatase* activity ultimately benefits the plant via root hairs (rhizosphere). In conventional systems, high chemical fertilizer applications reduce microbial diversity (PGPR), soil enzyme activity (except *acid phosphatase*), and nutrient availability in the plant rhizosphere. Excessive use of chemicals create an acidic environment that favors *Acidobacteria* and *Firmicutes'* growth in higher abundance. That’s why in conventional systems, higher *acid phosphatase* activity was observed. A few common microbial groups such as (*Halophaga, Acidimicrobia, Micrococci, Paenibacillus,* and others) were also found in low numbers but equal proportions in both systems and were sustained despite different treatments. Overall, rhizomicrobiome changed according to the treatments of both systems. For a short period, the conventional system appeared to be the best approach in terms of productivity. Still, the organic system comes with healthy, eco-friendly, and sustainable productivity in the long run.

## Methods

### Experimental setup and soil sample collection

Experimental orchards of *Mangifera indica* L. cv. Dashehari (located at Central Institute for subtropical horticulture, co-ordinates 26° 45′ to 27° 10′ N latitude, 80° 30′ to 80° 55′ E longitude and 123 m above sea level at Lucknow, India), were long term (˃20 years) treated with Organic (Farmyard Manure, Vermicompost and Mulching) and Conventional (chemical fertilizers, NPK and pesticides) treatments. Both treatments organic (G1) and conventional (G2) were set the area of 50 × 50 m planted with 25 Mango trees in three replications. The experimental setup is schematically represented (Supplementary S1). The experimental orchards are situated in the subtropical climate of central plain region and fifth agro-climatic zone of India with an annual rainfall 979 mm and average annual temperature 32.61 °C. The soil type is sandy loam (approximately 40% sand, 40% silt, and 20% clay) developed from alluvial sediments of the Ganga River.

The rhizospheric soil samples were collected from 0–30 cm depth by removing the upper litter layer from both organic and conventional treatment system. From each replication plot five soil samples were collected randomly. Soil was homogenized and passed through a 2 mm mesh and removed plant debris. All the samples were divided into two parts, one portion was used to test the soil enzymatic assay, and another portion was stored at − 20 °C for Metagenomic analysis.

### Culturable approach for the isolation and identification of microorganisms

The rhizosphere soil samples were used to isolate bacteria using serial dilution and spread/pour plate methods. The colony-forming unit (mL^−1^) for bacteria was observed in the Nutrient agar and Rose Bengal agar medium. All the cultures were characterized for morphological features. Two features-Staining (Gram reaction) and cultural characterization were studied as per the Bergeys Manual of Determinative Bacteriology^39^. For molecular characterization, the genomic DNA was extracted by DNA isolation kit (ZR Fungal/Bacterial DNA MiniPrep™). The amplification of gDNA was done by the method of Edwards et al.^40^. Finally, microbial identification was performed based on the percent similarity index (≥ 97%) in the NCBI Gene Bank.

### Culture media, chemicals, and reagent

Various media’s recipe and reagents used in the study including Aleksandrov agar medium **(**Glucose 5.0 g, FeCl_3_ 0.005 g, CaCO_3_ 0.1 g, Ca_3_(PO_4_)_2_ 2.0 g, AlKO_6_Si_2_ 2.0 g, MgSO_4_*.*7H_2_O 0.5 g, Agar 20 g, pH 7.2 and Distilled water 1000 mL), Chrome Azurol’s agar (CAS) medium (Dye solution Chrome azurol’s dye 60.50 mg, Distilled water 50.0 mL, 1 mM FeCl_3_.6H_2_O in 10 mM HCl 10.0 mL, Hexadecyl trimethyl ammonium bromide 72.90 mg, Distilled water 40 mL, pH 6.8 ± 0.2), JNFB medium (di potassium hydrogen phosphate 0.6 g, Potassium di hydrogen phosphate 1.8 g, MgSO_4_.7H_2_O 0.2 g, Malic acid 5.0 g, NaCl 0.1, CaCl_2_.2H_2_O 0.02, KOH 4.5, Fe-EDTA-1.64% -4 mL, Bromothymol blue 2 mL, Vitamin solution 1 mL, Micronutrient solution 2 mL, Agar 1.9 g, pH 5.8, Distilled water 1000 mL), King B agar (MgSO_4_ 0.4 g, K_2_HPO_4_ 1.0, Proteosepeptone 20.0 g, Glycerol 8 mL, Agar 20.0), Nutrient agar (Beef extract 3.0, Peptone 5.0, Agar 20.0), Pikovskaya’s agar (Glucose 10 g, (NH_4_)_2_SO_4_ 0.5 g, MgSO_4_.7H_2_0 0.1 g, Ca_3_(PO_4_) 25.0 g, NaCl 0.2 g, KCl 0.2 g, Rose Bengal agar (MgSO_4_ 0.5 g, Dipotassium phosphate 1.0 g, Rose Bengal 0.05 g, agar 15 g), Yeast extract 0.5 g, FeSO_4_.7H_2_O 0.002 g, MnSO_4_.H2O 0.002 g, agar 20 g, pH 7.0, Salkowasky reagent 1 mL of 0.5 M FeCl_3_ in 50 mL of 35% HClO_4_) and gram staining kit.

### Plant growth promotory properties

Plant growth promotory properties were tested for (a) Phosphate solubilization using NBRIP agar^41^. (b) Zn solubilization was tested according to Saravanan et al.^42^. (c) K solubilization tested according to Hu et al.^43^ on modified Aleksandrov agar medium (c) Siderophore production using Chrome Azurol-S-Agar media^44^. (d) Indole Acetic Acid production using Salkovaskaya reagent^45^. (e) Ammonia production using peptone water. (f) Hydrogen cyanide production using King’s B media amended with glycine^46^. Acetylene reduction assay (ARA) was performed by using an N-free JNfb medium for aerobic and microaerophilic isolates with the help of the Gas Chromatography technique^47^. The active culture (10^5^–10^6^ CFU mL^−1^) was inoculated in 8 mL of JNfb solid and semi-solid medium with sterilized Subba seal. The tubes were incubated for 15 days at 30 ± 2 °C. In each test tube, the acetylene gas (analog of N_2_ gas) was added (10% v/v) and incubated for four h at 30 ± 2 °C. The control treatment (without bacterial inoculation) was maintained.

For measurement, 1.0 mL of acetylene gas was injected by syringe and observed for acetylene reduction by using a gas chromatograph (Shimadzu-2014 model) with detector (FID) and Porapak N column using N_2_ as carrier gas. The conditions for GC operations were for temperature 110, 75, and 110 °C for injector, column, and detector, respectively. The standard ethylene retention period (100 rpm) was used by acetylene reduction assay to calculate the amount of ethylene production. Therefore, the ARA was expressed in terms of PPM of C_2_H_4_ produced slant^−1^ h^−1^.

### Soil *dehydrogenase*, *acid phosphatase*, and *alkaline phosphatase* activity

The soil *dehydrogenase* activity (DHA) was performed by following the protocol described by Casida et al.^48^. The *acid phosphatase* activity was measured according to Tabatabai and Bremner^49^. Finally, to test *alkaline phosphatase* activity, the methods described by Tabatabai and Bremner^49^ and Eivazi and Tabatabai^50^ were followed.

### Unculturable approach (metagenomic) microbiome analysis and amplicon sequencing by using Illumina Hi-Seq 2500

The DNA was isolated from soil samples using DNase Power Soil kit (Qiagen, USA) and 2% CTAB conventional DNA extraction method for the shoot and root samples as per the standard protocol. The DNA concentration was estimated using Qubit Fluorimeter (V.3.0). The V3–V4 region of 16S rRNA was amplified using specific V3 forward primer CCTACGGGNBGCASCAG and V4 reverse primer GACTACNVGGGTATCTAATCC. The amplified product was checked on 2% agarose gel, and gel purification was done to remove non-specific amplifications. By using NEB Next Ultra DNA library preparation kit, 5.0 ng of amplified product was used. Agilent 2200 Tape Station was used for library quantification and quality estimation. This prepared library, with the help of Illumina HiSeq 2500, was sequenced with 2 × 250 cycles chemistry. Sample details, sample summary, raw read summary, raw read summary with Phred quality score distribution, base composition distribution of the samples, trimmed and consensus read the synopsis, etc., are mentioned in Supplementary file S1.1, S1.2, S1.3, S1.4, S1.5, S1.6 and S1.7. The Metagenome sequences for organic G1-SRX8289747, conventional G2-SRX8289748 and, study PRJNA631113 were submitted NCBI-Sequence Read Archive (SRA).

For metagenome sequence, Fastq quality check was subjected to the FastQC program (version.0.11.8) to check the quality of the reads with default parameters. The base quality (Phred Score; Q), base composition, GC content, ambiguous bases (other than A, T, G, C), and adapter dimers were thoroughly checked before the Bioinformatics analysis (Supplementary file S1.1, S1.2, S1.3, S1.4, S1.5, S1.6 and S1.7). The Base quality score distribution observed that total reads (more than 80%) have Phred score more than 30 (> Q30; error-probability >  = 0.001). The sample Phred score distribution is provided in Supplementary file S1.4. In addition, the base composition distribution of the paired-end read sequences from the left and right end was calculated. In samples, the target V3–V4 region sequence composition bias was observed. In Supplementary file S1.5, the base compositions of the samples were provided. The GC content ranges from 30–60%.

### Identification of variable region (V3–V4) and other parameters

To extract the V3–V4 region from Illumina paired-end sequences. (a) Trimming of sequencing primers, the forward V3 specific primer, and reverse V4 specific primers (see “Methods” section) were trimmed using an In-house PERL script. The properly paired-end reads with Phred score quality (Q > 20) were considered for V3–V4 consensus generation. (b) From trimmed paired-end reads building the consensus V3–V4 region. First, the primer trimmed; high-quality paired-end reads were pair-wisely allowed to merge/stitch to get the V3–V4 amplicon consensus FASTA sequences. By using the FLASH program (version 1.2.11), the reads were merged with 10 bp (minimum overlap) to 240 bp (maximum overlap) with Zero mismatches. Thus, the contig length of 350 to 450 bp was formed while making a consensus V3–V4 sequence.

For Pre-processing of reads: Using UCHIME (version 11) de-novo chimera removal method in the tool V SEARCH, the Chimeras were removed. In Supplementary file S1.6, details of chimera filter based on an individual sample are given. For Taxonomy classification, the Operational Taxonomic Units (OTU) picking and taxonomy classification was performed using pre-processed V3–V4 sequences. All samples (pre-processed reads) were grouped into OTUs depend on their similarity in the sequences using the Uclust program (similarity = 0.97) available in QIIME software.

From 335,792 reads, a total of 45,394 OTUs were identified. Further, from 45,394 total OTUs, 38,923 OTUs with less than five reads were deleted, and 6471 OTUs were used for other observations (Supplementary file S1.7).

### Conditions for soil metagenome analysis

For metagenomic analysis, different conditions were followed; (i) Study: Bacterial samples Metagenomic analysis based on 16S rRNA gene (V3–V4 Region), (ii) No of Samples: 2, (iii) Sample Type: Total Mango rhizosphere gDNA, (iv) Library: 2 × 250 Paired-End Library, (v) Proposed platform: IlluminaHiSeq Rapid (vi). Data/Sample: 0.5 Million Reads/sample, (vii) Read Quality: 70% Reads will be Q30. (viii) Requirement: ~ 3–5 µg high-quality genomic DNA (Concentration: ≥ 50 ng/µl) should be for each sample. The DNA should not be degraded and should have an A260/280 ratio of ~ 1.8–2.0.

### Sequencing technology, strategy, details, and protocol used for the metagenomic study

This involves V3–V4 region amplification of the 16S rRNA gene (Bacteria) library preparation, quality checks, and sequencing on Illumina HiSeq (Rapid). It also requires bioinformatics analysis of the sequence data based on the denovo-assembly pipeline, advanced metagenomic analysis, etc. Sequencing technology used: Illumina, SBS; Strategy: amplicon library sequencing of microbiome samples; Amplification and sequencing of hypervariable (V3–V4) of the 16S rRNA gene; Details: paired-end 250 bp sequencing run will be performed on the HiSeq system; Protocol: design and synthesize the primers needed for amplification of V3–V4 region of 16S rRNA gene (Bacteria) and build a library and sequence on HiSeq (Rapid). For other deliverables of amplicon metagenomic sequencing, the α-Diversity analysis: diversity analysis was performed to provide the different diversity indices for diversity and rarefaction plots. Comparative heat map of bacterial diversity after metagenomic (V3 and V4) samples sequencing.

### Genomics, bioinformatics and statistical analysis

The bioinformatics pipeline according to Lozupone et al.^51^; D’Argenio et al.^52^; Caporaso et al.^53^; De-santis et al.^54,55^; Galand et al.^56^ and Arvindaraja et al.^57^ were performed for the variable regions (V3–V4) in terms of (1) Fastq quality checking (Base quality, base composition, GC content), (2) Filtering and identification of V3–V4 region from paired-end data (Read trimming and identification of V3–V4 sequences, Constructing consensus sequence from paired-end reads), (3) Operational Taxonomy Unit (OTUs), Taxonomy Classification and Relative abundance (Identification of OTUs, Assignment of taxonomy to each OUT, Identification of read and OTU abundance) (4) Alpha diversity with samples and rarefaction curves (Shannon, Chao1 and Observed species). For 16SrRNA library preparations and sequencing after DNA isolation the V3 and V4 primer amplification was done by using NEBNext protocol. After that paired-end sequencing (Illuminahiseq 2500), Fastq quality check, adaptor trim, duplicate, chimera removal, and paired-end stitching were done by using FLASH or ClustalO (sample consensus for binning). QIIME pipeline-based taxonomic annotation, picking up the operational taxonomic unit (OTU), OUT filtering (with < 5 reads), and finding representative sequencing, taxonomic classification were observed based on SILVA database. The data was presented as means ± S.D. (standard deviation of means) of five replicates. The significance difference between the means of different treatments, one-way ANOVA with Tukey's HSD test and Spearman's correlation were performed on SPSS software (version 26.0) with significance at P ˂0.05.

## Supplementary Information

Supplementary Information 1.

Supplementary Information 2.

Supplementary Information 3.

Supplementary Information 4.
