# Supplementary material for: Bacterial structure and dynamics in mango (Mangifera indica) orchards after long term organic and conventional treatments under subtropical ecosystem
Source: Sci Rep. 2021 Oct 15;11:20554. doi: 10.1038/s41598-021-00112-0 (PMC8519990; doi:10.1038/s41598-021-00112-0)
Supplement: Supplementary file 1 — Supplementary Information 1. [file 41598_2021_112_MOESM1_ESM.pdf]

Taxonomy Summary. Current Level:

		Total	G1	G2
Legend	Taxonomy	%	%	%
	Unclassified;Other	0.5%	0.7%	0.3%
	p Acidobacteria;Other	0.1%	0.2%	0.1%
	p Acidobacteria;c Acidobacteriia	2.3%	1.7%	2.8%
	p Acidobacteria;c Blastocatellia-(Subgroup-4)	9.6%	5.6%	13.6%
	p Acidobacteria;c Holophagae	0.3%	0.4%	0.3%
	p Acidobacteria;c Subgroup-11	0.1%	0.1%	0.1%
	p Acidobacteria;c Subgroup-15	0.0%	0.0%	0.0%
	p Acidobacteria;c Subgroup-17	0.2%	0.2%	0.1%
	p Acidobacteria;c Subgroup-18	0.0%	0.0%	0.0%
	p Acidobacteria;c Subgroup-20	0.0%	0.0%	0.0%
	p Acidobacteria;c Subgroup-22	0.3%	0.4%	0.2%
	p Acidobacteria;c Subgroup-25	0.1%	0.1%	0.1%
	p Acidobacteria;c Subgroup-5	0.4%	0.4%	0.4%
	p Acidobacteria;c Subgroup-6	6.6%	7.4%	5.7%
	p Acidobacteria;c Subgroup-9	0.0%	0.0%	0.0%
	p Actinobacteria;Other	0.0%	0.0%	0.1%
	p Actinobacteria;c 0319-7L14	0.1%	0.1%	0.1%
	p Actinobacteria;c Acidimicrobiia	2.8%	3.3%	2.2%
	p Actinobacteria;c Actinobacteria	13.4%	14.1%	12.7%
	p Actinobacteria;c MB-A2-108	0.7%	0.8%	0.5%
	p Actinobacteria;c Nitriliruptoria	0.0%	0.0%	0.0%
	p Actinobacteria;c Rubrobacteria	0.1%	0.0%	0.1%
	p Actinobacteria;c Thermoleophilia	1.2%	1.3%	1.1%
	p Armatimonadetes;c Fimbriimonadia	0.0%	0.0%	0.0%
	p BRC1;Other	0.0%	0.0%	0.0%
	p BRC1;c uncultured-bacterium	0.0%	0.0%	0.0%
	p Bacteroidetes;c Bacteroidia	11.8%	14.6%	9.0%
	p Bacteroidetes;c Ignavibacteria	0.2%	0.2%	0.1%
	p Chloroflexi;Other	0.0%	0.0%	0.0%
	p Chloroflexi;c AD3	0.0%	0.0%	0.0%
	p Chloroflexi;c Anaerolineae	2.0%	1.6%	2.4%
	p Chloroflexi;c Chloroflexia	1.7%	0.8%	2.6%
	p Chloroflexi;c Dehalococcoidia	0.4%	0.5%	0.3%
	p Chloroflexi;c Gitt-GS-136	0.3%	0.4%	0.1%
	p Chloroflexi;c JG30-KF-CM66	0.1%	0.2%	0.1%
	p Chloroflexi;c KD4-96	0.7%	0.9%	0.5%
	p Chloroflexi;c Ktedonobacteria	0.1%	0.0%	0.1%
	p Chloroflexi;c OLB14	0.1%	0.1%	0.1%
	p Chloroflexi;c P2-11E	0.2%	0.1%	0.2%
	p Chloroflexi;c TK10	0.3%	0.3%	0.3%
	p Dadabacteria;c Dadabacteriia	0.0%	0.0%	0.0%
	p Dependentiae;c Babeliae	0.1%	0.1%	0.0%
	p Elusimicrobia;c Elusimicrobia	0.0%	0.0%	0.0%
	p Elusimicrobia;c Lineage-IIa	0.1%	0.1%	0.0%
	p Elusimicrobia;c Lineage-IIb	0.0%	0.0%	0.0%
	p Entotheonellaeota;c Entotheonellia	0.1%	0.1%	0.1%
	p Euryarchaeota;c Methanomicrobia	0.0%	0.0%	0.0%
	p Euryarchaeota;c Thermoplasmata	1.8%	0.9%	2.8%
	p Firmicutes;c Bacilli	2.9%	0.9%	4.8%
	p Firmicutes;c Clostridia	0.0%	0.0%	0.0%
	p GAL15;c uncultured-bacterium	0.0%	0.0%	0.0%
	p Gemmatimonadetes;c AKAU4049	0.2%	0.3%	0.0%
	p Gemmatimonadetes;c BD2-11-terrestrial-group	0.1%	0.2%	0.0%
	p Gemmatimonadetes;c Gemmatimonadetes	2.6%	2.5%	2.8%
	p Gemmatimonadetes;c Longimicrobia	0.0%	0.0%	0.0%
	p Gemmatimonadetes;c S0134-terrestrial-group	0.5%	0.5%	0.5%
	p Hydrogenedentes;c Hydrogenedentia	0.0%	0.0%	0.0%
	p Latescibacteria;Other	0.3%	0.4%	0.1%
	p Latescibacteria;c Latescibacteria	0.2%	0.3%	0.0%
	p Latescibacteria;c uncultured-Acidobacterium-sp.	0.0%	0.0%	0.0%
	p Latescibacteria;c uncultured-Fibrobacteres-bacterium	0.0%	0.0%	0.0%
	p Latescibacteria;c uncultured-Pelobacter-sp.	0.0%	0.0%	0.0%
	p Latescibacteria;c uncultured-bacterium	0.4%	0.5%	0.3%
	p Latescibacteria;c uncultured-prokaryote	0.0%	0.0%	0.0%
	p Latescibacteria;c uncultured-proteobacterium	0.0%	0.0%	0.0%

	p Latescibacteria;c uncultured-soil-bacterium	0.0%	0.1%	0.0%
	p Nanoarchaeaeota;c Nanohaloarchaeia	0.1%	0.1%	0.0%
	p Nanoarchaeaeota;c Woesearchaeia	0.2%	0.4%	0.1%
	p Nitrospirae;c Nitrospira	0.5%	0.4%	0.7%
	p Patescibacteria;Other	0.0%	0.0%	0.0%
	p Patescibacteria;c ABY1	0.1%	0.0%	0.1%
	p Patescibacteria;c Berkelbacteria	0.0%	0.0%	0.0%
	p Patescibacteria;c Gracilibacteria	0.0%	0.0%	0.0%
	p Patescibacteria;c Microgenomatia	0.0%	0.0%	0.0%
	p Patescibacteria;c Parcubacteria	0.1%	0.1%	0.2%
	p Patescibacteria;c Saccharimonadia	0.5%	0.4%	0.6%
	p Patescibacteria;c WWE3	0.0%	0.0%	0.0%
	p Planctomycetes;Other	0.0%	0.0%	0.0%
	p Planctomycetes;c BD7-11	0.0%	0.0%	0.0%
	p Planctomycetes;c OM190	0.0%	0.0%	0.0%
	p Planctomycetes;c Phycisphaerae	1.7%	1.4%	2.1%
	p Planctomycetes;c Pla3-lineage	0.0%	0.0%	0.0%
	p Planctomycetes;c Pla4-lineage	0.1%	0.1%	0.1%
	p Planctomycetes;c Planctomycetacia	0.4%	0.4%	0.5%
	p Proteobacteria;Other	0.0%	0.0%	0.0%
	p Proteobacteria;c Alphaproteobacteria	10.3%	11.1%	9.5%
	p Proteobacteria;c Deltaproteobacteria	2.7%	2.8%	2.6%
	p Proteobacteria;c Gammaproteobacteria	8.7%	10.8%	6.6%
	p Rokubacteria;c NC10	1.2%	1.6%	0.9%
	p Thaumarchaeota;c Group-1.1c	0.0%	0.0%	0.0%
	p Thaumarchaeota;c Nitrososphaeria	1.3%	1.3%	1.3%
	p Verrucomicrobia;c Verrucomicrobiae	6.1%	5.5%	6.8%
	p WPS-2;Other	0.0%	0.0%	0.0%
	p Zixibacteria;Other	0.0%	0.0%	0.0%
	p Zixibacteria;c uncultured-bacterium	0.0%	0.0%	0.0%

Taxonomy Summary. Current Level:

		Total	G1	G2
Legend	Taxonomy	%	%	%
	Unclassified;Other;Other	0.5%	0.7%	0.3%
	p Acidobacteria;Other;Other	0.1%	0.2%	0.1%
	p Acidobacteria;c Acidobacteriia;o Acidobacteriales	0.3%	0.2%	0.4%
	p Acidobacteria;c Acidobacteriia;o Solibacterales	1.8%	1.4%	2.3%
	p Acidobacteria;c Acidobacteriia;o Subgroup-12	0.0%	0.0%	0.0%
	p Acidobacteria;c Acidobacteriia;o Subgroup-13	0.0%	0.0%	0.0%
	p Acidobacteria;c Acidobacteriia;o Subgroup-2	0.1%	0.2%	0.1%
	p Acidobacteria;c Blastocatellia-(Subgroup-4);Other	2.6%	1.6%	3.6%
	p Acidobacteria;c Blastocatellia-(Subgroup-4);o 11-24	0.4%	0.4%	0.4%
	p Acidobacteria;c Blastocatellia-(Subgroup-4);o Blastocatellales	0.7%	0.5%	0.9%
	p Acidobacteria;c Blastocatellia-(Subgroup-4);o DS-100	0.0%	0.0%	0.0%
	p Acidobacteria;c Blastocatellia-(Subgroup-4);o Elev-16S-573	5.9%	3.1%	8.7%
	p Acidobacteria;c Holophagae;o Holophagales	0.0%	0.0%	0.0%
	p Acidobacteria;c Holophagae;o Subgroup-7	0.3%	0.4%	0.3%
	p Acidobacteria;c Subgroup-11;Other	0.1%	0.1%	0.1%
	p Acidobacteria;c Subgroup-11;o uncultured-bacterium	0.0%	0.0%	0.0%
	p Acidobacteria;c Subgroup-15;Other	0.0%	0.0%	0.0%
	p Acidobacteria;c Subgroup-15;o uncultured-bacterium	0.0%	0.0%	0.0%
	p Acidobacteria;c Subgroup-17;Other	0.1%	0.1%	0.0%
	p Acidobacteria;c Subgroup-17;o uncultured-Acidobacteria-bacterium	0.0%	0.0%	0.0%
	p Acidobacteria;c Subgroup-17;o uncultured-Acidobacteriales-bacterium	0.0%	0.0%	0.0%
	p Acidobacteria;c Subgroup-17;o uncultured-bacterium	0.1%	0.1%	0.0%
	p Acidobacteria;c Subgroup-18;Other	0.0%	0.0%	0.0%
	p Acidobacteria;c Subgroup-18;o uncultured-bacterium	0.0%	0.0%	0.0%
	p Acidobacteria;c Subgroup-20;o uncultured-bacterium	0.0%	0.0%	0.0%
	p Acidobacteria;c Subgroup-22;Other	0.1%	0.1%	0.1%
	p Acidobacteria;c Subgroup-22;o uncultured-Acidobacterium-sp.	0.0%	0.1%	0.0%
	p Acidobacteria;c Subgroup-22;o uncultured-bacterium	0.1%	0.2%	0.1%
	p Acidobacteria;c Subgroup-25;Other	0.0%	0.1%	0.0%
	p Acidobacteria;c Subgroup-25;o uncultured-Acidobacteria-bacterium	0.0%	0.0%	0.0%
	p Acidobacteria;c Subgroup-25;o uncultured-bacterium	0.0%	0.0%	0.0%
	p Acidobacteria;c Subgroup-25;o uncultured-soil-bacterium	0.0%	0.0%	0.0%
	p Acidobacteria;c Subgroup-5;Other	0.0%	0.0%	0.0%
	p Acidobacteria;c Subgroup-5;o uncultured-Acidobacteria-bacterium	0.2%	0.2%	0.2%

	p Acidobacteria;c Subgroup-5;o uncultured-Acidobacterium-sp.	0.0%	0.0%	0.0%
	p Acidobacteria;c Subgroup-5;o uncultured-bacterium	0.2%	0.2%	0.2%
	p Acidobacteria;c Subgroup-6;Other	5.4%	6.2%	4.6%
	p Acidobacteria;c Subgroup-6;o Acidobacteria-bacterium-WX90	0.0%	0.0%	0.0%
	p Acidobacteria;c Subgroup-6;o Unknown-Order	0.0%	0.0%	0.0%
	p Acidobacteria;c Subgroup-6;o uncultured-Acidobacteria-bacterium	0.2%	0.2%	0.2%
	p Acidobacteria;c Subgroup-6;o uncultured-Acidobacteriaceae-bacterium	0.0%	0.0%	0.0%
	p Acidobacteria;c Subgroup-6;o uncultured-Acidobacteriales-bacterium	0.1%	0.1%	0.1%
	p Acidobacteria;c Subgroup-6;o uncultured-Acidobacterium-sp.	0.0%	0.0%	0.0%
	p Acidobacteria;c Subgroup-6;o uncultured-Holophagae-bacterium	0.0%	0.0%	0.0%
	p Acidobacteria;c Subgroup-6;o uncultured-bacterium	0.8%	0.9%	0.8%
	p Acidobacteria;c Subgroup-6;o uncultured-beta-proteobacterium	0.0%	0.0%	0.0%
	p Acidobacteria;c Subgroup-6;o uncultured-proteobacterium	0.0%	0.0%	0.0%
	p Acidobacteria;c Subgroup-9;Other	0.0%	0.0%	0.0%
	p Actinobacteria;Other;Other	0.0%	0.0%	0.1%
	p Actinobacteria;c 0319-7L14;Other	0.0%	0.0%	0.1%
	p Actinobacteria;c 0319-7L14;o uncultured-bacterium	0.0%	0.0%	0.0%
	p Actinobacteria;c Acidimicrobiia;Other	0.3%	0.3%	0.3%
	p Actinobacteria;c Acidimicrobiia;o Acidimicrobiales	0.0%	0.0%	0.0%
	p Actinobacteria;c Acidimicrobiia;o Actinomarinales	0.7%	1.3%	0.1%
	p Actinobacteria;c Acidimicrobiia;o IMCC26256	0.5%	0.5%	0.4%
	p Actinobacteria;c Acidimicrobiia;o Microtrichales	0.8%	0.8%	0.7%
	p Actinobacteria;c Acidimicrobiia;o uncultured	0.5%	0.3%	0.8%
	p Actinobacteria;c Actinobacteria;Other	0.0%	0.0%	0.0%
	p Actinobacteria;c Actinobacteria;o Corynebacteriales	1.4%	0.7%	2.2%
	p Actinobacteria;c Actinobacteria;o Frankiales	0.2%	0.1%	0.2%
	p Actinobacteria;c Actinobacteria;o Glycomycetales	1.0%	1.9%	0.1%
	p Actinobacteria;c Actinobacteria;o Kineosporiales	0.0%	0.0%	0.0%
	p Actinobacteria;c Actinobacteria;o Micrococcales	0.6%	0.8%	0.5%
	p Actinobacteria;c Actinobacteria;o Micromonosporales	0.5%	0.5%	0.5%
	p Actinobacteria;c Actinobacteria;o Propionibacteriales	1.6%	1.6%	1.5%
	p Actinobacteria;c Actinobacteria;o Pseudonocardiales	0.3%	0.2%	0.4%
	p Actinobacteria;c Actinobacteria;o Streptomycetales	7.6%	8.0%	7.3%
	p Actinobacteria;c Actinobacteria;o Streptosporangiales	0.1%	0.2%	0.0%
	p Actinobacteria;c MB-A2-108;Other	0.1%	0.1%	0.1%
	p Actinobacteria;c MB-A2-108;o uncultured-actinobacterium	0.0%	0.0%	0.0%
	p Actinobacteria;c MB-A2-108;o uncultured-bacterium	0.6%	0.7%	0.4%
	p Actinobacteria;c MB-A2-108;o uncultured-bacterium-contig00016	0.0%	0.0%	0.0%
	p Actinobacteria;c Nitriliruptoria;o Euzebyales	0.0%	0.0%	0.0%
	p Actinobacteria;c Rubrobacteria;o Rubrobacterales	0.1%	0.0%	0.1%
	p Actinobacteria;c Thermoleophilia;Other	0.1%	0.1%	0.1%
	p Actinobacteria;c Thermoleophilia;o Gaiellales	0.6%	0.7%	0.6%
	p Actinobacteria;c Thermoleophilia;o Solirubrobacterales	0.5%	0.5%	0.4%
	p Actinobacteria;c Thermoleophilia;o uncultured	0.0%	0.0%	0.0%
	p Armatimonadetes;c Fimbriimonadia;o Fimbriimonadales	0.0%	0.0%	0.0%
	p BRC1;Other;Other	0.0%	0.0%	0.0%
	p BRC1;c uncultured-bacterium;o uncultured-bacterium	0.0%	0.0%	0.0%
	p Bacteroidetes;c Bacteroidia;Other	0.0%	0.0%	0.0%
	p Bacteroidetes;c Bacteroidia;o Bacteroidetes-VC2.1-Bac22	0.0%	0.0%	0.0%
	p Bacteroidetes;c Bacteroidia;o Chitinophagales	8.4%	11.3%	5.4%
	p Bacteroidetes;c Bacteroidia;o Cytophagales	2.1%	2.1%	2.2%
	p Bacteroidetes;c Bacteroidia;o Flavobacteriales	0.4%	0.2%	0.5%
	p Bacteroidetes;c Bacteroidia;o Sphingobacteriales	0.9%	1.0%	0.9%
	p Bacteroidetes;c Ignavibacteria;o Ignavibacteriales	0.0%	0.0%	0.0%
	p Bacteroidetes;c Ignavibacteria;o Kryptoniales	0.1%	0.1%	0.0%
	p Bacteroidetes;c Ignavibacteria;o OPB56	0.1%	0.0%	0.1%
	p Bacteroidetes;c Ignavibacteria;o SJA-28	0.0%	0.1%	0.0%
	p Chloroflexi;Other;Other	0.0%	0.0%	0.0%
	p Chloroflexi;c AD3;o uncultured-bacterium	0.0%	0.0%	0.0%
	p Chloroflexi;c Anaerolineae;Other	0.0%	0.0%	0.0%
	p Chloroflexi;c Anaerolineae;o Anaerolineales	1.2%	0.9%	1.5%
	p Chloroflexi;c Anaerolineae;o Ardenticatenales	0.2%	0.1%	0.2%
	p Chloroflexi;c Anaerolineae;o Caldilineales	0.0%	0.0%	0.1%
	p Chloroflexi;c Anaerolineae;o RBG-13-54-9	0.1%	0.1%	0.1%
	p Chloroflexi;c Anaerolineae;o SBR1031	0.4%	0.4%	0.5%
	p Chloroflexi;c Anaerolineae;o uncultured-Bellilinea-sp.	0.0%	0.0%	0.0%
	p Chloroflexi;c Chloroflexia;o Chloroflexales	1.2%	0.4%	2.0%
	p Chloroflexi;c Chloroflexia;o Kallotenuales	0.0%	0.0%	0.0%
	p Chloroflexi;c Chloroflexia;o Thermomicrobiales	0.5%	0.4%	0.6%

	p Chloroflexi;c Dehalococcoidia;o S085	0.4%	0.5%	0.3%
	p Chloroflexi;c Gitt-GS-136;Other	0.0%	0.0%	0.0%
	p Chloroflexi;c Gitt-GS-136;o uncultured-bacterium	0.3%	0.4%	0.1%
	p Chloroflexi;c JG30-KF-CM66;Other	0.0%	0.0%	0.0%
	p Chloroflexi;c JG30-KF-CM66;o uncultured-Chloroflexi-bacterium	0.0%	0.0%	0.0%
	p Chloroflexi;c JG30-KF-CM66;o uncultured-bacterium	0.1%	0.1%	0.1%
	p Chloroflexi;c KD4-96;Other	0.2%	0.3%	0.1%
	p Chloroflexi;c KD4-96;o uncultured-Chloroflexi-bacterium	0.1%	0.1%	0.1%
	p Chloroflexi;c KD4-96;o uncultured-bacterium	0.4%	0.5%	0.3%
	p Chloroflexi;c Ktedonobacteria;o C0119	0.0%	0.0%	0.0%
	p Chloroflexi;c Ktedonobacteria;o Ktedonobacterales	0.0%	0.0%	0.1%
	p Chloroflexi;c OLB14;Other	0.0%	0.0%	0.0%
	p Chloroflexi;c OLB14;o uncultured-bacterium	0.1%	0.1%	0.1%
	p Chloroflexi;c OLB14;o uncultured-gamma-proteobacterium	0.0%	0.0%	0.0%
	p Chloroflexi;c P2-11E;o uncultured-bacterium	0.2%	0.1%	0.2%
	p Chloroflexi;c TK10;Other	0.2%	0.2%	0.2%
	p Chloroflexi;c TK10;o uncultured-Chloroflexi-bacterium	0.0%	0.0%	0.1%
	p Chloroflexi;c TK10;o uncultured-bacterium	0.0%	0.1%	0.0%
	p Dadabacteria;c Dadabacteriia;o Dadabacteriales	0.0%	0.0%	0.0%
	p Dependentiae;c Babeliae;o Babeliales	0.1%	0.1%	0.0%
	p Elusimicrobia;c Elusimicrobia;o MVP-88	0.0%	0.0%	0.0%
	p Elusimicrobia;c Lineage-IIa;Other	0.0%	0.0%	0.0%
	p Elusimicrobia;c Lineage-IIa;o uncultured-bacterium	0.0%	0.1%	0.0%
	p Elusimicrobia;c Lineage-IIb;o uncultured-bacterium	0.0%	0.0%	0.0%
	p Elusimicrobia;c Lineage-IIb;o uncultured-soil-bacterium	0.0%	0.0%	0.0%
	p Entotheonellaeota;c Entotheonellia;o Entotheonellales	0.1%	0.1%	0.1%
	p Euryarchaeota;c Methanomicrobia;o Methanosarcinales	0.0%	0.0%	0.0%
	p Euryarchaeota;c Thermoplasmata;Other	0.1%	0.1%	0.1%
	p Euryarchaeota;c Thermoplasmata;o Marine-Group-II	1.3%	0.7%	1.8%
	p Euryarchaeota;c Thermoplasmata;o Methanomassiliicoccales	0.5%	0.0%	0.9%
	p Euryarchaeota;c Thermoplasmata;o uncultured	0.0%	0.0%	0.0%
	p Firmicutes;c Bacilli;o Bacillales	2.9%	0.9%	4.8%
	p Firmicutes;c Clostridia;o Clostridiales	0.0%	0.0%	0.0%
	p GAL15;c uncultured-bacterium;o uncultured-bacterium	0.0%	0.0%	0.0%
	p Gemmatimonadetes;c AKAU4049;Other	0.2%	0.3%	0.0%
	p Gemmatimonadetes;c BD2-11-terrestrial-group;Other	0.0%	0.0%	0.0%
	p Gemmatimonadetes;c BD2-11-terrestrial-group;o uncultured-Gemmatimonadales-bacterium	0.0%	0.0%	0.0%
	p Gemmatimonadetes;c BD2-11-terrestrial-group;o uncultured-Gemmatimonadetes-bacterium	0.0%	0.1%	0.0%
	p Gemmatimonadetes;c BD2-11-terrestrial-group;o uncultured-bacterium	0.0%	0.0%	0.0%
	p Gemmatimonadetes;c BD2-11-terrestrial-group;o uncultured-soil-bacterium	0.0%	0.0%	0.0%
	p Gemmatimonadetes;c Gemmatimonadetes;o Gemmatimonadales	2.6%	2.5%	2.8%
	p Gemmatimonadetes;c Longimicrobia;o Longimicrobiales	0.0%	0.0%	0.0%
	p Gemmatimonadetes;c S0134-terrestrial-group;Other	0.1%	0.1%	0.1%
	p Gemmatimonadetes;c S0134-terrestrial-group;o uncultured-Gemmatimonadales-bacterium	0.2%	0.2%	0.2%
	p Gemmatimonadetes;c S0134-terrestrial-group;o uncultured-Gemmatimonadetes-bacterium	0.0%	0.0%	0.0%
	p Gemmatimonadetes;c S0134-terrestrial-group;o uncultured-bacterium	0.2%	0.2%	0.2%
	p Hydrogenedentes;c Hydrogenedentia;o Hydrogenedentiales	0.0%	0.0%	0.0%
	p Latescibacteria;Other;Other	0.3%	0.4%	0.1%
	p Latescibacteria;c Latescibacteria;o Latescibacterales	0.2%	0.3%	0.0%
	p Latescibacteria;c uncultured-Acidobacterium-sp.;o uncultured-Acidobacterium-sp.	0.0%	0.0%	0.0%
	p Latescibacteria;c uncultured-Fibrobacteres-bacterium;o uncultured-Fibrobacteres-bacterium	0.0%	0.0%	0.0%
	p Latescibacteria;c uncultured-Pelobacter-sp.;o uncultured-Pelobacter-sp.	0.0%	0.0%	0.0%
	p Latescibacteria;c uncultured-bacterium;o uncultured-bacterium	0.4%	0.5%	0.3%
	p Latescibacteria;c uncultured-prokaryote;o uncultured-prokaryote	0.0%	0.0%	0.0%
	p Latescibacteria;c uncultured-proteobacterium;o uncultured-proteobacterium	0.0%	0.0%	0.0%
	p Latescibacteria;c uncultured-soil-bacterium;o uncultured-soil-bacterium	0.0%	0.1%	0.0%
	p Nanoarchaeaeota;c Nanohaloarchaeia;o Aenigmarchaeales	0.1%	0.1%	0.0%
	p Nanoarchaeaeota;c Nanohaloarchaeia;o Deep-Sea-Euryarchaeotic-Group(DSEG)	0.0%	0.0%	0.0%
	p Nanoarchaeaeota;c Woesearchaeia;Other	0.1%	0.2%	0.1%
	p Nanoarchaeaeota;c Woesearchaeia;o Candidatus-Amesbacteria-bacterium-GW2011_GWC1_47_15	0.0%	0.0%	0.0%
	p Nanoarchaeaeota;c Woesearchaeia;o Candidatus-Pacearchaeota-archaeon-RBG_19FT_COMBO_34_9	0.0%	0.0%	0.0%
	p Nanoarchaeaeota;c Woesearchaeia;o Candidatus-Staskawiczbacteria-bacterium-RIFOXYA2_FULL_32_7	0.1%	0.1%	0.0%
	p Nanoarchaeaeota;c Woesearchaeia;o uncultured-euryarchaeote	0.0%	0.0%	0.0%
	p Nitrospirae;c Nitrospira;o Nitrospirales	0.5%	0.4%	0.7%
	p Patescibacteria;Other;Other	0.0%	0.0%	0.0%
	p Patescibacteria;c ABY1;Other	0.0%	0.0%	0.0%
	p Patescibacteria;c ABY1;o Candidatus-Kuenenbacteria	0.0%	0.0%	0.1%
	p Patescibacteria;c ABY1;o Candidatus-Magasanikbacteria	0.0%	0.0%	0.0%
	p Patescibacteria;c ABY1;o Candidatus-Uhrbacteria	0.0%	0.0%	0.0%

p Patescibacteria;c Berkelbacteria;o uncultured-bacterium	0.0%	0.0%	0.0%
p Patescibacteria;c Gracilibacteria;o Candidatus-Abawacabacteria	0.0%	0.0%	0.0%
p Patescibacteria;c Gracilibacteria;o Candidatus-Peribacteria	0.0%	0.0%	0.0%
p Patescibacteria;c Microgenomatia;o Candidatus-Woesebacteria	0.0%	0.0%	0.0%
p Patescibacteria;c Parcubacteria;Other	0.0%	0.0%	0.0%
p Patescibacteria;c Parcubacteria;o Candidatus-Azambacteria	0.0%	0.0%	0.0%
p Patescibacteria;c Parcubacteria;o Candidatus-Kaiserbacteria	0.0%	0.0%	0.0%
p Patescibacteria;c Parcubacteria;o Candidatus-Nomurabacteria	0.0%	0.0%	0.0%
p Patescibacteria;c Parcubacteria;o Candidatus-Terrybacteria	0.0%	0.0%	0.0%
p Patescibacteria;c Parcubacteria;o Candidatus-Yanofskybacteria	0.0%	0.0%	0.0%
p Patescibacteria;c Parcubacteria;o GWA2-38-13b	0.0%	0.0%	0.0%
p Patescibacteria;c Parcubacteria;o uncultured-bacterium	0.0%	0.0%	0.0%
p Patescibacteria;c Saccharimonadia;o Saccharimonadales	0.5%	0.4%	0.6%
p Patescibacteria;c WWE3;Other	0.0%	0.0%	0.0%
p Planctomycetes;Other;Other	0.0%	0.0%	0.0%
p Planctomycetes;c BD7-11;o uncultured-Planctomycetales-bacterium	0.0%	0.0%	0.0%
p Planctomycetes;c BD7-11;o uncultured-bacterium	0.0%	0.0%	0.0%
p Planctomycetes;c OM190;Other	0.0%	0.0%	0.0%
p Planctomycetes;c OM190;o uncultured-bacterium	0.0%	0.0%	0.0%
p Planctomycetes;c OM190;o uncultured-soil-bacterium	0.0%	0.0%	0.0%
p Planctomycetes;c Phycisphaerae;Other	0.2%	0.2%	0.2%
p Planctomycetes;c Phycisphaerae;o CCM11a	0.2%	0.2%	0.2%
p Planctomycetes;c Phycisphaerae;o Phycisphaerales	0.1%	0.1%	0.1%
p Planctomycetes;c Phycisphaerae;o Pla1-lineage	0.0%	0.1%	0.0%
p Planctomycetes;c Phycisphaerae;o Tepidisphaerales	1.1%	0.8%	1.4%
p Planctomycetes;c Phycisphaerae;o mle1-8	0.1%	0.0%	0.1%
p Planctomycetes;c Pla3-lineage;Other	0.0%	0.0%	0.0%
p Planctomycetes;c Pla4-lineage;Other	0.0%	0.0%	0.0%
p Planctomycetes;c Pla4-lineage;o uncultured-bacterium	0.0%	0.0%	0.0%
p Planctomycetes;c Pla4-lineage;o uncultured-prokaryote	0.0%	0.0%	0.0%
p Planctomycetes;c Planctomycetacia;o Gemmatales	0.2%	0.1%	0.2%
p Planctomycetes;c Planctomycetacia;o Pirellulales	0.2%	0.2%	0.2%
p Planctomycetes;c Planctomycetacia;o Planctomycetales	0.1%	0.1%	0.1%
p Planctomycetes;c Planctomycetacia;o uncultured	0.0%	0.0%	0.0%
p Proteobacteria;Other;Other	0.0%	0.0%	0.0%
p Proteobacteria;c Alphaproteobacteria;Other	1.8%	1.7%	2.0%
p Proteobacteria;c Alphaproteobacteria;o Azospirillales	0.1%	0.1%	0.0%
p Proteobacteria;c Alphaproteobacteria;o Caulobacterales	0.3%	0.4%	0.2%
p Proteobacteria;c Alphaproteobacteria;o Elsterales	0.1%	0.1%	0.1%
p Proteobacteria;c Alphaproteobacteria;o Holosporales	0.0%	0.0%	0.0%
p Proteobacteria;c Alphaproteobacteria;o Micropepsales	0.1%	0.0%	0.1%
p Proteobacteria;c Alphaproteobacteria;o Reyranellales	0.2%	0.3%	0.2%
p Proteobacteria;c Alphaproteobacteria;o Rhizobiales	5.3%	5.8%	4.9%
p Proteobacteria;c Alphaproteobacteria;o Rhodobacterales	0.0%	0.0%	0.0%
p Proteobacteria;c Alphaproteobacteria;o Rhodospirillales	0.0%	0.0%	0.1%
p Proteobacteria;c Alphaproteobacteria;o Rhodovibrionales	0.0%	0.0%	0.0%
p Proteobacteria;c Alphaproteobacteria;o Sneathiellales	0.0%	0.0%	0.0%
p Proteobacteria;c Alphaproteobacteria;o Sphingomonadales	1.9%	2.3%	1.6%
p Proteobacteria;c Alphaproteobacteria;o Tistrellales	0.0%	0.0%	0.0%
p Proteobacteria;c Alphaproteobacteria;o uncultured	0.4%	0.4%	0.4%
p Proteobacteria;c Deltaproteobacteria;Other	0.2%	0.3%	0.2%
p Proteobacteria;c Deltaproteobacteria;o Bdellovibrionales	0.0%	0.1%	0.0%
p Proteobacteria;c Deltaproteobacteria;o Desulfarculales	0.2%	0.1%	0.2%
p Proteobacteria;c Deltaproteobacteria;o Desulfuromonadales	0.0%	0.0%	0.1%
p Proteobacteria;c Deltaproteobacteria;o MBNT15	0.1%	0.1%	0.2%
p Proteobacteria;c Deltaproteobacteria;o Myxococcales	1.5%	1.7%	1.4%
p Proteobacteria;c Deltaproteobacteria;o NB1-j	0.4%	0.5%	0.3%
p Proteobacteria;c Deltaproteobacteria;o Oligoflexales	0.0%	0.0%	0.0%
p Proteobacteria;c Deltaproteobacteria;o RCP2-54	0.0%	0.0%	0.0%
p Proteobacteria;c Deltaproteobacteria;o SAR324-clade(Marine-group-B)	0.0%	0.0%	0.0%
p Proteobacteria;c Deltaproteobacteria;o Syntrophobacterales	0.1%	0.0%	0.1%
p Proteobacteria;c Gammaproteobacteria;Other	0.5%	0.6%	0.4%
p Proteobacteria;c Gammaproteobacteria;o Acidiferrobacterales	0.0%	0.0%	0.0%
p Proteobacteria;c Gammaproteobacteria;o Aeromonadales	0.0%	0.0%	0.0%
p Proteobacteria;c Gammaproteobacteria;o Betaproteobacteriales	4.5%	4.3%	4.8%
p Proteobacteria;c Gammaproteobacteria;o CCD24	0.2%	0.3%	0.1%
p Proteobacteria;c Gammaproteobacteria;o Cellvibrionales	0.0%	0.0%	0.0%
p Proteobacteria;c Gammaproteobacteria;o Diplorickettsiales	0.0%	0.0%	0.0%
p Proteobacteria;c Gammaproteobacteria;o EPR3968-O8a-Bc78	0.0%	0.0%	0.0%

	p Proteobacteria;c Gammaproteobacteria;o Enterobacteriales	0.0%	0.0%	0.0%
	p Proteobacteria;c Gammaproteobacteria;o Gammaproteobacteria-Incertae-Sedis	0.0%	0.0%	0.0%
	p Proteobacteria;c Gammaproteobacteria;o JG36-GS-52	0.0%	0.0%	0.0%
	p Proteobacteria;c Gammaproteobacteria;o JG36-TzT-191	0.0%	0.0%	0.0%
	p Proteobacteria;c Gammaproteobacteria;o KI89A-clade	0.0%	0.0%	0.0%
	p Proteobacteria;c Gammaproteobacteria;o PLTA13	0.7%	1.3%	0.1%
	p Proteobacteria;c Gammaproteobacteria;o Pseudomonadales	0.5%	0.8%	0.2%
	p Proteobacteria;c Gammaproteobacteria;o R7C24	0.0%	0.0%	0.0%
	p Proteobacteria;c Gammaproteobacteria;o Salinisphaerales	0.1%	0.0%	0.1%
	p Proteobacteria;c Gammaproteobacteria;o Steroidobacterales	1.4%	2.0%	0.7%
	p Proteobacteria;c Gammaproteobacteria;o Xanthomonadales	0.8%	1.4%	0.1%
	p Rokubacteria;c NC10;o Rokubacteriales	1.2%	1.6%	0.9%
	p Thaumarchaeota;c Group-1.1c;Other	0.0%	0.0%	0.0%
	p Thaumarchaeota;c Nitrososphaeria;o Nitrosopumilales	0.0%	0.0%	0.0%
	p Thaumarchaeota;c Nitrososphaeria;o Nitrososphaerales	1.3%	1.3%	1.3%
	p Thaumarchaeota;c Nitrososphaeria;o Nitrosotaleales	0.0%	0.0%	0.0%
	p Verrucomicrobia;c Verrucomicrobiae;Other	0.0%	0.0%	0.0%
	p Verrucomicrobia;c Verrucomicrobiae;o Chthoniobacterales	2.1%	1.3%	2.8%
	p Verrucomicrobia;c Verrucomicrobiae;o Methylacidiphilales	0.0%	0.0%	0.0%
	p Verrucomicrobia;c Verrucomicrobiae;o Opitutales	1.4%	2.5%	0.3%
	p Verrucomicrobia;c Verrucomicrobiae;o Pedosphaerales	2.6%	1.6%	3.6%
	p Verrucomicrobia;c Verrucomicrobiae;o Verrucomicrobiales	0.1%	0.1%	0.1%
	p WPS-2;Other;Other	0.0%	0.0%	0.0%
	p Zixibacteria;Other;Other	0.0%	0.0%	0.0%
	p Zixibacteria;c uncultured-bacterium;o uncultured-bacterium	0.0%	0.0%	0.0%

Taxonomy Summary. Current Level:

		Total	G1	G2
Legend	Taxonomy	%	%	%
	Unclassified;Other;Other;Other	0.5%	0.7%	0.3%
	p Acidobacteria;Other;Other;Other	0.1%	0.2%	0.1%
	p Acidobacteria;c Acidobacteriia;o Acidobacteriales;Other	0.1%	0.0%	0.1%
	p Acidobacteria;c Acidobacteriia;o Acidobacteriales;f Acidobacteriaceae-(Subgroup-1)	0.0%	0.0%	0.0%
	p Acidobacteria;c Acidobacteriia;o Acidobacteriales;f Koribacteraceae	0.1%	0.0%	0.1%
	p Acidobacteria;c Acidobacteriia;o Acidobacteriales;f uncultured	0.2%	0.1%	0.2%
	p Acidobacteria;c Acidobacteriia;o Solibacterales;f Solibacteraceae-(Subgroup-3)	1.8%	1.4%	2.3%
	p Acidobacteria;c Acidobacteriia;o Subgroup-12;f uncultured-bacterium	0.0%	0.0%	0.0%
	p Acidobacteria;c Acidobacteriia;o Subgroup-13;f uncultured-Acidobacteria-bacterium	0.0%	0.0%	0.0%
	p Acidobacteria;c Acidobacteriia;o Subgroup-2;Other	0.0%	0.0%	0.0%
	p Acidobacteria;c Acidobacteriia;o Subgroup-2;f uncultured-Acidobacteria-bacterium	0.0%	0.0%	0.0%
	p Acidobacteria;c Acidobacteriia;o Subgroup-2;f uncultured-bacterium	0.1%	0.1%	0.0%
	p Acidobacteria;c Acidobacteriia;o Subgroup-2;f uncultured-forest-soil-bacterium	0.0%	0.0%	0.0%
	p Acidobacteria;c Acidobacteriia;o Subgroup-2;f uncultured-soil-bacterium	0.0%	0.0%	0.0%
	p Acidobacteria;c Blastocatellia-(Subgroup-4);Other;Other	2.6%	1.6%	3.6%
	p Acidobacteria;c Blastocatellia-(Subgroup-4);o 11-24;Other	0.1%	0.0%	0.1%
	p Acidobacteria;c Blastocatellia-(Subgroup-4);o 11-24;f uncultured-Acidobacteria-bacterium	0.2%	0.2%	0.2%
	p Acidobacteria;c Blastocatellia-(Subgroup-4);o 11-24;f uncultured-Acidobacteriales-bacterium	0.0%	0.0%	0.0%
	p Acidobacteria;c Blastocatellia-(Subgroup-4);o 11-24;f uncultured-bacterium	0.1%	0.2%	0.1%
	p Acidobacteria;c Blastocatellia-(Subgroup-4);o Blastocatellales;f Blastocatellaceae	0.7%	0.5%	0.9%
	p Acidobacteria;c Blastocatellia-(Subgroup-4);o DS-100;Other	0.0%	0.0%	0.0%
	p Acidobacteria;c Blastocatellia-(Subgroup-4);o DS-100;f uncultured-Acidobacteria-bacterium	0.0%	0.0%	0.0%
	p Acidobacteria;c Blastocatellia-(Subgroup-4);o DS-100;f uncultured-bacterium	0.0%	0.0%	0.0%
	p Acidobacteria;c Blastocatellia-(Subgroup-4);o Elev-16S-573;Other	5.1%	2.3%	8.0%
	p Acidobacteria;c Blastocatellia-(Subgroup-4);o Elev-16S-573;f uncultured-Acidobacteria-bacterium	0.0%	0.0%	0.0%
	p Acidobacteria;c Blastocatellia-(Subgroup-4);o Elev-16S-573;f uncultured-bacterium	0.7%	0.8%	0.7%
	p Acidobacteria;c Holophagae;o Holophagales;f Holophagaceae	0.0%	0.0%	0.0%
	p Acidobacteria;c Holophagae;o Subgroup-7;Other	0.2%	0.2%	0.2%
	p Acidobacteria;c Holophagae;o Subgroup-7;f uncultured-Acidobacteria-bacterium	0.0%	0.0%	0.0%
	p Acidobacteria;c Holophagae;o Subgroup-7;f uncultured-Acidobacteriales-bacterium	0.0%	0.0%	0.0%
	p Acidobacteria;c Holophagae;o Subgroup-7;f uncultured-Acidobacterium-sp.	0.0%	0.0%	0.0%
	p Acidobacteria;c Holophagae;o Subgroup-7;f uncultured-bacterium	0.1%	0.2%	0.1%
	p Acidobacteria;c Subgroup-11;Other;Other	0.1%	0.1%	0.1%
	p Acidobacteria;c Subgroup-11;o uncultured-bacterium;f uncultured-bacterium	0.0%	0.0%	0.0%
	p Acidobacteria;c Subgroup-15;Other;Other	0.0%	0.0%	0.0%
	p Acidobacteria;c Subgroup-15;o uncultured-bacterium;f uncultured-bacterium	0.0%	0.0%	0.0%
	p Acidobacteria;c Subgroup-17;Other;Other	0.1%	0.1%	0.0%

p Acidobacteria;c Subgroup-17;o uncultured-Acidobacteria-bacterium;f uncultured-Acidobacteria-bacterium	0.0%	0.0%	0.0%
p Acidobacteria;c Subgroup-17;o uncultured-Acidobacteriales-bacterium;f uncultured-Acidobacteriales-bacterium	0.0%	0.0%	0.0%
p Acidobacteria;c Subgroup-17;o uncultured-bacterium;f uncultured-bacterium	0.1%	0.1%	0.0%
p Acidobacteria;c Subgroup-18;Other;Other	0.0%	0.0%	0.0%
p Acidobacteria;c Subgroup-18;o uncultured-bacterium;f uncultured-bacterium	0.0%	0.0%	0.0%
p Acidobacteria;c Subgroup-20;o uncultured-bacterium;f uncultured-bacterium	0.0%	0.0%	0.0%
p Acidobacteria;c Subgroup-22;Other;Other	0.1%	0.1%	0.1%
p Acidobacteria;c Subgroup-22;o uncultured-Acidobacterium-sp.;f uncultured-Acidobacterium-sp.	0.0%	0.1%	0.0%
p Acidobacteria;c Subgroup-22;o uncultured-bacterium;f uncultured-bacterium	0.1%	0.2%	0.1%
p Acidobacteria;c Subgroup-25;Other;Other	0.0%	0.1%	0.0%
p Acidobacteria;c Subgroup-25;o uncultured-Acidobacteria-bacterium;f uncultured-Acidobacteria-bacterium	0.0%	0.0%	0.0%
p Acidobacteria;c Subgroup-25;o uncultured-bacterium;f uncultured-bacterium	0.0%	0.0%	0.0%
p Acidobacteria;c Subgroup-25;o uncultured-soil-bacterium;f uncultured-soil-bacterium	0.0%	0.0%	0.0%
p Acidobacteria;c Subgroup-5;Other;Other	0.0%	0.0%	0.0%
p Acidobacteria;c Subgroup-5;o uncultured-Acidobacteria-bacterium;f uncultured-Acidobacteria-bacterium	0.2%	0.2%	0.2%
p Acidobacteria;c Subgroup-5;o uncultured-Acidobacterium-sp.;f uncultured-Acidobacterium-sp.	0.0%	0.0%	0.0%
p Acidobacteria;c Subgroup-5;o uncultured-bacterium;f uncultured-bacterium	0.2%	0.2%	0.2%
p Acidobacteria;c Subgroup-6;Other;Other	5.4%	6.2%	4.6%
p Acidobacteria;c Subgroup-6;o Acidobacteria-bacterium-WX90;f Acidobacteria-bacterium-WX90	0.0%	0.0%	0.0%
p Acidobacteria;c Subgroup-6;o Unknown-Order;f Unknown-Family	0.0%	0.0%	0.0%
p Acidobacteria;c Subgroup-6;o uncultured-Acidobacteria-bacterium;f uncultured-Acidobacteria-bacterium	0.2%	0.2%	0.2%
p Acidobacteria;c Subgroup-6;o uncultured-Acidobacteriaceae-bacterium;f uncultured-Acidobacteriaceae-bacterium	0.0%	0.0%	0.0%
p Acidobacteria;c Subgroup-6;o uncultured-Acidobacteriales-bacterium;f uncultured-Acidobacteriales-bacterium	0.1%	0.1%	0.1%
p Acidobacteria;c Subgroup-6;o uncultured-Acidobacterium-sp.;f uncultured-Acidobacterium-sp.	0.0%	0.0%	0.0%
p Acidobacteria;c Subgroup-6;o uncultured-Holophagae-bacterium;f uncultured-Holophagae-bacterium	0.0%	0.0%	0.0%
p Acidobacteria;c Subgroup-6;o uncultured-bacterium;f uncultured-bacterium	0.8%	0.9%	0.8%
p Acidobacteria;c Subgroup-6;o uncultured-beta-proteobacterium;f uncultured-beta-proteobacterium	0.0%	0.0%	0.0%
p Acidobacteria;c Subgroup-6;o uncultured-proteobacterium;f uncultured-proteobacterium	0.0%	0.0%	0.0%
p Acidobacteria;c Subgroup-9;Other;Other	0.0%	0.0%	0.0%
p Actinobacteria;Other;Other;Other	0.0%	0.0%	0.1%
p Actinobacteria;c 0319-7L14;Other;Other	0.0%	0.0%	0.1%
p Actinobacteria;c 0319-7L14;o uncultured-bacterium;f uncultured-bacterium	0.0%	0.0%	0.0%
p Actinobacteria;c Acidimicrobiia;Other;Other	0.3%	0.3%	0.3%
p Actinobacteria;c Acidimicrobiia;o Acidimicrobiales;f Acidimicrobiaceae	0.0%	0.0%	0.0%
p Actinobacteria;c Acidimicrobiia;o Actinomarinales;f uncultured	0.7%	1.3%	0.1%
p Actinobacteria;c Acidimicrobiia;o IMCC26256;Other	0.4%	0.4%	0.3%
p Actinobacteria;c Acidimicrobiia;o IMCC26256;f uncultured-Acidimicrobiales-bacterium	0.0%	0.0%	0.0%
p Actinobacteria;c Acidimicrobiia;o IMCC26256;f uncultured-Acidimicrobidae-bacterium	0.0%	0.0%	0.0%
p Actinobacteria;c Acidimicrobiia;o IMCC26256;f uncultured-actinobacterium	0.0%	0.0%	0.1%
p Actinobacteria;c Acidimicrobiia;o IMCC26256;f uncultured-bacterium	0.0%	0.0%	0.0%
p Actinobacteria;c Acidimicrobiia;o IMCC26256;f uncultured-organism	0.0%	0.0%	0.0%
p Actinobacteria;c Acidimicrobiia;o Microtrichales;Other	0.1%	0.1%	0.1%
p Actinobacteria;c Acidimicrobiia;o Microtrichales;f Iamiaceae	0.2%	0.2%	0.2%
p Actinobacteria;c Acidimicrobiia;o Microtrichales;f Ilumatobacteraceae	0.2%	0.3%	0.2%
p Actinobacteria;c Acidimicrobiia;o Microtrichales;f Microtrichaceae	0.0%	0.0%	0.0%
p Actinobacteria;c Acidimicrobiia;o Microtrichales;f uncultured	0.2%	0.2%	0.2%
p Actinobacteria;c Acidimicrobiia;o uncultured;Other	0.3%	0.2%	0.5%
p Actinobacteria;c Acidimicrobiia;o uncultured;f uncultured-Acidimicrobidae-bacterium	0.0%	0.0%	0.1%
p Actinobacteria;c Acidimicrobiia;o uncultured;f uncultured-actinobacterium	0.0%	0.0%	0.0%
p Actinobacteria;c Acidimicrobiia;o uncultured;f uncultured-bacterium	0.1%	0.0%	0.1%
p Actinobacteria;c Acidimicrobiia;o uncultured;f uncultured-soil-bacterium	0.0%	0.0%	0.1%
p Actinobacteria;c Actinobacteria;Other;Other	0.0%	0.0%	0.0%
p Actinobacteria;c Actinobacteria;o Corynebacteriales;f Mycobacteriaceae	0.3%	0.1%	0.5%
p Actinobacteria;c Actinobacteria;o Corynebacteriales;f Nocardiaceae	1.1%	0.5%	1.7%
p Actinobacteria;c Actinobacteria;o Frankiales;f Cryptosporangiaceae	0.1%	0.0%	0.1%
p Actinobacteria;c Actinobacteria;o Frankiales;f Frankiaceae	0.0%	0.0%	0.0%
p Actinobacteria;c Actinobacteria;o Frankiales;f Geodermatophilaceae	0.0%	0.0%	0.1%
p Actinobacteria;c Actinobacteria;o Frankiales;f Nakamurellaceae	0.0%	0.0%	0.0%
p Actinobacteria;c Actinobacteria;o Frankiales;f Sporichthyaceae	0.0%	0.0%	0.1%
p Actinobacteria;c Actinobacteria;o Frankiales;f uncultured	0.0%	0.0%	0.0%
p Actinobacteria;c Actinobacteria;o Glycomycetales;f Glycomycetaceae	1.0%	1.9%	0.1%
p Actinobacteria;c Actinobacteria;o Kineosporiales;f Kineosporiaceae	0.0%	0.0%	0.0%
p Actinobacteria;c Actinobacteria;o Micrococcales;Other	0.0%	0.0%	0.0%
p Actinobacteria;c Actinobacteria;o Micrococcales;f Cellulomonadaceae	0.0%	0.0%	0.0%
p Actinobacteria;c Actinobacteria;o Micrococcales;f Demequinaceae	0.0%	0.0%	0.0%
p Actinobacteria;c Actinobacteria;o Micrococcales;f Intrasporangiaceae	0.1%	0.1%	0.1%
p Actinobacteria;c Actinobacteria;o Micrococcales;f Microbacteriaceae	0.1%	0.2%	0.1%
p Actinobacteria;c Actinobacteria;o Micrococcales;f Micrococcaceae	0.2%	0.0%	0.3%
p Actinobacteria;c Actinobacteria;o Micrococcales;f Promicromonosporaceae	0.3%	0.5%	0.0%

p Actinobacteria;c Actinobacteria;o Micromonosporales;f Micromonosporaceae	0.5%	0.5%	0.5%
p Actinobacteria;c Actinobacteria;o Propionibacteriales;f Nocardioidaceae	1.6%	1.6%	1.5%
p Actinobacteria;c Actinobacteria;o Propionibacteriales;f Propionibacteriaceae	0.0%	0.0%	0.0%
p Actinobacteria;c Actinobacteria;o Pseudonocardiales;f Pseudonocardiaceae	0.3%	0.2%	0.4%
p Actinobacteria;c Actinobacteria;o Streptomycetales;f Streptomycetaceae	7.6%	8.0%	7.3%
p Actinobacteria;c Actinobacteria;o Streptosporangiales;f Streptosporangiaceae	0.1%	0.2%	0.0%
p Actinobacteria;c Actinobacteria;o Streptosporangiales;f Thermomonosporaceae	0.0%	0.0%	0.0%
p Actinobacteria;c MB-A2-108;Other;Other	0.1%	0.1%	0.1%
p Actinobacteria;c MB-A2-108;o uncultured-actinobacterium;f uncultured-actinobacterium	0.0%	0.0%	0.0%
p Actinobacteria;c MB-A2-108;o uncultured-bacterium;f uncultured-bacterium	0.6%	0.7%	0.4%
p Actinobacteria;c MB-A2-108;o uncultured-bacterium-contig00016;f uncultured-bacterium-contig00016	0.0%	0.0%	0.0%
p Actinobacteria;c Nitriliruptoria;o Euzebyales;f Euzebyaceae	0.0%	0.0%	0.0%
p Actinobacteria;c Rubrobacteria;o Rubrobacterales;f Rubrobacteriaceae	0.1%	0.0%	0.1%
p Actinobacteria;c Thermoleophilia;Other;Other	0.1%	0.1%	0.1%
p Actinobacteria;c Thermoleophilia;o Gaiellales;Other	0.0%	0.0%	0.0%
p Actinobacteria;c Thermoleophilia;o Gaiellales;f uncultured	0.6%	0.7%	0.6%
p Actinobacteria;c Thermoleophilia;o Solirubrobacterales;f 67-14	0.3%	0.4%	0.2%
p Actinobacteria;c Thermoleophilia;o Solirubrobacterales;f Solirubrobacteraceae	0.2%	0.2%	0.2%
p Actinobacteria;c Thermoleophilia;o uncultured;f uncultured-bacterium	0.0%	0.0%	0.0%
p Armatimonadetes;c Fimbriimonadia;o Fimbriimonadales;f Fimbriimonadaceae	0.0%	0.0%	0.0%
p BRC1;Other;Other;Other	0.0%	0.0%	0.0%
p BRC1;c uncultured-bacterium;o uncultured-bacterium;f uncultured-bacterium	0.0%	0.0%	0.0%
p Bacteroidetes;c Bacteroidia;Other;Other	0.0%	0.0%	0.0%
p Bacteroidetes;c Bacteroidia;o Bacteroidetes-VC2.1-Bac22;Other	0.0%	0.0%	0.0%
p Bacteroidetes;c Bacteroidia;o Chitinophagales;Other	0.0%	0.0%	0.0%
p Bacteroidetes;c Bacteroidia;o Chitinophagales;f 37-13	0.1%	0.1%	0.1%
p Bacteroidetes;c Bacteroidia;o Chitinophagales;f Chitinophagaceae	8.0%	11.0%	5.1%
p Bacteroidetes;c Bacteroidia;o Chitinophagales;f Saprospiraceae	0.3%	0.2%	0.3%
p Bacteroidetes;c Bacteroidia;o Chitinophagales;f uncultured	0.0%	0.0%	0.0%
p Bacteroidetes;c Bacteroidia;o Cytophagales;f Cytophagaceae	0.0%	0.0%	0.0%
p Bacteroidetes;c Bacteroidia;o Cytophagales;f Hymenobacteraceae	0.1%	0.0%	0.2%
p Bacteroidetes;c Bacteroidia;o Cytophagales;f Microscillaceae	2.0%	2.0%	2.0%
p Bacteroidetes;c Bacteroidia;o Cytophagales;f Spirosomaceae	0.0%	0.0%	0.0%
p Bacteroidetes;c Bacteroidia;o Flavobacteriales;f Crocinitomicaceae	0.0%	0.0%	0.0%
p Bacteroidetes;c Bacteroidia;o Flavobacteriales;f Flavobacteriaceae	0.3%	0.1%	0.5%
p Bacteroidetes;c Bacteroidia;o Flavobacteriales;f NS9-marine-group	0.0%	0.0%	0.0%
p Bacteroidetes;c Bacteroidia;o Sphingobacteriales;Other	0.0%	0.0%	0.0%
p Bacteroidetes;c Bacteroidia;o Sphingobacteriales;f AKYH767	0.3%	0.2%	0.3%
p Bacteroidetes;c Bacteroidia;o Sphingobacteriales;f NS11-12-marine-group	0.0%	0.0%	0.0%
p Bacteroidetes;c Bacteroidia;o Sphingobacteriales;f Sphingobacteriaceae	0.3%	0.5%	0.1%
p Bacteroidetes;c Bacteroidia;o Sphingobacteriales;f env.OPS-17	0.3%	0.2%	0.4%
p Bacteroidetes;c Ignavibacteria;o Ignavibacteriales;f uncultured-bacterium	0.0%	0.0%	0.0%
p Bacteroidetes;c Ignavibacteria;o Kryptoniales;f BSV26	0.1%	0.1%	0.0%
p Bacteroidetes;c Ignavibacteria;o OPB56;Other	0.0%	0.0%	0.0%
p Bacteroidetes;c Ignavibacteria;o OPB56;f uncultured-bacterium	0.0%	0.0%	0.0%
p Bacteroidetes;c Ignavibacteria;o OPB56;f uncultured-bacterium-#0319-6E22	0.0%	0.0%	0.0%
p Bacteroidetes;c Ignavibacteria;o OPB56;f uncultured-bacterium-KF-JG30-B11	0.0%	0.0%	0.0%
p Bacteroidetes;c Ignavibacteria;o SJA-28;f uncultured-bacterium	0.0%	0.1%	0.0%
p Chloroflexi;Other;Other;Other	0.0%	0.0%	0.0%
p Chloroflexi;c AD3;o uncultured-bacterium;f uncultured-bacterium	0.0%	0.0%	0.0%
p Chloroflexi;c Anaerolineae;Other;Other	0.0%	0.0%	0.0%
p Chloroflexi;c Anaerolineae;o Anaerolineales;f Anaerolineaceae	1.2%	0.9%	1.5%
p Chloroflexi;c Anaerolineae;o Ardenticatenales;f uncultured	0.2%	0.1%	0.2%
p Chloroflexi;c Anaerolineae;o Caldilineales;f Caldilineaceae	0.0%	0.0%	0.1%
p Chloroflexi;c Anaerolineae;o RBG-13-54-9;Other	0.0%	0.0%	0.1%
p Chloroflexi;c Anaerolineae;o RBG-13-54-9;f uncultured-Caldilineaceae-bacterium	0.0%	0.0%	0.0%
p Chloroflexi;c Anaerolineae;o RBG-13-54-9;f uncultured-sludge-bacterium-A31	0.0%	0.0%	0.0%
p Chloroflexi;c Anaerolineae;o SBR1031;Other	0.1%	0.2%	0.0%
p Chloroflexi;c Anaerolineae;o SBR1031;f A4b	0.3%	0.2%	0.4%
p Chloroflexi;c Anaerolineae;o SBR1031;f uncultured-Caldilineaceae-bacterium	0.0%	0.0%	0.0%
p Chloroflexi;c Anaerolineae;o SBR1031;f uncultured-bacterium	0.0%	0.0%	0.0%
p Chloroflexi;c Anaerolineae;o SBR1031;f uncultured-soil-bacterium	0.0%	0.0%	0.0%
p Chloroflexi;c Anaerolineae;o uncultured-Bellilinea-sp.;f uncultured-Bellilinea-sp.	0.0%	0.0%	0.0%
p Chloroflexi;c Chloroflexia;o Chloroflexales;Other	0.0%	0.0%	0.0%
p Chloroflexi;c Chloroflexia;o Chloroflexales;f Chloroflexaceae	0.0%	0.0%	0.0%
p Chloroflexi;c Chloroflexia;o Chloroflexales;f Herpetosiphonaceae	0.0%	0.0%	0.0%
p Chloroflexi;c Chloroflexia;o Chloroflexales;f Roseiflexaceae	1.2%	0.4%	2.0%
p Chloroflexi;c Chloroflexia;o Kallotenuales;f AKIW781	0.0%	0.0%	0.0%
p Chloroflexi;c Chloroflexia;o Thermomicrobiales;f AKYG1722	0.1%	0.1%	0.2%

	p Chloroflexi;c Chloroflexia;o Thermomicrobiales;f JG30-KF-CM45	0.3%	0.2%	0.4%
	p Chloroflexi;c Chloroflexia;o Thermomicrobiales;f Thermomicrobiaceae	0.0%	0.0%	0.0%
	p Chloroflexi;c Dehalococcoidia;o S085;Other	0.2%	0.3%	0.1%
	p Chloroflexi;c Dehalococcoidia;o S085;f uncultured-Chloroflexi-bacterium	0.0%	0.0%	0.0%
	p Chloroflexi;c Dehalococcoidia;o S085;f uncultured-bacterium	0.1%	0.2%	0.1%
	p Chloroflexi;c Dehalococcoidia;o S085;f uncultured-soil-bacterium	0.0%	0.0%	0.0%
	p Chloroflexi;c Gitt-GS-136;Other;Other	0.0%	0.0%	0.0%
	p Chloroflexi;c Gitt-GS-136;o uncultured-bacterium;f uncultured-bacterium	0.3%	0.4%	0.1%
	p Chloroflexi;c JG30-KF-CM66;Other;Other	0.0%	0.0%	0.0%
	p Chloroflexi;c JG30-KF-CM66;o uncultured-Chloroflexi-bacterium;f uncultured-Chloroflexi-bacterium	0.0%	0.0%	0.0%
	p Chloroflexi;c JG30-KF-CM66;o uncultured-bacterium;f uncultured-bacterium	0.1%	0.1%	0.1%
	p Chloroflexi;c KD4-96;Other;Other	0.2%	0.3%	0.1%
	p Chloroflexi;c KD4-96;o uncultured-Chloroflexi-bacterium;f uncultured-Chloroflexi-bacterium	0.1%	0.1%	0.1%
	p Chloroflexi;c KD4-96;o uncultured-bacterium;f uncultured-bacterium	0.4%	0.5%	0.3%
	p Chloroflexi;c Ktedonobacteria;o C0119;Other	0.0%	0.0%	0.0%
	p Chloroflexi;c Ktedonobacteria;o C0119;f uncultured-soil-bacterium	0.0%	0.0%	0.0%
	p Chloroflexi;c Ktedonobacteria;o Ktedonobacterales;f Ktedonobacteraceae	0.0%	0.0%	0.1%
	p Chloroflexi;c OLB14;Other;Other	0.0%	0.0%	0.0%
	p Chloroflexi;c OLB14;o uncultured-bacterium;f uncultured-bacterium	0.1%	0.1%	0.1%
	p Chloroflexi;c OLB14;o uncultured-gamma-proteobacterium;f uncultured-gamma-proteobacterium	0.0%	0.0%	0.0%
	p Chloroflexi;c P2-11E;o uncultured-bacterium;f uncultured-bacterium	0.2%	0.1%	0.2%
	p Chloroflexi;c TK10;Other;Other	0.2%	0.2%	0.2%
	p Chloroflexi;c TK10;o uncultured-Chloroflexi-bacterium;f uncultured-Chloroflexi-bacterium	0.0%	0.0%	0.1%
	p Chloroflexi;c TK10;o uncultured-bacterium;f uncultured-bacterium	0.0%	0.1%	0.0%
	p Dadabacteria;c Dadabacteriia;o Dadabacteriales;f uncultured-soil-bacterium	0.0%	0.0%	0.0%
	p Dependentiae;c Babeliae;o Babeliales;Other	0.0%	0.0%	0.0%
	p Dependentiae;c Babeliae;o Babeliales;f Babeliaceae	0.0%	0.0%	0.0%
	p Dependentiae;c Babeliae;o Babeliales;f Vermiphilaceae	0.0%	0.0%	0.0%
	p Elusimicrobia;c Elusimicrobia;o MVP-88;Other	0.0%	0.0%	0.0%
	p Elusimicrobia;c Lineage-IIa;Other;Other	0.0%	0.0%	0.0%
	p Elusimicrobia;c Lineage-IIa;o uncultured-bacterium;f uncultured-bacterium	0.0%	0.1%	0.0%
	p Elusimicrobia;c Lineage-IIb;o uncultured-bacterium;f uncultured-bacterium	0.0%	0.0%	0.0%
	p Elusimicrobia;c Lineage-IIb;o uncultured-soil-bacterium;f uncultured-soil-bacterium	0.0%	0.0%	0.0%
	p Entotheonellaeota;c Entotheonellia;o Entotheonellales;f Entotheonellaceae	0.1%	0.1%	0.1%
	p Euryarchaeota;c Methanomicrobia;o Methanosarcinales;f Methanosarcinaceae	0.0%	0.0%	0.0%
	p Euryarchaeota;c Thermoplasmata;Other;Other	0.1%	0.1%	0.1%
	p Euryarchaeota;c Thermoplasmata;o Marine-Group-II;Other	0.2%	0.1%	0.2%
	p Euryarchaeota;c Thermoplasmata;o Marine-Group-II;f uncultured-archaeon	1.1%	0.6%	1.6%
	p Euryarchaeota;c Thermoplasmata;o Marine-Group-II;f uncultured-haloarchaeon	0.0%	0.0%	0.0%
	p Euryarchaeota;c Thermoplasmata;o Methanomassiliicoccales;f uncultured	0.5%	0.0%	0.9%
	p Euryarchaeota;c Thermoplasmata;o uncultured;Other	0.0%	0.0%	0.0%
	p Firmicutes;c Bacilli;o Bacillales;f Bacillaceae	2.7%	0.8%	4.6%
	p Firmicutes;c Bacilli;o Bacillales;f Paenibacillaceae	0.2%	0.1%	0.3%
	p Firmicutes;c Bacilli;o Bacillales;f Planococcaceae	0.0%	0.0%	0.0%
	p Firmicutes;c Bacilli;o Bacillales;f Thermoactinomycetaceae	0.0%	0.0%	0.0%
	p Firmicutes;c Clostridia;o Clostridiales;f Clostridiaceae-1	0.0%	0.0%	0.0%
	p Firmicutes;c Clostridia;o Clostridiales;f Heliobacteriaceae	0.0%	0.0%	0.0%
	p Firmicutes;c Clostridia;o Clostridiales;f Peptostreptococcaceae	0.0%	0.0%	0.0%
	p Firmicutes;c Clostridia;o Clostridiales;f Ruminococcaceae	0.0%	0.0%	0.0%
	p GAL15;c uncultured-bacterium;o uncultured-bacterium;f uncultured-bacterium	0.0%	0.0%	0.0%
	p Gemmatimonadetes;c AKAU4049;Other;Other	0.2%	0.3%	0.0%
	p Gemmatimonadetes;c BD2-11-terrestrial-group;Other;Other	0.0%	0.0%	0.0%
	p Gemmatimonadetes;c BD2-11-terrestrial-group;o uncultured-Gemmatimonadales-bacterium;f uncultured-Gemmatimonadales-bacterium	0.0%	0.0%	0.0%
	p Gemmatimonadetes;c BD2-11-terrestrial-group;o uncultured-Gemmatimonadetes-bacterium;f uncultured-Gemmatimonadetes-bacterium	0.0%	0.1%	0.0%
	p Gemmatimonadetes;c BD2-11-terrestrial-group;o uncultured-bacterium;f uncultured-bacterium	0.0%	0.0%	0.0%
	p Gemmatimonadetes;c BD2-11-terrestrial-group;o uncultured-soil-bacterium;f uncultured-soil-bacterium	0.0%	0.0%	0.0%
	p Gemmatimonadetes;c Gemmatimonadetes;o Gemmatimonadales;f Gemmatimonadaceae	2.6%	2.5%	2.8%
	p Gemmatimonadetes;c Longimicrobia;o Longimicrobiales;f Longimicrobiaceae	0.0%	0.0%	0.0%
	p Gemmatimonadetes;c S0134-terrestrial-group;Other;Other	0.1%	0.1%	0.1%
	p Gemmatimonadetes;c S0134-terrestrial-group;o uncultured-Gemmatimonadales-bacterium;f uncultured-Gemmatimonadales-bacterium	0.2%	0.2%	0.2%
	p Gemmatimonadetes;c S0134-terrestrial-group;o uncultured-Gemmatimonadetes-bacterium;f uncultured-Gemmatimonadetes-bacterium	0.0%	0.0%	0.0%
	p Gemmatimonadetes;c S0134-terrestrial-group;o uncultured-bacterium;f uncultured-bacterium	0.2%	0.2%	0.2%
	p Hydrogenedentes;c Hydrogenedentia;o Hydrogenedentiales;f Hydrogenedensaceae	0.0%	0.0%	0.0%
	p Latescibacteria;Other;Other;Other	0.3%	0.4%	0.1%
	p Latescibacteria;c Latescibacteria;o Latescibacterales;f Latescibacteraceae	0.2%	0.3%	0.0%
	p Latescibacteria;c uncultured-Acidobacterium-sp.;o uncultured-Acidobacterium-sp.;f uncultured-Acidobacterium-sp.	0.0%	0.0%	0.0%

p Latescibacteria;c uncultured-Fibrobacteres-bacterium;o uncultured-Fibrobacteres-bacterium;f uncultured-Fibrobacteres-bacterium	0.0%	0.0%	0.0%
p Latescibacteria;c uncultured-Pelobacter-sp.;o uncultured-Pelobacter-sp.;f uncultured-Pelobacter-sp.	0.0%	0.0%	0.0%
p Latescibacteria;c uncultured-bacterium;o uncultured-bacterium;f uncultured-bacterium	0.4%	0.5%	0.3%
p Latescibacteria;c uncultured-prokaryote;o uncultured-prokaryote;f uncultured-prokaryote	0.0%	0.0%	0.0%
p Latescibacteria;c uncultured-proteobacterium;o uncultured-proteobacterium;f uncultured-proteobacterium	0.0%	0.0%	0.0%
p Latescibacteria;c uncultured-soil-bacterium;o uncultured-soil-bacterium;f uncultured-soil-bacterium	0.0%	0.1%	0.0%
p Nanoarchaeaeota;c Nanohaloarchaeia;o Aenigmarchaeales;Other	0.0%	0.0%	0.0%
p Nanoarchaeaeota;c Nanohaloarchaeia;o Aenigmarchaeales;f uncultured-archaeon	0.0%	0.1%	0.0%
p Nanoarchaeaeota;c Nanohaloarchaeia;o Deep-Sea-Euryarchaeotic-Group(DSEG);Other	0.0%	0.0%	0.0%
p Nanoarchaeaeota;c Woesearchaeia;Other;Other	0.1%	0.2%	0.1%
p Nanoarchaeaeota;c Woesearchaeia;o Candidatus-Amesbacteria-bacterium-GW2011_GWC1_47_15;f Candidatus-Amesbacteria-bacterium-GW2011_GWC1_47_15	0.0%	0.0%	0.0%
p Nanoarchaeaeota;c Woesearchaeia;o Candidatus-Pacearchaeota-archaeon-RBG_19FT_COMBO_34_9;f Candidatus-Pacearchaeota-archaeon-RBG_19FT_COMBO_34_9	0.0%	0.0%	0.0%
p Nanoarchaeaeota;c Woesearchaeia;o Candidatus-Staskawiczbacteria-bacterium-RIFOXYA2_FULL_32_7;f Candidatus-Staskawiczbacteria-bacterium-RIFOXYA2_FULL_32_7	0.1%	0.1%	0.0%
p Nanoarchaeaeota;c Woesearchaeia;o uncultured-euryarchaeote;f uncultured-euryarchaeote	0.0%	0.0%	0.0%
p Nitrospirae;c Nitrospira;o Nitrospirales;f Nitrospiraceae	0.5%	0.4%	0.7%
p Patescibacteria;Other;Other;Other	0.0%	0.0%	0.0%
p Patescibacteria;c ABY1;Other;Other	0.0%	0.0%	0.0%
p Patescibacteria;c ABY1;o Candidatus-Kuenenbacteria;f uncultured-bacterium	0.0%	0.0%	0.1%
p Patescibacteria;c ABY1;o Candidatus-Magasanikbacteria;Other	0.0%	0.0%	0.0%
p Patescibacteria;c ABY1;o Candidatus-Magasanikbacteria;f uncultured-bacterium	0.0%	0.0%	0.0%
p Patescibacteria;c ABY1;o Candidatus-Uhrbacteria;Other	0.0%	0.0%	0.0%
p Patescibacteria;c Berkelbacteria;o uncultured-bacterium;f uncultured-bacterium	0.0%	0.0%	0.0%
p Patescibacteria;c Gracilibacteria;o Candidatus-Abawacabacteria;Other	0.0%	0.0%	0.0%
p Patescibacteria;c Gracilibacteria;o Candidatus-Abawacabacteria;f Candidatus-Abawacabacteria-bacterium-RBG_16_42_10	0.0%	0.0%	0.0%
p Patescibacteria;c Gracilibacteria;o Candidatus-Peribacteria;f Candidatus-Peribacteria-bacterium-RIFCSPHIGHO2_02_FULL_53_20	0.0%	0.0%	0.0%
p Patescibacteria;c Microgenomatia;o Candidatus-Woesebacteria;Other	0.0%	0.0%	0.0%
p Patescibacteria;c Parcubacteria;Other;Other	0.0%	0.0%	0.0%
p Patescibacteria;c Parcubacteria;o Candidatus-Azambacteria;Other	0.0%	0.0%	0.0%
p Patescibacteria;c Parcubacteria;o Candidatus-Azambacteria;f uncultured-bacterium	0.0%	0.0%	0.0%
p Patescibacteria;c Parcubacteria;o Candidatus-Kaiserbacteria;Other	0.0%	0.0%	0.0%
p Patescibacteria;c Parcubacteria;o Candidatus-Nomurabacteria;Other	0.0%	0.0%	0.0%
p Patescibacteria;c Parcubacteria;o Candidatus-Terrybacteria;f uncultured-bacterium	0.0%	0.0%	0.0%
p Patescibacteria;c Parcubacteria;o Candidatus-Yanofskybacteria;Other	0.0%	0.0%	0.0%
p Patescibacteria;c Parcubacteria;o Candidatus-Yanofskybacteria;f Candidatus-Yanofskybacteria-bacterium-RIFCSPHIGHO2_02_FULL_46_19	0.0%	0.0%	0.0%
p Patescibacteria;c Parcubacteria;o Candidatus-Yanofskybacteria;f uncultured-bacterium	0.0%	0.0%	0.0%
p Patescibacteria;c Parcubacteria;o Candidatus-Yanofskybacteria;f uncultured-deep-sea-bacterium	0.0%	0.0%	0.0%
p Patescibacteria;c Parcubacteria;o GWA2-38-13b;f uncultured-bacterium	0.0%	0.0%	0.0%
p Patescibacteria;c Parcubacteria;o GWA2-38-13b;f uncultured-deep-sea-bacterium	0.0%	0.0%	0.0%
p Patescibacteria;c Parcubacteria;o uncultured-bacterium;f uncultured-bacterium	0.0%	0.0%	0.0%
p Patescibacteria;c Saccharimonadia;o Saccharimonadales;Other	0.2%	0.2%	0.3%
p Patescibacteria;c Saccharimonadia;o Saccharimonadales;f Saccharimonadaceae	0.1%	0.0%	0.2%
p Patescibacteria;c Saccharimonadia;o Saccharimonadales;f uncultured-Candidatus-Saccharibacteria-bacterium	0.0%	0.0%	0.1%
p Patescibacteria;c Saccharimonadia;o Saccharimonadales;f uncultured-bacterium	0.1%	0.1%	0.1%
p Patescibacteria;c WWE3;Other;Other	0.0%	0.0%	0.0%
p Planctomycetes;Other;Other;Other	0.0%	0.0%	0.0%
p Planctomycetes;c BD7-11;o uncultured-Planctomycetales-bacterium;f uncultured-Planctomycetales-bacterium	0.0%	0.0%	0.0%
p Planctomycetes;c BD7-11;o uncultured-bacterium;f uncultured-bacterium	0.0%	0.0%	0.0%
p Planctomycetes;c OM190;Other;Other	0.0%	0.0%	0.0%
p Planctomycetes;c OM190;o uncultured-bacterium;f uncultured-bacterium	0.0%	0.0%	0.0%
p Planctomycetes;c OM190;o uncultured-soil-bacterium;f uncultured-soil-bacterium	0.0%	0.0%	0.0%
p Planctomycetes;c Phycisphaerae;Other;Other	0.2%	0.2%	0.2%
p Planctomycetes;c Phycisphaerae;o CCM11a;Other	0.1%	0.0%	0.1%
p Planctomycetes;c Phycisphaerae;o CCM11a;f uncultured-Planctomycetia-bacterium	0.0%	0.0%	0.0%
p Planctomycetes;c Phycisphaerae;o CCM11a;f uncultured-bacterium	0.1%	0.2%	0.1%
p Planctomycetes;c Phycisphaerae;o Phycisphaerales;Other	0.0%	0.0%	0.0%
p Planctomycetes;c Phycisphaerae;o Phycisphaerales;f Phycisphaeraceae	0.1%	0.1%	0.1%
p Planctomycetes;c Phycisphaerae;o Pla1-lineage;Other	0.0%	0.0%	0.0%
p Planctomycetes;c Phycisphaerae;o Pla1-lineage;f uncultured-bacterium	0.0%	0.0%	0.0%
p Planctomycetes;c Phycisphaerae;o Tepidisphaerales;f CPla-3-termite-group	0.0%	0.0%	0.0%
p Planctomycetes;c Phycisphaerae;o Tepidisphaerales;f Tepidisphaeraceae	0.0%	0.0%	0.0%
p Planctomycetes;c Phycisphaerae;o Tepidisphaerales;f WD2101-soil-group	1.1%	0.8%	1.4%
p Planctomycetes;c Phycisphaerae;o mle1-8;Other	0.0%	0.0%	0.0%
p Planctomycetes;c Phycisphaerae;o mle1-8;f uncultured-bacterium	0.1%	0.0%	0.1%

p Planctomycetes;c Pla3-lineage;Other;Other	0.0%	0.0%	0.0%
p Planctomycetes;c Pla4-lineage;Other;Other	0.0%	0.0%	0.0%
p Planctomycetes;c Pla4-lineage;o uncultured-bacterium;f uncultured-bacterium	0.0%	0.0%	0.0%
p Planctomycetes;c Pla4-lineage;o uncultured-prokaryote;f uncultured-prokaryote	0.0%	0.0%	0.0%
p Planctomycetes;c Planctomycetacia;o Gemmatales;f Gemmataceae	0.2%	0.1%	0.2%
p Planctomycetes;c Planctomycetacia;o Pirellulales;f Pirellulaceae	0.2%	0.2%	0.2%
p Planctomycetes;c Planctomycetacia;o Planctomycetales;Other	0.0%	0.0%	0.0%
p Planctomycetes;c Planctomycetacia;o Planctomycetales;f Rubinisphaeraceae	0.0%	0.0%	0.0%
p Planctomycetes;c Planctomycetacia;o Planctomycetales;f Schlesneriaceae	0.0%	0.0%	0.0%
p Planctomycetes;c Planctomycetacia;o Planctomycetales;f uncultured	0.0%	0.0%	0.0%
p Planctomycetes;c Planctomycetacia;o uncultured;f uncultured-bacterium	0.0%	0.0%	0.0%
p Proteobacteria;Other;Other;Other	0.0%	0.0%	0.0%
p Proteobacteria;c Alphaproteobacteria;Other;Other	1.8%	1.7%	2.0%
p Proteobacteria;c Alphaproteobacteria;o Azospirillales;f Azospirillaceae	0.0%	0.0%	0.0%
p Proteobacteria;c Alphaproteobacteria;o Azospirillales;f Inquilinaceae	0.0%	0.0%	0.0%
p Proteobacteria;c Alphaproteobacteria;o Azospirillales;f uncultured	0.0%	0.1%	0.0%
p Proteobacteria;c Alphaproteobacteria;o Caulobacterales;Other	0.0%	0.0%	0.0%
p Proteobacteria;c Alphaproteobacteria;o Caulobacterales;f Caulobacteraceae	0.1%	0.2%	0.1%
p Proteobacteria;c Alphaproteobacteria;o Caulobacterales;f Hyphomonadaceae	0.1%	0.2%	0.1%
p Proteobacteria;c Alphaproteobacteria;o Caulobacterales;f Parvularculaceae	0.0%	0.0%	0.0%
p Proteobacteria;c Alphaproteobacteria;o Elsterales;f Elsteraceae	0.0%	0.0%	0.0%
p Proteobacteria;c Alphaproteobacteria;o Elsterales;f uncultured	0.1%	0.1%	0.1%
p Proteobacteria;c Alphaproteobacteria;o Holosporales;f Holosporaceae	0.0%	0.0%	0.0%
p Proteobacteria;c Alphaproteobacteria;o Micropepsales;f Micropepsaceae	0.1%	0.0%	0.1%
p Proteobacteria;c Alphaproteobacteria;o Reyranellales;f Reyranellaceae	0.2%	0.3%	0.2%
p Proteobacteria;c Alphaproteobacteria;o Rhizobiales;Other	0.0%	0.1%	0.0%
p Proteobacteria;c Alphaproteobacteria;o Rhizobiales;f A0839	0.0%	0.0%	0.1%
p Proteobacteria;c Alphaproteobacteria;o Rhizobiales;f Amb-16S-1323	0.0%	0.0%	0.0%
p Proteobacteria;c Alphaproteobacteria;o Rhizobiales;f Beijerinckiaceae	0.1%	0.1%	0.1%
p Proteobacteria;c Alphaproteobacteria;o Rhizobiales;f D05-2	0.0%	0.0%	0.0%
p Proteobacteria;c Alphaproteobacteria;o Rhizobiales;f Devosiaceae	0.2%	0.3%	0.1%
p Proteobacteria;c Alphaproteobacteria;o Rhizobiales;f Hyphomicrobiaceae	0.3%	0.6%	0.1%
p Proteobacteria;c Alphaproteobacteria;o Rhizobiales;f KF-JG30-B3	0.8%	0.7%	0.9%
p Proteobacteria;c Alphaproteobacteria;o Rhizobiales;f Labraceae	0.0%	0.0%	0.0%
p Proteobacteria;c Alphaproteobacteria;o Rhizobiales;f Methyloligellaceae	0.4%	0.4%	0.4%
p Proteobacteria;c Alphaproteobacteria;o Rhizobiales;f Rhizobiaceae	0.7%	0.9%	0.5%
p Proteobacteria;c Alphaproteobacteria;o Rhizobiales;f Rhizobiales-Incertae-Sedis	0.3%	0.2%	0.3%
p Proteobacteria;c Alphaproteobacteria;o Rhizobiales;f Rhodomicrobiaceae	0.0%	0.0%	0.0%
p Proteobacteria;c Alphaproteobacteria;o Rhizobiales;f Xanthobacteraceae	2.3%	2.5%	2.2%
p Proteobacteria;c Alphaproteobacteria;o Rhizobiales;f uncultured	0.1%	0.2%	0.1%
p Proteobacteria;c Alphaproteobacteria;o Rhodobacterales;f Rhodobacteraceae	0.0%	0.0%	0.0%
p Proteobacteria;c Alphaproteobacteria;o Rhodospirillales;f Rhodospirillaceae	0.0%	0.0%	0.1%
p Proteobacteria;c Alphaproteobacteria;o Rhodospirillales;f uncultured	0.0%	0.0%	0.0%
p Proteobacteria;c Alphaproteobacteria;o Rhodovibrionales;f Fodinicurvataceae	0.0%	0.0%	0.0%
p Proteobacteria;c Alphaproteobacteria;o Sneathiellales;f Sneathiellaceae	0.0%	0.0%	0.0%
p Proteobacteria;c Alphaproteobacteria;o Sphingomonadales;f Sphingomonadaceae	1.9%	2.3%	1.6%
p Proteobacteria;c Alphaproteobacteria;o Tistrellales;f Geminicoccaceae	0.0%	0.0%	0.0%
p Proteobacteria;c Alphaproteobacteria;o uncultured;Other	0.2%	0.2%	0.2%
p Proteobacteria;c Alphaproteobacteria;o uncultured;f uncultured-Acetobacteraceae-bacterium	0.0%	0.0%	0.0%
p Proteobacteria;c Alphaproteobacteria;o uncultured;f uncultured-Rhodospirillaceae-bacterium	0.0%	0.0%	0.0%
p Proteobacteria;c Alphaproteobacteria;o uncultured;f uncultured-Rhodospirillales-bacterium	0.0%	0.0%	0.0%
p Proteobacteria;c Alphaproteobacteria;o uncultured;f uncultured-Stella-sp.	0.0%	0.0%	0.0%
p Proteobacteria;c Alphaproteobacteria;o uncultured;f uncultured-bacterium	0.1%	0.1%	0.1%
p Proteobacteria;c Alphaproteobacteria;o uncultured;f uncultured-soil-bacterium	0.0%	0.1%	0.0%
p Proteobacteria;c Deltaproteobacteria;Other;Other	0.2%	0.3%	0.2%
p Proteobacteria;c Deltaproteobacteria;o Bdellovibrionales;f Bdellovibrionaceae	0.0%	0.1%	0.0%
p Proteobacteria;c Deltaproteobacteria;o Desulfarculales;f Desulfarculaceae	0.2%	0.1%	0.2%
p Proteobacteria;c Deltaproteobacteria;o Desulfuromonadales;Other	0.0%	0.0%	0.0%
p Proteobacteria;c Deltaproteobacteria;o Desulfuromonadales;f Geobacteraceae	0.0%	0.0%	0.1%
p Proteobacteria;c Deltaproteobacteria;o MBNT15;Other	0.1%	0.0%	0.2%
p Proteobacteria;c Deltaproteobacteria;o MBNT15;f uncultured-bacterium	0.0%	0.0%	0.0%
p Proteobacteria;c Deltaproteobacteria;o MBNT15;f uncultured-proteobacterium	0.0%	0.0%	0.1%
p Proteobacteria;c Deltaproteobacteria;o Myxococcales;Other	0.1%	0.1%	0.0%
p Proteobacteria;c Deltaproteobacteria;o Myxococcales;f Archangiaceae	0.0%	0.0%	0.0%
p Proteobacteria;c Deltaproteobacteria;o Myxococcales;f BIrii41	0.2%	0.2%	0.2%
p Proteobacteria;c Deltaproteobacteria;o Myxococcales;f Blfdi19	0.0%	0.0%	0.0%
p Proteobacteria;c Deltaproteobacteria;o Myxococcales;f Eel-36e1D6	0.0%	0.0%	0.0%
p Proteobacteria;c Deltaproteobacteria;o Myxococcales;f Haliangiaceae	0.3%	0.4%	0.3%
p Proteobacteria;c Deltaproteobacteria;o Myxococcales;f Nannocystaceae	0.0%	0.0%	0.0%

p Proteobacteria;c Deltaproteobacteria;o Myxococcales;f P3OB-42	0.0%	0.0%	0.1%
p Proteobacteria;c Deltaproteobacteria;o Myxococcales;f Phaselicystidaceae	0.0%	0.0%	0.0%
p Proteobacteria;c Deltaproteobacteria;o Myxococcales;f Polyangiaceae	0.0%	0.1%	0.0%
p Proteobacteria;c Deltaproteobacteria;o Myxococcales;f Sandaracinaceae	0.1%	0.1%	0.1%
p Proteobacteria;c Deltaproteobacteria;o Myxococcales;f UASB-TL25	0.0%	0.0%	0.0%
p Proteobacteria;c Deltaproteobacteria;o Myxococcales;f Vulgatibacteraceae	0.0%	0.0%	0.0%
p Proteobacteria;c Deltaproteobacteria;o Myxococcales;f bacteriap25	0.6%	0.7%	0.6%
p Proteobacteria;c Deltaproteobacteria;o Myxococcales;f mle1-27	0.0%	0.0%	0.0%
p Proteobacteria;c Deltaproteobacteria;o Myxococcales;f uncultured	0.0%	0.0%	0.0%
p Proteobacteria;c Deltaproteobacteria;o NB1-j;Other	0.0%	0.0%	0.0%
p Proteobacteria;c Deltaproteobacteria;o NB1-j;f uncultured-Green-Bay-ferromanganous-micronodule-bacterium-MND4	0.0%	0.0%	0.0%
p Proteobacteria;c Deltaproteobacteria;o NB1-j;f uncultured-bacterium	0.3%	0.4%	0.2%
p Proteobacteria;c Deltaproteobacteria;o NB1-j;f uncultured-proteobacterium	0.0%	0.0%	0.0%
p Proteobacteria;c Deltaproteobacteria;o NB1-j;f uncultured-soil-bacterium	0.0%	0.0%	0.0%
p Proteobacteria;c Deltaproteobacteria;o Oligoflexales;f Oligoflexaceae	0.0%	0.0%	0.0%
p Proteobacteria;c Deltaproteobacteria;o RCP2-54;f uncultured-bacterium	0.0%	0.0%	0.0%
p Proteobacteria;c Deltaproteobacteria;o RCP2-54;f uncultured-prokaryote	0.0%	0.0%	0.0%
p Proteobacteria;c Deltaproteobacteria;o SAR324-clade(Marine-group-B);f bacterium-enrichment-culture-clone-B30(2011)	0.0%	0.0%	0.0%
p Proteobacteria;c Deltaproteobacteria;o Syntrophobacterales;f Syntrophaceae	0.1%	0.0%	0.1%
p Proteobacteria;c Gammaproteobacteria;Other;Other	0.5%	0.6%	0.4%
p Proteobacteria;c Gammaproteobacteria;o Acidiferrobacterales;f Acidiferrobacteraceae	0.0%	0.0%	0.0%
p Proteobacteria;c Gammaproteobacteria;o Aeromonadales;f Aeromonadaceae	0.0%	0.0%	0.0%
p Proteobacteria;c Gammaproteobacteria;o Betaproteobacteriales;Other	1.7%	1.4%	2.1%
p Proteobacteria;c Gammaproteobacteria;o Betaproteobacteriales;f A21b	0.0%	0.0%	0.0%
p Proteobacteria;c Gammaproteobacteria;o Betaproteobacteriales;f B1-7BS	0.1%	0.1%	0.1%
p Proteobacteria;c Gammaproteobacteria;o Betaproteobacteriales;f Burkholderiaceae	1.1%	1.3%	0.8%
p Proteobacteria;c Gammaproteobacteria;o Betaproteobacteriales;f Chromobacteriaceae	0.1%	0.1%	0.0%
p Proteobacteria;c Gammaproteobacteria;o Betaproteobacteriales;f Rhodocyclaceae	0.3%	0.2%	0.5%
p Proteobacteria;c Gammaproteobacteria;o Betaproteobacteriales;f SC-I-84	0.6%	0.5%	0.6%
p Proteobacteria;c Gammaproteobacteria;o Betaproteobacteriales;f TRA3-20	0.7%	0.7%	0.7%
p Proteobacteria;c Gammaproteobacteria;o CCD24;f uncultured-bacterium	0.2%	0.3%	0.1%
p Proteobacteria;c Gammaproteobacteria;o Cellvibrionales;Other	0.0%	0.0%	0.0%
p Proteobacteria;c Gammaproteobacteria;o Cellvibrionales;f Halieaceae	0.0%	0.0%	0.0%
p Proteobacteria;c Gammaproteobacteria;o Diplorickettsiales;f Diplorickettsiaceae	0.0%	0.0%	0.0%
p Proteobacteria;c Gammaproteobacteria;o EPR3968-O8a-Bc78;f uncultured-gamma-proteobacterium	0.0%	0.0%	0.0%
p Proteobacteria;c Gammaproteobacteria;o Enterobacteriales;f Enterobacteriaceae	0.0%	0.0%	0.0%
p Proteobacteria;c Gammaproteobacteria;o Gammaproteobacteria-Incertae-Sedis;f Unknown-Family	0.0%	0.0%	0.0%
p Proteobacteria;c Gammaproteobacteria;o JG36-GS-52;f uncultured-bacterium	0.0%	0.0%	0.0%
p Proteobacteria;c Gammaproteobacteria;o JG36-TzT-191;Other	0.0%	0.0%	0.0%
p Proteobacteria;c Gammaproteobacteria;o JG36-TzT-191;f uncultured-bacterium	0.0%	0.0%	0.0%
p Proteobacteria;c Gammaproteobacteria;o KI89A-clade;Other	0.0%	0.0%	0.0%
p Proteobacteria;c Gammaproteobacteria;o PLTA13;Other	0.0%	0.0%	0.0%
p Proteobacteria;c Gammaproteobacteria;o PLTA13;f uncultured-bacterium	0.7%	1.2%	0.1%
p Proteobacteria;c Gammaproteobacteria;o PLTA13;f uncultured-proteobacterium	0.0%	0.0%	0.0%
p Proteobacteria;c Gammaproteobacteria;o Pseudomonadales;f Moraxellaceae	0.0%	0.1%	0.0%
p Proteobacteria;c Gammaproteobacteria;o Pseudomonadales;f Pseudomonadaceae	0.4%	0.7%	0.1%
p Proteobacteria;c Gammaproteobacteria;o R7C24;Other	0.0%	0.0%	0.0%
p Proteobacteria;c Gammaproteobacteria;o R7C24;f uncultured-bacterium	0.0%	0.0%	0.0%
p Proteobacteria;c Gammaproteobacteria;o Salinisphaerales;Other	0.0%	0.0%	0.0%
p Proteobacteria;c Gammaproteobacteria;o Salinisphaerales;f Solimonadaceae	0.0%	0.0%	0.0%
p Proteobacteria;c Gammaproteobacteria;o Steroidobacterales;f Steroidobacteraceae	1.4%	2.0%	0.7%
p Proteobacteria;c Gammaproteobacteria;o Xanthomonadales;f Rhodanobacteraceae	0.0%	0.0%	0.1%
p Proteobacteria;c Gammaproteobacteria;o Xanthomonadales;f Xanthomonadaceae	0.7%	1.4%	0.1%
p Rokubacteria;c NC10;o Rokubacteriales;Other	0.0%	0.0%	0.0%
p Rokubacteria;c NC10;o Rokubacteriales;f uncultured-Gram-positive-bacterium	0.1%	0.1%	0.0%
p Rokubacteria;c NC10;o Rokubacteriales;f uncultured-bacterium	1.2%	1.5%	0.8%
p Thaumarchaeota;c Group-1.1c;Other;Other	0.0%	0.0%	0.0%
p Thaumarchaeota;c Nitrososphaeria;o Nitrosopumilales;f Nitrosopumilaceae	0.0%	0.0%	0.0%
p Thaumarchaeota;c Nitrososphaeria;o Nitrososphaerales;f Nitrososphaeraceae	1.3%	1.3%	1.3%
p Thaumarchaeota;c Nitrososphaeria;o Nitrosotaleales;f Nitrosotaleaceae	0.0%	0.0%	0.0%
p Verrucomicrobia;c Verrucomicrobiae;Other;Other	0.0%	0.0%	0.0%
p Verrucomicrobia;c Verrucomicrobiae;o Chthoniobacterales;Other	0.1%	0.1%	0.1%
p Verrucomicrobia;c Verrucomicrobiae;o Chthoniobacterales;f Chthoniobacteraceae	1.9%	1.0%	2.7%
p Verrucomicrobia;c Verrucomicrobiae;o Chthoniobacterales;f Xiphinematobacteraceae	0.2%	0.3%	0.1%
p Verrucomicrobia;c Verrucomicrobiae;o Methylacidiphilales;f Methylacidiphilaceae	0.0%	0.0%	0.0%
p Verrucomicrobia;c Verrucomicrobiae;o Opitutales;f Opitutaceae	1.4%	2.5%	0.3%
p Verrucomicrobia;c Verrucomicrobiae;o Pedosphaerales;f Pedosphaeraceae	2.6%	1.6%	3.6%
p Verrucomicrobia;c Verrucomicrobiae;o Verrucomicrobiales;Other	0.0%	0.0%	0.0%
p Verrucomicrobia;c Verrucomicrobiae;o Verrucomicrobiales;f Akkermansiaceae	0.0%	0.0%	0.0%

	p Verrucomicrobia;c Verrucomicrobiae;o Verrucomicrobiales;f Rubritaleaceae	0.1%	0.1%	0.1%
	p Verrucomicrobia;c Verrucomicrobiae;o Verrucomicrobiales;f Verrucomicrobiaceae	0.0%	0.0%	0.0%
	p WPS-2;Other;Other;Other	0.0%	0.0%	0.0%
	p Zixibacteria;Other;Other;Other	0.0%	0.0%	0.0%
	p Zixibacteria;c uncultured-bacterium;o uncultured-bacterium;f uncultured-bacterium	0.0%	0.0%	0.0%

Taxonomy Summary. Current Level:

		Total	G1	G2
Legend	Taxonomy	%	%	%
	Unclassified;Other;Other;Other;Other	0.5%	0.7%	0.3%
	p Acidobacteria;Other;Other;Other;Other	0.1%	0.2%	0.1%
	p Acidobacteria;c Acidobacteriia;o Acidobacteriales;Other;Other	0.1%	0.0%	0.1%
	p Acidobacteria;c Acidobacteriia;o Acidobacteriales;f Acidobacteriaceae-(Subgroup-1);g uncultured	0.0%	0.0%	0.0%
	p Acidobacteria;c Acidobacteriia;o Acidobacteriales;f Koribacteraceae;g Candidatus-Koribacter	0.1%	0.0%	0.1%
	p Acidobacteria;c Acidobacteriia;o Acidobacteriales;f uncultured;Other	0.1%	0.1%	0.1%
	p Acidobacteria;c Acidobacteriia;o Acidobacteriales;f uncultured;g uncultured-Acidobacteria-bacterium	0.0%	0.0%	0.1%
	p Acidobacteria;c Acidobacteriia;o Acidobacteriales;f uncultured;g uncultured-Acidobacteriaceae-bacterium	0.0%	0.0%	0.0%
	p Acidobacteria;c Acidobacteriia;o Acidobacteriales;f uncultured;g uncultured-bacterium	0.0%	0.0%	0.0%
	p Acidobacteria;c Acidobacteriia;o Solibacterales;f Solibacteraceae-(Subgroup-3);Other	0.0%	0.0%	0.1%
	p Acidobacteria;c Acidobacteriia;o Solibacterales;f Solibacteraceae-(Subgroup-3);g Candidatus-Solibacter	1.8%	1.4%	2.2%
	p Acidobacteria;c Acidobacteriia;o Subgroup-12;f uncultured-bacterium;g uncultured-bacterium	0.0%	0.0%	0.0%
	p Acidobacteria;c Acidobacteriia;o Subgroup-13;f uncultured-Acidobacteria-bacterium;g uncultured-Acidobacteria-bacterium	0.0%	0.0%	0.0%
	p Acidobacteria;c Acidobacteriia;o Subgroup-2;Other;Other	0.0%	0.0%	0.0%
	p Acidobacteria;c Acidobacteriia;o Subgroup-2;f uncultured-Acidobacteria-bacterium;g uncultured-Acidobacteria-bacterium	0.0%	0.0%	0.0%
	p Acidobacteria;c Acidobacteriia;o Subgroup-2;f uncultured-bacterium;g uncultured-bacterium	0.1%	0.1%	0.0%
	p Acidobacteria;c Acidobacteriia;o Subgroup-2;f uncultured-forest-soil-bacterium;g uncultured-forest-soil-bacterium	0.0%	0.0%	0.0%
	p Acidobacteria;c Acidobacteriia;o Subgroup-2;f uncultured-soil-bacterium;g uncultured-soil-bacterium	0.0%	0.0%	0.0%
	p Acidobacteria;c Blastocatellia-(Subgroup-4);Other;Other;Other	2.6%	1.6%	3.6%
	p Acidobacteria;c Blastocatellia-(Subgroup-4);o 11-24;Other;Other	0.1%	0.0%	0.1%
	p Acidobacteria;c Blastocatellia-(Subgroup-4);o 11-24;f uncultured-Acidobacteria-bacterium;g uncultured-Acidobacteria-bacterium	0.2%	0.2%	0.2%
	p Acidobacteria;c Blastocatellia-(Subgroup-4);o 11-24;f uncultured-Acidobacteriales-bacterium;g uncultured-Acidobacteriales-bacterium	0.0%	0.0%	0.0%
	p Acidobacteria;c Blastocatellia-(Subgroup-4);o 11-24;f uncultured-bacterium;g uncultured-bacterium	0.1%	0.2%	0.1%
	p Acidobacteria;c Blastocatellia-(Subgroup-4);o Blastocatellales;f Blastocatellaceae;Other	0.2%	0.1%	0.4%
	p Acidobacteria;c Blastocatellia-(Subgroup-4);o Blastocatellales;f Blastocatellaceae;g Blastocatella	0.1%	0.1%	0.1%
	p Acidobacteria;c Blastocatellia-(Subgroup-4);o Blastocatellales;f Blastocatellaceae;g Stenotrophobacter	0.1%	0.0%	0.1%
	p Acidobacteria;c Blastocatellia-(Subgroup-4);o Blastocatellales;f Blastocatellaceae;g uncultured	0.3%	0.3%	0.3%
	p Acidobacteria;c Blastocatellia-(Subgroup-4);o DS-100;Other;Other	0.0%	0.0%	0.0%
	p Acidobacteria;c Blastocatellia-(Subgroup-4);o DS-100;f uncultured-Acidobacteria-bacterium;g uncultured-Acidobacteria-bacterium	0.0%	0.0%	0.0%
	p Acidobacteria;c Blastocatellia-(Subgroup-4);o DS-100;f uncultured-bacterium;g uncultured-bacterium	0.0%	0.0%	0.0%
	p Acidobacteria;c Blastocatellia-(Subgroup-4);o Elev-16S-573;Other;Other	5.1%	2.3%	8.0%
	p Acidobacteria;c Blastocatellia-(Subgroup-4);o Elev-16S-573;f uncultured-Acidobacteria-bacterium;g uncultured-Acidobacteria-bacterium	0.0%	0.0%	0.0%
	p Acidobacteria;c Blastocatellia-(Subgroup-4);o Elev-16S-573;f uncultured-bacterium;g uncultured-bacterium	0.7%	0.8%	0.7%
	p Acidobacteria;c Holophagae;o Holophagales;f Holophagaceae;g Geothrix	0.0%	0.0%	0.0%
	p Acidobacteria;c Holophagae;o Subgroup-7;Other;Other	0.2%	0.2%	0.2%
	p Acidobacteria;c Holophagae;o Subgroup-7;f uncultured-Acidobacteria-bacterium;g uncultured-Acidobacteria-bacterium	0.0%	0.0%	0.0%
	p Acidobacteria;c Holophagae;o Subgroup-7;f uncultured-Acidobacteriales-bacterium;g uncultured-Acidobacteriales-bacterium	0.0%	0.0%	0.0%
	p Acidobacteria;c Holophagae;o Subgroup-7;f uncultured-Acidobacterium-sp.;g uncultured-Acidobacterium-sp.	0.0%	0.0%	0.0%
	p Acidobacteria;c Holophagae;o Subgroup-7;f uncultured-bacterium;g uncultured-bacterium	0.1%	0.2%	0.1%
	p Acidobacteria;c Subgroup-11;Other;Other;Other	0.1%	0.1%	0.1%
	p Acidobacteria;c Subgroup-11;o uncultured-bacterium;f uncultured-bacterium;g uncultured-bacterium	0.0%	0.0%	0.0%
	p Acidobacteria;c Subgroup-15;Other;Other;Other	0.0%	0.0%	0.0%
	p Acidobacteria;c Subgroup-15;o uncultured-bacterium;f uncultured-bacterium;g uncultured-bacterium	0.0%	0.0%	0.0%
	p Acidobacteria;c Subgroup-17;Other;Other;Other	0.1%	0.1%	0.0%
	p Acidobacteria;c Subgroup-17;o uncultured-Acidobacteria-bacterium;f uncultured-Acidobacteria-bacterium;g uncultured-Acidobacteria-bacterium	0.0%	0.0%	0.0%
	p Acidobacteria;c Subgroup-17;o uncultured-Acidobacteriales-bacterium;f uncultured-Acidobacteriales-bacterium;g uncultured-Acidobacteriales-bacterium	0.0%	0.0%	0.0%
	p Acidobacteria;c Subgroup-17;o uncultured-bacterium;f uncultured-bacterium;g uncultured-bacterium	0.1%	0.1%	0.0%
	p Acidobacteria;c Subgroup-18;Other;Other;Other	0.0%	0.0%	0.0%
	p Acidobacteria;c Subgroup-18;o uncultured-bacterium;f uncultured-bacterium;g uncultured-bacterium	0.0%	0.0%	0.0%
	p Acidobacteria;c Subgroup-20;o uncultured-bacterium;f uncultured-bacterium;g uncultured-bacterium	0.0%	0.0%	0.0%
	p Acidobacteria;c Subgroup-22;Other;Other;Other	0.1%	0.1%	0.1%

	p Acidobacteria;c Subgroup-22;o uncultured-Acidobacterium-sp.;f uncultured-Acidobacterium-sp.;g uncultured-Acidobacterium-sp.	0.0%	0.1%	0.0%
	p Acidobacteria;c Subgroup-22;o uncultured-bacterium;f uncultured-bacterium;g uncultured-bacterium	0.1%	0.2%	0.1%
	p Acidobacteria;c Subgroup-25;Other;Other;Other	0.0%	0.1%	0.0%
	p Acidobacteria;c Subgroup-25;o uncultured-Acidobacteria-bacterium;f uncultured-Acidobacteria-bacterium;g uncultured-Acidobacteria-bacterium	0.0%	0.0%	0.0%
	p Acidobacteria;c Subgroup-25;o uncultured-bacterium;f uncultured-bacterium;g uncultured-bacterium	0.0%	0.0%	0.0%
	p Acidobacteria;c Subgroup-25;o uncultured-soil-bacterium;f uncultured-soil-bacterium;g uncultured-soil-bacterium	0.0%	0.0%	0.0%
	p Acidobacteria;c Subgroup-5;Other;Other;Other	0.0%	0.0%	0.0%
	p Acidobacteria;c Subgroup-5;o uncultured-Acidobacteria-bacterium;f uncultured-Acidobacteria-bacterium;g uncultured-Acidobacteria-bacterium	0.2%	0.2%	0.2%
	p Acidobacteria;c Subgroup-5;o uncultured-Acidobacterium-sp.;f uncultured-Acidobacterium-sp.;g uncultured-Acidobacterium-sp.	0.0%	0.0%	0.0%
	p Acidobacteria;c Subgroup-5;o uncultured-bacterium;f uncultured-bacterium;g uncultured-bacterium	0.2%	0.2%	0.2%
	p Acidobacteria;c Subgroup-6;Other;Other;Other	5.4%	6.2%	4.6%
	p Acidobacteria;c Subgroup-6;o Acidobacteria-bacterium-WX90;f Acidobacteria-bacterium-WX90;g Acidobacteria-bacterium-WX90	0.0%	0.0%	0.0%
	p Acidobacteria;c Subgroup-6;o Unknown-Order;f Unknown-Family;g Luteitalea	0.0%	0.0%	0.0%
	p Acidobacteria;c Subgroup-6;o uncultured-Acidobacteria-bacterium;f uncultured-Acidobacteria-bacterium;g uncultured-Acidobacteria-bacterium	0.2%	0.2%	0.2%
	p Acidobacteria;c Subgroup-6;o uncultured-Acidobacteriaceae-bacterium;f uncultured-Acidobacteriaceae-bacterium;g uncultured-Acidobacteriaceae-bacterium	0.0%	0.0%	0.0%
	p Acidobacteria;c Subgroup-6;o uncultured-Acidobacteriales-bacterium;f uncultured-Acidobacteriales-bacterium;g uncultured-Acidobacteriales-bacterium	0.1%	0.1%	0.1%
	p Acidobacteria;c Subgroup-6;o uncultured-Acidobacterium-sp.;f uncultured-Acidobacterium-sp.;g uncultured-Acidobacterium-sp.	0.0%	0.0%	0.0%
	p Acidobacteria;c Subgroup-6;o uncultured-Holophagae-bacterium;f uncultured-Holophagae-bacterium;g uncultured-Holophagae-bacterium	0.0%	0.0%	0.0%
	p Acidobacteria;c Subgroup-6;o uncultured-bacterium;f uncultured-bacterium;g uncultured-bacterium	0.8%	0.9%	0.8%
	p Acidobacteria;c Subgroup-6;o uncultured-beta-proteobacterium;f uncultured-beta-proteobacterium;g uncultured-beta-proteobacterium	0.0%	0.0%	0.0%
	p Acidobacteria;c Subgroup-6;o uncultured-proteobacterium;f uncultured-proteobacterium;g uncultured-proteobacterium	0.0%	0.0%	0.0%
	p Acidobacteria;c Subgroup-9;Other;Other;Other	0.0%	0.0%	0.0%
	p Actinobacteria;Other;Other;Other;Other	0.0%	0.0%	0.1%
	p Actinobacteria;c 0319-7L14;Other;Other;Other	0.0%	0.0%	0.1%
	p Actinobacteria;c 0319-7L14;o uncultured-bacterium;f uncultured-bacterium;g uncultured-bacterium	0.0%	0.0%	0.0%
	p Actinobacteria;c Acidimicrobiia;Other;Other;Other	0.3%	0.3%	0.3%
	p Actinobacteria;c Acidimicrobiia;o Acidimicrobiales;f Acidimicrobiaceae;g uncultured	0.0%	0.0%	0.0%
	p Actinobacteria;c Acidimicrobiia;o Actinomarinales;f uncultured;Other	0.1%	0.1%	0.0%
	p Actinobacteria;c Acidimicrobiia;o Actinomarinales;f uncultured;g uncultured-Actinomycetales-bacterium	0.6%	1.2%	0.1%
	p Actinobacteria;c Acidimicrobiia;o Actinomarinales;f uncultured;g uncultured-actinobacterium	0.0%	0.0%	0.0%
	p Actinobacteria;c Acidimicrobiia;o IMCC26256;Other;Other	0.4%	0.4%	0.3%
	p Actinobacteria;c Acidimicrobiia;o IMCC26256;f uncultured-Acidimicrobiales-bacterium;g uncultured-Acidimicrobiales-bacterium	0.0%	0.0%	0.0%
	p Actinobacteria;c Acidimicrobiia;o IMCC26256;f uncultured-Acidimicrobidae-bacterium;g uncultured-Acidimicrobidae-bacterium	0.0%	0.0%	0.0%
	p Actinobacteria;c Acidimicrobiia;o IMCC26256;f uncultured-actinobacterium;g uncultured-actinobacterium	0.0%	0.0%	0.1%
	p Actinobacteria;c Acidimicrobiia;o IMCC26256;f uncultured-bacterium;g uncultured-bacterium	0.0%	0.0%	0.0%
	p Actinobacteria;c Acidimicrobiia;o IMCC26256;f uncultured-organism;g uncultured-organism	0.0%	0.0%	0.0%
	p Actinobacteria;c Acidimicrobiia;o Microtrichales;Other;Other	0.1%	0.1%	0.1%
	p Actinobacteria;c Acidimicrobiia;o Microtrichales;f Iamiaceae;g Iamia	0.2%	0.2%	0.2%
	p Actinobacteria;c Acidimicrobiia;o Microtrichales;f Ilumatobacteraceae;Other	0.0%	0.0%	0.0%
	p Actinobacteria;c Acidimicrobiia;o Microtrichales;f Ilumatobacteraceae;g Ilumatobacter	0.1%	0.1%	0.1%
	p Actinobacteria;c Acidimicrobiia;o Microtrichales;f Ilumatobacteraceae;g uncultured	0.1%	0.2%	0.1%
	p Actinobacteria;c Acidimicrobiia;o Microtrichales;f Ilumatobacteraceae;g uncultured-bacterium	0.0%	0.0%	0.0%
	p Actinobacteria;c Acidimicrobiia;o Microtrichales;f Microtrichaceae;g uncultured	0.0%	0.0%	0.0%
	p Actinobacteria;c Acidimicrobiia;o Microtrichales;f uncultured;Other	0.1%	0.1%	0.1%
	p Actinobacteria;c Acidimicrobiia;o Microtrichales;f uncultured;g uncultured-bacterium	0.1%	0.1%	0.1%
	p Actinobacteria;c Acidimicrobiia;o uncultured;Other;Other	0.3%	0.2%	0.5%
	p Actinobacteria;c Acidimicrobiia;o uncultured;f uncultured-Acidimicrobidae-bacterium;g uncultured-Acidimicrobidae-bacterium	0.0%	0.0%	0.1%
	p Actinobacteria;c Acidimicrobiia;o uncultured;f uncultured-actinobacterium;g uncultured-actinobacterium	0.0%	0.0%	0.0%
	p Actinobacteria;c Acidimicrobiia;o uncultured;f uncultured-bacterium;g uncultured-bacterium	0.1%	0.0%	0.1%
	p Actinobacteria;c Acidimicrobiia;o uncultured;f uncultured-soil-bacterium;g uncultured-soil-bacterium	0.0%	0.0%	0.1%
	p Actinobacteria;c Actinobacteria;Other;Other;Other	0.0%	0.0%	0.0%
	p Actinobacteria;c Actinobacteria;o Corynebacteriales;f Mycobacteriaceae;g Mycobacterium	0.3%	0.1%	0.5%
	p Actinobacteria;c Actinobacteria;o Corynebacteriales;f Nocardiaceae;g Nocardia	0.9%	0.5%	1.4%
	p Actinobacteria;c Actinobacteria;o Corynebacteriales;f Nocardiaceae;g Rhodococcus	0.2%	0.1%	0.3%
	p Actinobacteria;c Actinobacteria;o Frankiales;f Cryptosporangiaceae;g Cryptosporangium	0.0%	0.0%	0.0%
	p Actinobacteria;c Actinobacteria;o Frankiales;f Cryptosporangiaceae;g Fodinicola	0.0%	0.0%	0.1%
	p Actinobacteria;c Actinobacteria;o Frankiales;f Frankiaceae;g Frankia	0.0%	0.0%	0.0%

	p Actinobacteria;c Actinobacteria;o Frankiales;f Geodermatophilaceae;Other	0.0%	0.0%	0.0%
	p Actinobacteria;c Actinobacteria;o Frankiales;f Geodermatophilaceae;g Blastococcus	0.0%	0.0%	0.0%
	p Actinobacteria;c Actinobacteria;o Frankiales;f Geodermatophilaceae;g Geodermatophilus	0.0%	0.0%	0.0%
	p Actinobacteria;c Actinobacteria;o Frankiales;f Nakamurellaceae;g Nakamurella	0.0%	0.0%	0.0%
	p Actinobacteria;c Actinobacteria;o Frankiales;f Sporichthyaceae;g Sporichthya	0.0%	0.0%	0.0%
	p Actinobacteria;c Actinobacteria;o Frankiales;f Sporichthyaceae;g uncultured	0.0%	0.0%	0.0%
	p Actinobacteria;c Actinobacteria;o Frankiales;f uncultured;Other	0.0%	0.0%	0.0%
	p Actinobacteria;c Actinobacteria;o Frankiales;f uncultured;g uncultured-bacterium	0.0%	0.0%	0.0%
	p Actinobacteria;c Actinobacteria;o Glycomycetales;f Glycomycetaceae;g Glycomyces	1.0%	1.9%	0.1%
	p Actinobacteria;c Actinobacteria;o Glycomycetales;f Glycomycetaceae;g uncultured	0.0%	0.0%	0.0%
	p Actinobacteria;c Actinobacteria;o Kineosporiales;f Kineosporiaceae;Other	0.0%	0.0%	0.0%
	p Actinobacteria;c Actinobacteria;o Micrococcales;Other;Other	0.0%	0.0%	0.0%
	p Actinobacteria;c Actinobacteria;o Micrococcales;f Cellulomonadaceae;g Cellulomonas	0.0%	0.0%	0.0%
	p Actinobacteria;c Actinobacteria;o Micrococcales;f Demequinaceae;Other	0.0%	0.0%	0.0%
	p Actinobacteria;c Actinobacteria;o Micrococcales;f Intrasporangiaceae;Other	0.1%	0.1%	0.1%
	p Actinobacteria;c Actinobacteria;o Micrococcales;f Microbacteriaceae;Other	0.0%	0.0%	0.0%
	p Actinobacteria;c Actinobacteria;o Micrococcales;f Microbacteriaceae;g Agromyces	0.1%	0.2%	0.1%
	p Actinobacteria;c Actinobacteria;o Micrococcales;f Microbacteriaceae;g Leifsonia	0.0%	0.0%	0.0%
	p Actinobacteria;c Actinobacteria;o Micrococcales;f Microbacteriaceae;g Microbacterium	0.0%	0.0%	0.0%
	p Actinobacteria;c Actinobacteria;o Micrococcales;f Micrococcaceae;g Arthrobacter	0.0%	0.0%	0.0%
	p Actinobacteria;c Actinobacteria;o Micrococcales;f Micrococcaceae;g Pseudarthrobacter	0.1%	0.0%	0.2%
	p Actinobacteria;c Actinobacteria;o Micrococcales;f Promicromonosporaceae;g Cellulosimicrobium	0.0%	0.0%	0.0%
	p Actinobacteria;c Actinobacteria;o Micrococcales;f Promicromonosporaceae;g Promicromonospora	0.3%	0.5%	0.0%
	p Actinobacteria;c Actinobacteria;o Micromonosporales;f Micromonosporaceae;Other	0.2%	0.2%	0.3%
	p Actinobacteria;c Actinobacteria;o Micromonosporales;f Micromonosporaceae;g Actinoplanes	0.0%	0.0%	0.0%
	p Actinobacteria;c Actinobacteria;o Micromonosporales;f Micromonosporaceae;g Dactylosporangium	0.0%	0.0%	0.0%
	p Actinobacteria;c Actinobacteria;o Micromonosporales;f Micromonosporaceae;g Krasilnikovia	0.0%	0.0%	0.0%
	p Actinobacteria;c Actinobacteria;o Micromonosporales;f Micromonosporaceae;g Luedemannella	0.0%	0.0%	0.0%
	p Actinobacteria;c Actinobacteria;o Micromonosporales;f Micromonosporaceae;g Micromonospora	0.2%	0.2%	0.1%
	p Actinobacteria;c Actinobacteria;o Micromonosporales;f Micromonosporaceae;g Phytomonospora	0.0%	0.0%	0.0%
	p Actinobacteria;c Actinobacteria;o Micromonosporales;f Micromonosporaceae;g Stackebrandtia	0.0%	0.0%	0.0%
	p Actinobacteria;c Actinobacteria;o Micromonosporales;f Micromonosporaceae;g uncultured	0.1%	0.0%	0.1%
	p Actinobacteria;c Actinobacteria;o Propionibacteriales;f Nocardioidaceae;Other	0.3%	0.2%	0.3%
	p Actinobacteria;c Actinobacteria;o Propionibacteriales;f Nocardioidaceae;g Actinopolymorpha	0.0%	0.0%	0.0%
	p Actinobacteria;c Actinobacteria;o Propionibacteriales;f Nocardioidaceae;g Aeromicrobium	0.0%	0.1%	0.0%
	p Actinobacteria;c Actinobacteria;o Propionibacteriales;f Nocardioidaceae;g Kribbella	0.7%	0.9%	0.5%
	p Actinobacteria;c Actinobacteria;o Propionibacteriales;f Nocardioidaceae;g Marmoricola	0.0%	0.0%	0.0%
	p Actinobacteria;c Actinobacteria;o Propionibacteriales;f Nocardioidaceae;g Nocardioides	0.5%	0.4%	0.7%
	p Actinobacteria;c Actinobacteria;o Propionibacteriales;f Propionibacteriaceae;g Microlunatus	0.0%	0.0%	0.0%
	p Actinobacteria;c Actinobacteria;o Propionibacteriales;f Propionibacteriaceae;g uncultured	0.0%	0.0%	0.0%
	p Actinobacteria;c Actinobacteria;o Pseudonocardiales;f Pseudonocardiaceae;Other	0.1%	0.0%	0.1%
	p Actinobacteria;c Actinobacteria;o Pseudonocardiales;f Pseudonocardiaceae;g Actinophytocola	0.0%	0.0%	0.1%
	p Actinobacteria;c Actinobacteria;o Pseudonocardiales;f Pseudonocardiaceae;g Allokutzneria	0.0%	0.0%	0.0%
	p Actinobacteria;c Actinobacteria;o Pseudonocardiales;f Pseudonocardiaceae;g Amycolatopsis	0.0%	0.0%	0.0%
	p Actinobacteria;c Actinobacteria;o Pseudonocardiales;f Pseudonocardiaceae;g Crossiella	0.0%	0.0%	0.1%
	p Actinobacteria;c Actinobacteria;o Pseudonocardiales;f Pseudonocardiaceae;g Pseudonocardia	0.1%	0.1%	0.1%
	p Actinobacteria;c Actinobacteria;o Streptomycetales;f Streptomycetaceae;Other	0.0%	0.0%	0.0%
	p Actinobacteria;c Actinobacteria;o Streptomycetales;f Streptomycetaceae;g Streptomyces	7.6%	8.0%	7.3%
	p Actinobacteria;c Actinobacteria;o Streptosporangiales;f Streptosporangiaceae;Other	0.0%	0.0%	0.0%
	p Actinobacteria;c Actinobacteria;o Streptosporangiales;f Streptosporangiaceae;g Nonomuraea	0.1%	0.2%	0.0%
	p Actinobacteria;c Actinobacteria;o Streptosporangiales;f Streptosporangiaceae;g uncultured	0.0%	0.0%	0.0%
	p Actinobacteria;c Actinobacteria;o Streptosporangiales;f Thermomonosporaceae;Other	0.0%	0.0%	0.0%
	p Actinobacteria;c MB-A2-108;Other;Other;Other	0.1%	0.1%	0.1%
	p Actinobacteria;c MB-A2-108;o uncultured-actinobacterium;f uncultured-actinobacterium;g uncultured-actinobacterium	0.0%	0.0%	0.0%
	p Actinobacteria;c MB-A2-108;o uncultured-bacterium;f uncultured-bacterium;g uncultured-bacterium	0.6%	0.7%	0.4%
	p Actinobacteria;c MB-A2-108;o uncultured-bacterium-contig00016;f uncultured-bacterium-contig00016;g uncultured-bacterium-contig00016	0.0%	0.0%	0.0%
	p Actinobacteria;c Nitriliruptoria;o Euzebyales;f Euzebyaceae;g uncultured	0.0%	0.0%	0.0%
	p Actinobacteria;c Rubrobacteria;o Rubrobacterales;f Rubrobacteriaceae;g Rubrobacter	0.1%	0.0%	0.1%
	p Actinobacteria;c Thermoleophilia;Other;Other;Other	0.1%	0.1%	0.1%
	p Actinobacteria;c Thermoleophilia;o Gaiellales;Other;Other	0.0%	0.0%	0.0%
	p Actinobacteria;c Thermoleophilia;o Gaiellales;f uncultured;Other	0.2%	0.2%	0.1%
	p Actinobacteria;c Thermoleophilia;o Gaiellales;f uncultured;g uncultured-Gaiella-sp.	0.1%	0.1%	0.1%
	p Actinobacteria;c Thermoleophilia;o Gaiellales;f uncultured;g uncultured-Rubrobacteria-bacterium	0.0%	0.0%	0.0%
	p Actinobacteria;c Thermoleophilia;o Gaiellales;f uncultured;g uncultured-actinobacterium	0.0%	0.0%	0.0%
	p Actinobacteria;c Thermoleophilia;o Gaiellales;f uncultured;g uncultured-bacterium	0.4%	0.4%	0.3%
	p Actinobacteria;c Thermoleophilia;o Solirubrobacterales;f 67-14;Other	0.0%	0.0%	0.0%
	p Actinobacteria;c Thermoleophilia;o Solirubrobacterales;f 67-14;g uncultured-actinobacterium	0.0%	0.0%	0.0%
	p Actinobacteria;c Thermoleophilia;o Solirubrobacterales;f 67-14;g uncultured-bacterium	0.3%	0.3%	0.2%

	p Actinobacteria;c Thermoleophilia;o Solirubrobacterales;f Solirubrobacteraceae;g Conexibacter	0.0%	0.0%	0.0%
	p Actinobacteria;c Thermoleophilia;o Solirubrobacterales;f Solirubrobacteraceae;g Solirubrobacter	0.2%	0.2%	0.2%
	p Actinobacteria;c Thermoleophilia;o uncultured;f uncultured-bacterium;g uncultured-bacterium	0.0%	0.0%	0.0%
	p Armatimonadetes;c Fimbriimonadia;o Fimbriimonadales;f Fimbriimonadaceae;Other	0.0%	0.0%	0.0%
	p Armatimonadetes;c Fimbriimonadia;o Fimbriimonadales;f Fimbriimonadaceae;g uncultured-bacterium	0.0%	0.0%	0.0%
	p BRC1;Other;Other;Other;Other	0.0%	0.0%	0.0%
	p BRC1;c uncultured-bacterium;o uncultured-bacterium;f uncultured-bacterium;g uncultured-bacterium	0.0%	0.0%	0.0%
	p Bacteroidetes;c Bacteroidia;Other;Other;Other	0.0%	0.0%	0.0%
	p Bacteroidetes;c Bacteroidia;o Bacteroidetes-VC2.1-Bac22;Other;Other	0.0%	0.0%	0.0%
	p Bacteroidetes;c Bacteroidia;o Chitinophagales;Other;Other	0.0%	0.0%	0.0%
	p Bacteroidetes;c Bacteroidia;o Chitinophagales;f 37-13;Other	0.0%	0.0%	0.0%
	p Bacteroidetes;c Bacteroidia;o Chitinophagales;f 37-13;g uncultured-bacterium	0.0%	0.1%	0.0%
	p Bacteroidetes;c Bacteroidia;o Chitinophagales;f Chitinophagaceae;Other	0.4%	0.4%	0.5%
	p Bacteroidetes;c Bacteroidia;o Chitinophagales;f Chitinophagaceae;g Chitinophaga	2.4%	4.8%	0.0%
	p Bacteroidetes;c Bacteroidia;o Chitinophagales;f Chitinophagaceae;g Dinghuibacter	0.0%	0.0%	0.0%
	p Bacteroidetes;c Bacteroidia;o Chitinophagales;f Chitinophagaceae;g Ferruginibacter	0.0%	0.0%	0.0%
	p Bacteroidetes;c Bacteroidia;o Chitinophagales;f Chitinophagaceae;g Filimonas	0.0%	0.0%	0.0%
	p Bacteroidetes;c Bacteroidia;o Chitinophagales;f Chitinophagaceae;g Flavihumibacter	0.3%	0.5%	0.0%
	p Bacteroidetes;c Bacteroidia;o Chitinophagales;f Chitinophagaceae;g Flavisolibacter	0.4%	0.2%	0.6%
	p Bacteroidetes;c Bacteroidia;o Chitinophagales;f Chitinophagaceae;g Flavitalea	0.2%	0.3%	0.1%
	p Bacteroidetes;c Bacteroidia;o Chitinophagales;f Chitinophagaceae;g Niabella	0.0%	0.0%	0.0%
	p Bacteroidetes;c Bacteroidia;o Chitinophagales;f Chitinophagaceae;g Niastella	0.8%	1.2%	0.4%
	p Bacteroidetes;c Bacteroidia;o Chitinophagales;f Chitinophagaceae;g Niveitalea	0.0%	0.1%	0.0%
	p Bacteroidetes;c Bacteroidia;o Chitinophagales;f Chitinophagaceae;g Parafilimonas	0.1%	0.1%	0.2%
	p Bacteroidetes;c Bacteroidia;o Chitinophagales;f Chitinophagaceae;g Terrimonas	1.4%	2.3%	0.5%
	p Bacteroidetes;c Bacteroidia;o Chitinophagales;f Chitinophagaceae;g uncultured	1.9%	1.2%	2.6%
	p Bacteroidetes;c Bacteroidia;o Chitinophagales;f Saprospiraceae;g uncultured	0.3%	0.2%	0.3%
	p Bacteroidetes;c Bacteroidia;o Chitinophagales;f uncultured;g uncultured-bacterium	0.0%	0.0%	0.0%
	p Bacteroidetes;c Bacteroidia;o Cytophagales;f Cytophagaceae;g Sporocytophaga	0.0%	0.0%	0.0%
	p Bacteroidetes;c Bacteroidia;o Cytophagales;f Hymenobacteraceae;g Adhaeribacter	0.1%	0.0%	0.2%
	p Bacteroidetes;c Bacteroidia;o Cytophagales;f Microscillaceae;Other	0.3%	0.1%	0.4%
	p Bacteroidetes;c Bacteroidia;o Cytophagales;f Microscillaceae;g Ohtaekwangia	1.0%	1.3%	0.7%
	p Bacteroidetes;c Bacteroidia;o Cytophagales;f Microscillaceae;g uncultured	0.7%	0.7%	0.8%
	p Bacteroidetes;c Bacteroidia;o Cytophagales;f Spirosomaceae;g Dyadobacter	0.0%	0.0%	0.0%
	p Bacteroidetes;c Bacteroidia;o Flavobacteriales;f Crocinitomicaceae;Other	0.0%	0.0%	0.0%
	p Bacteroidetes;c Bacteroidia;o Flavobacteriales;f Crocinitomicaceae;g Fluviicola	0.0%	0.0%	0.0%
	p Bacteroidetes;c Bacteroidia;o Flavobacteriales;f Flavobacteriaceae;g Flavobacterium	0.3%	0.1%	0.5%
	p Bacteroidetes;c Bacteroidia;o Flavobacteriales;f NS9-marine-group;Other	0.0%	0.0%	0.0%
	p Bacteroidetes;c Bacteroidia;o Flavobacteriales;f NS9-marine-group;g uncultured-bacterium	0.0%	0.0%	0.0%
	p Bacteroidetes;c Bacteroidia;o Sphingobacteriales;Other;Other	0.0%	0.0%	0.0%
	p Bacteroidetes;c Bacteroidia;o Sphingobacteriales;f AKYH767;Other	0.1%	0.1%	0.1%
	p Bacteroidetes;c Bacteroidia;o Sphingobacteriales;f AKYH767;g uncultured-bacterium	0.2%	0.2%	0.2%
	p Bacteroidetes;c Bacteroidia;o Sphingobacteriales;f NS11-12-marine-group;g uncultured-bacterium	0.0%	0.0%	0.0%
	p Bacteroidetes;c Bacteroidia;o Sphingobacteriales;f Sphingobacteriaceae;Other	0.0%	0.0%	0.0%
	p Bacteroidetes;c Bacteroidia;o Sphingobacteriales;f Sphingobacteriaceae;g Olivibacter	0.0%	0.0%	0.0%
	p Bacteroidetes;c Bacteroidia;o Sphingobacteriales;f Sphingobacteriaceae;g Pedobacter	0.0%	0.0%	0.0%
	p Bacteroidetes;c Bacteroidia;o Sphingobacteriales;f Sphingobacteriaceae;g Solitalea	0.1%	0.2%	0.1%
	p Bacteroidetes;c Bacteroidia;o Sphingobacteriales;f Sphingobacteriaceae;g Sphingobacterium	0.2%	0.3%	0.0%
	p Bacteroidetes;c Bacteroidia;o Sphingobacteriales;f Sphingobacteriaceae;g uncultured	0.0%	0.0%	0.0%
	p Bacteroidetes;c Bacteroidia;o Sphingobacteriales;f env.OPS-17;Other	0.1%	0.1%	0.1%
	p Bacteroidetes;c Bacteroidia;o Sphingobacteriales;f env.OPS-17;g uncultured-bacterium	0.2%	0.1%	0.3%
	p Bacteroidetes;c Ignavibacteria;o Ignavibacteriales;f uncultured-bacterium;g uncultured-bacterium	0.0%	0.0%	0.0%
	p Bacteroidetes;c Ignavibacteria;o Kryptoniales;f BSV26;Other	0.1%	0.1%	0.0%
	p Bacteroidetes;c Ignavibacteria;o Kryptoniales;f BSV26;g uncultured-Chlorobi-bacterium	0.0%	0.0%	0.0%
	p Bacteroidetes;c Ignavibacteria;o OPB56;Other;Other	0.0%	0.0%	0.0%
	p Bacteroidetes;c Ignavibacteria;o OPB56;f uncultured-bacterium;g uncultured-bacterium	0.0%	0.0%	0.0%
	p Bacteroidetes;c Ignavibacteria;o OPB56;f uncultured-bacterium-#0319-6E22;g uncultured-bacterium-#0319-6E22	0.0%	0.0%	0.0%
	p Bacteroidetes;c Ignavibacteria;o OPB56;f uncultured-bacterium-KF-JG30-B11;g uncultured-bacterium-KF-JG30-B11	0.0%	0.0%	0.0%
	p Bacteroidetes;c Ignavibacteria;o SJA-28;f uncultured-bacterium;g uncultured-bacterium	0.0%	0.1%	0.0%
	p Chloroflexi;Other;Other;Other;Other	0.0%	0.0%	0.0%
	p Chloroflexi;c AD3;o uncultured-bacterium;f uncultured-bacterium;g uncultured-bacterium	0.0%	0.0%	0.0%
	p Chloroflexi;c Anaerolineae;Other;Other;Other	0.0%	0.0%	0.0%
	p Chloroflexi;c Anaerolineae;o Anaerolineales;f Anaerolineaceae;Other	0.3%	0.3%	0.4%
	p Chloroflexi;c Anaerolineae;o Anaerolineales;f Anaerolineaceae;g Anaerolinea	0.0%	0.0%	0.0%
	p Chloroflexi;c Anaerolineae;o Anaerolineales;f Anaerolineaceae;g Longilinea	0.4%	0.2%	0.7%
	p Chloroflexi;c Anaerolineae;o Anaerolineales;f Anaerolineaceae;g uncultured	0.4%	0.4%	0.4%
	p Chloroflexi;c Anaerolineae;o Ardenticatenales;f uncultured;Other	0.1%	0.0%	0.1%
	p Chloroflexi;c Anaerolineae;o Ardenticatenales;f uncultured;g uncultured-Litorilinea-sp.	0.1%	0.1%	0.2%
	p Chloroflexi;c Anaerolineae;o Ardenticatenales;f uncultured;g uncultured-prokaryote	0.0%	0.0%	0.0%

	p Chloroflexi;c Anaerolineae;o Caldilineales;f Caldilineaceae;g Litorilinea	0.0%	0.0%	0.0%
	p Chloroflexi;c Anaerolineae;o Caldilineales;f Caldilineaceae;g uncultured	0.0%	0.0%	0.0%
	p Chloroflexi;c Anaerolineae;o RBG-13-54-9;Other;Other	0.0%	0.0%	0.1%
	p Chloroflexi;c Anaerolineae;o RBG-13-54-9;f uncultured-Caldilineaceae-bacterium;g uncultured-Caldilineaceae-bacterium	0.0%	0.0%	0.0%
	p Chloroflexi;c Anaerolineae;o RBG-13-54-9;f uncultured-sludge-bacterium-A31;g uncultured-sludge-bacterium-A31	0.0%	0.0%	0.0%
	p Chloroflexi;c Anaerolineae;o SBR1031;Other;Other	0.1%	0.2%	0.0%
	p Chloroflexi;c Anaerolineae;o SBR1031;f A4b;Other	0.0%	0.0%	0.1%
	p Chloroflexi;c Anaerolineae;o SBR1031;f A4b;g uncultured-Caldilineales-bacterium	0.1%	0.0%	0.1%
	p Chloroflexi;c Anaerolineae;o SBR1031;f A4b;g uncultured-bacterium	0.1%	0.1%	0.2%
	p Chloroflexi;c Anaerolineae;o SBR1031;f A4b;g uncultured-sludge-bacterium-H8	0.0%	0.0%	0.0%
	p Chloroflexi;c Anaerolineae;o SBR1031;f A4b;g uncultured-sludge-bacterium-S14	0.0%	0.0%	0.0%
	p Chloroflexi;c Anaerolineae;o SBR1031;f A4b;g uncultured-soil-bacterium	0.0%	0.0%	0.0%
	p Chloroflexi;c Anaerolineae;o SBR1031;f uncultured-Caldilineaceae-bacterium;g uncultured-Caldilineaceae-bacterium	0.0%	0.0%	0.0%
	p Chloroflexi;c Anaerolineae;o SBR1031;f uncultured-bacterium;g uncultured-bacterium	0.0%	0.0%	0.0%
	p Chloroflexi;c Anaerolineae;o SBR1031;f uncultured-soil-bacterium;g uncultured-soil-bacterium	0.0%	0.0%	0.0%
	p Chloroflexi;c Anaerolineae;o uncultured-Bellilinea-sp.;f uncultured-Bellilinea-sp.;g uncultured-Bellilinea-sp.	0.0%	0.0%	0.0%
	p Chloroflexi;c Chloroflexia;o Chloroflexales;Other;Other	0.0%	0.0%	0.0%
	p Chloroflexi;c Chloroflexia;o Chloroflexales;f Chloroflexaceae;Other	0.0%	0.0%	0.0%
	p Chloroflexi;c Chloroflexia;o Chloroflexales;f Chloroflexaceae;g Candidatus-Chloroploca	0.0%	0.0%	0.0%
	p Chloroflexi;c Chloroflexia;o Chloroflexales;f Herpetosiphonaceae;g Herpetosiphon	0.0%	0.0%	0.0%
	p Chloroflexi;c Chloroflexia;o Chloroflexales;f Roseiflexaceae;g Roseiflexus	0.0%	0.0%	0.0%
	p Chloroflexi;c Chloroflexia;o Chloroflexales;f Roseiflexaceae;g uncultured	1.2%	0.4%	2.0%
	p Chloroflexi;c Chloroflexia;o Kallotenuales;f AKIW781;Other	0.0%	0.0%	0.0%
	p Chloroflexi;c Chloroflexia;o Kallotenuales;f AKIW781;g uncultured-bacterium	0.0%	0.0%	0.0%
	p Chloroflexi;c Chloroflexia;o Thermomicrobiales;f AKYG1722;Other	0.0%	0.1%	0.0%
	p Chloroflexi;c Chloroflexia;o Thermomicrobiales;f AKYG1722;g uncultured-Chloroflexi-bacterium	0.1%	0.1%	0.1%
	p Chloroflexi;c Chloroflexia;o Thermomicrobiales;f AKYG1722;g uncultured-bacterium	0.0%	0.0%	0.0%
	p Chloroflexi;c Chloroflexia;o Thermomicrobiales;f AKYG1722;g uncultured-soil-bacterium	0.0%	0.0%	0.0%
	p Chloroflexi;c Chloroflexia;o Thermomicrobiales;f JG30-KF-CM45;Other	0.2%	0.2%	0.3%
	p Chloroflexi;c Chloroflexia;o Thermomicrobiales;f JG30-KF-CM45;g uncultured-Chloroflexi-bacterium	0.0%	0.0%	0.0%
	p Chloroflexi;c Chloroflexia;o Thermomicrobiales;f JG30-KF-CM45;g uncultured-bacterium	0.1%	0.1%	0.1%
	p Chloroflexi;c Chloroflexia;o Thermomicrobiales;f JG30-KF-CM45;g uncultured-soil-bacterium	0.0%	0.0%	0.0%
	p Chloroflexi;c Chloroflexia;o Thermomicrobiales;f Thermomicrobiaceae;g Nitrolancea	0.0%	0.0%	0.0%
	p Chloroflexi;c Dehalococcoidia;o S085;Other;Other	0.2%	0.3%	0.1%
	p Chloroflexi;c Dehalococcoidia;o S085;f uncultured-Chloroflexi-bacterium;g uncultured-Chloroflexi-bacterium	0.0%	0.0%	0.0%
	p Chloroflexi;c Dehalococcoidia;o S085;f uncultured-bacterium;g uncultured-bacterium	0.1%	0.2%	0.1%
	p Chloroflexi;c Dehalococcoidia;o S085;f uncultured-soil-bacterium;g uncultured-soil-bacterium	0.0%	0.0%	0.0%
	p Chloroflexi;c Gitt-GS-136;Other;Other;Other	0.0%	0.0%	0.0%
	p Chloroflexi;c Gitt-GS-136;o uncultured-bacterium;f uncultured-bacterium;g uncultured-bacterium	0.3%	0.4%	0.1%
	p Chloroflexi;c JG30-KF-CM66;Other;Other;Other	0.0%	0.0%	0.0%
	p Chloroflexi;c JG30-KF-CM66;o uncultured-Chloroflexi-bacterium;f uncultured-Chloroflexi-bacterium;g uncultured-Chloroflexi-bacterium	0.0%	0.0%	0.0%
	p Chloroflexi;c JG30-KF-CM66;o uncultured-bacterium;f uncultured-bacterium;g uncultured-bacterium	0.1%	0.1%	0.1%
	p Chloroflexi;c KD4-96;Other;Other;Other	0.2%	0.3%	0.1%
	p Chloroflexi;c KD4-96;o uncultured-Chloroflexi-bacterium;f uncultured-Chloroflexi-bacterium;g uncultured-Chloroflexi-bacterium	0.1%	0.1%	0.1%
	p Chloroflexi;c KD4-96;o uncultured-bacterium;f uncultured-bacterium;g uncultured-bacterium	0.4%	0.5%	0.3%
	p Chloroflexi;c Ktedonobacteria;o C0119;Other;Other	0.0%	0.0%	0.0%
	p Chloroflexi;c Ktedonobacteria;o C0119;f uncultured-soil-bacterium;g uncultured-soil-bacterium	0.0%	0.0%	0.0%
	p Chloroflexi;c Ktedonobacteria;o Ktedonobacterales;f Ktedonobacteraceae;Other	0.0%	0.0%	0.0%
	p Chloroflexi;c Ktedonobacteria;o Ktedonobacterales;f Ktedonobacteraceae;g Thermosporothrix	0.0%	0.0%	0.0%
	p Chloroflexi;c Ktedonobacteria;o Ktedonobacterales;f Ktedonobacteraceae;g uncultured	0.0%	0.0%	0.0%
	p Chloroflexi;c OLB14;Other;Other;Other	0.0%	0.0%	0.0%
	p Chloroflexi;c OLB14;o uncultured-bacterium;f uncultured-bacterium;g uncultured-bacterium	0.1%	0.1%	0.1%
	p Chloroflexi;c OLB14;o uncultured-gamma-proteobacterium;f uncultured-gamma-proteobacterium;g uncultured-gamma-proteobacterium	0.0%	0.0%	0.0%
	p Chloroflexi;c P2-11E;o uncultured-bacterium;f uncultured-bacterium;g uncultured-bacterium	0.2%	0.1%	0.2%
	p Chloroflexi;c TK10;Other;Other;Other	0.2%	0.2%	0.2%
	p Chloroflexi;c TK10;o uncultured-Chloroflexi-bacterium;f uncultured-Chloroflexi-bacterium;g uncultured-Chloroflexi-bacterium	0.0%	0.0%	0.1%
	p Chloroflexi;c TK10;o uncultured-bacterium;f uncultured-bacterium;g uncultured-bacterium	0.0%	0.1%	0.0%
	p Dadabacteria;c Dadabacteriia;o Dadabacteriales;f uncultured-soil-bacterium;g uncultured-soil-bacterium	0.0%	0.0%	0.0%
	p Dependentiae;c Babeliae;o Babeliales;Other;Other	0.0%	0.0%	0.0%
	p Dependentiae;c Babeliae;o Babeliales;f Babeliaceae;g uncultured-bacterium	0.0%	0.0%	0.0%
	p Dependentiae;c Babeliae;o Babeliales;f Vermiphilaceae;Other	0.0%	0.0%	0.0%
	p Dependentiae;c Babeliae;o Babeliales;f Vermiphilaceae;g uncultured-bacterium	0.0%	0.0%	0.0%
	p Elusimicrobia;c Elusimicrobia;o MVP-88;Other;Other	0.0%	0.0%	0.0%
	p Elusimicrobia;c Lineage-IIa;Other;Other;Other	0.0%	0.0%	0.0%

	p Elusimicrobia;c Lineage-IIa;o uncultured-bacterium;f uncultured-bacterium;g uncultured-bacterium	0.0%	0.1%	0.0%
	p Elusimicrobia;c Lineage-IIb;o uncultured-bacterium;f uncultured-bacterium;g uncultured-bacterium	0.0%	0.0%	0.0%
	p Elusimicrobia;c Lineage-IIb;o uncultured-soil-bacterium;f uncultured-soil-bacterium;g uncultured-soil-bacterium	0.0%	0.0%	0.0%
	p Entotheonellaeota;c Entotheonellia;o Entotheonellales;f Entotheonellaceae;Other	0.0%	0.0%	0.0%
	p Entotheonellaeota;c Entotheonellia;o Entotheonellales;f Entotheonellaceae;g Candidatus-Entotheonella	0.0%	0.0%	0.0%
	p Entotheonellaeota;c Entotheonellia;o Entotheonellales;f Entotheonellaceae;g uncultured-bacterium	0.0%	0.1%	0.0%
	p Euryarchaeota;c Methanomicrobia;o Methanosarcinales;f Methanosarcinaceae;g Methanosarcina	0.0%	0.0%	0.0%
	p Euryarchaeota;c Thermoplasmata;Other;Other;Other	0.1%	0.1%	0.1%
	p Euryarchaeota;c Thermoplasmata;o Marine-Group-II;Other;Other	0.2%	0.1%	0.2%
	p Euryarchaeota;c Thermoplasmata;o Marine-Group-II;f uncultured-archaeon;g uncultured-archaeon	1.1%	0.6%	1.6%
	p Euryarchaeota;c Thermoplasmata;o Marine-Group-II;f uncultured-haloarchaeon;g uncultured-haloarchaeon	0.0%	0.0%	0.0%
	p Euryarchaeota;c Thermoplasmata;o Methanomassiliicoccales;f uncultured;g uncultured-archaeon	0.5%	0.0%	0.9%
	p Euryarchaeota;c Thermoplasmata;o uncultured;Other;Other	0.0%	0.0%	0.0%
	p Firmicutes;c Bacilli;o Bacillales;f Bacillaceae;Other	0.0%	0.0%	0.0%
	p Firmicutes;c Bacilli;o Bacillales;f Bacillaceae;g Bacillus	2.6%	0.8%	4.5%
	p Firmicutes;c Bacilli;o Bacillales;f Bacillaceae;g Fictibacillus	0.0%	0.0%	0.0%
	p Firmicutes;c Bacilli;o Bacillales;f Paenibacillaceae;g Brevibacillus	0.0%	0.0%	0.0%
	p Firmicutes;c Bacilli;o Bacillales;f Paenibacillaceae;g Oxalophagus	0.1%	0.0%	0.2%
	p Firmicutes;c Bacilli;o Bacillales;f Paenibacillaceae;g Paenibacillus	0.1%	0.1%	0.1%
	p Firmicutes;c Bacilli;o Bacillales;f Paenibacillaceae;g Thermobacillus	0.0%	0.0%	0.0%
	p Firmicutes;c Bacilli;o Bacillales;f Planococcaceae;g Domibacillus	0.0%	0.0%	0.0%
	p Firmicutes;c Bacilli;o Bacillales;f Planococcaceae;g Lysinibacillus	0.0%	0.0%	0.0%
	p Firmicutes;c Bacilli;o Bacillales;f Planococcaceae;g Sporosarcina	0.0%	0.0%	0.0%
	p Firmicutes;c Bacilli;o Bacillales;f Thermoactinomycetaceae;g Shimazuella	0.0%	0.0%	0.0%
	p Firmicutes;c Clostridia;o Clostridiales;f Clostridiaceae-1;g Clostridium-sensu-stricto-1	0.0%	0.0%	0.0%
	p Firmicutes;c Clostridia;o Clostridiales;f Heliobacteriaceae;g Hydrogenispora	0.0%	0.0%	0.0%
	p Firmicutes;c Clostridia;o Clostridiales;f Peptostreptococcaceae;g Sporacetigenium	0.0%	0.0%	0.0%
	p Firmicutes;c Clostridia;o Clostridiales;f Ruminococcaceae;g Ruminococcus-2	0.0%	0.0%	0.0%
	p GAL15;c uncultured-bacterium;o uncultured-bacterium;f uncultured-bacterium;g uncultured-bacterium	0.0%	0.0%	0.0%
	p Gemmatimonadetes;c AKAU4049;Other;Other;Other	0.2%	0.3%	0.0%
	p Gemmatimonadetes;c BD2-11-terrestrial-group;Other;Other;Other	0.0%	0.0%	0.0%
	p Gemmatimonadetes;c BD2-11-terrestrial-group;o uncultured-Gemmatimonadales-bacterium;f uncultured-Gemmatimonadales-bacterium;g uncultured-Gemmatimonadales-bacterium	0.0%	0.0%	0.0%
	p Gemmatimonadetes;c BD2-11-terrestrial-group;o uncultured-Gemmatimonadetes-bacterium;f uncultured-Gemmatimonadetes-bacterium;g uncultured-Gemmatimonadetes-bacterium	0.0%	0.1%	0.0%
	p Gemmatimonadetes;c BD2-11-terrestrial-group;o uncultured-bacterium;f uncultured-bacterium;g uncultured-bacterium	0.0%	0.0%	0.0%
	p Gemmatimonadetes;c BD2-11-terrestrial-group;o uncultured-soil-bacterium;f uncultured-soil-bacterium;g uncultured-soil-bacterium	0.0%	0.0%	0.0%
	p Gemmatimonadetes;c Gemmatimonadetes;o Gemmatimonadales;f Gemmatimonadaceae;Other	0.2%	0.2%	0.2%
	p Gemmatimonadetes;c Gemmatimonadetes;o Gemmatimonadales;f Gemmatimonadaceae;g Gemmatimonas	0.1%	0.1%	0.1%
	p Gemmatimonadetes;c Gemmatimonadetes;o Gemmatimonadales;f Gemmatimonadaceae;g Gemmatirosa	0.1%	0.0%	0.1%
	p Gemmatimonadetes;c Gemmatimonadetes;o Gemmatimonadales;f Gemmatimonadaceae;g uncultured	2.2%	2.2%	2.3%
	p Gemmatimonadetes;c Longimicrobia;o Longimicrobiales;f Longimicrobiaceae;g uncultured-bacterium	0.0%	0.0%	0.0%
	p Gemmatimonadetes;c S0134-terrestrial-group;Other;Other;Other	0.1%	0.1%	0.1%
	p Gemmatimonadetes;c S0134-terrestrial-group;o uncultured-Gemmatimonadales-bacterium;f uncultured-Gemmatimonadales-bacterium;g uncultured-Gemmatimonadales-bacterium	0.2%	0.2%	0.2%
	p Gemmatimonadetes;c S0134-terrestrial-group;o uncultured-Gemmatimonadetes-bacterium;f uncultured-Gemmatimonadetes-bacterium;g uncultured-Gemmatimonadetes-bacterium	0.0%	0.0%	0.0%
	p Gemmatimonadetes;c S0134-terrestrial-group;o uncultured-bacterium;f uncultured-bacterium;g uncultured-bacterium	0.2%	0.2%	0.2%
	p Hydrogenedentes;c Hydrogenedentia;o Hydrogenedentiales;f Hydrogenedensaceae;g uncultured-bacterium	0.0%	0.0%	0.0%
	p Hydrogenedentes;c Hydrogenedentia;o Hydrogenedentiales;f Hydrogenedensaceae;g uncultured-bacterium-SJP-3	0.0%	0.0%	0.0%
	p Latescibacteria;Other;Other;Other;Other	0.3%	0.4%	0.1%
	p Latescibacteria;c Latescibacteria;o Latescibacterales;f Latescibacteraceae;Other	0.0%	0.0%	0.0%
	p Latescibacteria;c Latescibacteria;o Latescibacterales;f Latescibacteraceae;g uncultured-bacterium	0.1%	0.2%	0.0%
	p Latescibacteria;c uncultured-Acidobacterium-sp.;o uncultured-Acidobacterium-sp.;f uncultured-Acidobacterium-sp.;g uncultured-Acidobacterium-sp.	0.0%	0.0%	0.0%
	p Latescibacteria;c uncultured-Fibrobacteres-bacterium;o uncultured-Fibrobacteres-bacterium;f uncultured-Fibrobacteres-bacterium;g uncultured-Fibrobacteres-bacterium	0.0%	0.0%	0.0%
	p Latescibacteria;c uncultured-Pelobacter-sp.;o uncultured-Pelobacter-sp.;f uncultured-Pelobacter-sp.;g uncultured-Pelobacter-sp.	0.0%	0.0%	0.0%
	p Latescibacteria;c uncultured-bacterium;o uncultured-bacterium;f uncultured-bacterium;g uncultured-bacterium	0.4%	0.5%	0.3%
	p Latescibacteria;c uncultured-prokaryote;o uncultured-prokaryote;f uncultured-prokaryote;g uncultured-prokaryote	0.0%	0.0%	0.0%
	p Latescibacteria;c uncultured-proteobacterium;o uncultured-proteobacterium;f uncultured-proteobacterium;g uncultured-proteobacterium	0.0%	0.0%	0.0%
	p Latescibacteria;c uncultured-soil-bacterium;o uncultured-soil-bacterium;f uncultured-soil-bacterium;g uncultured-soil-bacterium	0.0%	0.1%	0.0%
	p Nanoarchaeaeota;c Nanohaloarchaeia;o Aenigmarchaeales;Other;Other	0.0%	0.0%	0.0%
	p Nanoarchaeaeota;c Nanohaloarchaeia;o Aenigmarchaeales;f uncultured-archaeon;g uncultured-archaeon	0.0%	0.1%	0.0%
	p Nanoarchaeaeota;c Nanohaloarchaeia;o Deep-Sea-Euryarchaeotic-Group(DSEG);Other;Other	0.0%	0.0%	0.0%
	p Nanoarchaeaeota;c Woesearchaeia;Other;Other;Other	0.1%	0.2%	0.1%

	p Nanoarchaeaeota;c Woesearchaeia;o Candidatus-Amesbacteria-bacterium-GW2011_GWC1_47_15;f Candidatus-Amesbacteria-bacterium-GW2011_GWC1_47_15;g Candidatus-Amesbacteria-bacterium-GW2011_GWC1_47_15	0.0%	0.0%	0.0%
	p Nanoarchaeaeota;c Woesearchaeia;o Candidatus-Pacearchaeota-archaeon-RBG_19FT_COMBO_34_9;f Candidatus-Pacearchaeota-archaeon-RBG_19FT_COMBO_34_9;g Candidatus-Pacearchaeota-archaeon-RBG_19FT_COMBO_34_9	0.0%	0.0%	0.0%
	p Nanoarchaeaeota;c Woesearchaeia;o Candidatus-Staskawiczbacteria-bacterium-RIFOXYA2_FULL_32_7;f Candidatus-Staskawiczbacteria-bacterium-RIFOXYA2_FULL_32_7;g Candidatus-Staskawiczbacteria-bacterium-RIFOXYA2_FULL_32_7	0.1%	0.1%	0.0%
	p Nanoarchaeaeota;c Woesearchaeia;o uncultured-euryarchaeote;f uncultured-euryarchaeote;g uncultured-euryarchaeote	0.0%	0.0%	0.0%
	p Nitrospirae;c Nitrospira;o Nitrospirales;f Nitrospiraceae;g Nitrospira	0.5%	0.4%	0.7%
	p Patescibacteria;Other;Other;Other;Other	0.0%	0.0%	0.0%
	p Patescibacteria;c ABY1;Other;Other;Other	0.0%	0.0%	0.0%
	p Patescibacteria;c ABY1;o Candidatus-Kuenenbacteria;f uncultured-bacterium;g uncultured-bacterium	0.0%	0.0%	0.1%
	p Patescibacteria;c ABY1;o Candidatus-Magasanikbacteria;Other;Other	0.0%	0.0%	0.0%
	p Patescibacteria;c ABY1;o Candidatus-Magasanikbacteria;f uncultured-bacterium;g uncultured-bacterium	0.0%	0.0%	0.0%
	p Patescibacteria;c ABY1;o Candidatus-Uhrbacteria;Other;Other	0.0%	0.0%	0.0%
	p Patescibacteria;c Berkelbacteria;o uncultured-bacterium;f uncultured-bacterium;g uncultured-bacterium	0.0%	0.0%	0.0%
	p Patescibacteria;c Gracilibacteria;o Candidatus-Abawacabacteria;Other;Other	0.0%	0.0%	0.0%
	p Patescibacteria;c Gracilibacteria;o Candidatus-Abawacabacteria;f Candidatus-Abawacabacteria-bacterium-RBG_16_42_10;g Candidatus-Abawacabacteria-bacterium-RBG_16_42_10	0.0%	0.0%	0.0%
	p Patescibacteria;c Gracilibacteria;o Candidatus-Peribacteria;f Candidatus-Peribacteria-bacterium-RIFCSPHIGHO2_02_FULL_53_20;g Candidatus-Peribacteria-bacterium-RIFCSPHIGHO2_02_FULL_53_20	0.0%	0.0%	0.0%
	p Patescibacteria;c Microgenomatia;o Candidatus-Woesebacteria;Other;Other	0.0%	0.0%	0.0%
	p Patescibacteria;c Parcubacteria;Other;Other;Other	0.0%	0.0%	0.0%
	p Patescibacteria;c Parcubacteria;o Candidatus-Azambacteria;Other;Other	0.0%	0.0%	0.0%
	p Patescibacteria;c Parcubacteria;o Candidatus-Azambacteria;f uncultured-bacterium;g uncultured-bacterium	0.0%	0.0%	0.0%
	p Patescibacteria;c Parcubacteria;o Candidatus-Kaiserbacteria;Other;Other	0.0%	0.0%	0.0%
	p Patescibacteria;c Parcubacteria;o Candidatus-Nomurabacteria;Other;Other	0.0%	0.0%	0.0%
	p Patescibacteria;c Parcubacteria;o Candidatus-Terrybacteria;f uncultured-bacterium;g uncultured-bacterium	0.0%	0.0%	0.0%
	p Patescibacteria;c Parcubacteria;o Candidatus-Yanofskybacteria;Other;Other	0.0%	0.0%	0.0%
	p Patescibacteria;c Parcubacteria;o Candidatus-Yanofskybacteria;f Candidatus-Yanofskybacteria-bacterium-RIFCSPHIGHO2_02_FULL_46_19;g Candidatus-Yanofskybacteria-bacterium-RIFCSPHIGHO2_02_FULL_46_19	0.0%	0.0%	0.0%
	p Patescibacteria;c Parcubacteria;o Candidatus-Yanofskybacteria;f uncultured-bacterium;g uncultured-bacterium	0.0%	0.0%	0.0%
	p Patescibacteria;c Parcubacteria;o Candidatus-Yanofskybacteria;f uncultured-deep-sea-bacterium;g uncultured-deep-sea-bacterium	0.0%	0.0%	0.0%
	p Patescibacteria;c Parcubacteria;o GWA2-38-13b;f uncultured-bacterium;g uncultured-bacterium	0.0%	0.0%	0.0%
	p Patescibacteria;c Parcubacteria;o GWA2-38-13b;f uncultured-deep-sea-bacterium;g uncultured-deep-sea-bacterium	0.0%	0.0%	0.0%
	p Patescibacteria;c Parcubacteria;o uncultured-bacterium;f uncultured-bacterium;g uncultured-bacterium	0.0%	0.0%	0.0%
	p Patescibacteria;c Saccharimonadia;o Saccharimonadales;Other;Other	0.2%	0.2%	0.3%
	p Patescibacteria;c Saccharimonadia;o Saccharimonadales;f Saccharimonadaceae;g uncultured-bacterium	0.1%	0.0%	0.2%
	p Patescibacteria;c Saccharimonadia;o Saccharimonadales;f uncultured-Candidatus-Saccharibacteria-bacterium;g uncultured-Candidatus-Saccharibacteria-bacterium	0.0%	0.0%	0.1%
	p Patescibacteria;c Saccharimonadia;o Saccharimonadales;f uncultured-bacterium;g uncultured-bacterium	0.1%	0.1%	0.1%
	p Patescibacteria;c WWE3;Other;Other;Other	0.0%	0.0%	0.0%
	p Planctomycetes;Other;Other;Other;Other	0.0%	0.0%	0.0%
	p Planctomycetes;c BD7-11;o uncultured-Planctomycetales-bacterium;f uncultured-Planctomycetales-bacterium;g uncultured-Planctomycetales-bacterium	0.0%	0.0%	0.0%
	p Planctomycetes;c BD7-11;o uncultured-bacterium;f uncultured-bacterium;g uncultured-bacterium	0.0%	0.0%	0.0%
	p Planctomycetes;c OM190;Other;Other;Other	0.0%	0.0%	0.0%
	p Planctomycetes;c OM190;o uncultured-bacterium;f uncultured-bacterium;g uncultured-bacterium	0.0%	0.0%	0.0%
	p Planctomycetes;c OM190;o uncultured-soil-bacterium;f uncultured-soil-bacterium;g uncultured-soil-bacterium	0.0%	0.0%	0.0%
	p Planctomycetes;c Phycisphaerae;Other;Other;Other	0.2%	0.2%	0.2%
	p Planctomycetes;c Phycisphaerae;o CCM11a;Other;Other	0.1%	0.0%	0.1%
	p Planctomycetes;c Phycisphaerae;o CCM11a;f uncultured-Planctomycetia-bacterium;g uncultured-Planctomycetia-bacterium	0.0%	0.0%	0.0%
	p Planctomycetes;c Phycisphaerae;o CCM11a;f uncultured-bacterium;g uncultured-bacterium	0.1%	0.2%	0.1%
	p Planctomycetes;c Phycisphaerae;o Phycisphaerales;Other;Other	0.0%	0.0%	0.0%
	p Planctomycetes;c Phycisphaerae;o Phycisphaerales;f Phycisphaeraceae;Other	0.0%	0.0%	0.1%
	p Planctomycetes;c Phycisphaerae;o Phycisphaerales;f Phycisphaeraceae;g uncultured	0.1%	0.1%	0.1%
	p Planctomycetes;c Phycisphaerae;o Pla1-lineage;Other;Other	0.0%	0.0%	0.0%
	p Planctomycetes;c Phycisphaerae;o Pla1-lineage;f uncultured-bacterium;g uncultured-bacterium	0.0%	0.0%	0.0%
	p Planctomycetes;c Phycisphaerae;o Tepidisphaerales;f CPla-3-termite-group;Other	0.0%	0.0%	0.0%
	p Planctomycetes;c Phycisphaerae;o Tepidisphaerales;f CPla-3-termite-group;g uncultured-bacterium	0.0%	0.0%	0.0%
	p Planctomycetes;c Phycisphaerae;o Tepidisphaerales;f CPla-3-termite-group;g uncultured-soil-bacterium-PBS-22	0.0%	0.0%	0.0%
	p Planctomycetes;c Phycisphaerae;o Tepidisphaerales;f Tepidisphaeraceae;g uncultured-bacterium	0.0%	0.0%	0.0%
	p Planctomycetes;c Phycisphaerae;o Tepidisphaerales;f WD2101-soil-group;Other	0.4%	0.2%	0.6%
	p Planctomycetes;c Phycisphaerae;o Tepidisphaerales;f WD2101-soil-group;g uncultured-bacterium	0.6%	0.5%	0.7%
	p Planctomycetes;c Phycisphaerae;o Tepidisphaerales;f WD2101-soil-group;g uncultured-planctomycete	0.0%	0.0%	0.1%
	p Planctomycetes;c Phycisphaerae;o Tepidisphaerales;f WD2101-soil-group;g uncultured-soil-bacterium	0.0%	0.0%	0.0%
	p Planctomycetes;c Phycisphaerae;o mle1-8;Other;Other	0.0%	0.0%	0.0%
	p Planctomycetes;c Phycisphaerae;o mle1-8;f uncultured-bacterium;g uncultured-bacterium	0.1%	0.0%	0.1%
	p Planctomycetes;c Pla3-lineage;Other;Other;Other	0.0%	0.0%	0.0%

	p Planctomycetes;c Pla4-lineage;Other;Other;Other	0.0%	0.0%	0.0%
	p Planctomycetes;c Pla4-lineage;o uncultured-bacterium;f uncultured-bacterium;g uncultured-bacterium	0.0%	0.0%	0.0%
	p Planctomycetes;c Pla4-lineage;o uncultured-prokaryote;f uncultured-prokaryote;g uncultured-prokaryote	0.0%	0.0%	0.0%
	p Planctomycetes;c Planctomycetacia;o Gemmatales;f Gemmataceae;Other	0.0%	0.0%	0.0%
	p Planctomycetes;c Planctomycetacia;o Gemmatales;f Gemmataceae;g Fimbriiglobus	0.0%	0.0%	0.0%
	p Planctomycetes;c Planctomycetacia;o Gemmatales;f Gemmataceae;g Gemmata	0.1%	0.1%	0.1%
	p Planctomycetes;c Planctomycetacia;o Gemmatales;f Gemmataceae;g uncultured	0.1%	0.0%	0.1%
	p Planctomycetes;c Planctomycetacia;o Pirellulales;f Pirellulaceae;Other	0.0%	0.0%	0.1%
	p Planctomycetes;c Planctomycetacia;o Pirellulales;f Pirellulaceae;g Bythopirellula	0.0%	0.0%	0.0%
	p Planctomycetes;c Planctomycetacia;o Pirellulales;f Pirellulaceae;g Pirellula	0.1%	0.1%	0.1%
	p Planctomycetes;c Planctomycetacia;o Pirellulales;f Pirellulaceae;g uncultured	0.1%	0.0%	0.1%
	p Planctomycetes;c Planctomycetacia;o Planctomycetales;Other;Other	0.0%	0.0%	0.0%
	p Planctomycetes;c Planctomycetacia;o Planctomycetales;f Rubinisphaeraceae;g Planctomicrobium	0.0%	0.0%	0.0%
	p Planctomycetes;c Planctomycetacia;o Planctomycetales;f Schlesneriaceae;g Planctopirus	0.0%	0.0%	0.0%
	p Planctomycetes;c Planctomycetacia;o Planctomycetales;f uncultured;Other	0.0%	0.0%	0.0%
	p Planctomycetes;c Planctomycetacia;o Planctomycetales;f uncultured;g uncultured-Planctomyces-sp.	0.0%	0.0%	0.0%
	p Planctomycetes;c Planctomycetacia;o Planctomycetales;f uncultured;g uncultured-bacterium	0.0%	0.0%	0.0%
	p Planctomycetes;c Planctomycetacia;o uncultured;f uncultured-bacterium;g uncultured-bacterium	0.0%	0.0%	0.0%
	p Proteobacteria;Other;Other;Other;Other	0.0%	0.0%	0.0%
	p Proteobacteria;c Alphaproteobacteria;Other;Other;Other	1.8%	1.7%	2.0%
	p Proteobacteria;c Alphaproteobacteria;o Azospirillales;f Azospirillaceae;g Azospirillum	0.0%	0.0%	0.0%
	p Proteobacteria;c Alphaproteobacteria;o Azospirillales;f Inquilinaceae;g Inquilinus	0.0%	0.0%	0.0%
	p Proteobacteria;c Alphaproteobacteria;o Azospirillales;f uncultured;g uncultured-bacterium	0.0%	0.1%	0.0%
	p Proteobacteria;c Alphaproteobacteria;o Caulobacterales;Other;Other	0.0%	0.0%	0.0%
	p Proteobacteria;c Alphaproteobacteria;o Caulobacterales;f Caulobacteraceae;Other	0.0%	0.0%	0.0%
	p Proteobacteria;c Alphaproteobacteria;o Caulobacterales;f Caulobacteraceae;g Brevundimonas	0.0%	0.0%	0.0%
	p Proteobacteria;c Alphaproteobacteria;o Caulobacterales;f Caulobacteraceae;g Phenylobacterium	0.1%	0.1%	0.1%
	p Proteobacteria;c Alphaproteobacteria;o Caulobacterales;f Caulobacteraceae;g uncultured	0.0%	0.0%	0.0%
	p Proteobacteria;c Alphaproteobacteria;o Caulobacterales;f Hyphomonadaceae;g Hirschia	0.1%	0.1%	0.0%
	p Proteobacteria;c Alphaproteobacteria;o Caulobacterales;f Hyphomonadaceae;g uncultured	0.0%	0.1%	0.0%
	p Proteobacteria;c Alphaproteobacteria;o Caulobacterales;f Parvularculaceae;g Amphiplicatus	0.0%	0.0%	0.0%
	p Proteobacteria;c Alphaproteobacteria;o Elsterales;f Elsteraceae;g Lacibacterium	0.0%	0.0%	0.0%
	p Proteobacteria;c Alphaproteobacteria;o Elsterales;f uncultured;Other	0.0%	0.0%	0.0%
	p Proteobacteria;c Alphaproteobacteria;o Elsterales;f uncultured;g uncultured-Acetobacteraceae-bacterium	0.0%	0.0%	0.0%
	p Proteobacteria;c Alphaproteobacteria;o Elsterales;f uncultured;g uncultured-alpha-proteobacterium	0.0%	0.0%	0.0%
	p Proteobacteria;c Alphaproteobacteria;o Elsterales;f uncultured;g uncultured-bacterium	0.0%	0.0%	0.0%
	p Proteobacteria;c Alphaproteobacteria;o Holosporales;f Holosporaceae;g uncultured	0.0%	0.0%	0.0%
	p Proteobacteria;c Alphaproteobacteria;o Micropepsales;f Micropepsaceae;g uncultured	0.1%	0.0%	0.1%
	p Proteobacteria;c Alphaproteobacteria;o Reyranellales;f Reyranellaceae;g Reyranella	0.1%	0.1%	0.1%
	p Proteobacteria;c Alphaproteobacteria;o Reyranellales;f Reyranellaceae;g uncultured	0.1%	0.2%	0.0%
	p Proteobacteria;c Alphaproteobacteria;o Rhizobiales;Other;Other	0.0%	0.1%	0.0%
	p Proteobacteria;c Alphaproteobacteria;o Rhizobiales;f A0839;g uncultured-bacterium	0.0%	0.0%	0.1%
	p Proteobacteria;c Alphaproteobacteria;o Rhizobiales;f Amb-16S-1323;g uncultured-bacterium	0.0%	0.0%	0.0%
	p Proteobacteria;c Alphaproteobacteria;o Rhizobiales;f Beijerinckiaceae;Other	0.0%	0.0%	0.0%
	p Proteobacteria;c Alphaproteobacteria;o Rhizobiales;f Beijerinckiaceae;g Bosea	0.0%	0.0%	0.0%
	p Proteobacteria;c Alphaproteobacteria;o Rhizobiales;f Beijerinckiaceae;g Microvirga	0.1%	0.1%	0.1%
	p Proteobacteria;c Alphaproteobacteria;o Rhizobiales;f D05-2;g uncultured-bacterium	0.0%	0.0%	0.0%
	p Proteobacteria;c Alphaproteobacteria;o Rhizobiales;f Devosiaceae;Other	0.1%	0.1%	0.1%
	p Proteobacteria;c Alphaproteobacteria;o Rhizobiales;f Devosiaceae;g Devosia	0.0%	0.0%	0.0%
	p Proteobacteria;c Alphaproteobacteria;o Rhizobiales;f Devosiaceae;g uncultured	0.1%	0.2%	0.0%
	p Proteobacteria;c Alphaproteobacteria;o Rhizobiales;f Hyphomicrobiaceae;Other	0.0%	0.0%	0.0%
	p Proteobacteria;c Alphaproteobacteria;o Rhizobiales;f Hyphomicrobiaceae;g Hyphomicrobium	0.0%	0.0%	0.0%
	p Proteobacteria;c Alphaproteobacteria;o Rhizobiales;f Hyphomicrobiaceae;g Pedomicrobium	0.3%	0.5%	0.1%
	p Proteobacteria;c Alphaproteobacteria;o Rhizobiales;f Hyphomicrobiaceae;g uncultured	0.0%	0.0%	0.0%
	p Proteobacteria;c Alphaproteobacteria;o Rhizobiales;f KF-JG30-B3;Other	0.0%	0.0%	0.0%
	p Proteobacteria;c Alphaproteobacteria;o Rhizobiales;f KF-JG30-B3;g uncultured-bacterium	0.8%	0.7%	0.9%
	p Proteobacteria;c Alphaproteobacteria;o Rhizobiales;f Labraceae;g Labrys	0.0%	0.0%	0.0%
	p Proteobacteria;c Alphaproteobacteria;o Rhizobiales;f Methyloligellaceae;Other	0.0%	0.1%	0.0%
	p Proteobacteria;c Alphaproteobacteria;o Rhizobiales;f Methyloligellaceae;g uncultured	0.3%	0.3%	0.3%
	p Proteobacteria;c Alphaproteobacteria;o Rhizobiales;f Rhizobiaceae;Other	0.1%	0.1%	0.1%
	p Proteobacteria;c Alphaproteobacteria;o Rhizobiales;f Rhizobiaceae;g Allorhizobium-Neorhizobium-Pararhizobium-Rhizobium	0.3%	0.2%	0.3%
	p Proteobacteria;c Alphaproteobacteria;o Rhizobiales;f Rhizobiaceae;g Ensifer	0.3%	0.5%	0.1%
	p Proteobacteria;c Alphaproteobacteria;o Rhizobiales;f Rhizobiaceae;g Mesorhizobium	0.0%	0.0%	0.0%
	p Proteobacteria;c Alphaproteobacteria;o Rhizobiales;f Rhizobiales-Incertae-Sedis;g Bauldia	0.1%	0.1%	0.1%
	p Proteobacteria;c Alphaproteobacteria;o Rhizobiales;f Rhizobiales-Incertae-Sedis;g uncultured	0.2%	0.2%	0.2%
	p Proteobacteria;c Alphaproteobacteria;o Rhizobiales;f Rhodomicrobiaceae;g Rhodomicrobium	0.0%	0.0%	0.0%

p Proteobacteria;c Alphaproteobacteria;o Rhizobiales;f Xanthobacteraceae;Other	1.0%	1.0%	1.0%
p Proteobacteria;c Alphaproteobacteria;o Rhizobiales;f Xanthobacteraceae;g Ancylobacter	0.0%	0.0%	0.0%
p Proteobacteria;c Alphaproteobacteria;o Rhizobiales;f Xanthobacteraceae;g Bradyrhizobium	0.5%	0.5%	0.4%
p Proteobacteria;c Alphaproteobacteria;o Rhizobiales;f Xanthobacteraceae;g Pseudolabrys	0.1%	0.1%	0.1%
p Proteobacteria;c Alphaproteobacteria;o Rhizobiales;f Xanthobacteraceae;g Rhodoplanes	0.1%	0.1%	0.1%
p Proteobacteria;c Alphaproteobacteria;o Rhizobiales;f Xanthobacteraceae;g uncultured	0.7%	0.7%	0.6%
p Proteobacteria;c Alphaproteobacteria;o Rhizobiales;f uncultured;Other	0.0%	0.0%	0.0%
p Proteobacteria;c Alphaproteobacteria;o Rhizobiales;f uncultured;g uncultured-Rhizobiales-bacterium	0.0%	0.0%	0.0%
p Proteobacteria;c Alphaproteobacteria;o Rhizobiales;f uncultured;g uncultured-bacterium	0.1%	0.1%	0.1%
p Proteobacteria;c Alphaproteobacteria;o Rhodobacterales;f Rhodobacteraceae;Other	0.0%	0.0%	0.0%
p Proteobacteria;c Alphaproteobacteria;o Rhodobacterales;f Rhodobacteraceae;g uncultured	0.0%	0.0%	0.0%
p Proteobacteria;c Alphaproteobacteria;o Rhodospirillales;f Rhodospirillaceae;g uncultured	0.0%	0.0%	0.1%
p Proteobacteria;c Alphaproteobacteria;o Rhodospirillales;f uncultured;Other	0.0%	0.0%	0.0%
p Proteobacteria;c Alphaproteobacteria;o Rhodospirillales;f uncultured;g uncultured-Rhodospirillaceae-bacterium	0.0%	0.0%	0.0%
p Proteobacteria;c Alphaproteobacteria;o Rhodovibrionales;f Fodinicurvataceae;g uncultured	0.0%	0.0%	0.0%
p Proteobacteria;c Alphaproteobacteria;o Sneathiellales;f Sneathiellaceae;g uncultured	0.0%	0.0%	0.0%
p Proteobacteria;c Alphaproteobacteria;o Sphingomonadales;f Sphingomonadaceae;Other	0.1%	0.2%	0.1%
p Proteobacteria;c Alphaproteobacteria;o Sphingomonadales;f Sphingomonadaceae;g Altererythrobacter	0.1%	0.1%	0.1%
p Proteobacteria;c Alphaproteobacteria;o Sphingomonadales;f Sphingomonadaceae;g Novosphingobium	0.2%	0.3%	0.1%
p Proteobacteria;c Alphaproteobacteria;o Sphingomonadales;f Sphingomonadaceae;g Sphingobium	0.1%	0.1%	0.1%
p Proteobacteria;c Alphaproteobacteria;o Sphingomonadales;f Sphingomonadaceae;g Sphingomonas	1.3%	1.6%	1.1%
p Proteobacteria;c Alphaproteobacteria;o Sphingomonadales;f Sphingomonadaceae;g uncultured	0.0%	0.0%	0.0%
p Proteobacteria;c Alphaproteobacteria;o Tistrellales;f Geminicoccaceae;g Candidatus-Alysiosphaera	0.0%	0.0%	0.0%
p Proteobacteria;c Alphaproteobacteria;o Tistrellales;f Geminicoccaceae;g uncultured	0.0%	0.0%	0.0%
p Proteobacteria;c Alphaproteobacteria;o uncultured;Other;Other	0.2%	0.2%	0.2%
p Proteobacteria;c Alphaproteobacteria;o uncultured;f uncultured-Acetobacteraceae-bacterium;g uncultured-Acetobacteraceae-bacterium	0.0%	0.0%	0.0%
p Proteobacteria;c Alphaproteobacteria;o uncultured;f uncultured-Rhodospirillaceae-bacterium;g uncultured-Rhodospirillaceae-bacterium	0.0%	0.0%	0.0%
p Proteobacteria;c Alphaproteobacteria;o uncultured;f uncultured-Rhodospirillales-bacterium;g uncultured-Rhodospirillales-bacterium	0.0%	0.0%	0.0%
p Proteobacteria;c Alphaproteobacteria;o uncultured;f uncultured-Stella-sp.;g uncultured-Stella-sp.	0.0%	0.0%	0.0%
p Proteobacteria;c Alphaproteobacteria;o uncultured;f uncultured-bacterium;g uncultured-bacterium	0.1%	0.1%	0.1%
p Proteobacteria;c Alphaproteobacteria;o uncultured;f uncultured-soil-bacterium;g uncultured-soil-bacterium	0.0%	0.1%	0.0%
p Proteobacteria;c Deltaproteobacteria;Other;Other;Other	0.2%	0.3%	0.2%
p Proteobacteria;c Deltaproteobacteria;o Bdellovibrionales;f Bdellovibrionaceae;g Bdellovibrio	0.0%	0.1%	0.0%
p Proteobacteria;c Deltaproteobacteria;o Desulfarculales;f Desulfarculaceae;g uncultured	0.2%	0.1%	0.2%
p Proteobacteria;c Deltaproteobacteria;o Desulfuromonadales;Other;Other	0.0%	0.0%	0.0%
p Proteobacteria;c Deltaproteobacteria;o Desulfuromonadales;f Geobacteraceae;g Geobacter	0.0%	0.0%	0.1%
p Proteobacteria;c Deltaproteobacteria;o MBNT15;Other;Other	0.1%	0.0%	0.2%
p Proteobacteria;c Deltaproteobacteria;o MBNT15;f uncultured-bacterium;g uncultured-bacterium	0.0%	0.0%	0.0%
p Proteobacteria;c Deltaproteobacteria;o MBNT15;f uncultured-proteobacterium;g uncultured-proteobacterium	0.0%	0.0%	0.1%
p Proteobacteria;c Deltaproteobacteria;o Myxococcales;Other;Other	0.1%	0.1%	0.0%
p Proteobacteria;c Deltaproteobacteria;o Myxococcales;f Archangiaceae;g Anaeromyxobacter	0.0%	0.0%	0.0%
p Proteobacteria;c Deltaproteobacteria;o Myxococcales;f BIrii41;Other	0.0%	0.0%	0.0%
p Proteobacteria;c Deltaproteobacteria;o Myxococcales;f BIrii41;g uncultured-bacterium	0.1%	0.1%	0.1%
p Proteobacteria;c Deltaproteobacteria;o Myxococcales;f Blfdi19;Other	0.0%	0.0%	0.0%
p Proteobacteria;c Deltaproteobacteria;o Myxococcales;f Blfdi19;g uncultured-bacterium	0.0%	0.0%	0.0%
p Proteobacteria;c Deltaproteobacteria;o Myxococcales;f Eel-36e1D6;Other	0.0%	0.0%	0.0%
p Proteobacteria;c Deltaproteobacteria;o Myxococcales;f Haliangiaceae;g Haliangium	0.3%	0.4%	0.3%
p Proteobacteria;c Deltaproteobacteria;o Myxococcales;f Nannocystaceae;Other	0.0%	0.0%	0.0%
p Proteobacteria;c Deltaproteobacteria;o Myxococcales;f Nannocystaceae;g Nannocystis	0.0%	0.0%	0.0%
p Proteobacteria;c Deltaproteobacteria;o Myxococcales;f Nannocystaceae;g uncultured	0.0%	0.0%	0.0%
p Proteobacteria;c Deltaproteobacteria;o Myxococcales;f Nannocystaceae;g uncultured-bacterium	0.0%	0.0%	0.0%
p Proteobacteria;c Deltaproteobacteria;o Myxococcales;f P3OB-42;Other	0.0%	0.0%	0.0%
p Proteobacteria;c Deltaproteobacteria;o Myxococcales;f P3OB-42;g uncultured-bacterium	0.0%	0.0%	0.0%
p Proteobacteria;c Deltaproteobacteria;o Myxococcales;f Phaselicystidaceae;g Phaselicystis	0.0%	0.0%	0.0%
p Proteobacteria;c Deltaproteobacteria;o Myxococcales;f Polyangiaceae;Other	0.0%	0.0%	0.0%
p Proteobacteria;c Deltaproteobacteria;o Myxococcales;f Polyangiaceae;g Pajaroellobacter	0.0%	0.0%	0.0%
p Proteobacteria;c Deltaproteobacteria;o Myxococcales;f Polyangiaceae;g Polyangium	0.0%	0.0%	0.0%
p Proteobacteria;c Deltaproteobacteria;o Myxococcales;f Sandaracinaceae;g Sandaracinus	0.0%	0.0%	0.0%
p Proteobacteria;c Deltaproteobacteria;o Myxococcales;f Sandaracinaceae;g uncultured	0.0%	0.0%	0.1%
p Proteobacteria;c Deltaproteobacteria;o Myxococcales;f UASB-TL25;g uncultured-bacterium	0.0%	0.0%	0.0%
p Proteobacteria;c Deltaproteobacteria;o Myxococcales;f Vulgatibacteraceae;g Vulgatibacter	0.0%	0.0%	0.0%
p Proteobacteria;c Deltaproteobacteria;o Myxococcales;f bacteriap25;Other	0.5%	0.5%	0.5%
p Proteobacteria;c Deltaproteobacteria;o Myxococcales;f bacteriap25;g uncultured-delta-proteobacterium	0.0%	0.0%	0.0%
p Proteobacteria;c Deltaproteobacteria;o Myxococcales;f bacteriap25;g uncultured-proteobacterium	0.1%	0.1%	0.1%
p Proteobacteria;c Deltaproteobacteria;o Myxococcales;f bacteriap25;g uncultured-soil-bacterium	0.1%	0.1%	0.0%
p Proteobacteria;c Deltaproteobacteria;o Myxococcales;f bacteriap25;g unidentified	0.0%	0.0%	0.0%
p Proteobacteria;c Deltaproteobacteria;o Myxococcales;f mle1-27;Other	0.0%	0.0%	0.0%

p Proteobacteria;c Deltaproteobacteria;o Myxococcales;f mle1-27;g uncultured-delta-proteobacterium	0.0%	0.0%	0.0%
p Proteobacteria;c Deltaproteobacteria;o Myxococcales;f uncultured;Other	0.0%	0.0%	0.0%
p Proteobacteria;c Deltaproteobacteria;o Myxococcales;f uncultured;g uncultured-bacterium	0.0%	0.0%	0.0%
p Proteobacteria;c Deltaproteobacteria;o NB1-j;Other;Other	0.0%	0.0%	0.0%
p Proteobacteria;c Deltaproteobacteria;o NB1-j;f uncultured-Green-Bay-ferromanganous-micronodule-bacterium-MND4;g uncultured-Green-Bay-ferromanganous-micronodule-bacterium-MND4	0.0%	0.0%	0.0%
p Proteobacteria;c Deltaproteobacteria;o NB1-j;f uncultured-bacterium;g uncultured-bacterium	0.3%	0.4%	0.2%
p Proteobacteria;c Deltaproteobacteria;o NB1-j;f uncultured-proteobacterium;g uncultured-proteobacterium	0.0%	0.0%	0.0%
p Proteobacteria;c Deltaproteobacteria;o NB1-j;f uncultured-soil-bacterium;g uncultured-soil-bacterium	0.0%	0.0%	0.0%
p Proteobacteria;c Deltaproteobacteria;o Oligoflexales;f Oligoflexaceae;g Oligoflexus	0.0%	0.0%	0.0%
p Proteobacteria;c Deltaproteobacteria;o RCP2-54;f uncultured-bacterium;g uncultured-bacterium	0.0%	0.0%	0.0%
p Proteobacteria;c Deltaproteobacteria;o RCP2-54;f uncultured-prokaryote;g uncultured-prokaryote	0.0%	0.0%	0.0%
p Proteobacteria;c Deltaproteobacteria;o SAR324-clade(Marine-group-B);f bacterium-enrichment-culture-clone-B30(2011);g bacterium-enrichment-culture-clone-B30(2011)	0.0%	0.0%	0.0%
p Proteobacteria;c Deltaproteobacteria;o Syntrophobacterales;f Syntrophaceae;g uncultured	0.1%	0.0%	0.1%
p Proteobacteria;c Gammaproteobacteria;Other;Other;Other	0.5%	0.6%	0.4%
p Proteobacteria;c Gammaproteobacteria;o Acidiferrobacterales;f Acidiferrobacteraceae;g Sulfurifustis	0.0%	0.0%	0.0%
p Proteobacteria;c Gammaproteobacteria;o Aeromonadales;f Aeromonadaceae;g Aeromonas	0.0%	0.0%	0.0%
p Proteobacteria;c Gammaproteobacteria;o Betaproteobacteriales;Other;Other	1.7%	1.4%	2.1%
p Proteobacteria;c Gammaproteobacteria;o Betaproteobacteriales;f A21b;Other	0.0%	0.0%	0.0%
p Proteobacteria;c Gammaproteobacteria;o Betaproteobacteriales;f A21b;g uncultured-bacterium	0.0%	0.0%	0.0%
p Proteobacteria;c Gammaproteobacteria;o Betaproteobacteriales;f B1-7BS;Other	0.0%	0.0%	0.0%
p Proteobacteria;c Gammaproteobacteria;o Betaproteobacteriales;f B1-7BS;g uncultured-bacterium	0.1%	0.1%	0.1%
p Proteobacteria;c Gammaproteobacteria;o Betaproteobacteriales;f Burkholderiaceae;Other	0.2%	0.2%	0.3%
p Proteobacteria;c Gammaproteobacteria;o Betaproteobacteriales;f Burkholderiaceae;g Bordetella	0.0%	0.0%	0.0%
p Proteobacteria;c Gammaproteobacteria;o Betaproteobacteriales;f Burkholderiaceae;g Burkholderia-Caballeronia-Paraburkholderia	0.0%	0.0%	0.1%
p Proteobacteria;c Gammaproteobacteria;o Betaproteobacteriales;f Burkholderiaceae;g Comamonas	0.1%	0.1%	0.0%
p Proteobacteria;c Gammaproteobacteria;o Betaproteobacteriales;f Burkholderiaceae;g Cupriavidus	0.4%	0.7%	0.1%
p Proteobacteria;c Gammaproteobacteria;o Betaproteobacteriales;f Burkholderiaceae;g Hydrogenophaga	0.0%	0.0%	0.0%
p Proteobacteria;c Gammaproteobacteria;o Betaproteobacteriales;f Burkholderiaceae;g Massilia	0.0%	0.0%	0.0%
p Proteobacteria;c Gammaproteobacteria;o Betaproteobacteriales;f Burkholderiaceae;g Noviherbaspirillum	0.0%	0.0%	0.0%
p Proteobacteria;c Gammaproteobacteria;o Betaproteobacteriales;f Burkholderiaceae;g Ramlibacter	0.2%	0.2%	0.1%
p Proteobacteria;c Gammaproteobacteria;o Betaproteobacteriales;f Burkholderiaceae;g Rhizobacter	0.0%	0.0%	0.0%
p Proteobacteria;c Gammaproteobacteria;o Betaproteobacteriales;f Burkholderiaceae;g Sphaerotilus	0.0%	0.0%	0.0%
p Proteobacteria;c Gammaproteobacteria;o Betaproteobacteriales;f Burkholderiaceae;g Variovorax	0.1%	0.1%	0.0%
p Proteobacteria;c Gammaproteobacteria;o Betaproteobacteriales;f Burkholderiaceae;g uncultured	0.1%	0.0%	0.1%
p Proteobacteria;c Gammaproteobacteria;o Betaproteobacteriales;f Chromobacteriaceae;Other	0.0%	0.0%	0.0%
p Proteobacteria;c Gammaproteobacteria;o Betaproteobacteriales;f Chromobacteriaceae;g Paludibacterium	0.0%	0.0%	0.0%
p Proteobacteria;c Gammaproteobacteria;o Betaproteobacteriales;f Rhodocyclaceae;Other	0.3%	0.1%	0.4%
p Proteobacteria;c Gammaproteobacteria;o Betaproteobacteriales;f Rhodocyclaceae;g Azoarcus	0.0%	0.0%	0.0%
p Proteobacteria;c Gammaproteobacteria;o Betaproteobacteriales;f Rhodocyclaceae;g Thauera	0.0%	0.0%	0.0%
p Proteobacteria;c Gammaproteobacteria;o Betaproteobacteriales;f SC-I-84;Other	0.3%	0.3%	0.2%
p Proteobacteria;c Gammaproteobacteria;o Betaproteobacteriales;f SC-I-84;g uncultured-bacterium	0.2%	0.1%	0.2%
p Proteobacteria;c Gammaproteobacteria;o Betaproteobacteriales;f SC-I-84;g uncultured-beta-proteobacterium	0.1%	0.1%	0.2%
p Proteobacteria;c Gammaproteobacteria;o Betaproteobacteriales;f TRA3-20;Other	0.6%	0.7%	0.5%
p Proteobacteria;c Gammaproteobacteria;o Betaproteobacteriales;f TRA3-20;g uncultured-bacterium	0.1%	0.1%	0.2%
p Proteobacteria;c Gammaproteobacteria;o Betaproteobacteriales;f TRA3-20;g uncultured-gamma-proteobacterium	0.0%	0.0%	0.0%
p Proteobacteria;c Gammaproteobacteria;o CCD24;f uncultured-bacterium;g uncultured-bacterium	0.2%	0.3%	0.1%
p Proteobacteria;c Gammaproteobacteria;o Cellvibrionales;Other;Other	0.0%	0.0%	0.0%
p Proteobacteria;c Gammaproteobacteria;o Cellvibrionales;f Halieaceae;Other	0.0%	0.0%	0.0%
p Proteobacteria;c Gammaproteobacteria;o Diplorickettsiales;f Diplorickettsiaceae;g Aquicella	0.0%	0.0%	0.0%
p Proteobacteria;c Gammaproteobacteria;o EPR3968-O8a-Bc78;f uncultured-gamma-proteobacterium;g uncultured-gamma-proteobacterium	0.0%	0.0%	0.0%
p Proteobacteria;c Gammaproteobacteria;o Enterobacteriales;f Enterobacteriaceae;Other	0.0%	0.0%	0.0%
p Proteobacteria;c Gammaproteobacteria;o Enterobacteriales;f Enterobacteriaceae;g Pantoea	0.0%	0.0%	0.0%
p Proteobacteria;c Gammaproteobacteria;o Gammaproteobacteria-Incertae-Sedis;f Unknown-Family;g uncultured	0.0%	0.0%	0.0%
p Proteobacteria;c Gammaproteobacteria;o JG36-GS-52;f uncultured-bacterium;g uncultured-bacterium	0.0%	0.0%	0.0%
p Proteobacteria;c Gammaproteobacteria;o JG36-TzT-191;Other;Other	0.0%	0.0%	0.0%
p Proteobacteria;c Gammaproteobacteria;o JG36-TzT-191;f uncultured-bacterium;g uncultured-bacterium	0.0%	0.0%	0.0%
p Proteobacteria;c Gammaproteobacteria;o KI89A-clade;Other;Other	0.0%	0.0%	0.0%
p Proteobacteria;c Gammaproteobacteria;o PLTA13;Other;Other	0.0%	0.0%	0.0%
p Proteobacteria;c Gammaproteobacteria;o PLTA13;f uncultured-bacterium;g uncultured-bacterium	0.7%	1.2%	0.1%
p Proteobacteria;c Gammaproteobacteria;o PLTA13;f uncultured-proteobacterium;g uncultured-proteobacterium	0.0%	0.0%	0.0%
p Proteobacteria;c Gammaproteobacteria;o Pseudomonadales;f Moraxellaceae;g Acinetobacter	0.0%	0.0%	0.0%
p Proteobacteria;c Gammaproteobacteria;o Pseudomonadales;f Moraxellaceae;g uncultured	0.0%	0.0%	0.0%
p Proteobacteria;c Gammaproteobacteria;o Pseudomonadales;f Pseudomonadaceae;Other	0.0%	0.0%	0.0%
p Proteobacteria;c Gammaproteobacteria;o Pseudomonadales;f Pseudomonadaceae;g Azotobacter	0.0%	0.0%	0.0%
p Proteobacteria;c Gammaproteobacteria;o Pseudomonadales;f Pseudomonadaceae;g Pseudomonas	0.4%	0.7%	0.1%
p Proteobacteria;c Gammaproteobacteria;o R7C24;Other;Other	0.0%	0.0%	0.0%

	p Proteobacteria;c Gammaproteobacteria;o R7C24;f uncultured-bacterium;g uncultured-bacterium	0.0%	0.0%	0.0%
	p Proteobacteria;c Gammaproteobacteria;o Salinisphaerales;Other;Other	0.0%	0.0%	0.0%
	p Proteobacteria;c Gammaproteobacteria;o Salinisphaerales;f Solimonadaceae;Other	0.0%	0.0%	0.0%
	p Proteobacteria;c Gammaproteobacteria;o Steroidobacterales;f Steroidobacteraceae;g uncultured	1.4%	2.0%	0.7%
	p Proteobacteria;c Gammaproteobacteria;o Xanthomonadales;f Rhodanobacteraceae;Other	0.0%	0.0%	0.0%
	p Proteobacteria;c Gammaproteobacteria;o Xanthomonadales;f Rhodanobacteraceae;g Dokdonella	0.0%	0.0%	0.0%
	p Proteobacteria;c Gammaproteobacteria;o Xanthomonadales;f Rhodanobacteraceae;g Dyella	0.0%	0.0%	0.0%
	p Proteobacteria;c Gammaproteobacteria;o Xanthomonadales;f Rhodanobacteraceae;g uncultured	0.0%	0.0%	0.0%
	p Proteobacteria;c Gammaproteobacteria;o Xanthomonadales;f Xanthomonadaceae;Other	0.0%	0.0%	0.0%
	p Proteobacteria;c Gammaproteobacteria;o Xanthomonadales;f Xanthomonadaceae;g Arenimonas	0.1%	0.1%	0.0%
	p Proteobacteria;c Gammaproteobacteria;o Xanthomonadales;f Xanthomonadaceae;g Luteimonas	0.0%	0.0%	0.0%
	p Proteobacteria;c Gammaproteobacteria;o Xanthomonadales;f Xanthomonadaceae;g Lysobacter	0.6%	1.2%	0.0%
	p Proteobacteria;c Gammaproteobacteria;o Xanthomonadales;f Xanthomonadaceae;g Pseudoxanthomonas	0.0%	0.1%	0.0%
	p Proteobacteria;c Gammaproteobacteria;o Xanthomonadales;f Xanthomonadaceae;g Stenotrophomonas	0.0%	0.0%	0.0%
	p Proteobacteria;c Gammaproteobacteria;o Xanthomonadales;f Xanthomonadaceae;g Thermomonas	0.0%	0.0%	0.0%
	p Rokubacteria;c NC10;o Rokubacteriales;Other;Other	0.0%	0.0%	0.0%
	p Rokubacteria;c NC10;o Rokubacteriales;f uncultured-Gram-positive-bacterium;g uncultured-Gram-positive-bacterium	0.1%	0.1%	0.0%
	p Rokubacteria;c NC10;o Rokubacteriales;f uncultured-bacterium;g uncultured-bacterium	1.2%	1.5%	0.8%
	p Thaumarchaeota;c Group-1.1c;Other;Other;Other	0.0%	0.0%	0.0%
	p Thaumarchaeota;c Nitrososphaeria;o Nitrosopumilales;f Nitrosopumilaceae;g Candidatus-Nitrosotenuis	0.0%	0.0%	0.0%
	p Thaumarchaeota;c Nitrososphaeria;o Nitrososphaerales;f Nitrososphaeraceae;Other	0.5%	0.5%	0.5%
	p Thaumarchaeota;c Nitrososphaeria;o Nitrososphaerales;f Nitrososphaeraceae;g Candidatus-Nitrocosmicus	0.1%	0.1%	0.1%
	p Thaumarchaeota;c Nitrososphaeria;o Nitrososphaerales;f Nitrososphaeraceae;g Candidatus-Nitrososphaera	0.4%	0.4%	0.4%
	p Thaumarchaeota;c Nitrososphaeria;o Nitrososphaerales;f Nitrososphaeraceae;g uncultured-archaeon	0.3%	0.3%	0.2%
	p Thaumarchaeota;c Nitrososphaeria;o Nitrososphaerales;f Nitrososphaeraceae;g uncultured-bacterium	0.0%	0.0%	0.0%
	p Thaumarchaeota;c Nitrososphaeria;o Nitrosotaleales;f Nitrosotaleaceae;g uncultured-archaeon	0.0%	0.0%	0.0%
	p Verrucomicrobia;c Verrucomicrobiae;Other;Other;Other	0.0%	0.0%	0.0%
	p Verrucomicrobia;c Verrucomicrobiae;o Chthoniobacterales;Other;Other	0.1%	0.1%	0.1%
	p Verrucomicrobia;c Verrucomicrobiae;o Chthoniobacterales;f Chthoniobacteraceae;Other	0.0%	0.0%	0.0%
	p Verrucomicrobia;c Verrucomicrobiae;o Chthoniobacterales;f Chthoniobacteraceae;g Candidatus-Udaeobacter	1.9%	1.0%	2.7%
	p Verrucomicrobia;c Verrucomicrobiae;o Chthoniobacterales;f Xiphinematobacteraceae;g Candidatus-Xiphinematobacter	0.2%	0.3%	0.1%
	p Verrucomicrobia;c Verrucomicrobiae;o Methylacidiphilales;f Methylacidiphilaceae;g uncultured	0.0%	0.0%	0.0%
	p Verrucomicrobia;c Verrucomicrobiae;o Opitutales;f Opitutaceae;Other	1.2%	2.3%	0.1%
	p Verrucomicrobia;c Verrucomicrobiae;o Opitutales;f Opitutaceae;g Lacunisphaera	0.0%	0.0%	0.0%
	p Verrucomicrobia;c Verrucomicrobiae;o Opitutales;f Opitutaceae;g Opitutus	0.1%	0.1%	0.1%
	p Verrucomicrobia;c Verrucomicrobiae;o Pedosphaerales;f Pedosphaeraceae;Other	1.1%	0.6%	1.5%
	p Verrucomicrobia;c Verrucomicrobiae;o Pedosphaerales;f Pedosphaeraceae;g uncultured-Verrucomicrobia-bacterium	0.0%	0.0%	0.1%
	p Verrucomicrobia;c Verrucomicrobiae;o Pedosphaerales;f Pedosphaeraceae;g uncultured-bacterium	1.1%	0.7%	1.5%
	p Verrucomicrobia;c Verrucomicrobiae;o Pedosphaerales;f Pedosphaeraceae;g uncultured-soil-bacterium	0.3%	0.2%	0.4%
	p Verrucomicrobia;c Verrucomicrobiae;o Pedosphaerales;f Pedosphaeraceae;g uncultured-subdivision-3-bacterium	0.1%	0.0%	0.1%
	p Verrucomicrobia;c Verrucomicrobiae;o Verrucomicrobiales;Other;Other	0.0%	0.0%	0.0%
	p Verrucomicrobia;c Verrucomicrobiae;o Verrucomicrobiales;f Akkermansiaceae;g Akkermansia	0.0%	0.0%	0.0%
	p Verrucomicrobia;c Verrucomicrobiae;o Verrucomicrobiales;f Rubritaleaceae;g Luteolibacter	0.1%	0.1%	0.1%
	p Verrucomicrobia;c Verrucomicrobiae;o Verrucomicrobiales;f Verrucomicrobiaceae;Other	0.0%	0.0%	0.0%
	p Verrucomicrobia;c Verrucomicrobiae;o Verrucomicrobiales;f Verrucomicrobiaceae;g Verrucomicrobium	0.0%	0.0%	0.0%
	p Verrucomicrobia;c Verrucomicrobiae;o Verrucomicrobiales;f Verrucomicrobiaceae;g uncultured	0.0%	0.0%	0.0%
	p WPS-2;Other;Other;Other;Other	0.0%	0.0%	0.0%
	p Zixibacteria;Other;Other;Other;Other	0.0%	0.0%	0.0%
	p Zixibacteria;c uncultured-bacterium;o uncultured-bacterium;f uncultured-bacterium;g uncultured-bacterium	0.0%	0.0%	0.0%

Taxonomy Summary. Current Level:

Legend	Taxonomy	Total %	G1 %	G2 %
	Unclassified;Other;Other;Other;Other;Other	0.5%	0.7%	0.3%
	p Acidobacteria;Other;Other;Other;Other;Other	0.1%	0.2%	0.1%
	p Acidobacteria;c Acidobacteriia;o Acidobacteriales;Other;Other;Other	0.1%	0.0%	0.1%
	p Acidobacteria;c Acidobacteriia;o Acidobacteriales;f Acidobacteriaceae-(Subgroup-1);g uncultured;s uncultured-Acidobacteria-bacterium	0.0%	0.0%	0.0%
	p Acidobacteria;c Acidobacteriia;o Acidobacteriales;f Koribacteraceae;g Candidatus-Koribacter;Other	0.0%	0.0%	0.0%
	p Acidobacteria;c Acidobacteriia;o Acidobacteriales;f Koribacteraceae;g Candidatus-Koribacter;s uncultured-bacterium	0.1%	0.0%	0.1%
	p Acidobacteria;c Acidobacteriia;o Acidobacteriales;f uncultured;Other;Other	0.1%	0.1%	0.1%
	p Acidobacteria;c Acidobacteriia;o Acidobacteriales;f uncultured;g uncultured-Acidobacteria-bacterium;s uncultured-Acidobacteria-bacterium	0.0%	0.0%	0.1%
	p Acidobacteria;c Acidobacteriia;o Acidobacteriales;f uncultured;g uncultured-Acidobacteriaceae-bacterium;s uncultured-Acidobacteriaceae-bacterium	0.0%	0.0%	0.0%
	p Acidobacteria;c Acidobacteriia;o Acidobacteriales;f uncultured;g uncultured-bacterium;s uncultured-bacterium	0.0%	0.0%	0.0%
	p Acidobacteria;c Acidobacteriia;o Solibacterales;f Solibacteraceae-(Subgroup-3);Other;Other	0.0%	0.0%	0.1%
	p Acidobacteria;c Acidobacteriia;o Solibacterales;f Solibacteraceae-(Subgroup-3);g Candidatus-Solibacter;Other	0.9%	0.6%	1.3%

	p Acidobacteria;c Acidobacteriia;o Solibacterales;f Solibacteraceae-(Subgroup-3);g Candidatus-Solibacter;s uncultured-Acidobacteria-bacterium	0.3%	0.3%	0.3%
	p Acidobacteria;c Acidobacteriia;o Solibacterales;f Solibacteraceae-(Subgroup-3);g Candidatus-Solibacter;s uncultured-Acidobacteriales-bacterium	0.2%	0.2%	0.2%
	p Acidobacteria;c Acidobacteriia;o Solibacterales;f Solibacteraceae-(Subgroup-3);g Candidatus-Solibacter;s uncultured-bacterium	0.3%	0.3%	0.4%
	p Acidobacteria;c Acidobacteriia;o Subgroup-12;f uncultured-bacterium;g uncultured-bacterium;s uncultured-bacterium	0.0%	0.0%	0.0%
	p Acidobacteria;c Acidobacteriia;o Subgroup-13;f uncultured-Acidobacteria-bacterium;g uncultured-Acidobacteria-bacterium;s uncultured-Acidobacteria-bacterium	0.0%	0.0%	0.0%
	p Acidobacteria;c Acidobacteriia;o Subgroup-2;Other;Other;Other	0.0%	0.0%	0.0%
	p Acidobacteria;c Acidobacteriia;o Subgroup-2;f uncultured-Acidobacteria-bacterium;g uncultured-Acidobacteria-bacterium;s uncultured-Acidobacteria-bacterium	0.0%	0.0%	0.0%
	p Acidobacteria;c Acidobacteriia;o Subgroup-2;f uncultured-bacterium;g uncultured-bacterium;s uncultured-bacterium	0.1%	0.1%	0.0%
	p Acidobacteria;c Acidobacteriia;o Subgroup-2;f uncultured-forest-soil-bacterium;g uncultured-forest-soil-bacterium;s uncultured-forest-soil-bacterium	0.0%	0.0%	0.0%
	p Acidobacteria;c Acidobacteriia;o Subgroup-2;f uncultured-soil-bacterium;g uncultured-soil-bacterium;s uncultured-soil-bacterium	0.0%	0.0%	0.0%
	p Acidobacteria;c Blastocatellia-(Subgroup-4);Other;Other;Other;Other	2.6%	1.6%	3.6%
	p Acidobacteria;c Blastocatellia-(Subgroup-4);o 11-24;Other;Other;Other	0.1%	0.0%	0.1%
	p Acidobacteria;c Blastocatellia-(Subgroup-4);o 11-24;f uncultured-Acidobacteria-bacterium;g uncultured-Acidobacteria-bacterium;s uncultured-Acidobacteria-bacterium	0.2%	0.2%	0.2%
	p Acidobacteria;c Blastocatellia-(Subgroup-4);o 11-24;f uncultured-Acidobacteriales-bacterium;g uncultured-Acidobacteriales-bacterium;s uncultured-Acidobacteriales-bacterium	0.0%	0.0%	0.0%
	p Acidobacteria;c Blastocatellia-(Subgroup-4);o 11-24;f uncultured-bacterium;g uncultured-bacterium;s uncultured-bacterium	0.1%	0.2%	0.1%
	p Acidobacteria;c Blastocatellia-(Subgroup-4);o Blastocatellales;f Blastocatellaceae;Other;Other	0.2%	0.1%	0.4%
	p Acidobacteria;c Blastocatellia-(Subgroup-4);o Blastocatellales;f Blastocatellaceae;g Blastocatella;Other	0.0%	0.0%	0.0%
	p Acidobacteria;c Blastocatellia-(Subgroup-4);o Blastocatellales;f Blastocatellaceae;g Blastocatella;s uncultured-Acidobacteria-bacterium	0.0%	0.0%	0.0%
	p Acidobacteria;c Blastocatellia-(Subgroup-4);o Blastocatellales;f Blastocatellaceae;g Blastocatella;s uncultured-bacterium	0.1%	0.0%	0.1%
	p Acidobacteria;c Blastocatellia-(Subgroup-4);o Blastocatellales;f Blastocatellaceae;g Stenotrophobacter;Other	0.0%	0.0%	0.1%
	p Acidobacteria;c Blastocatellia-(Subgroup-4);o Blastocatellales;f Blastocatellaceae;g Stenotrophobacter;s uncultured-bacterium	0.0%	0.0%	0.0%
	p Acidobacteria;c Blastocatellia-(Subgroup-4);o Blastocatellales;f Blastocatellaceae;g uncultured;Other	0.0%	0.0%	0.0%
	p Acidobacteria;c Blastocatellia-(Subgroup-4);o Blastocatellales;f Blastocatellaceae;g uncultured;s uncultured-Acidobacteria-bacterium	0.3%	0.3%	0.3%
	p Acidobacteria;c Blastocatellia-(Subgroup-4);o Blastocatellales;f Blastocatellaceae;g uncultured;s uncultured-bacterium	0.0%	0.0%	0.0%
	p Acidobacteria;c Blastocatellia-(Subgroup-4);o DS-100;Other;Other;Other	0.0%	0.0%	0.0%
	p Acidobacteria;c Blastocatellia-(Subgroup-4);o DS-100;f uncultured-Acidobacteria-bacterium;g uncultured-Acidobacteria-bacterium;s uncultured-Acidobacteria-bacterium	0.0%	0.0%	0.0%
	p Acidobacteria;c Blastocatellia-(Subgroup-4);o DS-100;f uncultured-bacterium;g uncultured-bacterium;s uncultured-bacterium	0.0%	0.0%	0.0%
	p Acidobacteria;c Blastocatellia-(Subgroup-4);o Elev-16S-573;Other;Other;Other	5.1%	2.3%	8.0%
	p Acidobacteria;c Blastocatellia-(Subgroup-4);o Elev-16S-573;f uncultured-Acidobacteria-bacterium;g uncultured-Acidobacteria-bacterium;s uncultured-Acidobacteria-bacterium	0.0%	0.0%	0.0%
	p Acidobacteria;c Blastocatellia-(Subgroup-4);o Elev-16S-573;f uncultured-bacterium;g uncultured-bacterium;s uncultured-bacterium	0.7%	0.8%	0.7%
	p Acidobacteria;c Holophagae;o Holophagales;f Holophagaceae;g Geothrix;s uncultured-bacterium	0.0%	0.0%	0.0%
	p Acidobacteria;c Holophagae;o Subgroup-7;Other;Other;Other	0.2%	0.2%	0.2%
	p Acidobacteria;c Holophagae;o Subgroup-7;f uncultured-Acidobacteria-bacterium;g uncultured-Acidobacteria-bacterium;s uncultured-Acidobacteria-bacterium	0.0%	0.0%	0.0%
	p Acidobacteria;c Holophagae;o Subgroup-7;f uncultured-Acidobacteriales-bacterium;g uncultured-Acidobacteriales-bacterium;s uncultured-Acidobacteriales-bacterium	0.0%	0.0%	0.0%
	p Acidobacteria;c Holophagae;o Subgroup-7;f uncultured-Acidobacterium-sp.;g uncultured-Acidobacterium-sp.;s uncultured-Acidobacterium-sp.	0.0%	0.0%	0.0%
	p Acidobacteria;c Holophagae;o Subgroup-7;f uncultured-bacterium;g uncultured-bacterium;s uncultured-bacterium	0.1%	0.2%	0.1%
	p Acidobacteria;c Subgroup-11;Other;Other;Other;Other	0.1%	0.1%	0.1%
	p Acidobacteria;c Subgroup-11;o uncultured-bacterium;f uncultured-bacterium;g uncultured-bacterium;s uncultured-bacterium	0.0%	0.0%	0.0%
	p Acidobacteria;c Subgroup-15;Other;Other;Other;Other	0.0%	0.0%	0.0%
	p Acidobacteria;c Subgroup-15;o uncultured-bacterium;f uncultured-bacterium;g uncultured-bacterium;s uncultured-bacterium	0.0%	0.0%	0.0%
	p Acidobacteria;c Subgroup-17;Other;Other;Other;Other	0.1%	0.1%	0.0%
	p Acidobacteria;c Subgroup-17;o uncultured-Acidobacteria-bacterium;f uncultured-Acidobacteria-bacterium;g uncultured-Acidobacteria-bacterium;s uncultured-Acidobacteria-bacterium	0.0%	0.0%	0.0%
	p Acidobacteria;c Subgroup-17;o uncultured-Acidobacteriales-bacterium;f uncultured-Acidobacteriales-bacterium;g uncultured-Acidobacteriales-bacterium;s uncultured-Acidobacteriales-bacterium	0.0%	0.0%	0.0%
	p Acidobacteria;c Subgroup-17;o uncultured-bacterium;f uncultured-bacterium;g uncultured-bacterium;s uncultured-bacterium	0.1%	0.1%	0.0%
	p Acidobacteria;c Subgroup-18;Other;Other;Other;Other	0.0%	0.0%	0.0%
	p Acidobacteria;c Subgroup-18;o uncultured-bacterium;f uncultured-bacterium;g uncultured-bacterium;s uncultured-bacterium	0.0%	0.0%	0.0%

	p Acidobacteria;c Subgroup-20;o uncultured-bacterium;f uncultured-bacterium;g uncultured-bacterium;s uncultured-bacterium	0.0%	0.0%	0.0%
	p Acidobacteria;c Subgroup-22;Other;Other;Other;Other	0.1%	0.1%	0.1%
	p Acidobacteria;c Subgroup-22;o uncultured-Acidobacterium-sp.;f uncultured-Acidobacterium-sp.;g uncultured-Acidobacterium-sp.;s uncultured-Acidobacterium-sp.	0.0%	0.1%	0.0%
	p Acidobacteria;c Subgroup-22;o uncultured-bacterium;f uncultured-bacterium;g uncultured-bacterium;s uncultured-bacterium	0.1%	0.2%	0.1%
	p Acidobacteria;c Subgroup-25;Other;Other;Other;Other	0.0%	0.1%	0.0%
	p Acidobacteria;c Subgroup-25;o uncultured-Acidobacteria-bacterium;f uncultured-Acidobacteria-bacterium;g uncultured-Acidobacteria-bacterium;s uncultured-Acidobacteria-bacterium	0.0%	0.0%	0.0%
	p Acidobacteria;c Subgroup-25;o uncultured-bacterium;f uncultured-bacterium;g uncultured-bacterium;s uncultured-bacterium	0.0%	0.0%	0.0%
	p Acidobacteria;c Subgroup-25;o uncultured-soil-bacterium;f uncultured-soil-bacterium;g uncultured-soil-bacterium;s uncultured-soil-bacterium	0.0%	0.0%	0.0%
	p Acidobacteria;c Subgroup-5;Other;Other;Other;Other	0.0%	0.0%	0.0%
	p Acidobacteria;c Subgroup-5;o uncultured-Acidobacteria-bacterium;f uncultured-Acidobacteria-bacterium;g uncultured-Acidobacteria-bacterium;s uncultured-Acidobacteria-bacterium	0.2%	0.2%	0.2%
	p Acidobacteria;c Subgroup-5;o uncultured-Acidobacterium-sp.;f uncultured-Acidobacterium-sp.;g uncultured-Acidobacterium-sp.;s uncultured-Acidobacterium-sp.	0.0%	0.0%	0.0%
	p Acidobacteria;c Subgroup-5;o uncultured-bacterium;f uncultured-bacterium;g uncultured-bacterium;s uncultured-bacterium	0.2%	0.2%	0.2%
	p Acidobacteria;c Subgroup-6;Other;Other;Other;Other	5.4%	6.2%	4.6%
	p Acidobacteria;c Subgroup-6;o Acidobacteria-bacterium-WX90;f Acidobacteria-bacterium-WX90;g Acidobacteria-bacterium-WX90;s Acidobacteria-bacterium-WX90	0.0%	0.0%	0.0%
	p Acidobacteria;c Subgroup-6;o Unknown-Order;f Unknown-Family;g Luteitalea;Other	0.0%	0.0%	0.0%
	p Acidobacteria;c Subgroup-6;o Unknown-Order;f Unknown-Family;g Luteitalea;s uncultured-bacterium	0.0%	0.0%	0.0%
	p Acidobacteria;c Subgroup-6;o uncultured-Acidobacteria-bacterium;f uncultured-Acidobacteria-bacterium;g uncultured-Acidobacteria-bacterium;s uncultured-Acidobacteria-bacterium	0.2%	0.2%	0.2%
	p Acidobacteria;c Subgroup-6;o uncultured-Acidobacteriaceae-bacterium;f uncultured-Acidobacteriaceae-bacterium;g uncultured-Acidobacteriaceae-bacterium;s uncultured-Acidobacteriaceae-bacterium	0.0%	0.0%	0.0%
	p Acidobacteria;c Subgroup-6;o uncultured-Acidobacteriales-bacterium;f uncultured-Acidobacteriales-bacterium;g uncultured-Acidobacteriales-bacterium;s uncultured-Acidobacteriales-bacterium	0.1%	0.1%	0.1%
	p Acidobacteria;c Subgroup-6;o uncultured-Acidobacterium-sp.;f uncultured-Acidobacterium-sp.;g uncultured-Acidobacterium-sp.;s uncultured-Acidobacterium-sp.	0.0%	0.0%	0.0%
	p Acidobacteria;c Subgroup-6;o uncultured-Holophagae-bacterium;f uncultured-Holophagae-bacterium;g uncultured-Holophagae-bacterium;s uncultured-Holophagae-bacterium	0.0%	0.0%	0.0%
	p Acidobacteria;c Subgroup-6;o uncultured-bacterium;f uncultured-bacterium;g uncultured-bacterium;s uncultured-bacterium	0.8%	0.9%	0.8%
	p Acidobacteria;c Subgroup-6;o uncultured-beta-proteobacterium;f uncultured-beta-proteobacterium;g uncultured-beta-proteobacterium;s uncultured-beta-proteobacterium	0.0%	0.0%	0.0%
	p Acidobacteria;c Subgroup-6;o uncultured-proteobacterium;f uncultured-proteobacterium;g uncultured-proteobacterium;s uncultured-proteobacterium	0.0%	0.0%	0.0%
	p Acidobacteria;c Subgroup-9;Other;Other;Other;Other	0.0%	0.0%	0.0%
	p Actinobacteria;Other;Other;Other;Other;Other	0.0%	0.0%	0.1%
	p Actinobacteria;c 0319-7L14;Other;Other;Other;Other	0.0%	0.0%	0.1%
	p Actinobacteria;c 0319-7L14;o uncultured-bacterium;f uncultured-bacterium;g uncultured-bacterium;s uncultured-bacterium	0.0%	0.0%	0.0%
	p Actinobacteria;c Acidimicrobiia;Other;Other;Other;Other	0.3%	0.3%	0.3%
	p Actinobacteria;c Acidimicrobiia;o Acidimicrobiales;f Acidimicrobiaceae;g uncultured;s uncultured-bacterium	0.0%	0.0%	0.0%
	p Actinobacteria;c Acidimicrobiia;o Actinomarinales;f uncultured;Other;Other	0.1%	0.1%	0.0%
	p Actinobacteria;c Acidimicrobiia;o Actinomarinales;f uncultured;g uncultured-Actinomycetales-bacterium;s uncultured-Actinomycetales-bacterium	0.6%	1.2%	0.1%
	p Actinobacteria;c Acidimicrobiia;o Actinomarinales;f uncultured;g uncultured-actinobacterium;s uncultured-actinobacterium	0.0%	0.0%	0.0%
	p Actinobacteria;c Acidimicrobiia;o IMCC26256;Other;Other;Other	0.4%	0.4%	0.3%
	p Actinobacteria;c Acidimicrobiia;o IMCC26256;f uncultured-Acidimicrobiales-bacterium;g uncultured-Acidimicrobiales-bacterium;s uncultured-Acidimicrobiales-bacterium	0.0%	0.0%	0.0%
	p Actinobacteria;c Acidimicrobiia;o IMCC26256;f uncultured-Acidimicrobidae-bacterium;g uncultured-Acidimicrobidae-bacterium;s uncultured-Acidimicrobidae-bacterium	0.0%	0.0%	0.0%
	p Actinobacteria;c Acidimicrobiia;o IMCC26256;f uncultured-actinobacterium;g uncultured-actinobacterium;s uncultured-actinobacterium	0.0%	0.0%	0.1%
	p Actinobacteria;c Acidimicrobiia;o IMCC26256;f uncultured-bacterium;g uncultured-bacterium;s uncultured-bacterium	0.0%	0.0%	0.0%
	p Actinobacteria;c Acidimicrobiia;o IMCC26256;f uncultured-organism;g uncultured-organism;s uncultured-organism	0.0%	0.0%	0.0%
	p Actinobacteria;c Acidimicrobiia;o Microtrichales;Other;Other;Other	0.1%	0.1%	0.1%
	p Actinobacteria;c Acidimicrobiia;o Microtrichales;f Iamiaceae;g Iamia;Other	0.1%	0.1%	0.1%
	p Actinobacteria;c Acidimicrobiia;o Microtrichales;f Iamiaceae;g Iamia;s uncultured-bacterium	0.1%	0.1%	0.1%
	p Actinobacteria;c Acidimicrobiia;o Microtrichales;f Ilumatobacteraceae;Other;Other	0.0%	0.0%	0.0%
	p Actinobacteria;c Acidimicrobiia;o Microtrichales;f Ilumatobacteraceae;g Ilumatobacter;s uncultured-bacterium	0.1%	0.1%	0.1%
	p Actinobacteria;c Acidimicrobiia;o Microtrichales;f Ilumatobacteraceae;g uncultured;Other	0.0%	0.0%	0.0%
	p Actinobacteria;c Acidimicrobiia;o Microtrichales;f Ilumatobacteraceae;g uncultured;s uncultured-bacterium	0.1%	0.1%	0.1%
	p Actinobacteria;c Acidimicrobiia;o Microtrichales;f Ilumatobacteraceae;g uncultured-bacterium;s uncultured-bacterium	0.0%	0.0%	0.0%

	p Actinobacteria;c Acidimicrobiia;o Microtrichales;f Microtrichaceae;g uncultured;s uncultured-bacterium	0.0%	0.0%	0.0%
	p Actinobacteria;c Acidimicrobiia;o Microtrichales;f uncultured;Other;Other	0.1%	0.1%	0.1%
	p Actinobacteria;c Acidimicrobiia;o Microtrichales;f uncultured;g uncultured-bacterium;s uncultured-bacterium	0.1%	0.1%	0.1%
	p Actinobacteria;c Acidimicrobiia;o uncultured;Other;Other;Other	0.3%	0.2%	0.5%
	p Actinobacteria;c Acidimicrobiia;o uncultured;f uncultured-Acidimicrobidae-bacterium;g uncultured-Acidimicrobidae-bacterium;s uncultured-Acidimicrobidae-bacterium	0.0%	0.0%	0.1%
	p Actinobacteria;c Acidimicrobiia;o uncultured;f uncultured-actinobacterium;g uncultured-actinobacterium;s uncultured-actinobacterium	0.0%	0.0%	0.0%
	p Actinobacteria;c Acidimicrobiia;o uncultured;f uncultured-bacterium;g uncultured-bacterium;s uncultured-bacterium	0.1%	0.0%	0.1%
	p Actinobacteria;c Acidimicrobiia;o uncultured;f uncultured-soil-bacterium;g uncultured-soil-bacterium;s uncultured-soil-bacterium	0.0%	0.0%	0.1%
	p Actinobacteria;c Actinobacteria;Other;Other;Other;Other	0.0%	0.0%	0.0%
	p Actinobacteria;c Actinobacteria;o Corynebacteriales;f Mycobacteriaceae;g Mycobacterium;Other	0.0%	0.0%	0.1%
	p Actinobacteria;c Actinobacteria;o Corynebacteriales;f Mycobacteriaceae;g Mycobacterium;s uncultured-bacterium	0.3%	0.1%	0.4%
	p Actinobacteria;c Actinobacteria;o Corynebacteriales;f Nocardiaceae;g Nocardia;Other	0.0%	0.0%	0.0%
	p Actinobacteria;c Actinobacteria;o Corynebacteriales;f Nocardiaceae;g Nocardia;s Nocardia-niigatensis	0.0%	0.0%	0.0%
	p Actinobacteria;c Actinobacteria;o Corynebacteriales;f Nocardiaceae;g Nocardia;s Nocardia-pseudovaccinii-NBRC-100343	0.9%	0.4%	1.3%
	p Actinobacteria;c Actinobacteria;o Corynebacteriales;f Nocardiaceae;g Nocardia;s Nocardia-sp.-HBUD30303	0.0%	0.0%	0.0%
	p Actinobacteria;c Actinobacteria;o Corynebacteriales;f Nocardiaceae;g Nocardia;s Nocardia-sp.-HBUM-79084	0.0%	0.0%	0.0%
	p Actinobacteria;c Actinobacteria;o Corynebacteriales;f Nocardiaceae;g Rhodococcus;Other	0.2%	0.1%	0.3%
	p Actinobacteria;c Actinobacteria;o Corynebacteriales;f Nocardiaceae;g Rhodococcus;s Rhodococcus-ruber	0.0%	0.0%	0.0%
	p Actinobacteria;c Actinobacteria;o Frankiales;f Cryptosporangiaceae;g Cryptosporangium;s Cryptosporangium-aurantiacum	0.0%	0.0%	0.0%
	p Actinobacteria;c Actinobacteria;o Frankiales;f Cryptosporangiaceae;g Fodinicola;s uncultured-bacterium	0.0%	0.0%	0.1%
	p Actinobacteria;c Actinobacteria;o Frankiales;f Frankiaceae;g Frankia;s Frankia-sp.	0.0%	0.0%	0.0%
	p Actinobacteria;c Actinobacteria;o Frankiales;f Geodermatophilaceae;Other;Other	0.0%	0.0%	0.0%
	p Actinobacteria;c Actinobacteria;o Frankiales;f Geodermatophilaceae;g Blastococcus;s uncultured-bacterium	0.0%	0.0%	0.0%
	p Actinobacteria;c Actinobacteria;o Frankiales;f Geodermatophilaceae;g Geodermatophilus;Other	0.0%	0.0%	0.0%
	p Actinobacteria;c Actinobacteria;o Frankiales;f Nakamurellaceae;g Nakamurella;Other	0.0%	0.0%	0.0%
	p Actinobacteria;c Actinobacteria;o Frankiales;f Sporichthyaceae;g Sporichthya;s Sporichthya-polymorpha-DSM-43042	0.0%	0.0%	0.0%
	p Actinobacteria;c Actinobacteria;o Frankiales;f Sporichthyaceae;g uncultured;s uncultured-bacterium	0.0%	0.0%	0.0%
	p Actinobacteria;c Actinobacteria;o Frankiales;f uncultured;Other;Other	0.0%	0.0%	0.0%
	p Actinobacteria;c Actinobacteria;o Frankiales;f uncultured;g uncultured-bacterium;s uncultured-bacterium	0.0%	0.0%	0.0%
	p Actinobacteria;c Actinobacteria;o Glycomycetales;f Glycomycetaceae;g Glycomyces;Other	1.0%	1.9%	0.1%
	p Actinobacteria;c Actinobacteria;o Glycomycetales;f Glycomycetaceae;g uncultured;s uncultured-bacterium	0.0%	0.0%	0.0%
	p Actinobacteria;c Actinobacteria;o Kineosporiales;f Kineosporiaceae;Other;Other	0.0%	0.0%	0.0%
	p Actinobacteria;c Actinobacteria;o Micrococcales;Other;Other;Other	0.0%	0.0%	0.0%
	p Actinobacteria;c Actinobacteria;o Micrococcales;f Cellulomonadaceae;g Cellulomonas;Other	0.0%	0.0%	0.0%
	p Actinobacteria;c Actinobacteria;o Micrococcales;f Demequinaceae;Other;Other	0.0%	0.0%	0.0%
	p Actinobacteria;c Actinobacteria;o Micrococcales;f Intrasporangiaceae;Other;Other	0.1%	0.1%	0.1%
	p Actinobacteria;c Actinobacteria;o Micrococcales;f Microbacteriaceae;Other;Other	0.0%	0.0%	0.0%
	p Actinobacteria;c Actinobacteria;o Micrococcales;f Microbacteriaceae;g Agromyces;Other	0.1%	0.1%	0.0%
	p Actinobacteria;c Actinobacteria;o Micrococcales;f Microbacteriaceae;g Agromyces;s uncultured-bacterium	0.0%	0.0%	0.0%
	p Actinobacteria;c Actinobacteria;o Micrococcales;f Microbacteriaceae;g Leifsonia;Other	0.0%	0.0%	0.0%
	p Actinobacteria;c Actinobacteria;o Micrococcales;f Microbacteriaceae;g Microbacterium;Other	0.0%	0.0%	0.0%
	p Actinobacteria;c Actinobacteria;o Micrococcales;f Micrococcaceae;g Arthrobacter;Other	0.0%	0.0%	0.0%
	p Actinobacteria;c Actinobacteria;o Micrococcales;f Micrococcaceae;g Arthrobacter;s Arthrobacter-crystallopoietes	0.0%	0.0%	0.0%
	p Actinobacteria;c Actinobacteria;o Micrococcales;f Micrococcaceae;g Pseudarthrobacter;Other	0.1%	0.0%	0.2%
	p Actinobacteria;c Actinobacteria;o Micrococcales;f Promicromonosporaceae;g Cellulosimicrobium;s Cellulosimicrobium-cellulans	0.0%	0.0%	0.0%
	p Actinobacteria;c Actinobacteria;o Micrococcales;f Promicromonosporaceae;g Promicromonospora;Other	0.1%	0.3%	0.0%
	p Actinobacteria;c Actinobacteria;o Micrococcales;f Promicromonosporaceae;g Promicromonospora;s Cellulomonas-sp.-ZYG2	0.1%	0.2%	0.0%
	p Actinobacteria;c Actinobacteria;o Micromonosporales;f Micromonosporaceae;Other;Other	0.2%	0.2%	0.3%
	p Actinobacteria;c Actinobacteria;o Micromonosporales;f Micromonosporaceae;g Actinoplanes;s uncultured-bacterium	0.0%	0.0%	0.0%
	p Actinobacteria;c Actinobacteria;o Micromonosporales;f Micromonosporaceae;g Dactylosporangium;Other	0.0%	0.0%	0.0%
	p Actinobacteria;c Actinobacteria;o Micromonosporales;f Micromonosporaceae;g Krasilnikovia;s uncultured-bacterium	0.0%	0.0%	0.0%
	p Actinobacteria;c Actinobacteria;o Micromonosporales;f Micromonosporaceae;g Luedemannella;Other	0.0%	0.0%	0.0%
	p Actinobacteria;c Actinobacteria;o Micromonosporales;f Micromonosporaceae;g Luedemannella;s uncultured-bacterium	0.0%	0.0%	0.0%
	p Actinobacteria;c Actinobacteria;o Micromonosporales;f Micromonosporaceae;g Micromonospora;Other	0.2%	0.2%	0.1%
	p Actinobacteria;c Actinobacteria;o Micromonosporales;f Micromonosporaceae;g Phytomonospora;s Phytomonospora-endophytica	0.0%	0.0%	0.0%
	p Actinobacteria;c Actinobacteria;o Micromonosporales;f Micromonosporaceae;g Stackebrandtia;Other	0.0%	0.0%	0.0%
	p Actinobacteria;c Actinobacteria;o Micromonosporales;f Micromonosporaceae;g uncultured;Other	0.0%	0.0%	0.0%
	p Actinobacteria;c Actinobacteria;o Micromonosporales;f Micromonosporaceae;g uncultured;s uncultured-bacterium	0.1%	0.0%	0.1%
	p Actinobacteria;c Actinobacteria;o Propionibacteriales;f Nocardioidaceae;Other;Other	0.3%	0.2%	0.3%
	p Actinobacteria;c Actinobacteria;o Propionibacteriales;f Nocardioidaceae;g Actinopolymorpha;Other	0.0%	0.0%	0.0%
	p Actinobacteria;c Actinobacteria;o Propionibacteriales;f Nocardioidaceae;g Aeromicrobium;s uncultured-bacterium	0.0%	0.1%	0.0%
	p Actinobacteria;c Actinobacteria;o Propionibacteriales;f Nocardioidaceae;g Kribbella;s uncultured-bacterium	0.7%	0.9%	0.5%

	p Actinobacteria;c Actinobacteria;o Propionibacteriales;f Nocardioidaceae;g Marmoricola;s uncultured-bacterium	0.0%	0.0%	0.0%
	p Actinobacteria;c Actinobacteria;o Propionibacteriales;f Nocardioidaceae;g Nocardioides;Other	0.5%	0.3%	0.6%
	p Actinobacteria;c Actinobacteria;o Propionibacteriales;f Nocardioidaceae;g Nocardioides;s uncultured-Nocardioides-sp.	0.0%	0.0%	0.1%
	p Actinobacteria;c Actinobacteria;o Propionibacteriales;f Nocardioidaceae;g Nocardioides;s uncultured-actinobacterium	0.0%	0.0%	0.0%
	p Actinobacteria;c Actinobacteria;o Propionibacteriales;f Nocardioidaceae;g Nocardioides;s uncultured-bacterium	0.0%	0.0%	0.0%
	p Actinobacteria;c Actinobacteria;o Propionibacteriales;f Nocardioidaceae;g Nocardioides;s uncultured-organism	0.0%	0.0%	0.0%
	p Actinobacteria;c Actinobacteria;o Propionibacteriales;f Propionibacteriaceae;g Microlunatus;s uncultured-bacterium	0.0%	0.0%	0.0%
	p Actinobacteria;c Actinobacteria;o Propionibacteriales;f Propionibacteriaceae;g uncultured;s uncultured-bacterium	0.0%	0.0%	0.0%
	p Actinobacteria;c Actinobacteria;o Pseudonocardiales;f Pseudonocardiaceae;Other;Other	0.1%	0.0%	0.1%
	p Actinobacteria;c Actinobacteria;o Pseudonocardiales;f Pseudonocardiaceae;g Actinophytocola;Other	0.0%	0.0%	0.0%
	p Actinobacteria;c Actinobacteria;o Pseudonocardiales;f Pseudonocardiaceae;g Actinophytocola;s actinobacterium-ND2ZKDS4	0.0%	0.0%	0.0%
	p Actinobacteria;c Actinobacteria;o Pseudonocardiales;f Pseudonocardiaceae;g Allokutzneria;s Allokutzneria-multivorans	0.0%	0.0%	0.0%
	p Actinobacteria;c Actinobacteria;o Pseudonocardiales;f Pseudonocardiaceae;g Amycolatopsis;s uncultured-bacterium	0.0%	0.0%	0.0%
	p Actinobacteria;c Actinobacteria;o Pseudonocardiales;f Pseudonocardiaceae;g Crossiella;s uncultured-bacterium	0.0%	0.0%	0.1%
	p Actinobacteria;c Actinobacteria;o Pseudonocardiales;f Pseudonocardiaceae;g Pseudonocardia;Other	0.1%	0.0%	0.1%
	p Actinobacteria;c Actinobacteria;o Pseudonocardiales;f Pseudonocardiaceae;g Pseudonocardia;s uncultured-bacterium	0.0%	0.0%	0.0%
	p Actinobacteria;c Actinobacteria;o Streptomycetales;f Streptomycetaceae;Other;Other	0.0%	0.0%	0.0%
	p Actinobacteria;c Actinobacteria;o Streptomycetales;f Streptomycetaceae;g Streptomyces;Other	7.6%	8.0%	7.3%
	p Actinobacteria;c Actinobacteria;o Streptosporangiales;f Streptosporangiaceae;Other;Other	0.0%	0.0%	0.0%
	p Actinobacteria;c Actinobacteria;o Streptosporangiales;f Streptosporangiaceae;g Nonomuraea;s uncultured-bacterium	0.1%	0.2%	0.0%
	p Actinobacteria;c Actinobacteria;o Streptosporangiales;f Streptosporangiaceae;g uncultured;s uncultured-bacterium	0.0%	0.0%	0.0%
	p Actinobacteria;c Actinobacteria;o Streptosporangiales;f Thermomonosporaceae;Other;Other	0.0%	0.0%	0.0%
	p Actinobacteria;c MB-A2-108;Other;Other;Other;Other	0.1%	0.1%	0.1%
	p Actinobacteria;c MB-A2-108;o uncultured-actinobacterium;f uncultured-actinobacterium;g uncultured-actinobacterium;s uncultured-actinobacterium	0.0%	0.0%	0.0%
	p Actinobacteria;c MB-A2-108;o uncultured-bacterium;f uncultured-bacterium;g uncultured-bacterium;s uncultured-bacterium	0.6%	0.7%	0.4%
	p Actinobacteria;c MB-A2-108;o uncultured-bacterium-contig00016;f uncultured-bacterium-contig00016;g uncultured-bacterium-contig00016;s uncultured-bacterium-contig00016	0.0%	0.0%	0.0%
	p Actinobacteria;c Nitriliruptoria;o Euzebyales;f Euzebyaceae;g uncultured;Other	0.0%	0.0%	0.0%
	p Actinobacteria;c Rubrobacteria;o Rubrobacterales;f Rubrobacteriaceae;g Rubrobacter;Other	0.0%	0.0%	0.0%
	p Actinobacteria;c Rubrobacteria;o Rubrobacterales;f Rubrobacteriaceae;g Rubrobacter;s uncultured-Actinomycetales-bacterium	0.1%	0.0%	0.1%
	p Actinobacteria;c Rubrobacteria;o Rubrobacterales;f Rubrobacteriaceae;g Rubrobacter;s uncultured-bacterium	0.0%	0.0%	0.0%
	p Actinobacteria;c Thermoleophilia;Other;Other;Other;Other	0.1%	0.1%	0.1%
	p Actinobacteria;c Thermoleophilia;o Gaiellales;Other;Other;Other	0.0%	0.0%	0.0%
	p Actinobacteria;c Thermoleophilia;o Gaiellales;f uncultured;Other;Other	0.2%	0.2%	0.1%
	p Actinobacteria;c Thermoleophilia;o Gaiellales;f uncultured;g uncultured-Gaiella-sp.;s uncultured-Gaiella-sp.	0.1%	0.1%	0.1%
	p Actinobacteria;c Thermoleophilia;o Gaiellales;f uncultured;g uncultured-Rubrobacteria-bacterium;s uncultured-Rubrobacteria-bacterium	0.0%	0.0%	0.0%
	p Actinobacteria;c Thermoleophilia;o Gaiellales;f uncultured;g uncultured-actinobacterium;s uncultured-actinobacterium	0.0%	0.0%	0.0%
	p Actinobacteria;c Thermoleophilia;o Gaiellales;f uncultured;g uncultured-bacterium;s uncultured-bacterium	0.4%	0.4%	0.3%
	p Actinobacteria;c Thermoleophilia;o Solirubrobacterales;f 67-14;Other;Other	0.0%	0.0%	0.0%
	p Actinobacteria;c Thermoleophilia;o Solirubrobacterales;f 67-14;g uncultured-actinobacterium;s uncultured-actinobacterium	0.0%	0.0%	0.0%
	p Actinobacteria;c Thermoleophilia;o Solirubrobacterales;f 67-14;g uncultured-bacterium;s uncultured-bacterium	0.3%	0.3%	0.2%
	p Actinobacteria;c Thermoleophilia;o Solirubrobacterales;f Solirubrobacteraceae;g Conexibacter;s uncultured-bacterium	0.0%	0.0%	0.0%
	p Actinobacteria;c Thermoleophilia;o Solirubrobacterales;f Solirubrobacteraceae;g Solirubrobacter;Other	0.0%	0.0%	0.0%
	p Actinobacteria;c Thermoleophilia;o Solirubrobacterales;f Solirubrobacteraceae;g Solirubrobacter;s uncultured-bacterium	0.2%	0.2%	0.2%
	p Actinobacteria;c Thermoleophilia;o uncultured;f uncultured-bacterium;g uncultured-bacterium;s uncultured-bacterium	0.0%	0.0%	0.0%
	p Armatimonadetes;c Fimbriimonadia;o Fimbriimonadales;f Fimbriimonadaceae;Other;Other	0.0%	0.0%	0.0%
	p Armatimonadetes;c Fimbriimonadia;o Fimbriimonadales;f Fimbriimonadaceae;g uncultured-bacterium;s uncultured-bacterium	0.0%	0.0%	0.0%
	p BRC1;Other;Other;Other;Other;Other	0.0%	0.0%	0.0%
	p BRC1;c uncultured-bacterium;o uncultured-bacterium;f uncultured-bacterium;g uncultured-bacterium;s uncultured-bacterium	0.0%	0.0%	0.0%
	p Bacteroidetes;c Bacteroidia;Other;Other;Other;Other	0.0%	0.0%	0.0%
	p Bacteroidetes;c Bacteroidia;o Bacteroidetes-VC2.1-Bac22;Other;Other;Other	0.0%	0.0%	0.0%
	p Bacteroidetes;c Bacteroidia;o Chitinophagales;Other;Other;Other	0.0%	0.0%	0.0%
	p Bacteroidetes;c Bacteroidia;o Chitinophagales;f 37-13;Other;Other	0.0%	0.0%	0.0%
	p Bacteroidetes;c Bacteroidia;o Chitinophagales;f 37-13;g uncultured-bacterium;s uncultured-bacterium	0.0%	0.1%	0.0%
	p Bacteroidetes;c Bacteroidia;o Chitinophagales;f Chitinophagaceae;Other;Other	0.4%	0.4%	0.5%
	p Bacteroidetes;c Bacteroidia;o Chitinophagales;f Chitinophagaceae;g Chitinophaga;Other	2.3%	4.6%	0.0%
	p Bacteroidetes;c Bacteroidia;o Chitinophagales;f Chitinophagaceae;g Chitinophaga;s Chitinophaga-rupis	0.0%	0.0%	0.0%
	p Bacteroidetes;c Bacteroidia;o Chitinophagales;f Chitinophagaceae;g Chitinophaga;s Chitinophaga-sp.-A5I53	0.0%	0.0%	0.0%
	p Bacteroidetes;c Bacteroidia;o Chitinophagales;f Chitinophagaceae;g Chitinophaga;s uncultured-Flexibacter-sp.	0.0%	0.0%	0.0%
	p Bacteroidetes;c Bacteroidia;o Chitinophagales;f Chitinophagaceae;g Chitinophaga;s uncultured-bacterium	0.1%	0.1%	0.0%
	p Bacteroidetes;c Bacteroidia;o Chitinophagales;f Chitinophagaceae;g Dinghuibacter;s uncultured-bacterium	0.0%	0.0%	0.0%
	p Bacteroidetes;c Bacteroidia;o Chitinophagales;f Chitinophagaceae;g Ferruginibacter;Other	0.0%	0.0%	0.0%

p Bacteroidetes;c Bacteroidia;o Chitinophagales;f Chitinophagaceae;g Filimonas;s bacterium	0.0%	0.0%	0.0%
p Bacteroidetes;c Bacteroidia;o Chitinophagales;f Chitinophagaceae;g Flavihumibacter;s Flavihumibacter-petaseus-NBRC-106054	0.0%	0.0%	0.0%
p Bacteroidetes;c Bacteroidia;o Chitinophagales;f Chitinophagaceae;g Flavihumibacter;s uncultured-bacterium	0.3%	0.5%	0.0%
p Bacteroidetes;c Bacteroidia;o Chitinophagales;f Chitinophagaceae;g Flavisolibacter;Other	0.1%	0.1%	0.2%
p Bacteroidetes;c Bacteroidia;o Chitinophagales;f Chitinophagaceae;g Flavisolibacter;s Flavisolibacter-sp.-LCS9	0.0%	0.0%	0.0%
p Bacteroidetes;c Bacteroidia;o Chitinophagales;f Chitinophagaceae;g Flavisolibacter;s uncultured-Bacteroidetes-bacterium	0.0%	0.0%	0.0%
p Bacteroidetes;c Bacteroidia;o Chitinophagales;f Chitinophagaceae;g Flavisolibacter;s uncultured-Flavisolibacter-sp.	0.0%	0.0%	0.0%
p Bacteroidetes;c Bacteroidia;o Chitinophagales;f Chitinophagaceae;g Flavisolibacter;s uncultured-bacterium	0.2%	0.2%	0.3%
p Bacteroidetes;c Bacteroidia;o Chitinophagales;f Chitinophagaceae;g Flavitalea;s uncultured-bacterium	0.2%	0.3%	0.1%
p Bacteroidetes;c Bacteroidia;o Chitinophagales;f Chitinophagaceae;g Niabella;Other	0.0%	0.0%	0.0%
p Bacteroidetes;c Bacteroidia;o Chitinophagales;f Chitinophagaceae;g Niabella;s Niabella-sp.-UI6	0.0%	0.0%	0.0%
p Bacteroidetes;c Bacteroidia;o Chitinophagales;f Chitinophagaceae;g Niabella;s uncultured-bacterium	0.0%	0.0%	0.0%
p Bacteroidetes;c Bacteroidia;o Chitinophagales;f Chitinophagaceae;g Niastella;Other	0.2%	0.2%	0.1%
p Bacteroidetes;c Bacteroidia;o Chitinophagales;f Chitinophagaceae;g Niastella;s uncultured-Niastella-sp.	0.0%	0.0%	0.0%
p Bacteroidetes;c Bacteroidia;o Chitinophagales;f Chitinophagaceae;g Niastella;s uncultured-bacterium	0.6%	0.9%	0.3%
p Bacteroidetes;c Bacteroidia;o Chitinophagales;f Chitinophagaceae;g Niveitalea;s Niveitalea-solisilvae	0.0%	0.1%	0.0%
p Bacteroidetes;c Bacteroidia;o Chitinophagales;f Chitinophagaceae;g Parafilimonas;Other	0.0%	0.0%	0.0%
p Bacteroidetes;c Bacteroidia;o Chitinophagales;f Chitinophagaceae;g Parafilimonas;s uncultured-bacterium	0.1%	0.1%	0.2%
p Bacteroidetes;c Bacteroidia;o Chitinophagales;f Chitinophagaceae;g Terrimonas;Other	0.2%	0.3%	0.1%
p Bacteroidetes;c Bacteroidia;o Chitinophagales;f Chitinophagaceae;g Terrimonas;s uncultured-Bacteroidetes-bacterium	0.0%	0.1%	0.0%
p Bacteroidetes;c Bacteroidia;o Chitinophagales;f Chitinophagaceae;g Terrimonas;s uncultured-bacterium	1.1%	1.9%	0.3%
p Bacteroidetes;c Bacteroidia;o Chitinophagales;f Chitinophagaceae;g uncultured;Other	1.5%	0.9%	2.2%
p Bacteroidetes;c Bacteroidia;o Chitinophagales;f Chitinophagaceae;g uncultured;s bacterium	0.0%	0.0%	0.0%
p Bacteroidetes;c Bacteroidia;o Chitinophagales;f Chitinophagaceae;g uncultured;s estrogen-degrading-bacterium-KC2	0.0%	0.0%	0.0%
p Bacteroidetes;c Bacteroidia;o Chitinophagales;f Chitinophagaceae;g uncultured;s uncultured-Bacteroidetes-bacterium	0.1%	0.1%	0.1%
p Bacteroidetes;c Bacteroidia;o Chitinophagales;f Chitinophagaceae;g uncultured;s uncultured-Chitinophagaceae-bacterium	0.1%	0.1%	0.1%
p Bacteroidetes;c Bacteroidia;o Chitinophagales;f Chitinophagaceae;g uncultured;s uncultured-bacterium	0.1%	0.1%	0.2%
p Bacteroidetes;c Bacteroidia;o Chitinophagales;f Chitinophagaceae;g uncultured;s uncultured-soil-bacterium	0.0%	0.1%	0.0%
p Bacteroidetes;c Bacteroidia;o Chitinophagales;f Saprospiraceae;g uncultured;Other	0.0%	0.0%	0.0%
p Bacteroidetes;c Bacteroidia;o Chitinophagales;f Saprospiraceae;g uncultured;s uncultured-Bacteroidetes-bacterium	0.0%	0.0%	0.0%
p Bacteroidetes;c Bacteroidia;o Chitinophagales;f Saprospiraceae;g uncultured;s uncultured-bacterium	0.2%	0.2%	0.2%
p Bacteroidetes;c Bacteroidia;o Chitinophagales;f uncultured;g uncultured-bacterium;s uncultured-bacterium	0.0%	0.0%	0.0%
p Bacteroidetes;c Bacteroidia;o Cytophagales;f Cytophagaceae;g Sporocytophaga;s uncultured-bacterium	0.0%	0.0%	0.0%
p Bacteroidetes;c Bacteroidia;o Cytophagales;f Hymenobacteraceae;g Adhaeribacter;Other	0.0%	0.0%	0.0%
p Bacteroidetes;c Bacteroidia;o Cytophagales;f Hymenobacteraceae;g Adhaeribacter;s uncultured-bacterium	0.0%	0.0%	0.0%
p Bacteroidetes;c Bacteroidia;o Cytophagales;f Hymenobacteraceae;g Adhaeribacter;s uncultured-soil-bacterium	0.1%	0.0%	0.1%
p Bacteroidetes;c Bacteroidia;o Cytophagales;f Microscillaceae;Other;Other	0.3%	0.1%	0.4%
p Bacteroidetes;c Bacteroidia;o Cytophagales;f Microscillaceae;g Ohtaekwangia;Other	0.1%	0.0%	0.2%
p Bacteroidetes;c Bacteroidia;o Cytophagales;f Microscillaceae;g Ohtaekwangia;s Globodera-pallida	0.0%	0.0%	0.0%
p Bacteroidetes;c Bacteroidia;o Cytophagales;f Microscillaceae;g Ohtaekwangia;s uncultured-bacterium	0.9%	1.3%	0.5%
p Bacteroidetes;c Bacteroidia;o Cytophagales;f Microscillaceae;g uncultured;Other	0.4%	0.3%	0.6%
p Bacteroidetes;c Bacteroidia;o Cytophagales;f Microscillaceae;g uncultured;s uncultured-Flexibacter-sp.	0.0%	0.0%	0.1%
p Bacteroidetes;c Bacteroidia;o Cytophagales;f Microscillaceae;g uncultured;s uncultured-Ohtaekwangia-sp.	0.0%	0.0%	0.0%
p Bacteroidetes;c Bacteroidia;o Cytophagales;f Microscillaceae;g uncultured;s uncultured-Sphingobacteriales-bacterium	0.0%	0.0%	0.0%
p Bacteroidetes;c Bacteroidia;o Cytophagales;f Microscillaceae;g uncultured;s uncultured-bacterium	0.2%	0.3%	0.1%
p Bacteroidetes;c Bacteroidia;o Cytophagales;f Microscillaceae;g uncultured;s uncultured-soil-bacterium	0.1%	0.0%	0.1%
p Bacteroidetes;c Bacteroidia;o Cytophagales;f Spirosomaceae;g Dyadobacter;Other	0.0%	0.0%	0.0%
p Bacteroidetes;c Bacteroidia;o Flavobacteriales;f Crocinitomicaceae;Other;Other	0.0%	0.0%	0.0%
p Bacteroidetes;c Bacteroidia;o Flavobacteriales;f Crocinitomicaceae;g Fluviicola;Other	0.0%	0.0%	0.0%
p Bacteroidetes;c Bacteroidia;o Flavobacteriales;f Crocinitomicaceae;g Fluviicola;s uncultured-bacterium	0.0%	0.0%	0.0%
p Bacteroidetes;c Bacteroidia;o Flavobacteriales;f Flavobacteriaceae;g Flavobacterium;Other	0.3%	0.1%	0.4%
p Bacteroidetes;c Bacteroidia;o Flavobacteriales;f Flavobacteriaceae;g Flavobacterium;s uncultured-Bacteroidetes-bacterium	0.0%	0.0%	0.0%
p Bacteroidetes;c Bacteroidia;o Flavobacteriales;f Flavobacteriaceae;g Flavobacterium;s uncultured-bacterium	0.0%	0.0%	0.0%
p Bacteroidetes;c Bacteroidia;o Flavobacteriales;f NS9-marine-group;Other;Other	0.0%	0.0%	0.0%
p Bacteroidetes;c Bacteroidia;o Flavobacteriales;f NS9-marine-group;g uncultured-bacterium;s uncultured-bacterium	0.0%	0.0%	0.0%
p Bacteroidetes;c Bacteroidia;o Sphingobacteriales;Other;Other;Other	0.0%	0.0%	0.0%
p Bacteroidetes;c Bacteroidia;o Sphingobacteriales;f AKYH767;Other;Other	0.1%	0.1%	0.1%
p Bacteroidetes;c Bacteroidia;o Sphingobacteriales;f AKYH767;g uncultured-bacterium;s uncultured-bacterium	0.2%	0.2%	0.2%
p Bacteroidetes;c Bacteroidia;o Sphingobacteriales;f NS11-12-marine-group;g uncultured-bacterium;s uncultured-bacterium	0.0%	0.0%	0.0%
p Bacteroidetes;c Bacteroidia;o Sphingobacteriales;f Sphingobacteriaceae;Other;Other	0.0%	0.0%	0.0%
p Bacteroidetes;c Bacteroidia;o Sphingobacteriales;f Sphingobacteriaceae;g Olivibacter;Other	0.0%	0.0%	0.0%
p Bacteroidetes;c Bacteroidia;o Sphingobacteriales;f Sphingobacteriaceae;g Pedobacter;Other	0.0%	0.0%	0.0%
p Bacteroidetes;c Bacteroidia;o Sphingobacteriales;f Sphingobacteriaceae;g Solitalea;Other	0.0%	0.1%	0.0%
p Bacteroidetes;c Bacteroidia;o Sphingobacteriales;f Sphingobacteriaceae;g Solitalea;s Solitalea-canadensis	0.1%	0.1%	0.1%
p Bacteroidetes;c Bacteroidia;o Sphingobacteriales;f Sphingobacteriaceae;g Sphingobacterium;Other	0.2%	0.3%	0.0%

p Bacteroidetes;c Bacteroidia;o Sphingobacteriales;f Sphingobacteriaceae;g uncultured;s uncultured-bacterium	0.0%	0.0%	0.0%
p Bacteroidetes;c Bacteroidia;o Sphingobacteriales;f env.OPS-17;Other;Other	0.1%	0.1%	0.1%
p Bacteroidetes;c Bacteroidia;o Sphingobacteriales;f env.OPS-17;g uncultured-bacterium;s uncultured-bacterium	0.2%	0.1%	0.3%
p Bacteroidetes;c Ignavibacteria;o Ignavibacteriales;f uncultured-bacterium;g uncultured-bacterium;s uncultured-bacterium	0.0%	0.0%	0.0%
p Bacteroidetes;c Ignavibacteria;o Kryptoniales;f BSV26;Other;Other	0.1%	0.1%	0.0%
p Bacteroidetes;c Ignavibacteria;o Kryptoniales;f BSV26;g uncultured-Chlorobi-bacterium;s uncultured-Chlorobi-bacterium	0.0%	0.0%	0.0%
p Bacteroidetes;c Ignavibacteria;o OPB56;Other;Other;Other	0.0%	0.0%	0.0%
p Bacteroidetes;c Ignavibacteria;o OPB56;f uncultured-bacterium;g uncultured-bacterium;s uncultured-bacterium	0.0%	0.0%	0.0%
p Bacteroidetes;c Ignavibacteria;o OPB56;f uncultured-bacterium-#0319-6E22;g uncultured-bacterium-#0319-6E22;s uncultured-bacterium-#0319-6E22	0.0%	0.0%	0.0%
p Bacteroidetes;c Ignavibacteria;o OPB56;f uncultured-bacterium-KF-JG30-B11;g uncultured-bacterium-KF-JG30-B11;s uncultured-bacterium-KF-JG30-B11	0.0%	0.0%	0.0%
p Bacteroidetes;c Ignavibacteria;o SJA-28;f uncultured-bacterium;g uncultured-bacterium;s uncultured-bacterium	0.0%	0.1%	0.0%
p Chloroflexi;Other;Other;Other;Other;Other	0.0%	0.0%	0.0%
p Chloroflexi;c AD3;o uncultured-bacterium;f uncultured-bacterium;g uncultured-bacterium;s uncultured-bacterium	0.0%	0.0%	0.0%
p Chloroflexi;c Anaerolineae;Other;Other;Other;Other	0.0%	0.0%	0.0%
p Chloroflexi;c Anaerolineae;o Anaerolineales;f Anaerolineaceae;Other;Other	0.3%	0.3%	0.4%
p Chloroflexi;c Anaerolineae;o Anaerolineales;f Anaerolineaceae;g Anaerolinea;Other	0.0%	0.0%	0.0%
p Chloroflexi;c Anaerolineae;o Anaerolineales;f Anaerolineaceae;g Longilinea;s uncultured-Chloroflexi-bacterium	0.4%	0.2%	0.7%
p Chloroflexi;c Anaerolineae;o Anaerolineales;f Anaerolineaceae;g uncultured;Other	0.3%	0.3%	0.3%
p Chloroflexi;c Anaerolineae;o Anaerolineales;f Anaerolineaceae;g uncultured;s uncultured-Anaerolineae-bacterium	0.0%	0.0%	0.1%
p Chloroflexi;c Anaerolineae;o Anaerolineales;f Anaerolineaceae;g uncultured;s uncultured-Bellilinea-sp.	0.1%	0.1%	0.1%
p Chloroflexi;c Anaerolineae;o Anaerolineales;f Anaerolineaceae;g uncultured;s uncultured-sludge-bacterium-H39	0.0%	0.0%	0.0%
p Chloroflexi;c Anaerolineae;o Anaerolineales;f Anaerolineaceae;g uncultured;s uncultured-soil-bacterium	0.0%	0.0%	0.0%
p Chloroflexi;c Anaerolineae;o Ardenticatenales;f uncultured;Other;Other	0.1%	0.0%	0.1%
p Chloroflexi;c Anaerolineae;o Ardenticatenales;f uncultured;g uncultured-Litorilinea-sp.;s uncultured-Litorilinea-sp.	0.1%	0.1%	0.2%
p Chloroflexi;c Anaerolineae;o Ardenticatenales;f uncultured;g uncultured-prokaryote;s uncultured-prokaryote	0.0%	0.0%	0.0%
p Chloroflexi;c Anaerolineae;o Caldilineales;f Caldilineaceae;g Litorilinea;Other	0.0%	0.0%	0.0%
p Chloroflexi;c Anaerolineae;o Caldilineales;f Caldilineaceae;g Litorilinea;s uncultured-bacterium	0.0%	0.0%	0.0%
p Chloroflexi;c Anaerolineae;o Caldilineales;f Caldilineaceae;g Litorilinea;s uncultured-soil-bacterium	0.0%	0.0%	0.0%
p Chloroflexi;c Anaerolineae;o Caldilineales;f Caldilineaceae;g uncultured;Other	0.0%	0.0%	0.0%
p Chloroflexi;c Anaerolineae;o Caldilineales;f Caldilineaceae;g uncultured;s Chloroflexi-bacterium-ET1	0.0%	0.0%	0.0%
p Chloroflexi;c Anaerolineae;o Caldilineales;f Caldilineaceae;g uncultured;s uncultured-bacterium	0.0%	0.0%	0.0%
p Chloroflexi;c Anaerolineae;o RBG-13-54-9;Other;Other;Other	0.0%	0.0%	0.1%
p Chloroflexi;c Anaerolineae;o RBG-13-54-9;f uncultured-Caldilineaceae-bacterium;g uncultured-Caldilineaceae-bacterium;s uncultured-Caldilineaceae-bacterium	0.0%	0.0%	0.0%
p Chloroflexi;c Anaerolineae;o RBG-13-54-9;f uncultured-sludge-bacterium-A31;g uncultured-sludge-bacterium-A31;s uncultured-sludge-bacterium-A31	0.0%	0.0%	0.0%
p Chloroflexi;c Anaerolineae;o SBR1031;Other;Other;Other	0.1%	0.2%	0.0%
p Chloroflexi;c Anaerolineae;o SBR1031;f A4b;Other;Other	0.0%	0.0%	0.1%
p Chloroflexi;c Anaerolineae;o SBR1031;f A4b;g uncultured-Caldilineales-bacterium;s uncultured-Caldilineales-bacterium	0.1%	0.0%	0.1%
p Chloroflexi;c Anaerolineae;o SBR1031;f A4b;g uncultured-bacterium;s uncultured-bacterium	0.1%	0.1%	0.2%
p Chloroflexi;c Anaerolineae;o SBR1031;f A4b;g uncultured-sludge-bacterium-H8;s uncultured-sludge-bacterium-H8	0.0%	0.0%	0.0%
p Chloroflexi;c Anaerolineae;o SBR1031;f A4b;g uncultured-sludge-bacterium-S14;s uncultured-sludge-bacterium-S14	0.0%	0.0%	0.0%
p Chloroflexi;c Anaerolineae;o SBR1031;f A4b;g uncultured-soil-bacterium;s uncultured-soil-bacterium	0.0%	0.0%	0.0%
p Chloroflexi;c Anaerolineae;o SBR1031;f uncultured-Caldilineaceae-bacterium;g uncultured-Caldilineaceae-bacterium;s uncultured-Caldilineaceae-bacterium	0.0%	0.0%	0.0%
p Chloroflexi;c Anaerolineae;o SBR1031;f uncultured-bacterium;g uncultured-bacterium;s uncultured-bacterium	0.0%	0.0%	0.0%
p Chloroflexi;c Anaerolineae;o SBR1031;f uncultured-soil-bacterium;g uncultured-soil-bacterium;s uncultured-soil-bacterium	0.0%	0.0%	0.0%
p Chloroflexi;c Anaerolineae;o uncultured-Bellilinea-sp.;f uncultured-Bellilinea-sp.;g uncultured-Bellilinea-sp.;s uncultured-Bellilinea-sp.	0.0%	0.0%	0.0%
p Chloroflexi;c Chloroflexia;o Chloroflexales;Other;Other;Other	0.0%	0.0%	0.0%
p Chloroflexi;c Chloroflexia;o Chloroflexales;f Chloroflexaceae;Other;Other	0.0%	0.0%	0.0%
p Chloroflexi;c Chloroflexia;o Chloroflexales;f Chloroflexaceae;g Candidatus-Chloroploca;s uncultured-bacterium	0.0%	0.0%	0.0%
p Chloroflexi;c Chloroflexia;o Chloroflexales;f Herpetosiphonaceae;g Herpetosiphon;s uncultured-bacterium	0.0%	0.0%	0.0%
p Chloroflexi;c Chloroflexia;o Chloroflexales;f Roseiflexaceae;g Roseiflexus;s uncultured-bacterium	0.0%	0.0%	0.0%
p Chloroflexi;c Chloroflexia;o Chloroflexales;f Roseiflexaceae;g uncultured;Other	0.2%	0.1%	0.3%
p Chloroflexi;c Chloroflexia;o Chloroflexales;f Roseiflexaceae;g uncultured;s uncultured-bacterium	1.0%	0.3%	1.7%
p Chloroflexi;c Chloroflexia;o Chloroflexales;f Roseiflexaceae;g uncultured;s uncultured-soil-bacterium	0.0%	0.0%	0.0%
p Chloroflexi;c Chloroflexia;o Kallotenuales;f AKIW781;Other;Other	0.0%	0.0%	0.0%
p Chloroflexi;c Chloroflexia;o Kallotenuales;f AKIW781;g uncultured-bacterium;s uncultured-bacterium	0.0%	0.0%	0.0%
p Chloroflexi;c Chloroflexia;o Thermomicrobiales;f AKYG1722;Other;Other	0.0%	0.1%	0.0%
p Chloroflexi;c Chloroflexia;o Thermomicrobiales;f AKYG1722;g uncultured-Chloroflexi-bacterium;s uncultured-Chloroflexi-bacterium	0.1%	0.1%	0.1%
p Chloroflexi;c Chloroflexia;o Thermomicrobiales;f AKYG1722;g uncultured-bacterium;s uncultured-bacterium	0.0%	0.0%	0.0%
p Chloroflexi;c Chloroflexia;o Thermomicrobiales;f AKYG1722;g uncultured-soil-bacterium;s uncultured-soil-bacterium	0.0%	0.0%	0.0%
p Chloroflexi;c Chloroflexia;o Thermomicrobiales;f JG30-KF-CM45;Other;Other	0.2%	0.2%	0.3%

p Chloroflexi;c Chloroflexia;o Thermomicrobiales;f JG30-KF-CM45;g uncultured-Chloroflexi-bacterium;s uncultured-Chloroflexi-bacterium	0.0%	0.0%	0.0%
p Chloroflexi;c Chloroflexia;o Thermomicrobiales;f JG30-KF-CM45;g uncultured-bacterium;s uncultured-bacterium	0.1%	0.1%	0.1%
p Chloroflexi;c Chloroflexia;o Thermomicrobiales;f JG30-KF-CM45;g uncultured-soil-bacterium;s uncultured-soil-bacterium	0.0%	0.0%	0.0%
p Chloroflexi;c Chloroflexia;o Thermomicrobiales;f Thermomicrobiaceae;g Nitrolancea;s uncultured-bacterium	0.0%	0.0%	0.0%
p Chloroflexi;c Dehalococcoidia;o S085;Other;Other;Other	0.2%	0.3%	0.1%
p Chloroflexi;c Dehalococcoidia;o S085;f uncultured-Chloroflexi-bacterium;g uncultured-Chloroflexi-bacterium;s uncultured-Chloroflexi-bacterium	0.0%	0.0%	0.0%
p Chloroflexi;c Dehalococcoidia;o S085;f uncultured-bacterium;g uncultured-bacterium;s uncultured-bacterium	0.1%	0.2%	0.1%
p Chloroflexi;c Dehalococcoidia;o S085;f uncultured-soil-bacterium;g uncultured-soil-bacterium;s uncultured-soil-bacterium	0.0%	0.0%	0.0%
p Chloroflexi;c Gitt-GS-136;Other;Other;Other;Other	0.0%	0.0%	0.0%
p Chloroflexi;c Gitt-GS-136;o uncultured-bacterium;f uncultured-bacterium;g uncultured-bacterium;s uncultured-bacterium	0.3%	0.4%	0.1%
p Chloroflexi;c JG30-KF-CM66;Other;Other;Other;Other	0.0%	0.0%	0.0%
p Chloroflexi;c JG30-KF-CM66;o uncultured-Chloroflexi-bacterium;f uncultured-Chloroflexi-bacterium;g uncultured-Chloroflexi-bacterium;s uncultured-Chloroflexi-bacterium	0.0%	0.0%	0.0%
p Chloroflexi;c JG30-KF-CM66;o uncultured-bacterium;f uncultured-bacterium;g uncultured-bacterium;s uncultured-bacterium	0.1%	0.1%	0.1%
p Chloroflexi;c KD4-96;Other;Other;Other;Other	0.2%	0.3%	0.1%
p Chloroflexi;c KD4-96;o uncultured-Chloroflexi-bacterium;f uncultured-Chloroflexi-bacterium;g uncultured-Chloroflexi-bacterium;s uncultured-Chloroflexi-bacterium	0.1%	0.1%	0.1%
p Chloroflexi;c KD4-96;o uncultured-bacterium;f uncultured-bacterium;g uncultured-bacterium;s uncultured-bacterium	0.4%	0.5%	0.3%
p Chloroflexi;c Ktedonobacteria;o C0119;Other;Other;Other	0.0%	0.0%	0.0%
p Chloroflexi;c Ktedonobacteria;o C0119;f uncultured-soil-bacterium;g uncultured-soil-bacterium;s uncultured-soil-bacterium	0.0%	0.0%	0.0%
p Chloroflexi;c Ktedonobacteria;o Ktedonobacterales;f Ktedonobacteraceae;Other;Other	0.0%	0.0%	0.0%
p Chloroflexi;c Ktedonobacteria;o Ktedonobacterales;f Ktedonobacteraceae;g Thermosporothrix;s uncultured-bacterium	0.0%	0.0%	0.0%
p Chloroflexi;c Ktedonobacteria;o Ktedonobacterales;f Ktedonobacteraceae;g uncultured;Other	0.0%	0.0%	0.0%
p Chloroflexi;c OLB14;Other;Other;Other;Other	0.0%	0.0%	0.0%
p Chloroflexi;c OLB14;o uncultured-bacterium;f uncultured-bacterium;g uncultured-bacterium;s uncultured-bacterium	0.1%	0.1%	0.1%
p Chloroflexi;c OLB14;o uncultured-gamma-proteobacterium;f uncultured-gamma-proteobacterium;g uncultured-gamma-proteobacterium;s uncultured-gamma-proteobacterium	0.0%	0.0%	0.0%
p Chloroflexi;c P2-11E;o uncultured-bacterium;f uncultured-bacterium;g uncultured-bacterium;s uncultured-bacterium	0.2%	0.1%	0.2%
p Chloroflexi;c TK10;Other;Other;Other;Other	0.2%	0.2%	0.2%
p Chloroflexi;c TK10;o uncultured-Chloroflexi-bacterium;f uncultured-Chloroflexi-bacterium;g uncultured-Chloroflexi-bacterium;s uncultured-Chloroflexi-bacterium	0.0%	0.0%	0.1%
p Chloroflexi;c TK10;o uncultured-bacterium;f uncultured-bacterium;g uncultured-bacterium;s uncultured-bacterium	0.0%	0.1%	0.0%
p Dadabacteria;c Dadabacteriia;o Dadabacteriales;f uncultured-soil-bacterium;g uncultured-soil-bacterium;s uncultured-soil-bacterium	0.0%	0.0%	0.0%
p Dependentiae;c Babeliae;o Babeliales;Other;Other;Other	0.0%	0.0%	0.0%
p Dependentiae;c Babeliae;o Babeliales;f Babeliaceae;g uncultured-bacterium;s uncultured-bacterium	0.0%	0.0%	0.0%
p Dependentiae;c Babeliae;o Babeliales;f Vermiphilaceae;Other;Other	0.0%	0.0%	0.0%
p Dependentiae;c Babeliae;o Babeliales;f Vermiphilaceae;g uncultured-bacterium;s uncultured-bacterium	0.0%	0.0%	0.0%
p Elusimicrobia;c Elusimicrobia;o MVP-88;Other;Other;Other	0.0%	0.0%	0.0%
p Elusimicrobia;c Lineage-IIa;Other;Other;Other;Other	0.0%	0.0%	0.0%
p Elusimicrobia;c Lineage-IIa;o uncultured-bacterium;f uncultured-bacterium;g uncultured-bacterium;s uncultured-bacterium	0.0%	0.1%	0.0%
p Elusimicrobia;c Lineage-IIb;o uncultured-bacterium;f uncultured-bacterium;g uncultured-bacterium;s uncultured-bacterium	0.0%	0.0%	0.0%
p Elusimicrobia;c Lineage-IIb;o uncultured-soil-bacterium;f uncultured-soil-bacterium;g uncultured-soil-bacterium;s uncultured-soil-bacterium	0.0%	0.0%	0.0%
p Entotheonellaeota;c Entotheonellia;o Entotheonellales;f Entotheonellaceae;Other;Other	0.0%	0.0%	0.0%
p Entotheonellaeota;c Entotheonellia;o Entotheonellales;f Entotheonellaceae;g Candidatus-Entotheonella;Other	0.0%	0.0%	0.0%
p Entotheonellaeota;c Entotheonellia;o Entotheonellales;f Entotheonellaceae;g Candidatus-Entotheonella;s uncultured-delta-proteobacterium	0.0%	0.0%	0.0%
p Entotheonellaeota;c Entotheonellia;o Entotheonellales;f Entotheonellaceae;g uncultured-bacterium;s uncultured-bacterium	0.0%	0.1%	0.0%
p Euryarchaeota;c Methanomicrobia;o Methanosarcinales;f Methanosarcinaceae;g Methanosarcina;s uncultured-archaeon	0.0%	0.0%	0.0%
p Euryarchaeota;c Thermoplasmata;Other;Other;Other;Other	0.1%	0.1%	0.1%
p Euryarchaeota;c Thermoplasmata;o Marine-Group-II;Other;Other;Other	0.2%	0.1%	0.2%
p Euryarchaeota;c Thermoplasmata;o Marine-Group-II;f uncultured-archaeon;g uncultured-archaeon;s uncultured-archaeon	1.1%	0.6%	1.6%
p Euryarchaeota;c Thermoplasmata;o Marine-Group-II;f uncultured-haloarchaeon;g uncultured-haloarchaeon;s uncultured-haloarchaeon	0.0%	0.0%	0.0%
p Euryarchaeota;c Thermoplasmata;o Methanomassiliicoccales;f uncultured;g uncultured-archaeon;s uncultured-archaeon	0.5%	0.0%	0.9%
p Euryarchaeota;c Thermoplasmata;o uncultured;Other;Other;Other	0.0%	0.0%	0.0%
p Firmicutes;c Bacilli;o Bacillales;f Bacillaceae;Other;Other	0.0%	0.0%	0.0%
p Firmicutes;c Bacilli;o Bacillales;f Bacillaceae;g Bacillus;Other	0.2%	0.1%	0.4%
p Firmicutes;c Bacilli;o Bacillales;f Bacillaceae;g Bacillus;s Bacillus-acidiceler	0.0%	0.0%	0.0%

	p Firmicutes;c Bacilli;o Bacillales;f Bacillaceae;g Bacillus;s Bacillus-aryabhattai	0.1%	0.1%	0.1%
	p Firmicutes;c Bacilli;o Bacillales;f Bacillaceae;g Bacillus;s Bacillus-drentensis	1.4%	0.2%	2.6%
	p Firmicutes;c Bacilli;o Bacillales;f Bacillaceae;g Bacillus;s Bacillus-funiculus	0.8%	0.2%	1.3%
	p Firmicutes;c Bacilli;o Bacillales;f Bacillaceae;g Bacillus;s Bacillus-pumilus	0.0%	0.0%	0.0%
	p Firmicutes;c Bacilli;o Bacillales;f Bacillaceae;g Bacillus;s Bacillus-simplex	0.0%	0.1%	0.0%
	p Firmicutes;c Bacilli;o Bacillales;f Bacillaceae;g Bacillus;s uncultured-soil-bacterium	0.0%	0.0%	0.0%
	p Firmicutes;c Bacilli;o Bacillales;f Bacillaceae;g Fictibacillus;s Fictibacillus-barbaricus	0.0%	0.0%	0.0%
	p Firmicutes;c Bacilli;o Bacillales;f Paenibacillaceae;g Brevibacillus;s uncultured-bacterium	0.0%	0.0%	0.0%
	p Firmicutes;c Bacilli;o Bacillales;f Paenibacillaceae;g Oxalophagus;Other	0.1%	0.0%	0.2%
	p Firmicutes;c Bacilli;o Bacillales;f Paenibacillaceae;g Paenibacillus;Other	0.1%	0.1%	0.1%
	p Firmicutes;c Bacilli;o Bacillales;f Paenibacillaceae;g Thermobacillus;s Thermobacillus-xylanilyticus	0.0%	0.0%	0.0%
	p Firmicutes;c Bacilli;o Bacillales;f Planococcaceae;g Domibacillus;Other	0.0%	0.0%	0.0%
	p Firmicutes;c Bacilli;o Bacillales;f Planococcaceae;g Lysinibacillus;Other	0.0%	0.0%	0.0%
	p Firmicutes;c Bacilli;o Bacillales;f Planococcaceae;g Sporosarcina;s uncultured-bacterium	0.0%	0.0%	0.0%
	p Firmicutes;c Bacilli;o Bacillales;f Thermoactinomycetaceae;g Shimazuella;s uncultured-bacterium	0.0%	0.0%	0.0%
	p Firmicutes;c Clostridia;o Clostridiales;f Clostridiaceae-1;g Clostridium-sensu-stricto-1;Other	0.0%	0.0%	0.0%
	p Firmicutes;c Clostridia;o Clostridiales;f Heliobacteriaceae;g Hydrogenispora;s uncultured-bacterium	0.0%	0.0%	0.0%
	p Firmicutes;c Clostridia;o Clostridiales;f Peptostreptococcaceae;g Sporacetigenium;Other	0.0%	0.0%	0.0%
	p Firmicutes;c Clostridia;o Clostridiales;f Ruminococcaceae;g Ruminococcus-2;s uncultured-Papillibacter-sp.	0.0%	0.0%	0.0%
	p GAL15;c uncultured-bacterium;o uncultured-bacterium;f uncultured-bacterium;g uncultured-bacterium;s uncultured-bacterium	0.0%	0.0%	0.0%
	p Gemmatimonadetes;c AKAU4049;Other;Other;Other;Other	0.2%	0.3%	0.0%
	p Gemmatimonadetes;c BD2-11-terrestrial-group;Other;Other;Other;Other	0.0%	0.0%	0.0%
	p Gemmatimonadetes;c BD2-11-terrestrial-group;o uncultured-Gemmatimonadales-bacterium;f uncultured-Gemmatimonadales-bacterium;g uncultured-Gemmatimonadales-bacterium;s uncultured-Gemmatimonadales-bacterium	0.0%	0.0%	0.0%
	p Gemmatimonadetes;c BD2-11-terrestrial-group;o uncultured-Gemmatimonadetes-bacterium;f uncultured-Gemmatimonadetes-bacterium;g uncultured-Gemmatimonadetes-bacterium;s uncultured-Gemmatimonadetes-bacterium	0.0%	0.1%	0.0%
	p Gemmatimonadetes;c BD2-11-terrestrial-group;o uncultured-bacterium;f uncultured-bacterium;g uncultured-bacterium;s uncultured-bacterium	0.0%	0.0%	0.0%
	p Gemmatimonadetes;c BD2-11-terrestrial-group;o uncultured-soil-bacterium;f uncultured-soil-bacterium;g uncultured-soil-bacterium;s uncultured-soil-bacterium	0.0%	0.0%	0.0%
	p Gemmatimonadetes;c Gemmatimonadetes;o Gemmatimonadales;f Gemmatimonadaceae;Other;Other	0.2%	0.2%	0.2%
	p Gemmatimonadetes;c Gemmatimonadetes;o Gemmatimonadales;f Gemmatimonadaceae;g Gemmatimonas;Other	0.1%	0.1%	0.1%
	p Gemmatimonadetes;c Gemmatimonadetes;o Gemmatimonadales;f Gemmatimonadaceae;g Gemmatimonas;s uncultured-Gemmatimonadaceae-bacterium	0.0%	0.0%	0.0%
	p Gemmatimonadetes;c Gemmatimonadetes;o Gemmatimonadales;f Gemmatimonadaceae;g Gemmatimonas;s uncultured-bacterium	0.0%	0.0%	0.0%
	p Gemmatimonadetes;c Gemmatimonadetes;o Gemmatimonadales;f Gemmatimonadaceae;g Gemmatirosa;s uncultured-bacterium	0.1%	0.0%	0.1%
	p Gemmatimonadetes;c Gemmatimonadetes;o Gemmatimonadales;f Gemmatimonadaceae;g uncultured;Other	0.8%	0.9%	0.8%
	p Gemmatimonadetes;c Gemmatimonadetes;o Gemmatimonadales;f Gemmatimonadaceae;g uncultured;s uncultured-Gemmatimonadales-bacterium	0.5%	0.6%	0.5%
	p Gemmatimonadetes;c Gemmatimonadetes;o Gemmatimonadales;f Gemmatimonadaceae;g uncultured;s uncultured-Gemmatimonadetes-bacterium	0.1%	0.0%	0.1%
	p Gemmatimonadetes;c Gemmatimonadetes;o Gemmatimonadales;f Gemmatimonadaceae;g uncultured;s uncultured-Gemmatimonas-sp.	0.1%	0.0%	0.1%
	p Gemmatimonadetes;c Gemmatimonadetes;o Gemmatimonadales;f Gemmatimonadaceae;g uncultured;s uncultured-bacterium	0.5%	0.3%	0.8%
	p Gemmatimonadetes;c Gemmatimonadetes;o Gemmatimonadales;f Gemmatimonadaceae;g uncultured;s uncultured-low-G+C-Gram-positive-bacterium	0.0%	0.0%	0.0%
	p Gemmatimonadetes;c Gemmatimonadetes;o Gemmatimonadales;f Gemmatimonadaceae;g uncultured;s uncultured-proteobacterium	0.2%	0.4%	0.0%
	p Gemmatimonadetes;c Longimicrobia;o Longimicrobiales;f Longimicrobiaceae;g uncultured-bacterium;s uncultured-bacterium	0.0%	0.0%	0.0%
	p Gemmatimonadetes;c S0134-terrestrial-group;Other;Other;Other;Other	0.1%	0.1%	0.1%
	p Gemmatimonadetes;c S0134-terrestrial-group;o uncultured-Gemmatimonadales-bacterium;f uncultured-Gemmatimonadales-bacterium;g uncultured-Gemmatimonadales-bacterium;s uncultured-Gemmatimonadales-bacterium	0.2%	0.2%	0.2%
	p Gemmatimonadetes;c S0134-terrestrial-group;o uncultured-Gemmatimonadetes-bacterium;f uncultured-Gemmatimonadetes-bacterium;g uncultured-Gemmatimonadetes-bacterium;s uncultured-Gemmatimonadetes-bacterium	0.0%	0.0%	0.0%
	p Gemmatimonadetes;c S0134-terrestrial-group;o uncultured-bacterium;f uncultured-bacterium;g uncultured-bacterium;s uncultured-bacterium	0.2%	0.2%	0.2%
	p Hydrogenedentes;c Hydrogenedentia;o Hydrogenedentiales;f Hydrogenedensaceae;g uncultured-bacterium;s uncultured-bacterium	0.0%	0.0%	0.0%
	p Hydrogenedentes;c Hydrogenedentia;o Hydrogenedentiales;f Hydrogenedensaceae;g uncultured-bacterium-SJP-3;s uncultured-bacterium-SJP-3	0.0%	0.0%	0.0%
	p Latescibacteria;Other;Other;Other;Other;Other	0.3%	0.4%	0.1%
	p Latescibacteria;c Latescibacteria;o Latescibacterales;f Latescibacteraceae;Other;Other	0.0%	0.0%	0.0%
	p Latescibacteria;c Latescibacteria;o Latescibacterales;f Latescibacteraceae;g uncultured-bacterium;s uncultured-bacterium	0.1%	0.2%	0.0%
	p Latescibacteria;c uncultured-Acidobacterium-sp.;o uncultured-Acidobacterium-sp.;f uncultured-Acidobacterium-sp.;g uncultured-Acidobacterium-sp.;s uncultured-Acidobacterium-sp.	0.0%	0.0%	0.0%
	p Latescibacteria;c uncultured-Fibrobacteres-bacterium;o uncultured-Fibrobacteres-bacterium;f uncultured-Fibrobacteres-bacterium;g uncultured-Fibrobacteres-bacterium;s uncultured-Fibrobacteres-bacterium	0.0%	0.0%	0.0%

	p Latescibacteria;c uncultured-Pelobacter-sp.;o uncultured-Pelobacter-sp.;f uncultured-Pelobacter-sp.;g uncultured-Pelobacter-sp.;s uncultured-Pelobacter-sp.	0.0%	0.0%	0.0%
	p Latescibacteria;c uncultured-bacterium;o uncultured-bacterium;f uncultured-bacterium;g uncultured-bacterium;s uncultured-bacterium	0.4%	0.5%	0.3%
	p Latescibacteria;c uncultured-prokaryote;o uncultured-prokaryote;f uncultured-prokaryote;g uncultured-prokaryote;s uncultured-prokaryote	0.0%	0.0%	0.0%
	p Latescibacteria;c uncultured-proteobacterium;o uncultured-proteobacterium;f uncultured-proteobacterium;g uncultured-proteobacterium;s uncultured-proteobacterium	0.0%	0.0%	0.0%
	p Latescibacteria;c uncultured-soil-bacterium;o uncultured-soil-bacterium;f uncultured-soil-bacterium;g uncultured-soil-bacterium;s uncultured-soil-bacterium	0.0%	0.1%	0.0%
	p Nanoarchaeaeota;c Nanohaloarchaeia;o Aenigmarchaeales;Other;Other;Other	0.0%	0.0%	0.0%
	p Nanoarchaeaeota;c Nanohaloarchaeia;o Aenigmarchaeales;f uncultured-archaeon;g uncultured-archaeon;s uncultured-archaeon	0.0%	0.1%	0.0%
	p Nanoarchaeaeota;c Nanohaloarchaeia;o Deep-Sea-Euryarchaeotic-Group(DSEG);Other;Other;Other	0.0%	0.0%	0.0%
	p Nanoarchaeaeota;c Woesearchaeia;Other;Other;Other;Other	0.1%	0.2%	0.1%
	p Nanoarchaeaeota;c Woesearchaeia;o Candidatus-Amesbacteria-bacterium-GW2011_GWC1_47_15;f Candidatus-Amesbacteria-bacterium-GW2011_GWC1_47_15;g Candidatus-Amesbacteria-bacterium-GW2011_GWC1_47_15;s Candidatus-Amesbacteria-bacterium-GW2011_GWC1_47_15	0.0%	0.0%	0.0%
	p Nanoarchaeaeota;c Woesearchaeia;o Candidatus-Pacearchaeota-archaeon-RBG_19FT_COMBO_34_9;f Candidatus-Pacearchaeota-archaeon-RBG_19FT_COMBO_34_9;g Candidatus-Pacearchaeota-archaeon-RBG_19FT_COMBO_34_9;s Candidatus-Pacearchaeota-archaeon-RBG_19FT_COMBO_34_9	0.0%	0.0%	0.0%
	p Nanoarchaeaeota;c Woesearchaeia;o Candidatus-Staskawiczbacteria-bacterium-RIFOXYA2_FULL_32_7;f Candidatus-Staskawiczbacteria-bacterium-RIFOXYA2_FULL_32_7;g Candidatus-Staskawiczbacteria-bacterium-RIFOXYA2_FULL_32_7;s Candidatus-Staskawiczbacteria-bacterium-RIFOXYA2_FULL_32_7	0.1%	0.1%	0.0%
	p Nanoarchaeaeota;c Woesearchaeia;o uncultured-euryarchaeote;f uncultured-euryarchaeote;g uncultured-euryarchaeote;s uncultured-euryarchaeote	0.0%	0.0%	0.0%
	p Nitrospirae;c Nitrospira;o Nitrospirales;f Nitrospiraceae;g Nitrospira;Other	0.4%	0.3%	0.5%
	p Nitrospirae;c Nitrospira;o Nitrospirales;f Nitrospiraceae;g Nitrospira;s uncultured-Nitrospira-sp.	0.1%	0.0%	0.1%
	p Nitrospirae;c Nitrospira;o Nitrospirales;f Nitrospiraceae;g Nitrospira;s uncultured-Nitrospiraceae-bacterium	0.0%	0.0%	0.0%
	p Nitrospirae;c Nitrospira;o Nitrospirales;f Nitrospiraceae;g Nitrospira;s uncultured-bacterium	0.0%	0.0%	0.0%
	p Nitrospirae;c Nitrospira;o Nitrospirales;f Nitrospiraceae;g Nitrospira;s uncultured-soil-bacterium	0.0%	0.0%	0.1%
	p Patescibacteria;Other;Other;Other;Other;Other	0.0%	0.0%	0.0%
	p Patescibacteria;c ABY1;Other;Other;Other;Other	0.0%	0.0%	0.0%
	p Patescibacteria;c ABY1;o Candidatus-Kuenenbacteria;f uncultured-bacterium;g uncultured-bacterium;s uncultured-bacterium	0.0%	0.0%	0.1%
	p Patescibacteria;c ABY1;o Candidatus-Magasanikbacteria;Other;Other;Other	0.0%	0.0%	0.0%
	p Patescibacteria;c ABY1;o Candidatus-Magasanikbacteria;f uncultured-bacterium;g uncultured-bacterium;s uncultured-bacterium	0.0%	0.0%	0.0%
	p Patescibacteria;c ABY1;o Candidatus-Uhrbacteria;Other;Other;Other	0.0%	0.0%	0.0%
	p Patescibacteria;c Berkelbacteria;o uncultured-bacterium;f uncultured-bacterium;g uncultured-bacterium;s uncultured-bacterium	0.0%	0.0%	0.0%
	p Patescibacteria;c Gracilibacteria;o Candidatus-Abawacabacteria;Other;Other;Other	0.0%	0.0%	0.0%
	p Patescibacteria;c Gracilibacteria;o Candidatus-Abawacabacteria;f Candidatus-Abawacabacteria-bacterium-RBG_16_42_10;g Candidatus-Abawacabacteria-bacterium-RBG_16_42_10;s Candidatus-Abawacabacteria-bacterium-RBG_16_42_10	0.0%	0.0%	0.0%
	p Patescibacteria;c Gracilibacteria;o Candidatus-Peribacteria;f Candidatus-Peribacteria-bacterium-RIFCSPHIGHO2_02_FULL_53_20;g Candidatus-Peribacteria-bacterium-RIFCSPHIGHO2_02_FULL_53_20;s Candidatus-Peribacteria-bacterium-RIFCSPHIGHO2_02_FULL_53_20	0.0%	0.0%	0.0%
	p Patescibacteria;c Microgenomatia;o Candidatus-Woesebacteria;Other;Other;Other	0.0%	0.0%	0.0%
	p Patescibacteria;c Parcubacteria;Other;Other;Other;Other	0.0%	0.0%	0.0%
	p Patescibacteria;c Parcubacteria;o Candidatus-Azambacteria;Other;Other;Other	0.0%	0.0%	0.0%
	p Patescibacteria;c Parcubacteria;o Candidatus-Azambacteria;f uncultured-bacterium;g uncultured-bacterium;s uncultured-bacterium	0.0%	0.0%	0.0%
	p Patescibacteria;c Parcubacteria;o Candidatus-Kaiserbacteria;Other;Other;Other	0.0%	0.0%	0.0%
	p Patescibacteria;c Parcubacteria;o Candidatus-Nomurabacteria;Other;Other;Other	0.0%	0.0%	0.0%
	p Patescibacteria;c Parcubacteria;o Candidatus-Terrybacteria;f uncultured-bacterium;g uncultured-bacterium;s uncultured-bacterium	0.0%	0.0%	0.0%
	p Patescibacteria;c Parcubacteria;o Candidatus-Yanofskybacteria;Other;Other;Other	0.0%	0.0%	0.0%
	p Patescibacteria;c Parcubacteria;o Candidatus-Yanofskybacteria;f Candidatus-Yanofskybacteria-bacterium-RIFCSPHIGHO2_02_FULL_46_19;g Candidatus-Yanofskybacteria-bacterium-RIFCSPHIGHO2_02_FULL_46_19;s Candidatus-Yanofskybacteria-bacterium-RIFCSPHIGHO2_02_FULL_46_19	0.0%	0.0%	0.0%
	p Patescibacteria;c Parcubacteria;o Candidatus-Yanofskybacteria;f uncultured-bacterium;g uncultured-bacterium;s uncultured-bacterium	0.0%	0.0%	0.0%
	p Patescibacteria;c Parcubacteria;o Candidatus-Yanofskybacteria;f uncultured-deep-sea-bacterium;g uncultured-deep-sea-bacterium;s uncultured-deep-sea-bacterium	0.0%	0.0%	0.0%
	p Patescibacteria;c Parcubacteria;o GWA2-38-13b;f uncultured-bacterium;g uncultured-bacterium;s uncultured-bacterium	0.0%	0.0%	0.0%
	p Patescibacteria;c Parcubacteria;o GWA2-38-13b;f uncultured-deep-sea-bacterium;g uncultured-deep-sea-bacterium;s uncultured-deep-sea-bacterium	0.0%	0.0%	0.0%
	p Patescibacteria;c Parcubacteria;o uncultured-bacterium;f uncultured-bacterium;g uncultured-bacterium;s uncultured-bacterium	0.0%	0.0%	0.0%
	p Patescibacteria;c Saccharimonadia;o Saccharimonadales;Other;Other;Other	0.2%	0.2%	0.3%

	p Patescibacteria;c Saccharimonadia;o Saccharimonadales;f Saccharimonadaceae;g uncultured-bacterium;s uncultured-bacterium	0.1%	0.0%	0.2%
	p Patescibacteria;c Saccharimonadia;o Saccharimonadales;f uncultured-Candidatus-Saccharibacteria-bacterium;g uncultured-Candidatus-Saccharibacteria-bacterium;s uncultured-Candidatus-Saccharibacteria-bacterium	0.0%	0.0%	0.1%
	p Patescibacteria;c Saccharimonadia;o Saccharimonadales;f uncultured-bacterium;g uncultured-bacterium;s uncultured-bacterium	0.1%	0.1%	0.1%
	p Patescibacteria;c WWE3;Other;Other;Other;Other	0.0%	0.0%	0.0%
	p Planctomycetes;Other;Other;Other;Other;Other	0.0%	0.0%	0.0%
	p Planctomycetes;c BD7-11;o uncultured-Planctomycetales-bacterium;f uncultured-Planctomycetales-bacterium;g uncultured-Planctomycetales-bacterium;s uncultured-Planctomycetales-bacterium	0.0%	0.0%	0.0%
	p Planctomycetes;c BD7-11;o uncultured-bacterium;f uncultured-bacterium;g uncultured-bacterium;s uncultured-bacterium	0.0%	0.0%	0.0%
	p Planctomycetes;c OM190;Other;Other;Other;Other	0.0%	0.0%	0.0%
	p Planctomycetes;c OM190;o uncultured-bacterium;f uncultured-bacterium;g uncultured-bacterium;s uncultured-bacterium	0.0%	0.0%	0.0%
	p Planctomycetes;c OM190;o uncultured-soil-bacterium;f uncultured-soil-bacterium;g uncultured-soil-bacterium;s uncultured-soil-bacterium	0.0%	0.0%	0.0%
	p Planctomycetes;c Phycisphaerae;Other;Other;Other;Other	0.2%	0.2%	0.2%
	p Planctomycetes;c Phycisphaerae;o CCM11a;Other;Other;Other	0.1%	0.0%	0.1%
	p Planctomycetes;c Phycisphaerae;o CCM11a;f uncultured-Planctomycetia-bacterium;g uncultured-Planctomycetia-bacterium;s uncultured-Planctomycetia-bacterium	0.0%	0.0%	0.0%
	p Planctomycetes;c Phycisphaerae;o CCM11a;f uncultured-bacterium;g uncultured-bacterium;s uncultured-bacterium	0.1%	0.2%	0.1%
	p Planctomycetes;c Phycisphaerae;o Phycisphaerales;Other;Other;Other	0.0%	0.0%	0.0%
	p Planctomycetes;c Phycisphaerae;o Phycisphaerales;f Phycisphaeraceae;Other;Other	0.0%	0.0%	0.1%
	p Planctomycetes;c Phycisphaerae;o Phycisphaerales;f Phycisphaeraceae;g uncultured;Other	0.1%	0.1%	0.1%
	p Planctomycetes;c Phycisphaerae;o Phycisphaerales;f Phycisphaeraceae;g uncultured;s uncultured-Bacteroidetes-bacterium	0.0%	0.0%	0.0%
	p Planctomycetes;c Phycisphaerae;o Phycisphaerales;f Phycisphaeraceae;g uncultured;s uncultured-bacterium	0.0%	0.0%	0.0%
	p Planctomycetes;c Phycisphaerae;o Pla1-lineage;Other;Other;Other	0.0%	0.0%	0.0%
	p Planctomycetes;c Phycisphaerae;o Pla1-lineage;f uncultured-bacterium;g uncultured-bacterium;s uncultured-bacterium	0.0%	0.0%	0.0%
	p Planctomycetes;c Phycisphaerae;o Tepidisphaerales;f CPla-3-termite-group;Other;Other	0.0%	0.0%	0.0%
	p Planctomycetes;c Phycisphaerae;o Tepidisphaerales;f CPla-3-termite-group;g uncultured-bacterium;s uncultured-bacterium	0.0%	0.0%	0.0%
	p Planctomycetes;c Phycisphaerae;o Tepidisphaerales;f CPla-3-termite-group;g uncultured-soil-bacterium-PBS-22;s uncultured-soil-bacterium-PBS-22	0.0%	0.0%	0.0%
	p Planctomycetes;c Phycisphaerae;o Tepidisphaerales;f Tepidisphaeraceae;g uncultured-bacterium;s uncultured-bacterium	0.0%	0.0%	0.0%
	p Planctomycetes;c Phycisphaerae;o Tepidisphaerales;f WD2101-soil-group;Other;Other	0.4%	0.2%	0.6%
	p Planctomycetes;c Phycisphaerae;o Tepidisphaerales;f WD2101-soil-group;g uncultured-bacterium;s uncultured-bacterium	0.6%	0.5%	0.7%
	p Planctomycetes;c Phycisphaerae;o Tepidisphaerales;f WD2101-soil-group;g uncultured-planctomycete;s uncultured-planctomycete	0.0%	0.0%	0.1%
	p Planctomycetes;c Phycisphaerae;o Tepidisphaerales;f WD2101-soil-group;g uncultured-soil-bacterium;s uncultured-soil-bacterium	0.0%	0.0%	0.0%
	p Planctomycetes;c Phycisphaerae;o mle1-8;Other;Other;Other	0.0%	0.0%	0.0%
	p Planctomycetes;c Phycisphaerae;o mle1-8;f uncultured-bacterium;g uncultured-bacterium;s uncultured-bacterium	0.1%	0.0%	0.1%
	p Planctomycetes;c Pla3-lineage;Other;Other;Other;Other	0.0%	0.0%	0.0%
	p Planctomycetes;c Pla4-lineage;Other;Other;Other;Other	0.0%	0.0%	0.0%
	p Planctomycetes;c Pla4-lineage;o uncultured-bacterium;f uncultured-bacterium;g uncultured-bacterium;s uncultured-bacterium	0.0%	0.0%	0.0%
	p Planctomycetes;c Pla4-lineage;o uncultured-prokaryote;f uncultured-prokaryote;g uncultured-prokaryote;s uncultured-prokaryote	0.0%	0.0%	0.0%
	p Planctomycetes;c Planctomycetacia;o Gemmatales;f Gemmataceae;Other;Other	0.0%	0.0%	0.0%
	p Planctomycetes;c Planctomycetacia;o Gemmatales;f Gemmataceae;g Fimbriiglobus;s uncultured-bacterium	0.0%	0.0%	0.0%
	p Planctomycetes;c Planctomycetacia;o Gemmatales;f Gemmataceae;g Gemmata;Other	0.0%	0.0%	0.0%
	p Planctomycetes;c Planctomycetacia;o Gemmatales;f Gemmataceae;g Gemmata;s uncultured-Gemmata-sp.	0.0%	0.0%	0.0%
	p Planctomycetes;c Planctomycetacia;o Gemmatales;f Gemmataceae;g Gemmata;s uncultured-bacterium	0.1%	0.0%	0.1%
	p Planctomycetes;c Planctomycetacia;o Gemmatales;f Gemmataceae;g uncultured;Other	0.0%	0.0%	0.0%
	p Planctomycetes;c Planctomycetacia;o Gemmatales;f Gemmataceae;g uncultured;s uncultured-Planctomycetia-bacterium	0.0%	0.0%	0.0%
	p Planctomycetes;c Planctomycetacia;o Gemmatales;f Gemmataceae;g uncultured;s uncultured-bacterium	0.0%	0.0%	0.0%
	p Planctomycetes;c Planctomycetacia;o Gemmatales;f Gemmataceae;g uncultured;s uncultured-prokaryote	0.0%	0.0%	0.0%
	p Planctomycetes;c Planctomycetacia;o Gemmatales;f Gemmataceae;g uncultured;s uncultured-soil-bacterium	0.0%	0.0%	0.0%
	p Planctomycetes;c Planctomycetacia;o Pirellulales;f Pirellulaceae;Other;Other	0.0%	0.0%	0.1%
	p Planctomycetes;c Planctomycetacia;o Pirellulales;f Pirellulaceae;g Bythopirellula;Other	0.0%	0.0%	0.0%
	p Planctomycetes;c Planctomycetacia;o Pirellulales;f Pirellulaceae;g Pirellula;Other	0.0%	0.0%	0.0%
	p Planctomycetes;c Planctomycetacia;o Pirellulales;f Pirellulaceae;g Pirellula;s uncultured-bacterium	0.1%	0.1%	0.1%
	p Planctomycetes;c Planctomycetacia;o Pirellulales;f Pirellulaceae;g Pirellula;s uncultured-soil-bacterium	0.0%	0.0%	0.0%
	p Planctomycetes;c Planctomycetacia;o Pirellulales;f Pirellulaceae;g uncultured;Other	0.0%	0.0%	0.0%
	p Planctomycetes;c Planctomycetacia;o Pirellulales;f Pirellulaceae;g uncultured;s uncultured-Pasteuria-sp.	0.0%	0.0%	0.0%
	p Planctomycetes;c Planctomycetacia;o Pirellulales;f Pirellulaceae;g uncultured;s uncultured-bacterium	0.0%	0.0%	0.0%
	p Planctomycetes;c Planctomycetacia;o Planctomycetales;Other;Other;Other	0.0%	0.0%	0.0%
	p Planctomycetes;c Planctomycetacia;o Planctomycetales;f Rubinisphaeraceae;g Planctomicrobium;s uncultured-bacterium	0.0%	0.0%	0.0%
	p Planctomycetes;c Planctomycetacia;o Planctomycetales;f Schlesneriaceae;g Planctopirus;Other	0.0%	0.0%	0.0%

	p Planctomycetes;c Planctomycetacia;o Planctomycetales;f uncultured;Other;Other	0.0%	0.0%	0.0%
	p Planctomycetes;c Planctomycetacia;o Planctomycetales;f uncultured;g uncultured-Planctomyces-sp.;s uncultured-Planctomyces-sp.	0.0%	0.0%	0.0%
	p Planctomycetes;c Planctomycetacia;o Planctomycetales;f uncultured;g uncultured-bacterium;s uncultured-bacterium	0.0%	0.0%	0.0%
	p Planctomycetes;c Planctomycetacia;o uncultured;f uncultured-bacterium;g uncultured-bacterium;s uncultured-bacterium	0.0%	0.0%	0.0%
	p Proteobacteria;Other;Other;Other;Other;Other	0.0%	0.0%	0.0%
	p Proteobacteria;c Alphaproteobacteria;Other;Other;Other;Other	1.8%	1.7%	2.0%
	p Proteobacteria;c Alphaproteobacteria;o Azospirillales;f Azospirillaceae;g Azospirillum;s uncultured-Azospirillum-sp.	0.0%	0.0%	0.0%
	p Proteobacteria;c Alphaproteobacteria;o Azospirillales;f Inquilinaceae;g Inquilinus;s Inquilinus-limosus	0.0%	0.0%	0.0%
	p Proteobacteria;c Alphaproteobacteria;o Azospirillales;f uncultured;g uncultured-bacterium;s uncultured-bacterium	0.0%	0.1%	0.0%
	p Proteobacteria;c Alphaproteobacteria;o Caulobacterales;Other;Other;Other	0.0%	0.0%	0.0%
	p Proteobacteria;c Alphaproteobacteria;o Caulobacterales;f Caulobacteraceae;Other;Other	0.0%	0.0%	0.0%
	p Proteobacteria;c Alphaproteobacteria;o Caulobacterales;f Caulobacteraceae;g Brevundimonas;Other	0.0%	0.0%	0.0%
	p Proteobacteria;c Alphaproteobacteria;o Caulobacterales;f Caulobacteraceae;g Phenylobacterium;Other	0.0%	0.0%	0.0%
	p Proteobacteria;c Alphaproteobacteria;o Caulobacterales;f Caulobacteraceae;g Phenylobacterium;s uncultured-bacterium	0.1%	0.1%	0.1%
	p Proteobacteria;c Alphaproteobacteria;o Caulobacterales;f Caulobacteraceae;g uncultured;Other	0.0%	0.0%	0.0%
	p Proteobacteria;c Alphaproteobacteria;o Caulobacterales;f Caulobacteraceae;g uncultured;s uncultured-alpha-proteobacterium	0.0%	0.0%	0.0%
	p Proteobacteria;c Alphaproteobacteria;o Caulobacterales;f Hyphomonadaceae;g Hirschia;s uncultured-bacterium	0.1%	0.1%	0.0%
	p Proteobacteria;c Alphaproteobacteria;o Caulobacterales;f Hyphomonadaceae;g uncultured;Other	0.0%	0.0%	0.0%
	p Proteobacteria;c Alphaproteobacteria;o Caulobacterales;f Hyphomonadaceae;g uncultured;s uncultured-bacterium	0.0%	0.1%	0.0%
	p Proteobacteria;c Alphaproteobacteria;o Caulobacterales;f Parvularculaceae;g Amphiplicatus;s uncultured-bacterium	0.0%	0.0%	0.0%
	p Proteobacteria;c Alphaproteobacteria;o Elsterales;f Elsteraceae;g Lacibacterium;s Lacibacterium-aquatile	0.0%	0.0%	0.0%
	p Proteobacteria;c Alphaproteobacteria;o Elsterales;f uncultured;Other;Other	0.0%	0.0%	0.0%
	p Proteobacteria;c Alphaproteobacteria;o Elsterales;f uncultured;g uncultured-Acetobacteraceae-bacterium;s uncultured-Acetobacteraceae-bacterium	0.0%	0.0%	0.0%
	p Proteobacteria;c Alphaproteobacteria;o Elsterales;f uncultured;g uncultured-alpha-proteobacterium;s uncultured-alpha-proteobacterium	0.0%	0.0%	0.0%
	p Proteobacteria;c Alphaproteobacteria;o Elsterales;f uncultured;g uncultured-bacterium;s uncultured-bacterium	0.0%	0.0%	0.0%
	p Proteobacteria;c Alphaproteobacteria;o Holosporales;f Holosporaceae;g uncultured;s uncultured-bacterium	0.0%	0.0%	0.0%
	p Proteobacteria;c Alphaproteobacteria;o Micropepsales;f Micropepsaceae;g uncultured;Other	0.0%	0.0%	0.0%
	p Proteobacteria;c Alphaproteobacteria;o Micropepsales;f Micropepsaceae;g uncultured;s uncultured-bacterium	0.0%	0.0%	0.0%
	p Proteobacteria;c Alphaproteobacteria;o Micropepsales;f Micropepsaceae;g uncultured;s uncultured-proteobacterium	0.1%	0.0%	0.1%
	p Proteobacteria;c Alphaproteobacteria;o Reyranellales;f Reyranellaceae;g Reyranella;s uncultured-bacterium	0.1%	0.1%	0.1%
	p Proteobacteria;c Alphaproteobacteria;o Reyranellales;f Reyranellaceae;g uncultured;Other	0.0%	0.0%	0.0%
	p Proteobacteria;c Alphaproteobacteria;o Reyranellales;f Reyranellaceae;g uncultured;s uncultured-bacterium	0.1%	0.2%	0.0%
	p Proteobacteria;c Alphaproteobacteria;o Reyranellales;f Reyranellaceae;g uncultured;s uncultured-soil-bacterium	0.0%	0.0%	0.0%
	p Proteobacteria;c Alphaproteobacteria;o Rhizobiales;Other;Other;Other	0.0%	0.1%	0.0%
	p Proteobacteria;c Alphaproteobacteria;o Rhizobiales;f A0839;g uncultured-bacterium;s uncultured-bacterium	0.0%	0.0%	0.1%
	p Proteobacteria;c Alphaproteobacteria;o Rhizobiales;f Amb-16S-1323;g uncultured-bacterium;s uncultured-bacterium	0.0%	0.0%	0.0%
	p Proteobacteria;c Alphaproteobacteria;o Rhizobiales;f Beijerinckiaceae;Other;Other	0.0%	0.0%	0.0%
	p Proteobacteria;c Alphaproteobacteria;o Rhizobiales;f Beijerinckiaceae;g Bosea;s uncultured-bacterium	0.0%	0.0%	0.0%
	p Proteobacteria;c Alphaproteobacteria;o Rhizobiales;f Beijerinckiaceae;g Microvirga;s Microvirga-massiliensis	0.0%	0.0%	0.0%
	p Proteobacteria;c Alphaproteobacteria;o Rhizobiales;f Beijerinckiaceae;g Microvirga;s uncultured-bacterium	0.1%	0.1%	0.1%
	p Proteobacteria;c Alphaproteobacteria;o Rhizobiales;f D05-2;g uncultured-bacterium;s uncultured-bacterium	0.0%	0.0%	0.0%
	p Proteobacteria;c Alphaproteobacteria;o Rhizobiales;f Devosiaceae;Other;Other	0.1%	0.1%	0.1%
	p Proteobacteria;c Alphaproteobacteria;o Rhizobiales;f Devosiaceae;g Devosia;Other	0.0%	0.0%	0.0%
	p Proteobacteria;c Alphaproteobacteria;o Rhizobiales;f Devosiaceae;g uncultured;s uncultured-bacterium	0.1%	0.2%	0.0%
	p Proteobacteria;c Alphaproteobacteria;o Rhizobiales;f Hyphomicrobiaceae;Other;Other	0.0%	0.0%	0.0%
	p Proteobacteria;c Alphaproteobacteria;o Rhizobiales;f Hyphomicrobiaceae;g Hyphomicrobium;s uncultured-bacterium	0.0%	0.0%	0.0%
	p Proteobacteria;c Alphaproteobacteria;o Rhizobiales;f Hyphomicrobiaceae;g Pedomicrobium;Other	0.0%	0.0%	0.0%
	p Proteobacteria;c Alphaproteobacteria;o Rhizobiales;f Hyphomicrobiaceae;g Pedomicrobium;s uncultured-bacterium	0.3%	0.5%	0.1%
	p Proteobacteria;c Alphaproteobacteria;o Rhizobiales;f Hyphomicrobiaceae;g uncultured;s uncultured-bacterium	0.0%	0.0%	0.0%
	p Proteobacteria;c Alphaproteobacteria;o Rhizobiales;f KF-JG30-B3;Other;Other	0.0%	0.0%	0.0%
	p Proteobacteria;c Alphaproteobacteria;o Rhizobiales;f KF-JG30-B3;g uncultured-bacterium;s uncultured-bacterium	0.8%	0.7%	0.9%
	p Proteobacteria;c Alphaproteobacteria;o Rhizobiales;f Labraceae;g Labrys;Other	0.0%	0.0%	0.0%
	p Proteobacteria;c Alphaproteobacteria;o Rhizobiales;f Labraceae;g Labrys;s uncultured-bacterium	0.0%	0.0%	0.0%
	p Proteobacteria;c Alphaproteobacteria;o Rhizobiales;f Methyloligellaceae;Other;Other	0.0%	0.1%	0.0%
	p Proteobacteria;c Alphaproteobacteria;o Rhizobiales;f Methyloligellaceae;g uncultured;Other	0.3%	0.3%	0.3%
	p Proteobacteria;c Alphaproteobacteria;o Rhizobiales;f Methyloligellaceae;g uncultured;s uncultured-bacterium	0.0%	0.0%	0.0%
	p Proteobacteria;c Alphaproteobacteria;o Rhizobiales;f Rhizobiaceae;Other;Other	0.1%	0.1%	0.1%
	p Proteobacteria;c Alphaproteobacteria;o Rhizobiales;f Rhizobiaceae;g Allorhizobium-Neorhizobium-Pararhizobium-Rhizobium;Other	0.0%	0.0%	0.0%
	p Proteobacteria;c Alphaproteobacteria;o Rhizobiales;f Rhizobiaceae;g Allorhizobium-Neorhizobium-Pararhizobium-Rhizobium;s Rhizobium-etli	0.3%	0.2%	0.3%
	p Proteobacteria;c Alphaproteobacteria;o Rhizobiales;f Rhizobiaceae;g Ensifer;s Ensifer-adhaerens	0.3%	0.5%	0.1%
	p Proteobacteria;c Alphaproteobacteria;o Rhizobiales;f Rhizobiaceae;g Mesorhizobium;Other	0.0%	0.0%	0.0%
	p Proteobacteria;c Alphaproteobacteria;o Rhizobiales;f Rhizobiales-Incertae-Sedis;g Bauldia;Other	0.0%	0.0%	0.0%
	p Proteobacteria;c Alphaproteobacteria;o Rhizobiales;f Rhizobiales-Incertae-Sedis;g Bauldia;s uncultured-bacterium	0.1%	0.1%	0.1%
	p Proteobacteria;c Alphaproteobacteria;o Rhizobiales;f Rhizobiales-Incertae-Sedis;g uncultured;Other	0.0%	0.0%	0.0%

p Proteobacteria;c Alphaproteobacteria;o Rhizobiales;f Rhizobiales-Incertae-Sedis;g uncultured;s uncultured-bacterium	0.2%	0.1%	0.2%
p Proteobacteria;c Alphaproteobacteria;o Rhizobiales;f Rhodomicrobiaceae;g Rhodomicrobium;s uncultured-bacterium	0.0%	0.0%	0.0%
p Proteobacteria;c Alphaproteobacteria;o Rhizobiales;f Xanthobacteraceae;Other;Other	1.0%	1.0%	1.0%
p Proteobacteria;c Alphaproteobacteria;o Rhizobiales;f Xanthobacteraceae;g Ancylobacter;Other	0.0%	0.0%	0.0%
p Proteobacteria;c Alphaproteobacteria;o Rhizobiales;f Xanthobacteraceae;g Bradyrhizobium;Other	0.5%	0.5%	0.4%
p Proteobacteria;c Alphaproteobacteria;o Rhizobiales;f Xanthobacteraceae;g Pseudolabrys;s uncultured-bacterium	0.1%	0.1%	0.1%
p Proteobacteria;c Alphaproteobacteria;o Rhizobiales;f Xanthobacteraceae;g Rhodoplanes;s uncultured-bacterium	0.1%	0.1%	0.1%
p Proteobacteria;c Alphaproteobacteria;o Rhizobiales;f Xanthobacteraceae;g uncultured;Other	0.1%	0.2%	0.1%
p Proteobacteria;c Alphaproteobacteria;o Rhizobiales;f Xanthobacteraceae;g uncultured;s uncultured-bacterium	0.6%	0.6%	0.6%
p Proteobacteria;c Alphaproteobacteria;o Rhizobiales;f uncultured;Other;Other	0.0%	0.0%	0.0%
p Proteobacteria;c Alphaproteobacteria;o Rhizobiales;f uncultured;g uncultured-Rhizobiales-bacterium;s uncultured-Rhizobiales-bacterium	0.0%	0.0%	0.0%
p Proteobacteria;c Alphaproteobacteria;o Rhizobiales;f uncultured;g uncultured-bacterium;s uncultured-bacterium	0.1%	0.1%	0.1%
p Proteobacteria;c Alphaproteobacteria;o Rhodobacterales;f Rhodobacteraceae;Other;Other	0.0%	0.0%	0.0%
p Proteobacteria;c Alphaproteobacteria;o Rhodobacterales;f Rhodobacteraceae;g uncultured;Other	0.0%	0.0%	0.0%
p Proteobacteria;c Alphaproteobacteria;o Rhodospirillales;f Rhodospirillaceae;g uncultured;s uncultured-bacterium	0.0%	0.0%	0.1%
p Proteobacteria;c Alphaproteobacteria;o Rhodospirillales;f uncultured;Other;Other	0.0%	0.0%	0.0%
p Proteobacteria;c Alphaproteobacteria;o Rhodospirillales;f uncultured;g uncultured-Rhodospirillaceae-bacterium;s uncultured-Rhodospirillaceae-bacterium	0.0%	0.0%	0.0%
p Proteobacteria;c Alphaproteobacteria;o Rhodovibrionales;f Fodinicurvataceae;g uncultured;Other	0.0%	0.0%	0.0%
p Proteobacteria;c Alphaproteobacteria;o Sneathiellales;f Sneathiellaceae;g uncultured;s uncultured-bacterium	0.0%	0.0%	0.0%
p Proteobacteria;c Alphaproteobacteria;o Sphingomonadales;f Sphingomonadaceae;Other;Other	0.1%	0.2%	0.1%
p Proteobacteria;c Alphaproteobacteria;o Sphingomonadales;f Sphingomonadaceae;g Altererythrobacter;Other	0.0%	0.0%	0.0%
p Proteobacteria;c Alphaproteobacteria;o Sphingomonadales;f Sphingomonadaceae;g Altererythrobacter;s uncultured-bacterium	0.1%	0.1%	0.1%
p Proteobacteria;c Alphaproteobacteria;o Sphingomonadales;f Sphingomonadaceae;g Novosphingobium;Other	0.2%	0.3%	0.1%
p Proteobacteria;c Alphaproteobacteria;o Sphingomonadales;f Sphingomonadaceae;g Novosphingobium;s uncultured-bacterium	0.0%	0.0%	0.0%
p Proteobacteria;c Alphaproteobacteria;o Sphingomonadales;f Sphingomonadaceae;g Sphingobium;s Sphingobium-sp.-YL23	0.1%	0.1%	0.1%
p Proteobacteria;c Alphaproteobacteria;o Sphingomonadales;f Sphingomonadaceae;g Sphingomonas;Other	1.3%	1.6%	1.0%
p Proteobacteria;c Alphaproteobacteria;o Sphingomonadales;f Sphingomonadaceae;g Sphingomonas;s uncultured-Sphingomonas-sp.	0.0%	0.0%	0.0%
p Proteobacteria;c Alphaproteobacteria;o Sphingomonadales;f Sphingomonadaceae;g uncultured;Other	0.0%	0.0%	0.0%
p Proteobacteria;c Alphaproteobacteria;o Sphingomonadales;f Sphingomonadaceae;g uncultured;s uncultured-bacterium	0.0%	0.0%	0.0%
p Proteobacteria;c Alphaproteobacteria;o Tistrellales;f Geminicoccaceae;g Candidatus-Alysiosphaera;s uncultured-bacterium	0.0%	0.0%	0.0%
p Proteobacteria;c Alphaproteobacteria;o Tistrellales;f Geminicoccaceae;g uncultured;s uncultured-bacterium	0.0%	0.0%	0.0%
p Proteobacteria;c Alphaproteobacteria;o uncultured;Other;Other;Other	0.2%	0.2%	0.2%
p Proteobacteria;c Alphaproteobacteria;o uncultured;f uncultured-Acetobacteraceae-bacterium;g uncultured-Acetobacteraceae-bacterium;s uncultured-Acetobacteraceae-bacterium	0.0%	0.0%	0.0%
p Proteobacteria;c Alphaproteobacteria;o uncultured;f uncultured-Rhodospirillaceae-bacterium;g uncultured-Rhodospirillaceae-bacterium;s uncultured-Rhodospirillaceae-bacterium	0.0%	0.0%	0.0%
p Proteobacteria;c Alphaproteobacteria;o uncultured;f uncultured-Rhodospirillales-bacterium;g uncultured-Rhodospirillales-bacterium;s uncultured-Rhodospirillales-bacterium	0.0%	0.0%	0.0%
p Proteobacteria;c Alphaproteobacteria;o uncultured;f uncultured-Stella-sp.;g uncultured-Stella-sp.;s uncultured-Stella-sp.	0.0%	0.0%	0.0%
p Proteobacteria;c Alphaproteobacteria;o uncultured;f uncultured-bacterium;g uncultured-bacterium;s uncultured-bacterium	0.1%	0.1%	0.1%
p Proteobacteria;c Alphaproteobacteria;o uncultured;f uncultured-soil-bacterium;g uncultured-soil-bacterium;s uncultured-soil-bacterium	0.0%	0.1%	0.0%
p Proteobacteria;c Deltaproteobacteria;Other;Other;Other;Other	0.2%	0.3%	0.2%
p Proteobacteria;c Deltaproteobacteria;o Bdellovibrionales;f Bdellovibrionaceae;g Bdellovibrio;Other	0.0%	0.0%	0.0%
p Proteobacteria;c Deltaproteobacteria;o Bdellovibrionales;f Bdellovibrionaceae;g Bdellovibrio;s Bdellovibrionales-bacterium-RBG_16_40_8	0.0%	0.0%	0.0%
p Proteobacteria;c Deltaproteobacteria;o Bdellovibrionales;f Bdellovibrionaceae;g Bdellovibrio;s uncultured-bacterium	0.0%	0.1%	0.0%
p Proteobacteria;c Deltaproteobacteria;o Desulfarculales;f Desulfarculaceae;g uncultured;Other	0.1%	0.1%	0.1%
p Proteobacteria;c Deltaproteobacteria;o Desulfarculales;f Desulfarculaceae;g uncultured;s uncultured-Desulfuromonadales-bacterium	0.0%	0.0%	0.0%
p Proteobacteria;c Deltaproteobacteria;o Desulfarculales;f Desulfarculaceae;g uncultured;s uncultured-bacterium	0.1%	0.1%	0.1%
p Proteobacteria;c Deltaproteobacteria;o Desulfuromonadales;Other;Other;Other	0.0%	0.0%	0.0%
p Proteobacteria;c Deltaproteobacteria;o Desulfuromonadales;f Geobacteraceae;g Geobacter;Other	0.0%	0.0%	0.0%
p Proteobacteria;c Deltaproteobacteria;o Desulfuromonadales;f Geobacteraceae;g Geobacter;s uncultured-bacterium	0.0%	0.0%	0.0%
p Proteobacteria;c Deltaproteobacteria;o Desulfuromonadales;f Geobacteraceae;g Geobacter;s uncultured-delta-proteobacterium	0.0%	0.0%	0.0%
p Proteobacteria;c Deltaproteobacteria;o MBNT15;Other;Other;Other	0.1%	0.0%	0.2%
p Proteobacteria;c Deltaproteobacteria;o MBNT15;f uncultured-bacterium;g uncultured-bacterium;s uncultured-bacterium	0.0%	0.0%	0.0%
p Proteobacteria;c Deltaproteobacteria;o MBNT15;f uncultured-proteobacterium;g uncultured-proteobacterium;s uncultured-proteobacterium	0.0%	0.0%	0.1%
p Proteobacteria;c Deltaproteobacteria;o Myxococcales;Other;Other;Other	0.1%	0.1%	0.0%
p Proteobacteria;c Deltaproteobacteria;o Myxococcales;f Archangiaceae;g Anaeromyxobacter;Other	0.0%	0.0%	0.0%

p Proteobacteria;c Deltaproteobacteria;o Myxococcales;f Archangiaceae;g Anaeromyxobacter;s uncultured-Anaeromyxobacter-sp.	0.0%	0.0%	0.0%
p Proteobacteria;c Deltaproteobacteria;o Myxococcales;f Archangiaceae;g Anaeromyxobacter;s uncultured-bacterium	0.0%	0.0%	0.0%
p Proteobacteria;c Deltaproteobacteria;o Myxococcales;f Archangiaceae;g Anaeromyxobacter;s uncultured-delta-proteobacterium	0.0%	0.0%	0.0%
p Proteobacteria;c Deltaproteobacteria;o Myxococcales;f BIrii41;Other;Other	0.0%	0.0%	0.0%
p Proteobacteria;c Deltaproteobacteria;o Myxococcales;f BIrii41;g uncultured-bacterium;s uncultured-bacterium	0.1%	0.1%	0.1%
p Proteobacteria;c Deltaproteobacteria;o Myxococcales;f Blfdi19;Other;Other	0.0%	0.0%	0.0%
p Proteobacteria;c Deltaproteobacteria;o Myxococcales;f Blfdi19;g uncultured-bacterium;s uncultured-bacterium	0.0%	0.0%	0.0%
p Proteobacteria;c Deltaproteobacteria;o Myxococcales;f Eel-36e1D6;Other;Other	0.0%	0.0%	0.0%
p Proteobacteria;c Deltaproteobacteria;o Myxococcales;f Haliangiaceae;g Haliangium;Other	0.2%	0.2%	0.2%
p Proteobacteria;c Deltaproteobacteria;o Myxococcales;f Haliangiaceae;g Haliangium;s delta-proteobacterium-LWH25	0.0%	0.0%	0.0%
p Proteobacteria;c Deltaproteobacteria;o Myxococcales;f Haliangiaceae;g Haliangium;s uncultured-Kofleriaceae-bacterium	0.0%	0.0%	0.0%
p Proteobacteria;c Deltaproteobacteria;o Myxococcales;f Haliangiaceae;g Haliangium;s uncultured-Nannocystineae-bacterium	0.0%	0.0%	0.0%
p Proteobacteria;c Deltaproteobacteria;o Myxococcales;f Haliangiaceae;g Haliangium;s uncultured-bacterium	0.0%	0.1%	0.0%
p Proteobacteria;c Deltaproteobacteria;o Myxococcales;f Haliangiaceae;g Haliangium;s uncultured-prokaryote	0.0%	0.0%	0.0%
p Proteobacteria;c Deltaproteobacteria;o Myxococcales;f Nannocystaceae;Other;Other	0.0%	0.0%	0.0%
p Proteobacteria;c Deltaproteobacteria;o Myxococcales;f Nannocystaceae;g Nannocystis;Other	0.0%	0.0%	0.0%
p Proteobacteria;c Deltaproteobacteria;o Myxococcales;f Nannocystaceae;g Nannocystis;s Nannocystis-pusilla	0.0%	0.0%	0.0%
p Proteobacteria;c Deltaproteobacteria;o Myxococcales;f Nannocystaceae;g Nannocystis;s uncultured-bacterium	0.0%	0.0%	0.0%
p Proteobacteria;c Deltaproteobacteria;o Myxococcales;f Nannocystaceae;g uncultured;s uncultured-bacterium	0.0%	0.0%	0.0%
p Proteobacteria;c Deltaproteobacteria;o Myxococcales;f Nannocystaceae;g uncultured-bacterium;s uncultured-bacterium	0.0%	0.0%	0.0%
p Proteobacteria;c Deltaproteobacteria;o Myxococcales;f P3OB-42;Other;Other	0.0%	0.0%	0.0%
p Proteobacteria;c Deltaproteobacteria;o Myxococcales;f P3OB-42;g uncultured-bacterium;s uncultured-bacterium	0.0%	0.0%	0.0%
p Proteobacteria;c Deltaproteobacteria;o Myxococcales;f Phaselicystidaceae;g Phaselicystis;Other	0.0%	0.0%	0.0%
p Proteobacteria;c Deltaproteobacteria;o Myxococcales;f Phaselicystidaceae;g Phaselicystis;s uncultured-bacterium	0.0%	0.0%	0.0%
p Proteobacteria;c Deltaproteobacteria;o Myxococcales;f Polyangiaceae;Other;Other	0.0%	0.0%	0.0%
p Proteobacteria;c Deltaproteobacteria;o Myxococcales;f Polyangiaceae;g Pajaroellobacter;Other	0.0%	0.0%	0.0%
p Proteobacteria;c Deltaproteobacteria;o Myxococcales;f Polyangiaceae;g Pajaroellobacter;s uncultured-bacterium	0.0%	0.0%	0.0%
p Proteobacteria;c Deltaproteobacteria;o Myxococcales;f Polyangiaceae;g Polyangium;s uncultured-bacterium	0.0%	0.0%	0.0%
p Proteobacteria;c Deltaproteobacteria;o Myxococcales;f Sandaracinaceae;g Sandaracinus;Other	0.0%	0.0%	0.0%
p Proteobacteria;c Deltaproteobacteria;o Myxococcales;f Sandaracinaceae;g Sandaracinus;s uncultured-bacterium	0.0%	0.0%	0.0%
p Proteobacteria;c Deltaproteobacteria;o Myxococcales;f Sandaracinaceae;g uncultured;Other	0.0%	0.0%	0.0%
p Proteobacteria;c Deltaproteobacteria;o Myxococcales;f Sandaracinaceae;g uncultured;s delta-proteobacterium-LX33	0.0%	0.0%	0.0%
p Proteobacteria;c Deltaproteobacteria;o Myxococcales;f Sandaracinaceae;g uncultured;s uncultured-bacterium	0.0%	0.0%	0.0%
p Proteobacteria;c Deltaproteobacteria;o Myxococcales;f Sandaracinaceae;g uncultured;s uncultured-soil-bacterium	0.0%	0.0%	0.0%
p Proteobacteria;c Deltaproteobacteria;o Myxococcales;f UASB-TL25;g uncultured-bacterium;s uncultured-bacterium	0.0%	0.0%	0.0%
p Proteobacteria;c Deltaproteobacteria;o Myxococcales;f Vulgatibacteraceae;g Vulgatibacter;s uncultured-Hyalangium-sp.	0.0%	0.0%	0.0%
p Proteobacteria;c Deltaproteobacteria;o Myxococcales;f Vulgatibacteraceae;g Vulgatibacter;s uncultured-bacterium	0.0%	0.0%	0.0%
p Proteobacteria;c Deltaproteobacteria;o Myxococcales;f bacteriap25;Other;Other	0.5%	0.5%	0.5%
p Proteobacteria;c Deltaproteobacteria;o Myxococcales;f bacteriap25;g uncultured-delta-proteobacterium;s uncultured-delta-proteobacterium	0.0%	0.0%	0.0%
p Proteobacteria;c Deltaproteobacteria;o Myxococcales;f bacteriap25;g uncultured-proteobacterium;s uncultured-proteobacterium	0.1%	0.1%	0.1%
p Proteobacteria;c Deltaproteobacteria;o Myxococcales;f bacteriap25;g uncultured-soil-bacterium;s uncultured-soil-bacterium	0.1%	0.1%	0.0%
p Proteobacteria;c Deltaproteobacteria;o Myxococcales;f bacteriap25;g unidentified;s unidentified	0.0%	0.0%	0.0%
p Proteobacteria;c Deltaproteobacteria;o Myxococcales;f mle1-27;Other;Other	0.0%	0.0%	0.0%
p Proteobacteria;c Deltaproteobacteria;o Myxococcales;f mle1-27;g uncultured-delta-proteobacterium;s uncultured-delta-proteobacterium	0.0%	0.0%	0.0%
p Proteobacteria;c Deltaproteobacteria;o Myxococcales;f uncultured;Other;Other	0.0%	0.0%	0.0%
p Proteobacteria;c Deltaproteobacteria;o Myxococcales;f uncultured;g uncultured-bacterium;s uncultured-bacterium	0.0%	0.0%	0.0%
p Proteobacteria;c Deltaproteobacteria;o NB1-j;Other;Other;Other	0.0%	0.0%	0.0%
p Proteobacteria;c Deltaproteobacteria;o NB1-j;f uncultured-Green-Bay-ferromanganous-micronodule-bacterium-MND4;g uncultured-Green-Bay-ferromanganous-micronodule-bacterium-MND4;s uncultured-Green-Bay-ferromanganous-micronodule-bacterium-MND4	0.0%	0.0%	0.0%
p Proteobacteria;c Deltaproteobacteria;o NB1-j;f uncultured-bacterium;g uncultured-bacterium;s uncultured-bacterium	0.3%	0.4%	0.2%
p Proteobacteria;c Deltaproteobacteria;o NB1-j;f uncultured-proteobacterium;g uncultured-proteobacterium;s uncultured-proteobacterium	0.0%	0.0%	0.0%
p Proteobacteria;c Deltaproteobacteria;o NB1-j;f uncultured-soil-bacterium;g uncultured-soil-bacterium;s uncultured-soil-bacterium	0.0%	0.0%	0.0%
p Proteobacteria;c Deltaproteobacteria;o Oligoflexales;f Oligoflexaceae;g Oligoflexus;s uncultured-Desulfocurvus-sp.	0.0%	0.0%	0.0%
p Proteobacteria;c Deltaproteobacteria;o RCP2-54;f uncultured-bacterium;g uncultured-bacterium;s uncultured-bacterium	0.0%	0.0%	0.0%
p Proteobacteria;c Deltaproteobacteria;o RCP2-54;f uncultured-prokaryote;g uncultured-prokaryote;s uncultured-prokaryote	0.0%	0.0%	0.0%
p Proteobacteria;c Deltaproteobacteria;o SAR324-clade(Marine-group-B);f bacterium-enrichment-culture-clone-B30(2011);g bacterium-enrichment-culture-clone-B30(2011);s bacterium-enrichment-culture-clone-B30(2011)	0.0%	0.0%	0.0%
p Proteobacteria;c Deltaproteobacteria;o Syntrophobacterales;f Syntrophaceae;g uncultured;Other	0.0%	0.0%	0.0%

	p Proteobacteria;c Deltaproteobacteria;o Syntrophobacterales;f Syntrophaceae;g uncultured;s uncultured-Myxococcales-bacterium	0.0%	0.0%	0.0%
	p Proteobacteria;c Gammaproteobacteria;Other;Other;Other;Other	0.5%	0.6%	0.4%
	p Proteobacteria;c Gammaproteobacteria;o Acidiferrobacterales;f Acidiferrobacteraceae;g Sulfurifustis;Other	0.0%	0.0%	0.0%
	p Proteobacteria;c Gammaproteobacteria;o Acidiferrobacterales;f Acidiferrobacteraceae;g Sulfurifustis;s uncultured-bacterium	0.0%	0.0%	0.0%
	p Proteobacteria;c Gammaproteobacteria;o Aeromonadales;f Aeromonadaceae;g Aeromonas;Other	0.0%	0.0%	0.0%
	p Proteobacteria;c Gammaproteobacteria;o Betaproteobacteriales;Other;Other;Other	1.7%	1.4%	2.1%
	p Proteobacteria;c Gammaproteobacteria;o Betaproteobacteriales;f A21b;Other;Other	0.0%	0.0%	0.0%
	p Proteobacteria;c Gammaproteobacteria;o Betaproteobacteriales;f A21b;g uncultured-bacterium;s uncultured-bacterium	0.0%	0.0%	0.0%
	p Proteobacteria;c Gammaproteobacteria;o Betaproteobacteriales;f B1-7BS;Other;Other	0.0%	0.0%	0.0%
	p Proteobacteria;c Gammaproteobacteria;o Betaproteobacteriales;f B1-7BS;g uncultured-bacterium;s uncultured-bacterium	0.1%	0.1%	0.1%
	p Proteobacteria;c Gammaproteobacteria;o Betaproteobacteriales;f Burkholderiaceae;Other;Other	0.2%	0.2%	0.3%
	p Proteobacteria;c Gammaproteobacteria;o Betaproteobacteriales;f Burkholderiaceae;g Bordetella;Other	0.0%	0.0%	0.0%
	p Proteobacteria;c Gammaproteobacteria;o Betaproteobacteriales;f Burkholderiaceae;g Burkholderia-Caballeronia-Paraburkholderia;Other	0.0%	0.0%	0.1%
	p Proteobacteria;c Gammaproteobacteria;o Betaproteobacteriales;f Burkholderiaceae;g Comamonas;s uncultured-prokaryote	0.1%	0.1%	0.0%
	p Proteobacteria;c Gammaproteobacteria;o Betaproteobacteriales;f Burkholderiaceae;g Cupriavidus;Other	0.0%	0.0%	0.0%
	p Proteobacteria;c Gammaproteobacteria;o Betaproteobacteriales;f Burkholderiaceae;g Cupriavidus;s Cupriavidus-necator	0.4%	0.7%	0.1%
	p Proteobacteria;c Gammaproteobacteria;o Betaproteobacteriales;f Burkholderiaceae;g Cupriavidus;s uncultured-bacterium	0.0%	0.0%	0.0%
	p Proteobacteria;c Gammaproteobacteria;o Betaproteobacteriales;f Burkholderiaceae;g Hydrogenophaga;s uncultured-bacterium	0.0%	0.0%	0.0%
	p Proteobacteria;c Gammaproteobacteria;o Betaproteobacteriales;f Burkholderiaceae;g Massilia;Other	0.0%	0.0%	0.0%
	p Proteobacteria;c Gammaproteobacteria;o Betaproteobacteriales;f Burkholderiaceae;g Noviherbaspirillum;s uncultured-bacterium	0.0%	0.0%	0.0%
	p Proteobacteria;c Gammaproteobacteria;o Betaproteobacteriales;f Burkholderiaceae;g Ramlibacter;Other	0.1%	0.2%	0.1%
	p Proteobacteria;c Gammaproteobacteria;o Betaproteobacteriales;f Burkholderiaceae;g Ramlibacter;s uncultured-bacterium	0.0%	0.0%	0.0%
	p Proteobacteria;c Gammaproteobacteria;o Betaproteobacteriales;f Burkholderiaceae;g Rhizobacter;Other	0.0%	0.0%	0.0%
	p Proteobacteria;c Gammaproteobacteria;o Betaproteobacteriales;f Burkholderiaceae;g Rhizobacter;s uncultured-bacterium	0.0%	0.0%	0.0%
	p Proteobacteria;c Gammaproteobacteria;o Betaproteobacteriales;f Burkholderiaceae;g Sphaerotilus;Other	0.0%	0.0%	0.0%
	p Proteobacteria;c Gammaproteobacteria;o Betaproteobacteriales;f Burkholderiaceae;g Sphaerotilus;s uncultured-Burkholderiales-bacterium	0.0%	0.0%	0.0%
	p Proteobacteria;c Gammaproteobacteria;o Betaproteobacteriales;f Burkholderiaceae;g Variovorax;Other	0.1%	0.1%	0.0%
	p Proteobacteria;c Gammaproteobacteria;o Betaproteobacteriales;f Burkholderiaceae;g uncultured;Other	0.1%	0.0%	0.1%
	p Proteobacteria;c Gammaproteobacteria;o Betaproteobacteriales;f Chromobacteriaceae;Other;Other	0.0%	0.0%	0.0%
	p Proteobacteria;c Gammaproteobacteria;o Betaproteobacteriales;f Chromobacteriaceae;g Paludibacterium;Other	0.0%	0.0%	0.0%
	p Proteobacteria;c Gammaproteobacteria;o Betaproteobacteriales;f Rhodocyclaceae;Other;Other	0.3%	0.1%	0.4%
	p Proteobacteria;c Gammaproteobacteria;o Betaproteobacteriales;f Rhodocyclaceae;g Azoarcus;s uncultured-bacterium	0.0%	0.0%	0.0%
	p Proteobacteria;c Gammaproteobacteria;o Betaproteobacteriales;f Rhodocyclaceae;g Thauera;Other	0.0%	0.0%	0.0%
	p Proteobacteria;c Gammaproteobacteria;o Betaproteobacteriales;f Rhodocyclaceae;g Thauera;s uncultured-bacterium	0.0%	0.0%	0.0%
	p Proteobacteria;c Gammaproteobacteria;o Betaproteobacteriales;f SC-I-84;Other;Other	0.3%	0.3%	0.2%
	p Proteobacteria;c Gammaproteobacteria;o Betaproteobacteriales;f SC-I-84;g uncultured-bacterium;s uncultured-bacterium	0.2%	0.1%	0.2%
	p Proteobacteria;c Gammaproteobacteria;o Betaproteobacteriales;f SC-I-84;g uncultured-beta-proteobacterium;s uncultured-beta-proteobacterium	0.1%	0.1%	0.2%
	p Proteobacteria;c Gammaproteobacteria;o Betaproteobacteriales;f TRA3-20;Other;Other	0.6%	0.7%	0.5%
	p Proteobacteria;c Gammaproteobacteria;o Betaproteobacteriales;f TRA3-20;g uncultured-bacterium;s uncultured-bacterium	0.1%	0.1%	0.2%
	p Proteobacteria;c Gammaproteobacteria;o Betaproteobacteriales;f TRA3-20;g uncultured-gamma-proteobacterium;s uncultured-gamma-proteobacterium	0.0%	0.0%	0.0%
	p Proteobacteria;c Gammaproteobacteria;o CCD24;f uncultured-bacterium;g uncultured-bacterium;s uncultured-bacterium	0.2%	0.3%	0.1%
	p Proteobacteria;c Gammaproteobacteria;o Cellvibrionales;Other;Other;Other	0.0%	0.0%	0.0%
	p Proteobacteria;c Gammaproteobacteria;o Cellvibrionales;f Halieaceae;Other;Other	0.0%	0.0%	0.0%
	p Proteobacteria;c Gammaproteobacteria;o Diplorickettsiales;f Diplorickettsiaceae;g Aquicella;s uncultured-bacterium	0.0%	0.0%	0.0%
	p Proteobacteria;c Gammaproteobacteria;o EPR3968-O8a-Bc78;f uncultured-gamma-proteobacterium;g uncultured-gamma-proteobacterium;s uncultured-gamma-proteobacterium	0.0%	0.0%	0.0%
	p Proteobacteria;c Gammaproteobacteria;o Enterobacteriales;f Enterobacteriaceae;Other;Other	0.0%	0.0%	0.0%
	p Proteobacteria;c Gammaproteobacteria;o Enterobacteriales;f Enterobacteriaceae;g Pantoea;Other	0.0%	0.0%	0.0%
	p Proteobacteria;c Gammaproteobacteria;o Gammaproteobacteria-Incertae-Sedis;f Unknown-Family;g uncultured;Other	0.0%	0.0%	0.0%
	p Proteobacteria;c Gammaproteobacteria;o JG36-GS-52;f uncultured-bacterium;g uncultured-bacterium;s uncultured-bacterium	0.0%	0.0%	0.0%
	p Proteobacteria;c Gammaproteobacteria;o JG36-TzT-191;Other;Other;Other	0.0%	0.0%	0.0%
	p Proteobacteria;c Gammaproteobacteria;o JG36-TzT-191;f uncultured-bacterium;g uncultured-bacterium;s uncultured-bacterium	0.0%	0.0%	0.0%
	p Proteobacteria;c Gammaproteobacteria;o KI89A-clade;Other;Other;Other	0.0%	0.0%	0.0%
	p Proteobacteria;c Gammaproteobacteria;o PLTA13;Other;Other;Other	0.0%	0.0%	0.0%
	p Proteobacteria;c Gammaproteobacteria;o PLTA13;f uncultured-bacterium;g uncultured-bacterium;s uncultured-bacterium	0.7%	1.2%	0.1%
	p Proteobacteria;c Gammaproteobacteria;o PLTA13;f uncultured-proteobacterium;g uncultured-proteobacterium;s uncultured-proteobacterium	0.0%	0.0%	0.0%
	p Proteobacteria;c Gammaproteobacteria;o Pseudomonadales;f Moraxellaceae;g Acinetobacter;s Acinetobacter-calcoaceticus	0.0%	0.0%	0.0%

p Proteobacteria;c Gammaproteobacteria;o Pseudomonadales;f Moraxellaceae;g uncultured;Other	0.0%	0.0%	0.0%
p Proteobacteria;c Gammaproteobacteria;o Pseudomonadales;f Moraxellaceae;g uncultured;s gamma-proteobacterium-Y-134	0.0%	0.0%	0.0%
p Proteobacteria;c Gammaproteobacteria;o Pseudomonadales;f Pseudomonadaceae;Other;Other	0.0%	0.0%	0.0%
p Proteobacteria;c Gammaproteobacteria;o Pseudomonadales;f Pseudomonadaceae;g Azotobacter;Other	0.0%	0.0%	0.0%
p Proteobacteria;c Gammaproteobacteria;o Pseudomonadales;f Pseudomonadaceae;g Pseudomonas;Other	0.3%	0.5%	0.1%
p Proteobacteria;c Gammaproteobacteria;o Pseudomonadales;f Pseudomonadaceae;g Pseudomonas;s Pseudomonas-citronellolis	0.0%	0.0%	0.0%
p Proteobacteria;c Gammaproteobacteria;o Pseudomonadales;f Pseudomonadaceae;g Pseudomonas;s Pseudomonas-plecoglossicida	0.0%	0.0%	0.0%
p Proteobacteria;c Gammaproteobacteria;o Pseudomonadales;f Pseudomonadaceae;g Pseudomonas;s Pseudomonas-resinovorans	0.0%	0.1%	0.0%
p Proteobacteria;c Gammaproteobacteria;o Pseudomonadales;f Pseudomonadaceae;g Pseudomonas;s Pseudomonas-sp.-enrichment-culture-clone-HSL33	0.0%	0.0%	0.0%
p Proteobacteria;c Gammaproteobacteria;o R7C24;Other;Other;Other	0.0%	0.0%	0.0%
p Proteobacteria;c Gammaproteobacteria;o R7C24;f uncultured-bacterium;g uncultured-bacterium;s uncultured-bacterium	0.0%	0.0%	0.0%
p Proteobacteria;c Gammaproteobacteria;o Salinisphaerales;Other;Other;Other	0.0%	0.0%	0.0%
p Proteobacteria;c Gammaproteobacteria;o Salinisphaerales;f Solimonadaceae;Other;Other	0.0%	0.0%	0.0%
p Proteobacteria;c Gammaproteobacteria;o Steroidobacterales;f Steroidobacteraceae;g uncultured;Other	0.9%	1.3%	0.5%
p Proteobacteria;c Gammaproteobacteria;o Steroidobacterales;f Steroidobacteraceae;g uncultured;s uncultured-bacterium	0.4%	0.6%	0.1%
p Proteobacteria;c Gammaproteobacteria;o Steroidobacterales;f Steroidobacteraceae;g uncultured;s uncultured-gamma-proteobacterium	0.1%	0.2%	0.1%
p Proteobacteria;c Gammaproteobacteria;o Xanthomonadales;f Rhodanobacteraceae;Other;Other	0.0%	0.0%	0.0%
p Proteobacteria;c Gammaproteobacteria;o Xanthomonadales;f Rhodanobacteraceae;g Dokdonella;s uncultured-bacterium	0.0%	0.0%	0.0%
p Proteobacteria;c Gammaproteobacteria;o Xanthomonadales;f Rhodanobacteraceae;g Dyella;Other	0.0%	0.0%	0.0%
p Proteobacteria;c Gammaproteobacteria;o Xanthomonadales;f Rhodanobacteraceae;g Dyella;s uncultured-bacterium	0.0%	0.0%	0.0%
p Proteobacteria;c Gammaproteobacteria;o Xanthomonadales;f Rhodanobacteraceae;g uncultured;Other	0.0%	0.0%	0.0%
p Proteobacteria;c Gammaproteobacteria;o Xanthomonadales;f Rhodanobacteraceae;g uncultured;s uncultured-Xanthomonadaceae-bacterium	0.0%	0.0%	0.0%
p Proteobacteria;c Gammaproteobacteria;o Xanthomonadales;f Rhodanobacteraceae;g uncultured;s uncultured-bacterium	0.0%	0.0%	0.0%
p Proteobacteria;c Gammaproteobacteria;o Xanthomonadales;f Xanthomonadaceae;Other;Other	0.0%	0.0%	0.0%
p Proteobacteria;c Gammaproteobacteria;o Xanthomonadales;f Xanthomonadaceae;g Arenimonas;s uncultured-bacterium	0.1%	0.1%	0.0%
p Proteobacteria;c Gammaproteobacteria;o Xanthomonadales;f Xanthomonadaceae;g Luteimonas;Other	0.0%	0.0%	0.0%
p Proteobacteria;c Gammaproteobacteria;o Xanthomonadales;f Xanthomonadaceae;g Lysobacter;Other	0.2%	0.4%	0.0%
p Proteobacteria;c Gammaproteobacteria;o Xanthomonadales;f Xanthomonadaceae;g Lysobacter;s uncultured-bacterium	0.4%	0.8%	0.0%
p Proteobacteria;c Gammaproteobacteria;o Xanthomonadales;f Xanthomonadaceae;g Pseudoxanthomonas;Other	0.0%	0.0%	0.0%
p Proteobacteria;c Gammaproteobacteria;o Xanthomonadales;f Xanthomonadaceae;g Pseudoxanthomonas;s uncultured-bacterium	0.0%	0.0%	0.0%
p Proteobacteria;c Gammaproteobacteria;o Xanthomonadales;f Xanthomonadaceae;g Stenotrophomonas;Other	0.0%	0.0%	0.0%
p Proteobacteria;c Gammaproteobacteria;o Xanthomonadales;f Xanthomonadaceae;g Thermomonas;s uncultured-bacterium	0.0%	0.0%	0.0%
p Rokubacteria;c NC10;o Rokubacteriales;Other;Other;Other	0.0%	0.0%	0.0%
p Rokubacteria;c NC10;o Rokubacteriales;f uncultured-Gram-positive-bacterium;g uncultured-Gram-positive-bacterium;s uncultured-Gram-positive-bacterium	0.1%	0.1%	0.0%
p Rokubacteria;c NC10;o Rokubacteriales;f uncultured-bacterium;g uncultured-bacterium;s uncultured-bacterium	1.2%	1.5%	0.8%
p Thaumarchaeota;c Group-1.1c;Other;Other;Other;Other	0.0%	0.0%	0.0%
p Thaumarchaeota;c Nitrososphaeria;o Nitrosopumilales;f Nitrosopumilaceae;g Candidatus-Nitrosotenuis;s uncultured-archaeon	0.0%	0.0%	0.0%
p Thaumarchaeota;c Nitrososphaeria;o Nitrososphaerales;f Nitrososphaeraceae;Other;Other	0.5%	0.5%	0.5%
p Thaumarchaeota;c Nitrososphaeria;o Nitrososphaerales;f Nitrososphaeraceae;g Candidatus-Nitrocosmicus;s uncultured-archaeon	0.1%	0.1%	0.1%
p Thaumarchaeota;c Nitrososphaeria;o Nitrososphaerales;f Nitrososphaeraceae;g Candidatus-Nitrososphaera;Other	0.4%	0.4%	0.4%
p Thaumarchaeota;c Nitrososphaeria;o Nitrososphaerales;f Nitrososphaeraceae;g Candidatus-Nitrososphaera;s uncultured-archaeon	0.1%	0.0%	0.1%
p Thaumarchaeota;c Nitrososphaeria;o Nitrososphaerales;f Nitrososphaeraceae;g uncultured-archaeon;s uncultured-archaeon	0.3%	0.3%	0.2%
p Thaumarchaeota;c Nitrososphaeria;o Nitrososphaerales;f Nitrososphaeraceae;g uncultured-bacterium;s uncultured-bacterium	0.0%	0.0%	0.0%
p Thaumarchaeota;c Nitrososphaeria;o Nitrosotaleales;f Nitrosotaleaceae;g uncultured-archaeon;s uncultured-archaeon	0.0%	0.0%	0.0%
p Verrucomicrobia;c Verrucomicrobiae;Other;Other;Other;Other	0.0%	0.0%	0.0%
p Verrucomicrobia;c Verrucomicrobiae;o Chthoniobacterales;Other;Other;Other	0.1%	0.1%	0.1%
p Verrucomicrobia;c Verrucomicrobiae;o Chthoniobacterales;f Chthoniobacteraceae;Other;Other	0.0%	0.0%	0.0%
p Verrucomicrobia;c Verrucomicrobiae;o Chthoniobacterales;f Chthoniobacteraceae;g Candidatus-Udaeobacter;Other	0.5%	0.3%	0.7%
p Verrucomicrobia;c Verrucomicrobiae;o Chthoniobacterales;f Chthoniobacteraceae;g Candidatus-Udaeobacter;s uncultured-Spartobacteria-bacterium	0.0%	0.0%	0.0%
p Verrucomicrobia;c Verrucomicrobiae;o Chthoniobacterales;f Chthoniobacteraceae;g Candidatus-Udaeobacter;s uncultured-bacterium	1.3%	0.7%	1.9%
p Verrucomicrobia;c Verrucomicrobiae;o Chthoniobacterales;f Xiphinematobacteraceae;g Candidatus-Xiphinematobacter;Other	0.0%	0.0%	0.0%
p Verrucomicrobia;c Verrucomicrobiae;o Chthoniobacterales;f Xiphinematobacteraceae;g Candidatus-Xiphinematobacter;s uncultured-Verrucomicrobia-bacterium	0.0%	0.0%	0.0%

	p Verrucomicrobia;c Verrucomicrobiae;o Chthoniobacterales;f Xiphinematobacteraceae;g Candidatus-Xiphinematobacter;s uncultured-bacterium	0.2%	0.3%	0.1%
	p Verrucomicrobia;c Verrucomicrobiae;o Methylacidiphilales;f Methylacidiphilaceae;g uncultured;s candidate-division-WWE3-bacterium-RIFCSPLOWO2_01_FULL_53_14	0.0%	0.0%	0.0%
	p Verrucomicrobia;c Verrucomicrobiae;o Opitutales;f Opitutaceae;Other;Other	1.2%	2.3%	0.1%
	p Verrucomicrobia;c Verrucomicrobiae;o Opitutales;f Opitutaceae;g Lacunisphaera;Other	0.0%	0.0%	0.0%
	p Verrucomicrobia;c Verrucomicrobiae;o Opitutales;f Opitutaceae;g Lacunisphaera;s Opitutus-sp.	0.0%	0.0%	0.0%
	p Verrucomicrobia;c Verrucomicrobiae;o Opitutales;f Opitutaceae;g Lacunisphaera;s uncultured-bacterium	0.0%	0.0%	0.0%
	p Verrucomicrobia;c Verrucomicrobiae;o Opitutales;f Opitutaceae;g Opitutus;s uncultured-bacterium	0.1%	0.1%	0.1%
	p Verrucomicrobia;c Verrucomicrobiae;o Pedosphaerales;f Pedosphaeraceae;Other;Other	1.1%	0.6%	1.5%
	p Verrucomicrobia;c Verrucomicrobiae;o Pedosphaerales;f Pedosphaeraceae;g uncultured-Verrucomicrobia-bacterium;s uncultured-Verrucomicrobia-bacterium	0.0%	0.0%	0.1%
	p Verrucomicrobia;c Verrucomicrobiae;o Pedosphaerales;f Pedosphaeraceae;g uncultured-bacterium;s uncultured-bacterium	1.1%	0.7%	1.5%
	p Verrucomicrobia;c Verrucomicrobiae;o Pedosphaerales;f Pedosphaeraceae;g uncultured-soil-bacterium;s uncultured-soil-bacterium	0.3%	0.2%	0.4%
	p Verrucomicrobia;c Verrucomicrobiae;o Pedosphaerales;f Pedosphaeraceae;g uncultured-subdivision-3-bacterium;s uncultured-subdivision-3-bacterium	0.1%	0.0%	0.1%
	p Verrucomicrobia;c Verrucomicrobiae;o Verrucomicrobiales;Other;Other;Other	0.0%	0.0%	0.0%
	p Verrucomicrobia;c Verrucomicrobiae;o Verrucomicrobiales;f Akkermansiaceae;g Akkermansia;s uncultured-bacterium	0.0%	0.0%	0.0%
	p Verrucomicrobia;c Verrucomicrobiae;o Verrucomicrobiales;f Rubritaleaceae;g Luteolibacter;Other	0.0%	0.0%	0.0%
	p Verrucomicrobia;c Verrucomicrobiae;o Verrucomicrobiales;f Rubritaleaceae;g Luteolibacter;s Haloferula-sp.-BvORR071	0.0%	0.0%	0.0%
	p Verrucomicrobia;c Verrucomicrobiae;o Verrucomicrobiales;f Rubritaleaceae;g Luteolibacter;s uncultured-Verrucomicrobia-bacterium	0.0%	0.0%	0.0%
	p Verrucomicrobia;c Verrucomicrobiae;o Verrucomicrobiales;f Rubritaleaceae;g Luteolibacter;s uncultured-bacterium	0.0%	0.0%	0.0%
	p Verrucomicrobia;c Verrucomicrobiae;o Verrucomicrobiales;f Verrucomicrobiaceae;Other;Other	0.0%	0.0%	0.0%
	p Verrucomicrobia;c Verrucomicrobiae;o Verrucomicrobiales;f Verrucomicrobiaceae;g Verrucomicrobium;Other	0.0%	0.0%	0.0%
	p Verrucomicrobia;c Verrucomicrobiae;o Verrucomicrobiales;f Verrucomicrobiaceae;g uncultured;Other	0.0%	0.0%	0.0%
	p Verrucomicrobia;c Verrucomicrobiae;o Verrucomicrobiales;f Verrucomicrobiaceae;g uncultured;s uncultured-Verrucomicrobia-bacterium	0.0%	0.0%	0.0%
	p Verrucomicrobia;c Verrucomicrobiae;o Verrucomicrobiales;f Verrucomicrobiaceae;g uncultured;s uncultured-bacterium	0.0%	0.0%	0.0%
	p WPS-2;Other;Other;Other;Other;Other	0.0%	0.0%	0.0%
	p Zixibacteria;Other;Other;Other;Other;Other	0.0%	0.0%	0.0%
	p Zixibacteria;c uncultured-bacterium;o uncultured-bacterium;f uncultured-bacterium;g uncultured-bacterium;s uncultured-bacterium	0.0%	0.0%	0.0%
