# Supplementary material for: Bacterial structure and dynamics in mango (Mangifera indica) orchards after long term organic and conventional treatments under subtropical ecosystem
Source: Sci Rep. 2021 Oct 15;11:20554. doi: 10.1038/s41598-021-00112-0 (PMC8519990; doi:10.1038/s41598-021-00112-0)
Supplement: Supplementary file 4 — Supplementary Information 4. [file 41598_2021_112_MOESM4_ESM.doc]

Organic treatment (G1)

| 10 M  10 M  50x50 |  |  |
| --- | --- | --- |

10 M

10 M

50x50

Replicate - I 50m x50m Replicate - II 50m x50m Replicate - III 50m x50m

Conventional treatment (G2)

| 10 M  10 M  50x50 |  |  |
| --- | --- | --- |

Replicate - I 50m x50m Replicate - II 50m x50m Replicate - III 50m x50m

**Fig. S1** Schematic representation of organic and conventional treatments of *Mangifera indica* L. cv Dasehari experimental orchards.

**Table S1.1** Sample details and its quality check.

**Table S1.2.** Samples summary

**Table S1.3**. Raw read summary of the samples

**Table S1.4.** Raw read summary with Phred quality score distribution of the samples.

**Table S1.5.** Base composition distribution of the samples (%)

**Table S1.6.** Trimmed and Consensus read summary.

**Table S1.7.** Pre-processing read statistics of the samples.

**Table S1.8.** Comparative plant growth promotory properties of the bacteria isolated from organic and conventional system.

| **Isolates** | **PGPR tests** | | | | | |
| --- | --- | --- | --- | --- | --- | --- |
| **Organic system**  **(G1)** | **PO_4_** | **K** | **ZnO** | **ZnP** | **ZnCo3** | **Siderophore** |
| 1 | - | - | - | - | - | - |
| **2** | + | + | + | - | + | + |
| **3** | + | + | - | + | - | + |
| **4** | + | + | - | + | - | + |
| 5 | - | - | - | - | - | - |
| **8** | + | + | + | + | + | - |
| 9 | - | - | - | - | - | - |
| 10 | - | - | - | - | - | - |
| 11 | - | - | - | - | - | - |
| 12 | - | - | - | - | - | + |
| **13** | - | - | - | + | + | - |
| **14** | - | - | - | + | + | - |
| **15** | + | - | - | - | - | - |
| 16 | - | - | - | - | - | - |
| 17 | - | - | - | - | - | - |
| 18 | - | - | - | - | - | - |
| 19 | - | - | - | - | - | - |
| 20 | - | - | - | - | - | - |
| 21 | - | - | - | - | - | - |
| 22 | - | - | - | - | - | - |
| 23 | + | - | - | - | - | - |
| 24 | - | - | - | - | - | - |
| 25 | - | - | - | - | - | - |
| 26 | - | - | - | - | - | + |
| 27 | - | - | - | - | - | - |
| 28 | - | - | - | - | - | - |
| 29 | - | - | - | - | - | - |
| 30 | - | - | - | - | - | - |
| 31 | + | - | - | - | - | - |
| 32 | - | - | - | - | - | - |
| 33 | - | - | - | - | - | - |
| 34 | - | - | - | - | - | - |
| 35 | - | - | - | - | - | - |
| 36 | - | - | - | - | - | - |
| 37 | - | - | - | - | - | - |
| 38 | - | - | - | - | - | - |
| 39 | - | - | - | - | - | - |
| **Conventional system (G2)** | | | | | | |
| **I1** | + | + | - | - | - | - |
| **I2** | - | + | - | + | - | + |
| I3 | - | - | - | - | - | - |
| **I6** | - | + | - | - | - | + |
| I7 | - | - | - | - | - | - |
| **I8** | + | + | - | + | - | + |
| **I9** | + | + | - | + | - | + |
| I10 | - | - | - | - | - | - |
| I11 | - | - | - | - | - | - |
| I12 | - | - | - | - | - | - |
| I13 | - | - | - | - | - | - |
| I14 | - | - | - | - | - | - |
| I15 | - | - | - | - | - | - |
| I16 | - | - | - | - | - | - |
| I17 | - | - | - | - | - | - |
| I18 | - | - | - | - | - | - |
| I19 | - | - | - | - | - | - |
| I20 | - | - | - | - | - | - |
| I23 | - | - | - | - | - | - |


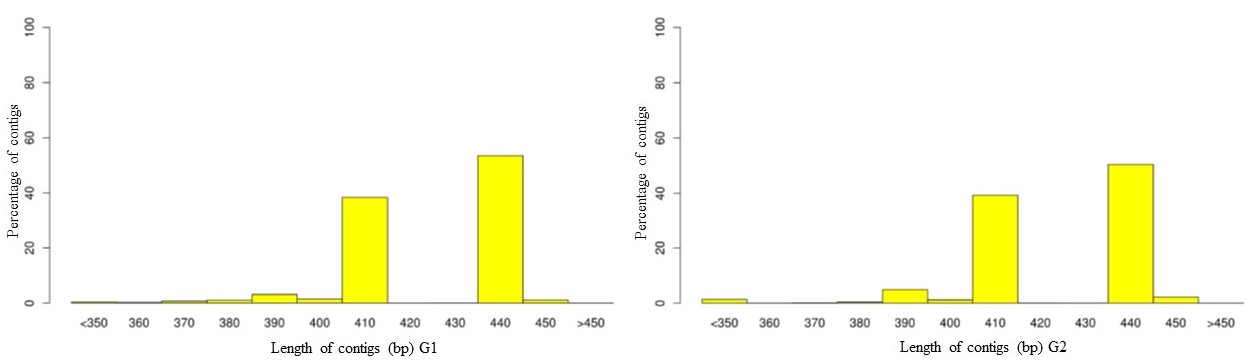


**Figure S2.1.** Contig length distribution for G1 (organic system) and G2 (conventional system).


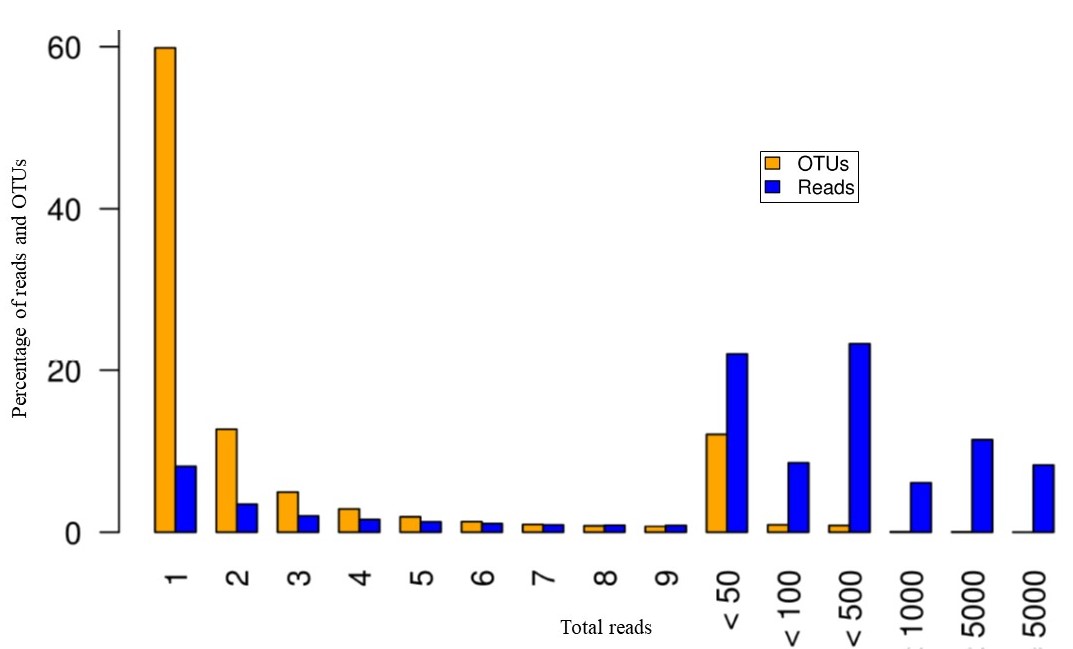


**Figure S2.2.** Reads and OTUs distribution.

**
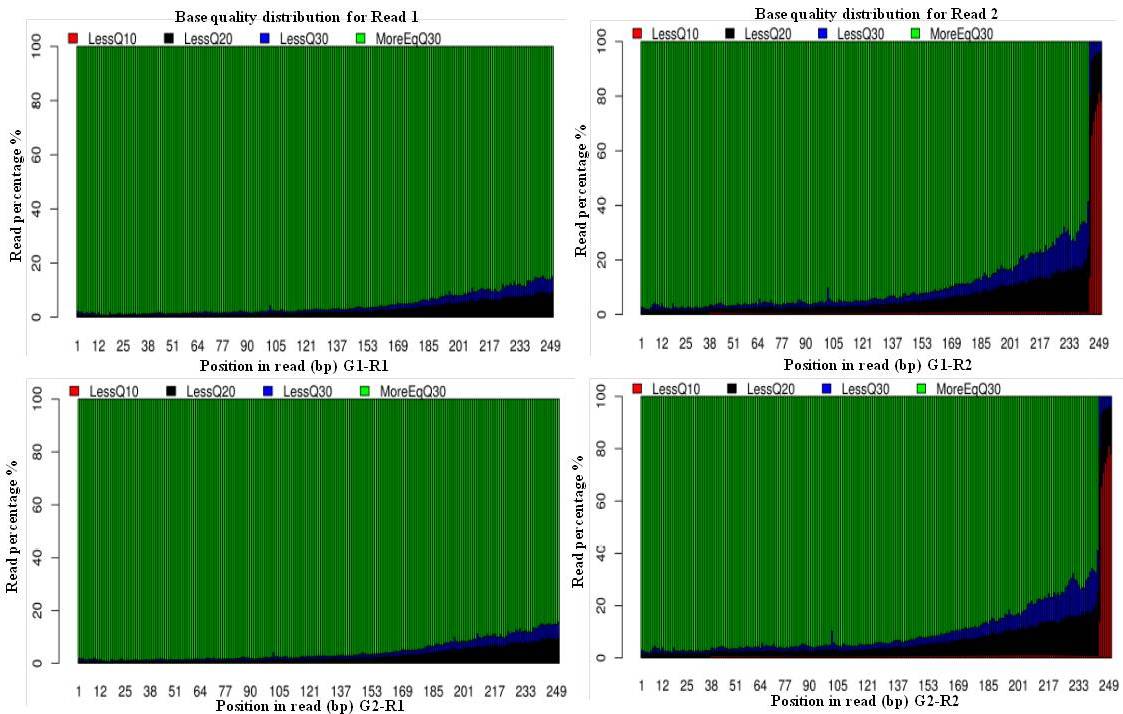
**

**Figure S2.3.** Base quality distribution of G1 and G2 samples.
