# Supplementary material for: Bacterial structure and dynamics in mango (Mangifera indica) orchards after long term organic and conventional treatments under subtropical ecosystem
Source: Sci Rep. 2021 Oct 15;11:20554. doi: 10.1038/s41598-021-00112-0 (PMC8519990; doi:10.1038/s41598-021-00112-0)
Supplement: Supplementary file 2 — Supplementary Information 2. [file 41598_2021_112_MOESM2_ESM.pdf]

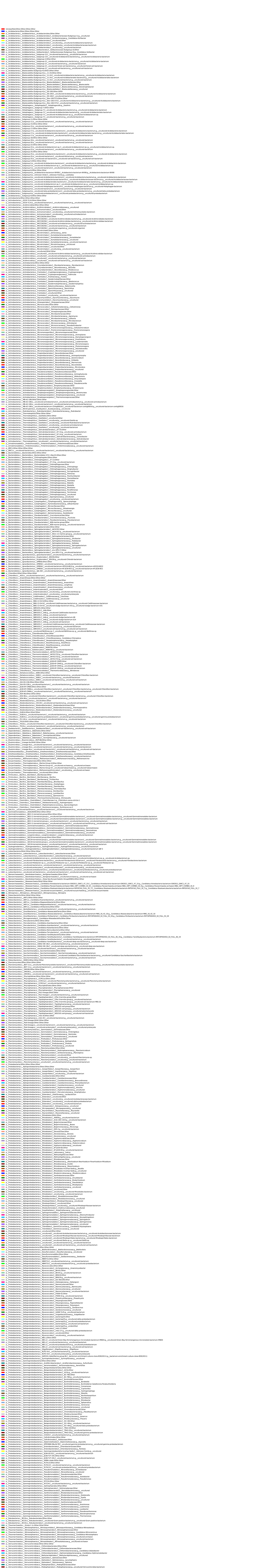
Unclassified;Other;Other;Other;Other
p__Acidobacteria;Other;Other;Other;Other
p__Acidobacteria;c__Acidobacteriia;o__Acidobacteriales;Other;Other
p__Acidobacteria;c__Acidobacteriia;o__Acidobacteriales;f__Acidobacteriaceae-(Subgroup-1);g__uncultured
p__Acidobacteria;c__Acidobacteriia;o__Acidobacteriales;f__Koribacteraceae;g__Candidatus-Koribacter
p__Acidobacteria;c__Acidobacteriia;o__Acidobacteriales;f__uncultured;Other
p__Acidobacteria;c__Acidobacteriia;o__Acidobacteriales;f__uncultured;g__uncultured-Acidobacteria-bacterium
p__Acidobacteria;c__Acidobacteriia;o__Acidobacteriales;f__uncultured;g__uncultured-Acidobacteriaceae-bacterium
p__Acidobacteria;c__Acidobacteriia;o__Acidobacteriales;f__uncultured;g__uncultured-bacterium
p__Acidobacteria;c__Acidobacteriia;o__Solibacterales;f__Solibacteraceae-(Subgroup-3);Other
p__Acidobacteria;c__Acidobacteriia;o__Solibacterales;f__Solibacteraceae-(Subgroup-3);g__Candidatus-Solibacter
p__Acidobacteria;c__Acidobacteriia;o__Subgroup-12;f__uncultured-bacterium;g__uncultured-bacterium
p__Acidobacteria;c__Acidobacteriia;o__Subgroup-13;f__uncultured-Acidobacteria-bacterium;g__uncultured-Acidobacteria-bacterium
p__Acidobacteria;c__Acidobacteriia;o__Subgroup-2;Other;Other
p__Acidobacteria;c__Acidobacteriia;o__Subgroup-2;f__uncultured-Acidobacteria-bacterium;g__uncultured-Acidobacteria-bacterium
p__Acidobacteria;c__Acidobacteriia;o__Subgroup-2;f__uncultured-bacterium;g__uncultured-bacterium
p__Acidobacteria;c__Acidobacteriia;o__Subgroup-2;f__uncultured-forest-soil-bacterium;g__uncultured-forest-soil-bacterium
p__Acidobacteria;c__Acidobacteriia;o__Subgroup-2;f__uncultured-soil-bacterium;g__uncultured-soil-bacterium
p__Acidobacteria;c__Blastocatellia-(Subgroup-4);Other;Other;Other
p__Acidobacteria;c__Blastocatellia-(Subgroup-4);o__11-24;Other;Other
p__Acidobacteria;c__Blastocatellia-(Subgroup-4);o__11-24;f__uncultured-Acidobacteria-bacterium;g__uncultured-Acidobacteria-bacterium
p__Acidobacteria;c__Blastocatellia-(Subgroup-4);o__11-24;f__uncultured-Acidobacteriales-bacterium;g__uncultured-Acidobacteriales-bacterium
p__Acidobacteria;c__Blastocatellia-(Subgroup-4);o__11-24;f__uncultured-bacterium;g__uncultured-bacterium
p__Acidobacteria;c__Blastocatellia-(Subgroup-4);o__Blastocatellales;f__Blastocatellaceae;Other
p__Acidobacteria;c__Blastocatellia-(Subgroup-4);o__Blastocatellales;f__Blastocatellaceae;g__Blastocatella
p__Acidobacteria;c__Blastocatellia-(Subgroup-4);o__Blastocatellales;f__Blastocatellaceae;g__Stenotrophobacter
p__Acidobacteria;c__Blastocatellia-(Subgroup-4);o__Blastocatellales;f__Blastocatellaceae;g__uncultured
p__Acidobacteria;c__Blastocatellia-(Subgroup-4);o__DS-100;Other;Other
p__Acidobacteria;c__Blastocatellia-(Subgroup-4);o__DS-100;f__uncultured-Acidobacteria-bacterium;g__uncultured-Acidobacteria-bacterium
p__Acidobacteria;c__Blastocatellia-(Subgroup-4);o__DS-100;f__uncultured-bacterium;g__uncultured-bacterium
p__Acidobacteria;c__Blastocatellia-(Subgroup-4);o__Elev-16S-573;Other;Other
p__Acidobacteria;c__Blastocatellia-(Subgroup-4);o__Elev-16S-573;f__uncultured-Acidobacteria-bacterium;g__uncultured-Acidobacteria-bacterium
p__Acidobacteria;c__Blastocatellia-(Subgroup-4);o__Elev-16S-573;f__uncultured-bacterium;g__uncultured-bacterium
p__Acidobacteria;c__Holophagae;o__Holophagales;f__Holophagaceae;g__Geothrix
p__Acidobacteria;c__Holophagae;o__Subgroup-7;Other;Other
p__Acidobacteria;c__Holophagae;o__Subgroup-7;f__uncultured-Acidobacteria-bacterium;g__uncultured-Acidobacteria-bacterium
p__Acidobacteria;c__Holophagae;o__Subgroup-7;f__uncultured-Acidobacteriales-bacterium;g__uncultured-Acidobacteriales-bacterium
p__Acidobacteria;c__Holophagae;o__Subgroup-7;f__uncultured-Acidobacterium-sp.;g__uncultured-Acidobacterium-sp.
p__Acidobacteria;c__Holophagae;o__Subgroup-7;f__uncultured-bacterium;g__uncultured-bacterium
p__Acidobacteria;c__Subgroup-11;Other;Other;Other
p__Acidobacteria;c__Subgroup-11;o__uncultured-bacterium;f__uncultured-bacterium;g__uncultured-bacterium
p__Acidobacteria;c__Subgroup-15;Other;Other;Other
p__Acidobacteria;c__Subgroup-15;o__uncultured-bacterium;f__uncultured-bacterium;g__uncultured-bacterium
p__Acidobacteria;c__Subgroup-17;Other;Other;Other
p__Acidobacteria;c__Subgroup-17;o__uncultured-Acidobacteria-bacterium;f__uncultured-Acidobacteria-bacterium;g__uncultured-Acidobacteria-bacterium
p__Acidobacteria;c__Subgroup-17;o__uncultured-Acidobacteriales-bacterium;f__uncultured-Acidobacteriales-bacterium;g__uncultured-Acidobacteriales-bacterium
p__Acidobacteria;c__Subgroup-17;o__uncultured-bacterium;f__uncultured-bacterium;g__uncultured-bacterium
p__Acidobacteria;c__Subgroup-18;Other;Other;Other
p__Acidobacteria;c__Subgroup-18;o__uncultured-bacterium;f__uncultured-bacterium;g__uncultured-bacterium
p__Acidobacteria;c__Subgroup-20;o__uncultured-bacterium;f__uncultured-bacterium;g__uncultured-bacterium
p__Acidobacteria;c__Subgroup-22;Other;Other;Other
p__Acidobacteria;c__Subgroup-22;o__uncultured-Acidobacterium-sp.;f__uncultured-Acidobacterium-sp.;g__uncultured-Acidobacterium-sp.
p__Acidobacteria;c__Subgroup-22;o__uncultured-bacterium;f__uncultured-bacterium;g__uncultured-bacterium
p__Acidobacteria;c__Subgroup-25;Other;Other;Other
p__Acidobacteria;c__Subgroup-25;o__uncultured-Acidobacteria-bacterium;f__uncultured-Acidobacteria-bacterium;g__uncultured-Acidobacteria-bacterium
p__Acidobacteria;c__Subgroup-25;o__uncultured-bacterium;f__uncultured-bacterium;g__uncultured-bacterium
p__Acidobacteria;c__Subgroup-25;o__uncultured-soil-bacterium;f__uncultured-soil-bacterium;g__uncultured-soil-bacterium
p__Acidobacteria;c__Subgroup-5;Other;Other;Other
p__Acidobacteria;c__Subgroup-5;o__uncultured-Acidobacteria-bacterium;f__uncultured-Acidobacteria-bacterium;g__uncultured-Acidobacteria-bacterium
p__Acidobacteria;c__Subgroup-5;o__uncultured-Acidobacterium-sp.;f__uncultured-Acidobacterium-sp.;g__uncultured-Acidobacterium-sp.
p__Acidobacteria;c__Subgroup-5;o__uncultured-bacterium;f__uncultured-bacterium;g__uncultured-bacterium
p__Acidobacteria;c__Subgroup-6;Other;Other;Other
p__Acidobacteria;c__Subgroup-6;o__Acidobacteria-bacterium-WX90;f__Acidobacteria-bacterium-WX90;g__Acidobacteria-bacterium-WX90
p__Acidobacteria;c__Subgroup-6;o__Unknown-Order;f__Unknown-Family;g__Luteitalea
p__Acidobacteria;c__Subgroup-6;o__uncultured-Acidobacteria-bacterium;f__uncultured-Acidobacteria-bacterium;g__uncultured-Acidobacteria-bacterium
p__Acidobacteria;c__Subgroup-6;o__uncultured-Acidobacteriaceae-bacterium;f__uncultured-Acidobacteriaceae-bacterium;g__uncultured-Acidobacteriaceae-bacterium
p__Acidobacteria;c__Subgroup-6;o__uncultured-Acidobacteriales-bacterium;f__uncultured-Acidobacteriales-bacterium;g__uncultured-Acidobacteriales-bacterium
p__Acidobacteria;c__Subgroup-6;o__uncultured-Acidobacterium-sp.;f__uncultured-Acidobacterium-sp.;g__uncultured-Acidobacterium-sp.
p__Acidobacteria;c__Subgroup-6;o__uncultured-Holophagae-bacterium;f__uncultured-Holophagae-bacterium;g__uncultured-Holophagae-bacterium
p__Acidobacteria;c__Subgroup-6;o__uncultured-bacterium;f__uncultured-bacterium;g__uncultured-bacterium
p__Acidobacteria;c__Subgroup-6;o__uncultured-beta-proteobacterium;f__uncultured-beta-proteobacterium;g__uncultured-beta-proteobacterium
p__Acidobacteria;c__Subgroup-6;o__uncultured-proteobacterium;f__uncultured-proteobacterium;g__uncultured-proteobacterium
p__Acidobacteria;c__Subgroup-9;Other;Other;Other
p__Actinobacteria;Other;Other;Other;Other
p__Actinobacteria;c__0319-7L14;Other;Other;Other
p__Actinobacteria;c__0319-7L14;o__uncultured-bacterium;f__uncultured-bacterium;g__uncultured-bacterium
p__Actinobacteria;c__Acidimicrobiia;Other;Other;Other
p__Actinobacteria;c__Acidimicrobiia;o__Acidimicrobiales;f__Acidimicrobiaceae;g__uncultured
p__Actinobacteria;c__Acidimicrobiia;o__Actinomarinales;f__uncultured;Other
p__Actinobacteria;c__Acidimicrobiia;o__Actinomarinales;f__uncultured;g__uncultured-Actinomycetales-bacterium
p__Actinobacteria;c__Acidimicrobiia;o__Actinomarinales;f__uncultured;g__uncultured-actinobacterium
p__Actinobacteria;c__Acidimicrobiia;o__IMCC26256;Other;Other
p__Actinobacteria;c__Acidimicrobiia;o__IMCC26256;f__uncultured-Acidimicrobiales-bacterium;g__uncultured-Acidimicrobiales-bacterium
p__Actinobacteria;c__Acidimicrobiia;o__IMCC26256;f__uncultured-Acidimicrobidae-bacterium;g__uncultured-Acidimicrobidae-bacterium
p__Actinobacteria;c__Acidimicrobiia;o__IMCC26256;f__uncultured-actinobacterium;g__uncultured-actinobacterium
p__Actinobacteria;c__Acidimicrobiia;o__IMCC26256;f__uncultured-bacterium;g__uncultured-bacterium
p__Actinobacteria;c__Acidimicrobiia;o__IMCC26256;f__uncultured-organism;g__uncultured-organism
p__Actinobacteria;c__Acidimicrobiia;o__Microtrichales;Other;Other
p__Actinobacteria;c__Acidimicrobiia;o__Microtrichales;f__Iamiaceae;g__Iamia
p__Actinobacteria;c__Acidimicrobiia;o__Microtrichales;f__Ilumatobacteraceae;Other
p__Actinobacteria;c__Acidimicrobiia;o__Microtrichales;f__Ilumatobacteraceae;g__Ilumatobacter
p__Actinobacteria;c__Acidimicrobiia;o__Microtrichales;f__Ilumatobacteraceae;g__uncultured
p__Actinobacteria;c__Acidimicrobiia;o__Microtrichales;f__Ilumatobacteraceae;g__uncultured-bacterium
p__Actinobacteria;c__Acidimicrobiia;o__Microtrichales;f__Microtrichaceae;g__uncultured
p__Actinobacteria;c__Acidimicrobiia;o__Microtrichales;f__uncultured;Other
p__Actinobacteria;c__Acidimicrobiia;o__Microtrichales;f__uncultured;g__uncultured-bacterium
p__Actinobacteria;c__Acidimicrobiia;o__uncultured;Other;Other
p__Actinobacteria;c__Acidimicrobiia;o__uncultured;f__uncultured-Acidimicrobidae-bacterium;g__uncultured-Acidimicrobidae-bacterium
p__Actinobacteria;c__Acidimicrobiia;o__uncultured;f__uncultured-actinobacterium;g__uncultured-actinobacterium
p__Actinobacteria;c__Acidimicrobiia;o__uncultured;f__uncultured-bacterium;g__uncultured-bacterium
p__Actinobacteria;c__Acidimicrobiia;o__uncultured;f__uncultured-soil-bacterium;g__uncultured-soil-bacterium
p__Actinobacteria;c__Actinobacteria;Other;Other;Other
p__Actinobacteria;c__Actinobacteria;o__Corynebacteriales;f__Mycobacteriaceae;g__Mycobacterium
p__Actinobacteria;c__Actinobacteria;o__Corynebacteriales;f__Nocardiaceae;g__Nocardia
p__Actinobacteria;c__Actinobacteria;o__Corynebacteriales;f__Nocardiaceae;g__Rhodococcus
p__Actinobacteria;c__Actinobacteria;o__Frankiales;f__Cryptosporangiaceae;g__Cryptosporangium
p__Actinobacteria;c__Actinobacteria;o__Frankiales;f__Cryptosporangiaceae;g__Fodinicola
p__Actinobacteria;c__Actinobacteria;o__Frankiales;f__Frankiaceae;g__Frankia
p__Actinobacteria;c__Actinobacteria;o__Frankiales;f__Geodermatophilaceae;Other
p__Actinobacteria;c__Actinobacteria;o__Frankiales;f__Geodermatophilaceae;g__Blastococcus
p__Actinobacteria;c__Actinobacteria;o__Frankiales;f__Geodermatophilaceae;g__Geodermatophilus
p__Actinobacteria;c__Actinobacteria;o__Frankiales;f__Nakamurellaceae;g__Nakamurella
p__Actinobacteria;c__Actinobacteria;o__Frankiales;f__Sporichthyaceae;g__Sporichthya
p__Actinobacteria;c__Actinobacteria;o__Frankiales;f__Sporichthyaceae;g__uncultured
p__Actinobacteria;c__Actinobacteria;o__Frankiales;f__uncultured;Other
p__Actinobacteria;c__Actinobacteria;o__Frankiales;f__uncultured;g__uncultured-bacterium
p__Actinobacteria;c__Actinobacteria;o__Glycomycetales;f__Glycomycetaceae;g__Glycomyces
p__Actinobacteria;c__Actinobacteria;o__Glycomycetales;f__Glycomycetaceae;g__uncultured
p__Actinobacteria;c__Actinobacteria;o__Kineosporiales;f__Kineosporiaceae;Other
p__Actinobacteria;c__Actinobacteria;o__Micrococcales;Other;Other
p__Actinobacteria;c__Actinobacteria;o__Micrococcales;f__Cellulomonadaceae;g__Cellulomonas
p__Actinobacteria;c__Actinobacteria;o__Micrococcales;f__Demequinaceae;Other
p__Actinobacteria;c__Actinobacteria;o__Micrococcales;f__Intrasporangiaceae;Other
p__Actinobacteria;c__Actinobacteria;o__Micrococcales;f__Microbacteriaceae;Other
p__Actinobacteria;c__Actinobacteria;o__Micrococcales;f__Microbacteriaceae;g__Agromyces
p__Actinobacteria;c__Actinobacteria;o__Micrococcales;f__Microbacteriaceae;g__Leifsonia
p__Actinobacteria;c__Actinobacteria;o__Micrococcales;f__Microbacteriaceae;g__Microbacterium
p__Actinobacteria;c__Actinobacteria;o__Micrococcales;f__Micrococcaceae;g__Arthrobacter
p__Actinobacteria;c__Actinobacteria;o__Micrococcales;f__Micrococcaceae;g__Pseudarthrobacter
p__Actinobacteria;c__Actinobacteria;o__Micrococcales;f__Promicromonosporaceae;g__Cellulosimicrobium
p__Actinobacteria;c__Actinobacteria;o__Micrococcales;f__Promicromonosporaceae;g__Promicromonospora
p__Actinobacteria;c__Actinobacteria;o__Micromonosporales;f__Micromonosporaceae;Other
p__Actinobacteria;c__Actinobacteria;o__Micromonosporales;f__Micromonosporaceae;g__Actinoplanes
p__Actinobacteria;c__Actinobacteria;o__Micromonosporales;f__Micromonosporaceae;g__Dactylosporangium
