# Supplementary material for: Bacterial structure and dynamics in mango (Mangifera indica) orchards after long term organic and conventional treatments under subtropical ecosystem
Source: Sci Rep. 2021 Oct 15;11:20554. doi: 10.1038/s41598-021-00112-0 (PMC8519990; doi:10.1038/s41598-021-00112-0)

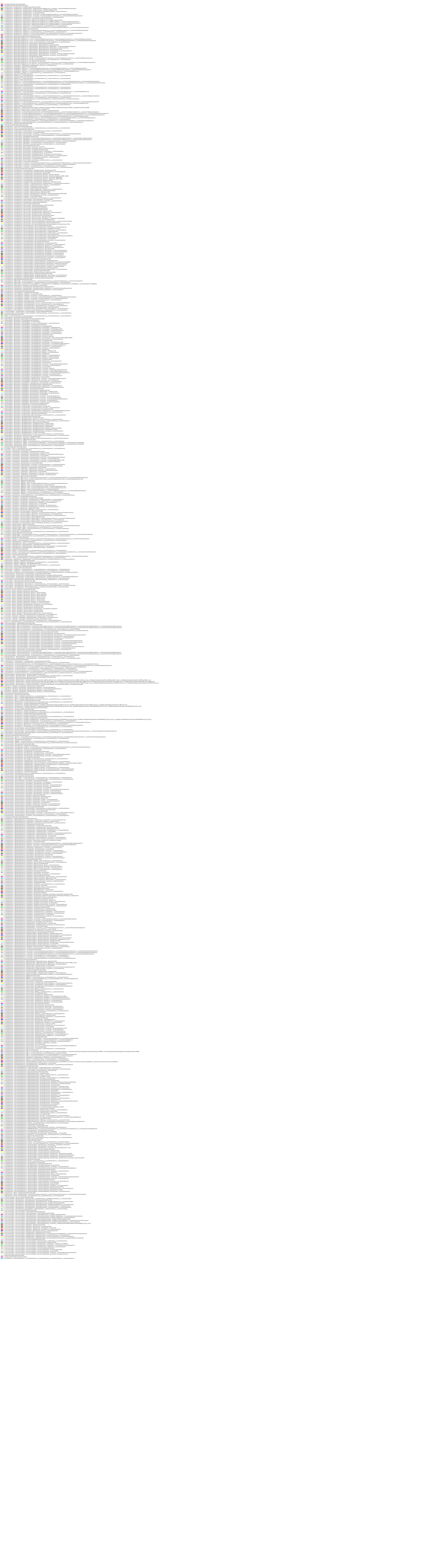

Supplement: Supplementary file 3 — Supplementary Information 3. [file 41598_2021_112_MOESM3_ESM.pdf]
